# Supplementary material for: Exploring genome gene content and morphological analysis to test recalcitrant nodes in the animal phylogeny
Source: PLoS One. 2023 Mar 23;18(3):e0282444. doi: 10.1371/journal.pone.0282444 (PMC10035847; doi:10.1371/journal.pone.0282444)
Supplement: S1 Fig — (PDF) [file pone.0282444.s001.pdf]

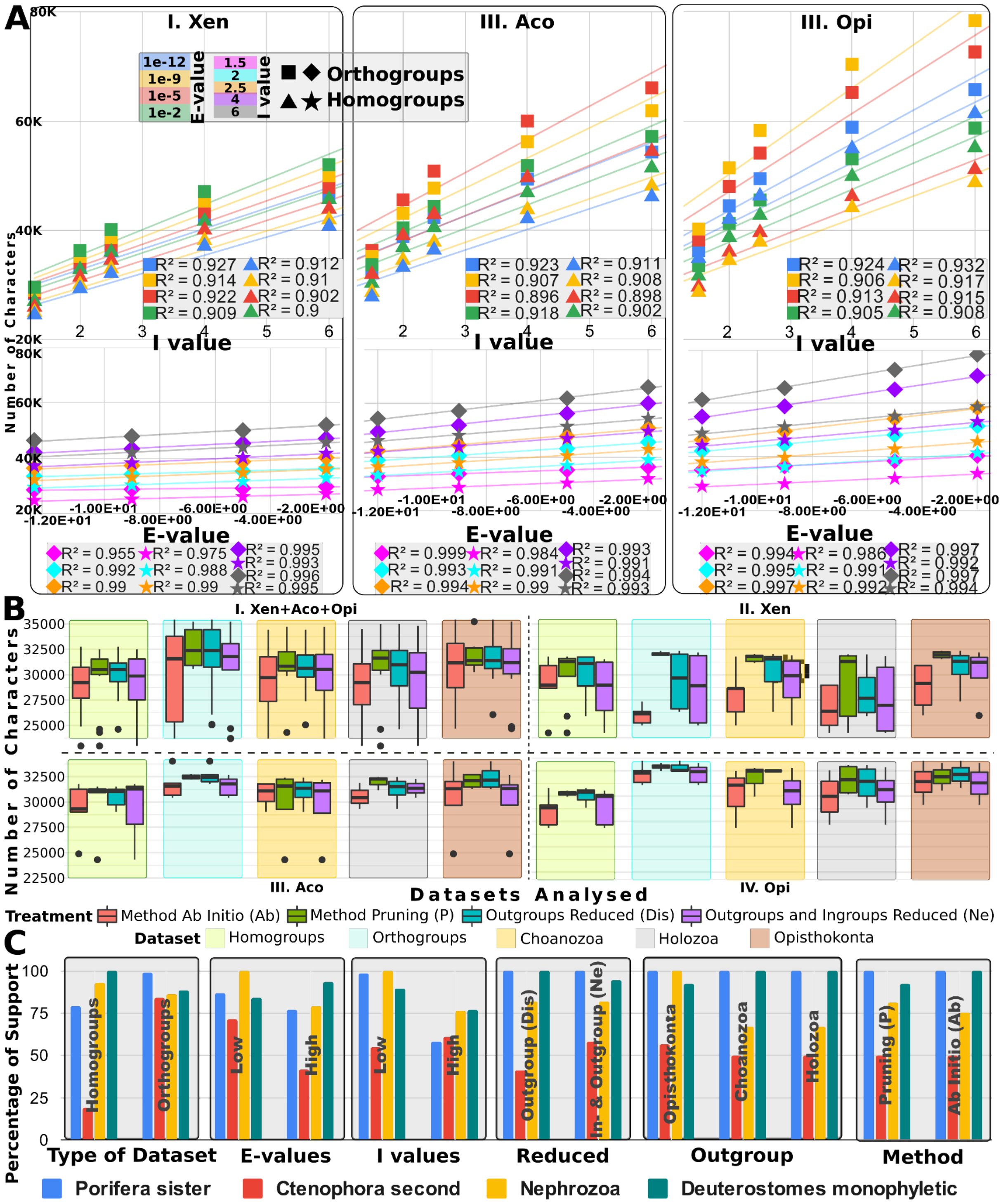

Supplementary Figure 1: Gene content – Number of ortho-/homo- groups and main tree topology supported by each dataset and the summary of Total Posterior evidence trees. The number of ortho-/homo- groups in each of the datasets for phylogenies represented in the scatter plots, box plots and the percentage of support for selected hypotheses are represented in the graphs. Showing the relationship between similarity (E-values) and granulation (I-values) tested on the final number of characters, as well as the outgroup choice and long branches species treatments of the dataset effects.

A. Numbers of characters for each combination of E-value and I-values for the three datasets Opi, Aco and Xen after the prediction of orthogroups and homogroups, trend lines and fit of

R^2 shown (Logarithmic trend line for the I-value graphs, top and linear trend line for the E-values, bottom).
B. The predicted number of characters for each treatment tested in this study for all predicted datasets with E-value 1e-3 and I-value 1.5.
C. The percentage of individual posterior trees supporting each of the tested hypotheses in the different settings of the Opi, Aco, and Xen datasets that were used in this study. Data in Suppl. Table 3.
