# Supplementary material for: Exploring genome gene content and morphological analysis to test recalcitrant nodes in the animal phylogeny
Source: PLoS One. 2023 Mar 23;18(3):e0282444. doi: 10.1371/journal.pone.0282444 (PMC10035847; doi:10.1371/journal.pone.0282444)
Supplement: S4 Fig — (PDF) [file pone.0282444.s004.pdf]

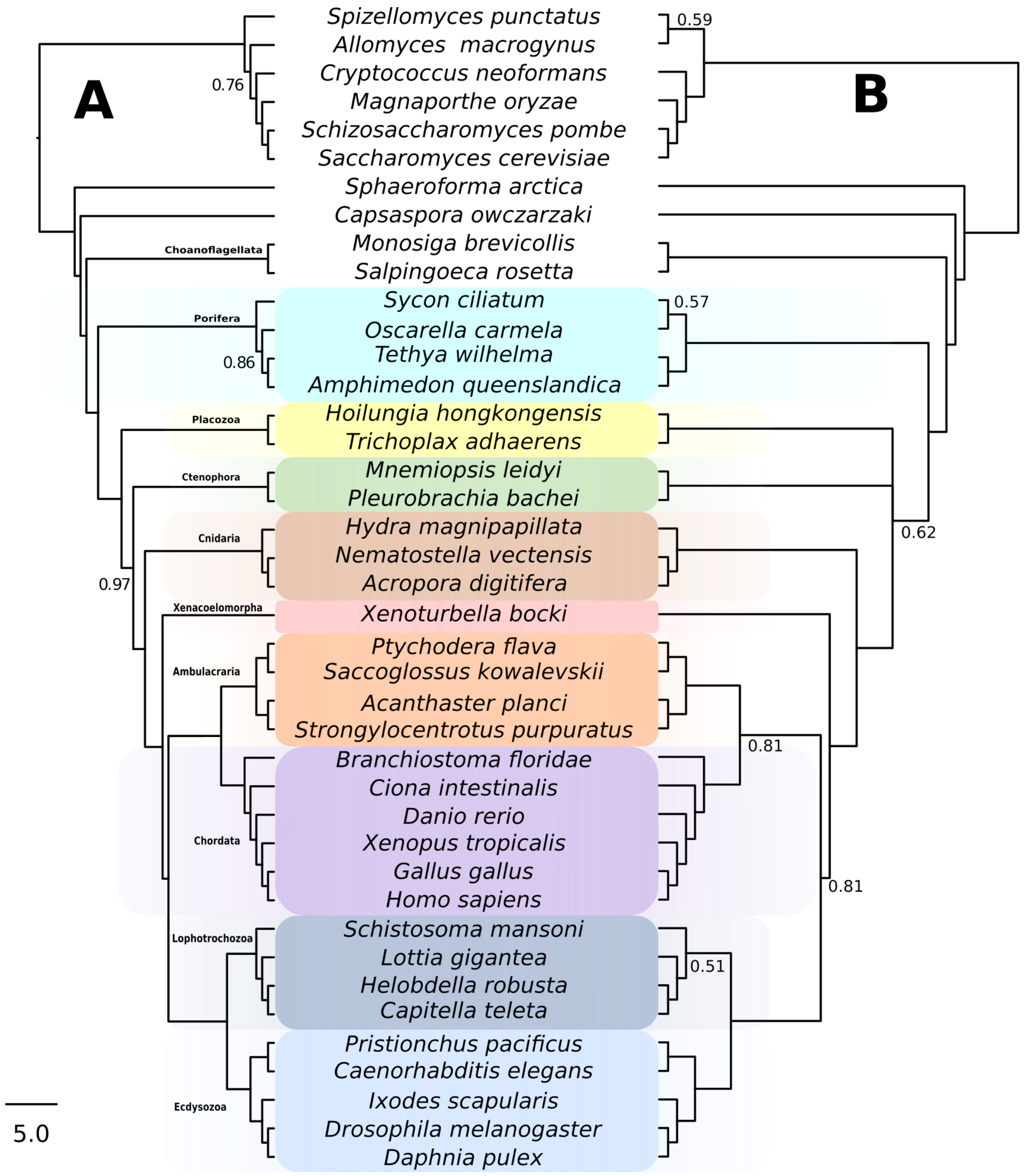

Supplementary Figure 4: **Gene content – Xen Orthogroups (TPCT Xen-ortho) vs Homogroups (TPCT Xen-homo).** A. Phylogeny based on orthogroups gene families predicted for 41 species. B. Phylogeny based on homogroups gene families predicted for 41 species. Each tree represents the consensus tree of 20 analyses (TCPT) performed with combinations of four E-values and five I-values. Each TPCT analysis included samples of trees of all convergent MCMC chains runs of trees for each dataset analysis. The trees are presented as cladograms with proportional branch lengths. Posterior probabilities lower than 0.99 are indicated.
