# Supplementary material for: Exploring genome gene content and morphological analysis to test recalcitrant nodes in the animal phylogeny
Source: PLoS One. 2023 Mar 23;18(3):e0282444. doi: 10.1371/journal.pone.0282444 (PMC10035847; doi:10.1371/journal.pone.0282444)
Supplement: S5 Fig — (PDF) [file pone.0282444.s005.pdf]

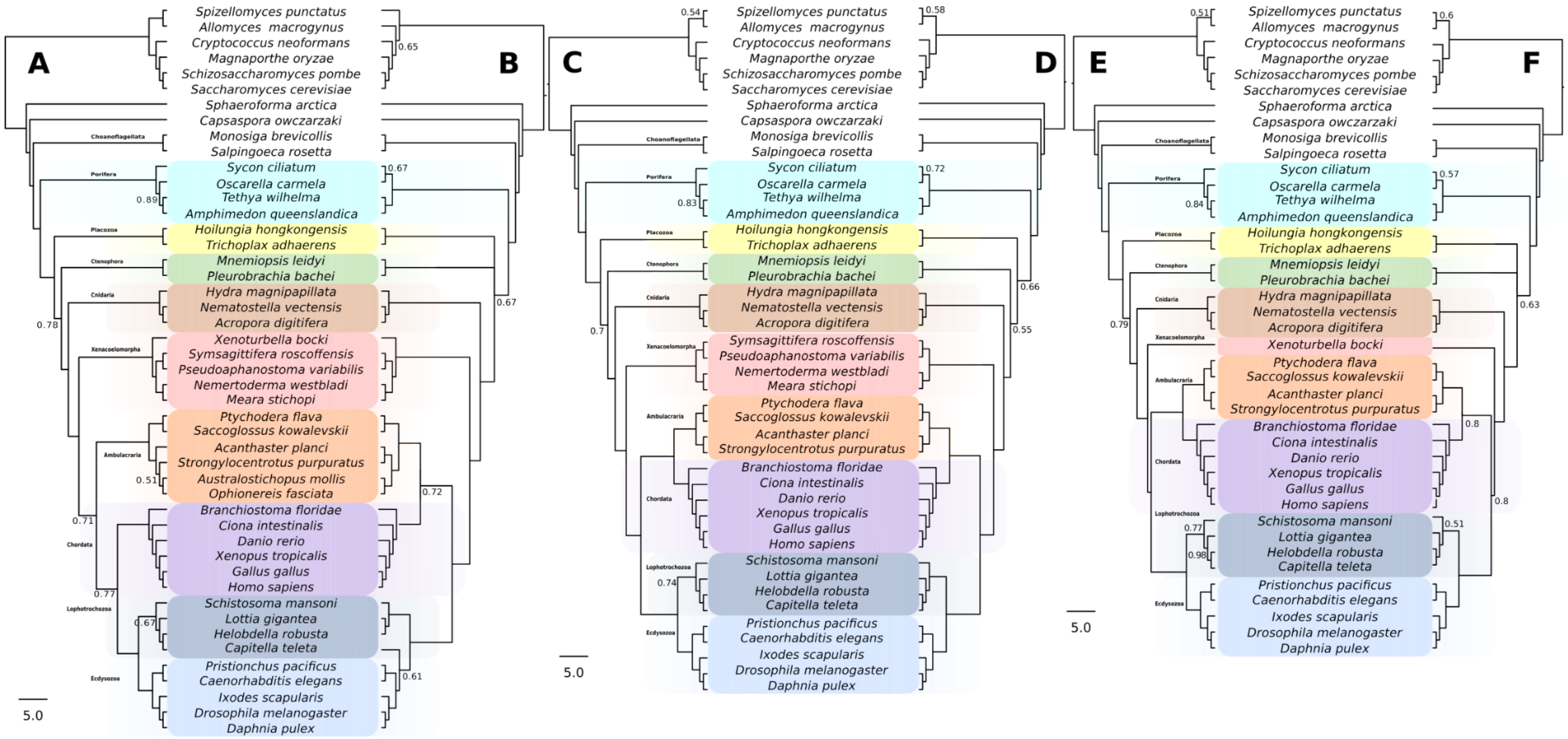

Supplementary Figure 5: **Gene content – TPCT trees second (replicate) run.** A. Phylogeny based on orthogroups gene families predicted for 47 species (Opi-ortho). B. Phylogeny based on homogroups gene families predicted for 47 species (Opi-homo). C. Phylogeny based on orthogroups gene families predicted for 44 species (Aco-ortho). D. Phylogeny based on homogroups gene families predicted for 44 species (Aco-homo). E. Phylogeny based on orthogroups gene families predicted for 41 species (Xen-ortho). F. Phylogeny based on homogroups gene families predicted for 41 species (Xen-homo). Each tree represents the consensus tree of 20 analyses (TCPTree) performed with combinations of four E-values and five I-values. Each tree included MCMC samples of all converged runs for each dataset. The trees are presented as cladograms with proportional branch lengths. Posterior probabilities lower than 0.99 are indicated.
