# Supplementary material for: Exploring genome gene content and morphological analysis to test recalcitrant nodes in the animal phylogeny
Source: PLoS One. 2023 Mar 23;18(3):e0282444. doi: 10.1371/journal.pone.0282444 (PMC10035847; doi:10.1371/journal.pone.0282444)
Supplement: S8 Fig — (PDF) [file pone.0282444.s008.pdf]

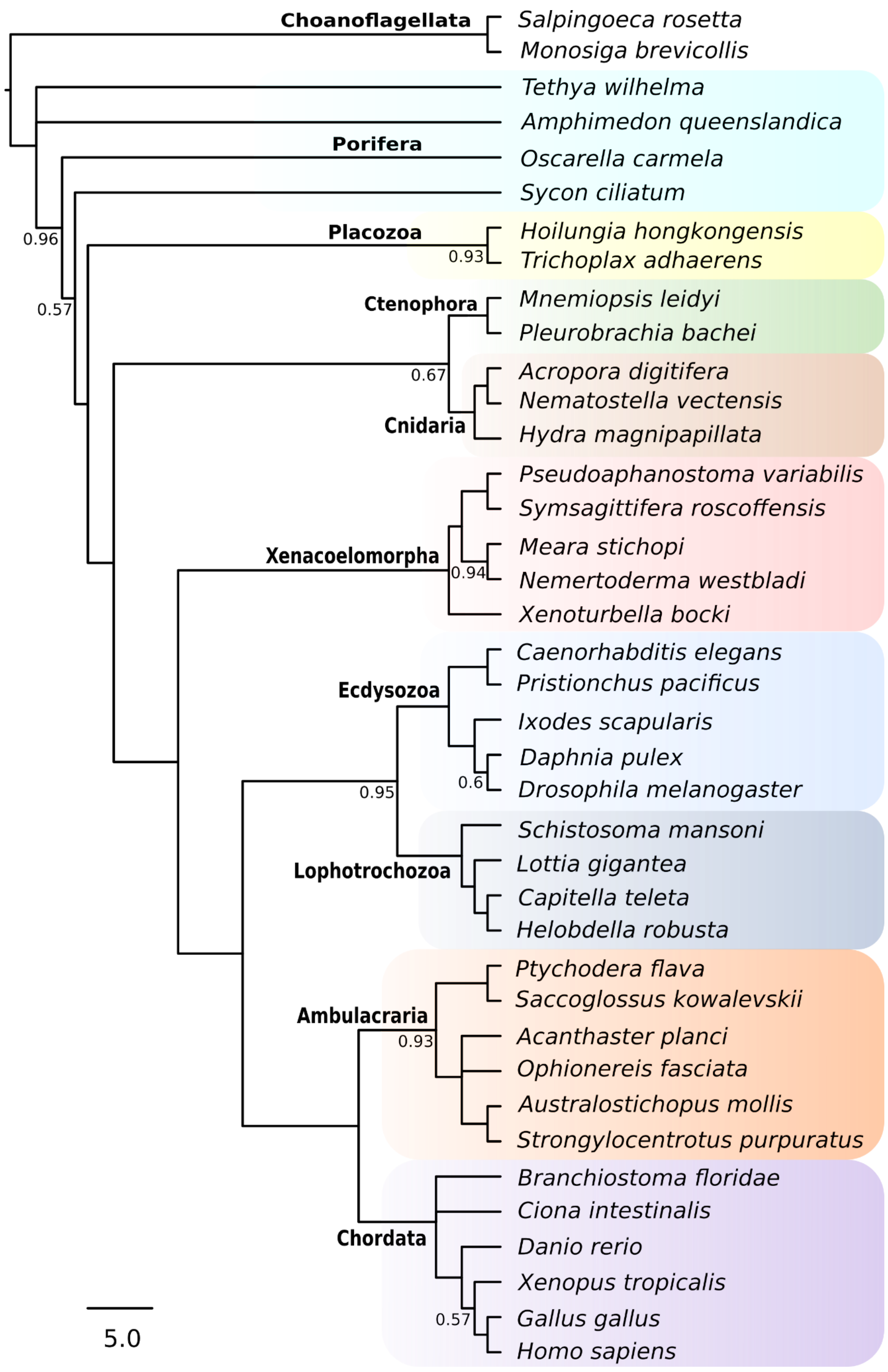

Supplementary Figure 8: Morphology – reductive coding, reduced outgroup sample (Bayesian analysis). Posterior probabilities lower than 0.99 are indicated.
