# Supplementary material for: Exploring genome gene content and morphological analysis to test recalcitrant nodes in the animal phylogeny
Source: PLoS One. 2023 Mar 23;18(3):e0282444. doi: 10.1371/journal.pone.0282444 (PMC10035847; doi:10.1371/journal.pone.0282444)
Supplement: S9 Fig — (PDF) [file pone.0282444.s009.pdf]

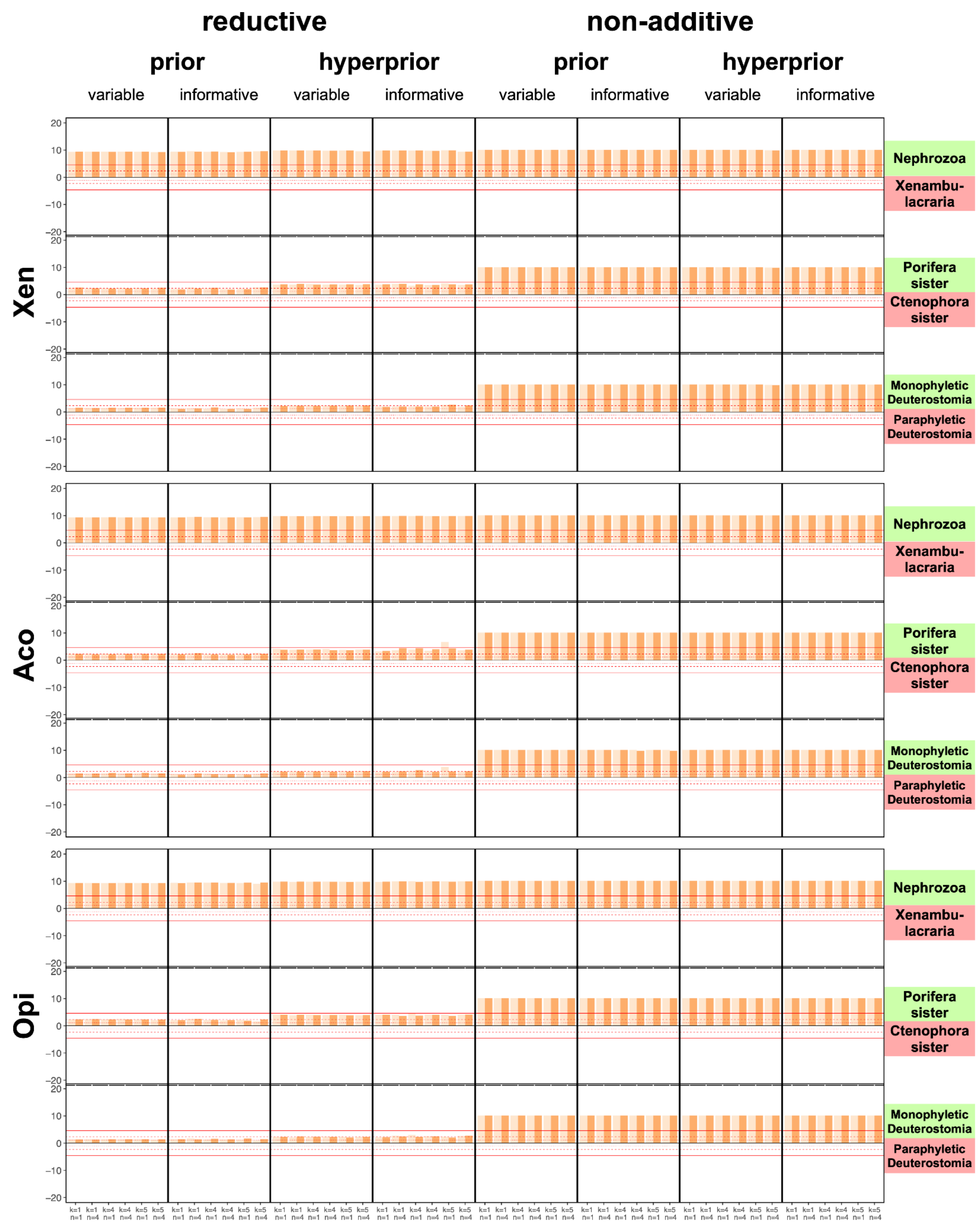

Supplementary Figure 9: Morphology – Statistical hypothesis testing. The two different codings are indicated on the top, the three competing hypotheses tested on the right side, and on the left the taxon sampling. Additionally, at the top the different model assumptions about the branch length prior (fixed prior vs hyperprior) and the ascertainment bias correction (invariant vs parsimony informative) are shown. At the bottom the combinations of the different number of rate categories *n* and number of transition rates *k* in the profile mixture are listed. The results of two replicate chains are indicated in shades of orange. For each tested hypothesis, positive values represent support for hypothesis indicated on the right side by light green squares, and negative values represent support for hypothesis indicated on the right side by light red squares. Interpretation of log-posterior odds was done according to Kass and Raftery [13]. Red lines indicate a very strong support level (5,-5).
