# Supplementary material for: Exploring genome gene content and morphological analysis to test recalcitrant nodes in the animal phylogeny
Source: PLoS One. 2023 Mar 23;18(3):e0282444. doi: 10.1371/journal.pone.0282444 (PMC10035847; doi:10.1371/journal.pone.0282444)
Supplement: S10 Fig — (PDF) [file pone.0282444.s010.pdf]

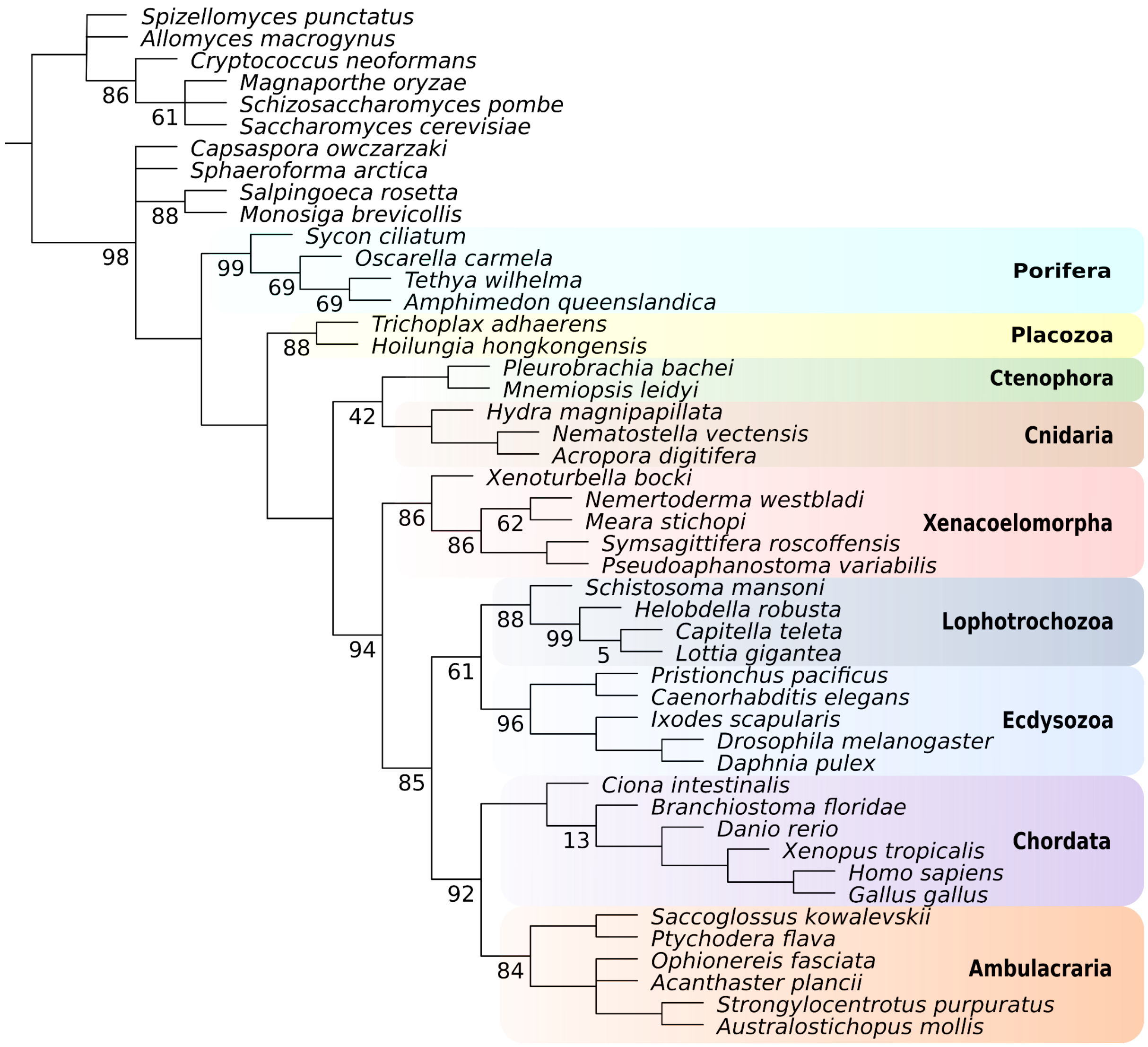

Supplementary Figure 10: Morphology – non-additive coding, full taxon sample (Maximum Parsimony). Bootstrap values lower than 100 are indicated.
