# Supplementary material for: Exploring genome gene content and morphological analysis to test recalcitrant nodes in the animal phylogeny
Source: PLoS One. 2023 Mar 23;18(3):e0282444. doi: 10.1371/journal.pone.0282444 (PMC10035847; doi:10.1371/journal.pone.0282444)
Supplement: S12 Fig — (PDF) [file pone.0282444.s012.pdf]

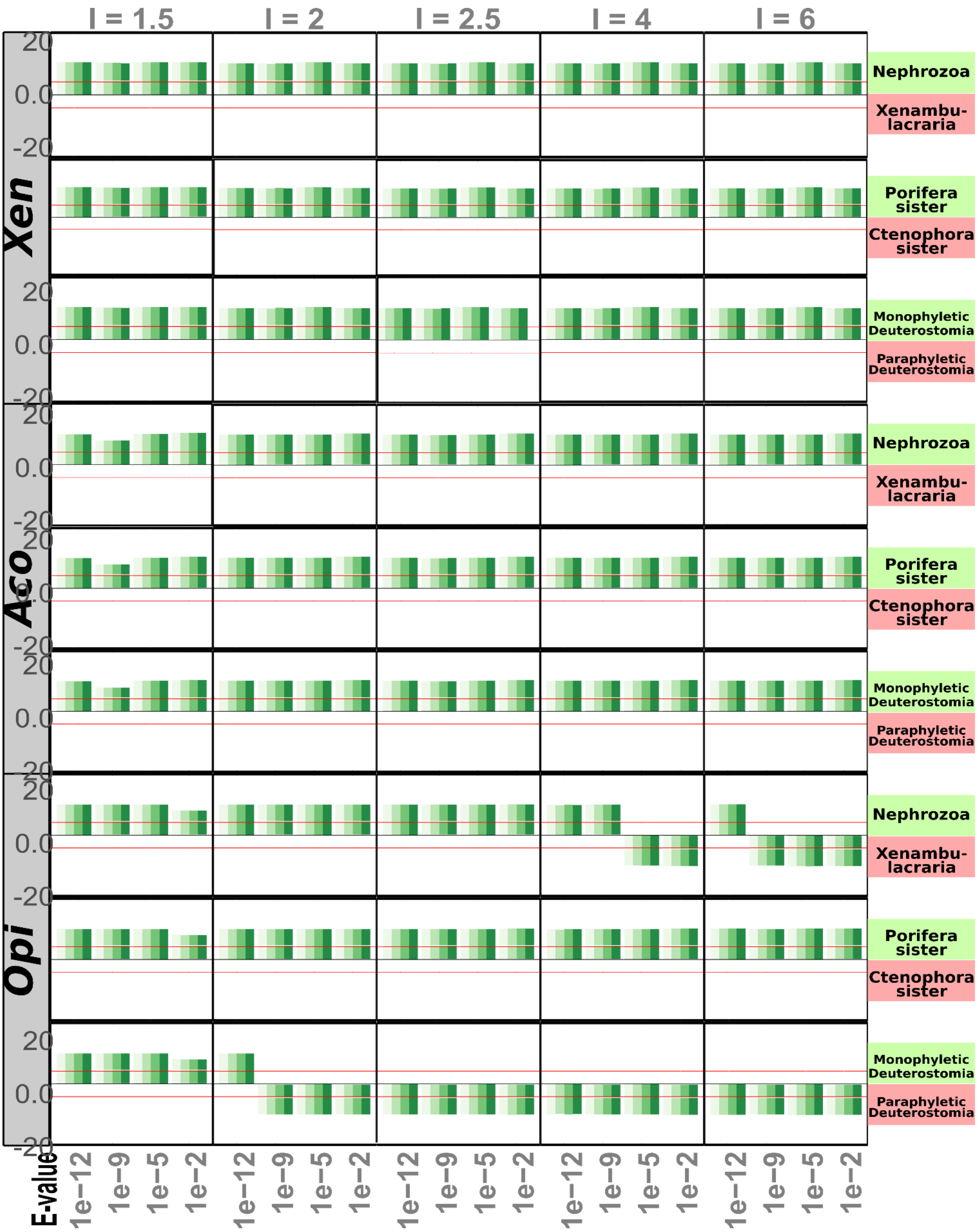

Supplementary Figure 12: Gene Content – Statistical hypothesis testing for the tested topologies in all orthogroups based datasets. The three datasets are indicated on the left side, the three competing hypotheses tested are indicated on the right side. The results of the four replicate chains are indicated in shades of green. I-values are indicated on top and E-values on the bottom. For each tested hypothesis, positive values represent support for hypothesis indicated on the right side by light green squares, and negative values represent support for hypothesis indicated on the right side by light red squares Interpretation of log-posterior odds was done according to Kass and Raftery [13]. Red lines indicate a very strong support level (5,-5).
