# Supplementary material for: Exploring genome gene content and morphological analysis to test recalcitrant nodes in the animal phylogeny
Source: PLoS One. 2023 Mar 23;18(3):e0282444. doi: 10.1371/journal.pone.0282444 (PMC10035847; doi:10.1371/journal.pone.0282444)
Supplement: S14 Fig — (PDF) [file pone.0282444.s014.pdf]

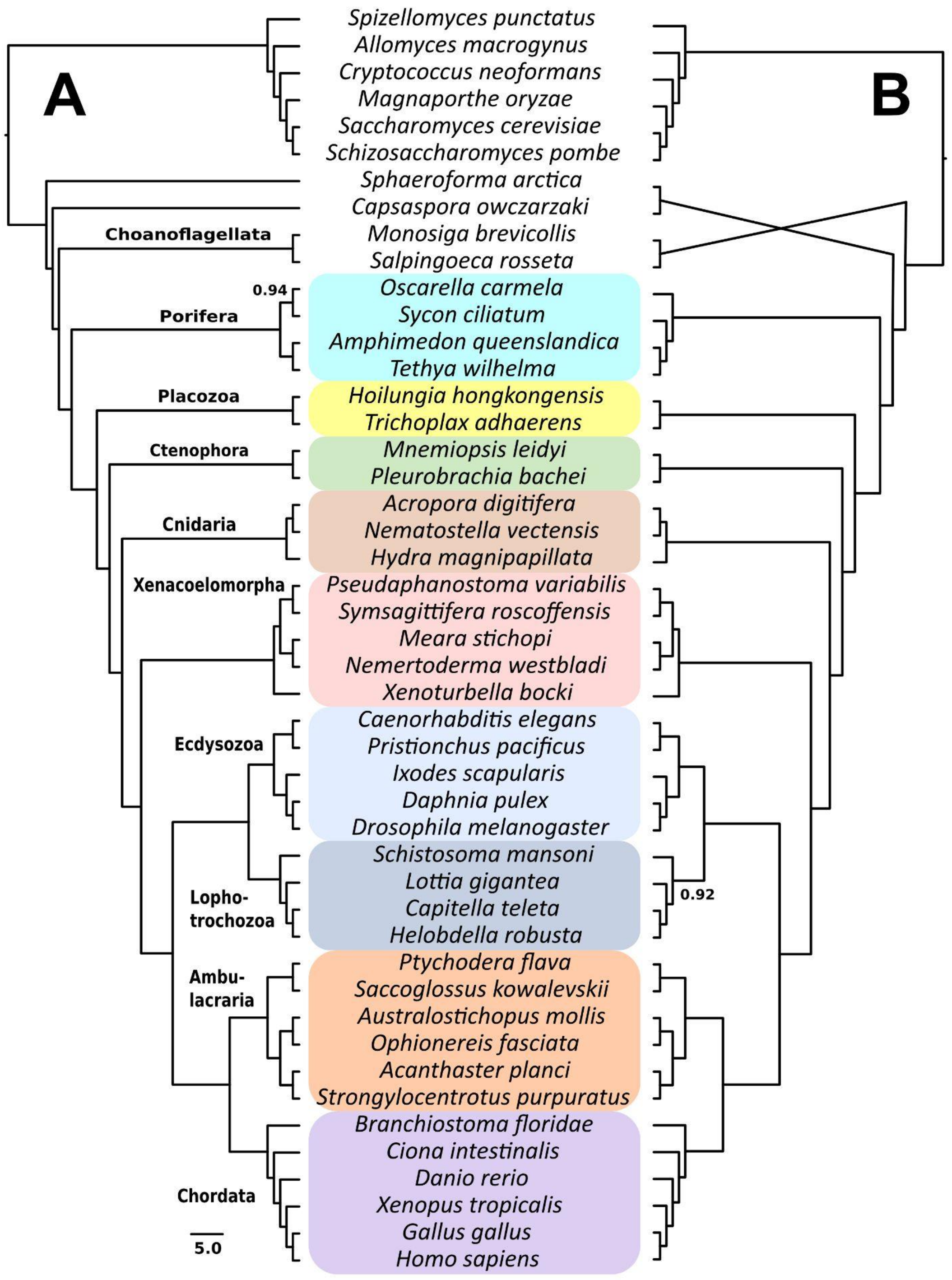

Supplementary Figure 14: "Total Evidence" Phylogeny of the combined gene content and morphological datasets. Opi taxon sampling (47 taxa) with the default methods settings for gene content (I-value of 1.5 and E-value of 1e-3) and the reductive coding morphology dataset. A: orthogroups+morphology, B: homogroups+morphology. Posterior probabilities lower than 0.99 are indicated.
