# Supplementary material for: Exploring genome gene content and morphological analysis to test recalcitrant nodes in the animal phylogeny
Source: PLoS One. 2023 Mar 23;18(3):e0282444. doi: 10.1371/journal.pone.0282444 (PMC10035847; doi:10.1371/journal.pone.0282444)
Supplement: S1 Table — (PDF) [file pone.0282444.s015.pdf]

Supp. Table 1: Information about all the data of the species used. In bold are the 40 core species.

Running number (for internal use only)	Species name	Four letters representation	Phylum	# Phyla	Class	Proteome source	Size (M)	Number of predicted proteins	Included in Aco (44 taxa)	Included in Xen (41 taxa)	BUSCO Eukaryota (255 proteins) C% *min valu	%	te (C)	ɔy (S)	ɘd (D)	ɘd (F)	g (M)	BUSCO Metazoa (954 proteins) C% *min value gray		% te (C)	ɔy (S)	ɘd (D)	ɘd (F)	g (M)
1	***Helobdella robusta***	HERO	Annelida	1	Clitellata	Ensembl	13	22812	Y	Y	97.3	S:96.5%,D:0.8%,F:1.6%,M:1.1%	248	246	2	4	3	90.1	S:89.3%,D:0.8%,F:4.7%,M:5.2%	860	852	8	45	49
2	***Capitella teleta***	CATE	Annelida	1	Polychaeta	Ensembl	17	29584	Y	Y	94.1	S:93.7%,D:0.4%,F:4.3%,M:1.6%	240	239	1	11	4	95	S:93.8%,D:1.2%,F:2.8%,M:2.2%	906	895	11	27	21
3	***Ixodes scapularis***	IXSC	Arthropoda	2	Arachnida	Ensembl	11	20361	Y	Y	72.6	S:71.4%,D:1.2%,F:16.9%,M:10.5%	185	182	3	43	27	78.3	S:77.9%,D:0.4%,F:12.7%,M:9.0%	747	743	4	121	86
4	***Daphnia pulex***	DAPU	Arthropoda	2	Branchiopoda	Ensembl	15	28309	Y	Y	97.7	S:96.5%,D:1.2%,F:0.4%,M:1.9%	249	246	3	1	5	94.9	S:93.5%,D:1.4%,F:1.5%,M:3.6%	905	892	13	14	35
5	***Drosophila melanogaster***	DRME	Arthropoda	2	Insecta	Ensembl	13	15929	Y	Y	100	S:97.6%,D:2.4%,F:0.0%,M:0.0%	255	249	6	0	0	99.3	S:95.7%,D:3.6%,F:0.2%,M:0.5%	947	913	34	2	5
6	***Saccharomyces cerevisiae***	SACE	Ascomycota	3	Saccharomycetes	Ensembl	3.9	6600	Y	Y	93.3	S:92.5%,D:0.8%,F:1.2%,M:5.5%	238	236	2	3	14	39.9	S:35.5%,D:4.4%,F:4.1%,M:56.0%	381	339	42	39	534
7	***Schizosaccharomyces pombe***	SCPO	Ascomycota	3	Schizosaccharomycetes	Ensembl	3.7	5146	Y	Y	96.9	S:95.3%,D:1.6%,F:0.4%,M:2.7%	247	243	4	1	7	49.8	S:46.1%,D:3.7%,F:2.8%,M:47.4%	475	440	35	27	452
8	***Magnaporthe oryzae***	MAOR	Ascomycota	3	Sordariomycetes	Ensembl	8.4	12755	Y	Y	98.5	S:97.3%,D:1.2%,F:0.8%,M:0.7%	251	248	3	2	2	48.9	S:47.5%,D:1.4%,F:4.7%,M:46.4%	466	453	13	45	443
9	***Cryptococcus neoformans var. neoformans JEC21***	CRNE	Basidiomycota	4	Tremellomycetes	Ensembl	4.9	19447	Y	Y	96.9	S:95.7%,D:1.2%,F:2.4%,M:0.7%	247	244	3	6	2	51.1	S:49.8%,D:1.3%,F:4.9%,M:44.0%	487	475	12	47	420
10	***Allomyces macrogynus***	ALMA	Blastocladiomycota	5	Blastocladiomycetes	Ensembl	13	6863	Y	Y	92.1	S:23.9%,D:68.2%,F:3.5%,M:4.4%	235	61	174	9	11	60.1	S:16.6%,D:43.5%,F:4.3%,M:35.6%	573	158	415	41	340
11	***Monosiga brevicollis***	MOBR	Choanozoa	6	Choanoflagellatea	NCBI	7.1	9091	Y	Y	78.8	S:78.8%,D:0.0%,F:7.5%,M:13.7%	201	201	0	19	35	59.7	S:59.3%,D:0.4%,F:6.4%,M:33.9%	570	566	4	61	323
12	***Salpingoeca rosseta***	SARO	Choanozoa	6	Choanoflagellatea	NCBI	10	11731	Y	Y	83.1	S:82.7%,D:0.4%,F:6.7%,M:10.2%	212	211	1	17	26	66.1	S:65.5%,D:0.6%,F:6.9%,M:27.0%	631	625	6	66	257
13	***Danio rerio***	DARE	Chordata	7	Actinopterygii	Ensembl	25	31537	Y	Y	99.3	S:92.2%,D:7.1%,F:0.8%,M:0.1%	253	235	18	2	0	98.1	S:90.3%,D:7.8%,F:0.7%,M:1.2%	935	861	74	7	12
14	***Xenopus tropicalis***	XETR	Chordata	7	Amphibia	Ensembl	17	20388	Y	Y	93.3	S:83.1%,D:10.2%,F:3.9%,M:2.8%	238	212	26	10	7	94.2	S:82.3%,D:11.9%,F:2.1%,M:3.7%	899	785	114	20	35
15	***Ciona intestinalis***	CIIN	Chordata	7	Ascidiacea	Ensembl	8.8	16918	Y	Y	85.5	S:83.1%,D:2.4%,F:7.1%,M:7.4%	218	212	6	18	19	78.7	S:76.8%,D:1.9%,F:6.4%,M:14.9%	751	733	18	61	142
16	***Gallus gallus***	GAGA	Chordata	7	Aves	Ensembl	16	21062	Y	Y	95.7	S:86.7%,D:9.0%,F:2.4%,M:1.9%	244	221	23	6	5	91.8	S:82.6%,D:9.2%,F:2.3%,M:5.9%	876	788	88	22	56
17	***Branchiostoma floridae***	BRFL	Chordata	7	Leptocardii	refSeq	15	27718	Y	Y	87.9	S:87.5%,D:0.4%,F:8.2%,M:3.9%	224	223	1	21	10	91.5	S:89.7%,D:1.8%,F:3.6%,M:4.9%	873	856	17	34	47
18	***Homo sapiens***	HOSA	Chordata	7	Mammalia	Ensembl	32	50304	Y	Y	100	S:71.4%,D:28.6%,F:0.0%,M:0.0%	255	182	73	0	0	100	S:74.6%,D:25.4%,F:0.0%,M:0.0%	954	712	242	0	0
19	***Spizellomyces punctatus DAOM BR117***	SPPU	Chytridiomycota	8	Chytridiomycetes	Ensembl	6.6		Y	Y	94.9	S:93.3%,D:1.6%,F:2.7%,M:2.4%	242	238	4	7	6	70.1	S:66.6%,D:3.5%,F:3.5%,M:26.4%	668	635	33	33	253
20	***Acropora digitifera***	ACDI	Cnidaria	9	Anthozoa	refSeq	19	33878	Y	Y	76	S:63.1%,D:12.9%,F:16.5%,M:7.5%	194	161	33	42	19	77.5	S:63.5%,D:14.0%,F:10.2%,M:12.3%	740	606	134	97	117
21	***Nematostella vectensis***	NEVE	Cnidaria	9	Anthozoa	refSeq	14	15881	Y	Y	93.4	S:91.4%,D:2.0%,F:5.5%,M:1.1%	238	233	5	14	3	89.7	S:87.9%,D:1.8%,F:4.8%,M:5.5%	856	839	17	46	52
22	***Hydra magnipapillata***	HYMA	Cnidaria	9	Hydrozoa	refSeq	13	32714	Y	Y	86.7	S:85.5%,D:1.2%,F:10.2%,M:3.1%	221	218	3	26	8	83.1	S:80.5%,D:2.6%,F:8.9%,M:8.0%	793	768	25	85	76
23	***Mnemiopsis leidyi***	MNLE	Ctenophora	10	Tentaculata	Neurobase	9.8	15881	Y	Y	83.5	S:82.7%,D:0.8%,F:7.8%,M:8.7%	213	211	2	20	22	73.5	S:73.0%,D:0.5%,F:4.9%,M:21.6%	701	696	5	47	206
24	***Pleurobrachia bachei***	PLBA	Ctenophora	10	Tentaculata	Neurobase	22	17190	Y	Y	46.7	S:46.7%,D:0.0%,F:19.2%,M:34.1%	119	119	0	49	87	47.2	S:46.3%,D:0.9%,F:11.3%,M:41.5%	451	442	9	108	395
25	***Acanthaster planci***	ACPL	Echinodermata	11	Asteroidea	refSeq	26	33214	Y	Y	99.6	S:69.8%,D:29.8%,F:0.4%,M:0.0%	254	178	76	1	0	98.8	S:62.6%,D:36.2%,F:0.5%,M:0.7%	942	597	345	5	7
26	***Strongylocentrotus purpuratus***	STPU	Echinodermata	11	Echinoidea	ryanlab	21	26776	Y	Y	86.3	S:82.4%,D:3.9%,F:11.0%,M:2.7%	220	210	10	28	7	87.9	S:83.4%,D:4.5%,F:7.4%,M:4.7%	839	796	43	71	44
27	*Australostichopus mollis*	AUMO	Echinodermata	11	Holothuroidea	ryanlab	6.4	49301	N	N	3.1	S:3.1%,D:0.0%,F:35.3%,M:61.6%	8	8	0	90	157	5.9	S:5.8%,D:0.1%,F:30.6%,M:63.5%	56	55	1	292	606
28	*Ophionereis fasciata*	OPFA	Echinodermata	11	Ophiuroidea	ryanlab	13		N	N	3.9	S:3.5%,D:0.4%,F:36.1%,M:60.0%	10	9	1	92	153	5.2	S:5.0%,D:0.2%,F:36.5%,M:58.3%	50	48	2	348	556
29	***Ptychodera flava***	PTFL	Hemichordata	12	Enteropneusta	Okinawa Institute of Science and Technology Graduate Universit	16	34647	Y	Y	53	S:51.4%,D:1.6%,F:25.1%,M:21.9%	135	131	4	64	56	56.4	S:54.0%,D:2.4%,F:15.3%,M:28.3%	538	515	23	146	270
30	***Saccoglossus kowalevskii***	SAKO	Hemichordata	12	Enteropneusta	Okinawa Institute of Science and Technology Graduate Universit	6.5	22111	Y	Y	87.8	S:84.3%,D:3.5%,F:9.4%,M:2.8%	224	215	9	24	7	90	S:84.4%,D:5.6%,F:7.1%,M:2.9%	858	805	53	68	28
31	***Capsaspora owczarzaki***	CAOW	Ichtyospore	13	Filasterea	NCBI	7.2	8932	Y	Y	93.8	S:91.8%,D:2.0%,F:2.4%,M:3.8%	239	234	5	6	10	74.7	S:72.3%,D:2.4%,F:3.7%,M:21.6%	713	690	23	35	206
32	***Sphaeroforma arctica***	SPAR	Ichtyospore	13	Ichthyophonida	NCBI	6.9	18730	Y	Y	63.1	S:60.0%,D:3.1%,F:23.5%,M:13.4%	161	153	8	60	34	47.5	S:45.4%,D:2.1%,F:14.8%,M:37.7%	453	433	20	141	360
33	***Lottia gigantea***	LOGI	Mollusca	14	Gastropoda	Ensembl	13	23340	Y	Y	96.5	S:95.7%,D:0.8%,F:2.4%,M:1.1%	246	244	2	6	3	96.6	S:95.9%,D:0.7%,F:1.5%,M:1.9%	922	915	7	14	18
34	***Caenorhabditis elegans***	CAEL	Nematoda	15	Chromadorea	Ensembl	14	21607	Y	Y	98.1	S:96.9%,D:1.2%,F:0.8%,M:1.1%	250	247	3	2	3	78.5	S:75.3%,D:3.2%,F:2.1%,M:19.4%	749	718	31	20	185
35	***Pristionchus pacificus***	PRPA	Nematoda	15	Chromadorea	Ensembl	13	28489	Y	Y	55.3	S:54.5%,D:0.8%,F:22.7%,M:22.0%	141	139	2	58	56	54.3	S:52.8%,D:1.5%,F:12.2%,M:33.5%	518	504	14	116	320
36	***Hoilungia hongkongensis***	HOHO	Placozoa	16		Data repository for: Eitel, M. https: //doi.org/10. 1371/journal.pbio. 2005359	26	64753	Y	Y	96.8	S:42.7%,D:54.1%,F:2.7%,M:0.5%	247	109	138	7	1	92.8	S:41.4%,D:51.4%,F:3.0%,M:4.2%	885	395	490	29	40
37	***Trichoplax adhaerens***	TRAD	Placozoa	16		Ensembl	6.9	11349	Y	Y	92.9	S:92.5%,D:0.4%,F:4.7%,M:2.4%	237	236	1	12	6	89.3	S:88.9%,D:0.4%,F:4.6%,M:6.1%	852	848	4	44	58
38	***Schistosoma mansoni***	SCMA	Platyhelminthes	17	Trematoda	Ensembl	7.8	11774	Y	Y	87.5	S:80.8%,D:6.7%,F:6.7%,M:5.8%	223	206	17	17	15	70.9	S:63.7%,D:7.2%,F:6.5%,M:22.6%	677	608	69	62	215
39	***Sycon ciliatum***	SYCI	Porifera	18	Calcarea	Link Email https: //datadryad. org/resource/doi: 10.5061/dryad. tn0f3/1	57.3	50731	Y	Y	97.3	S:91.0%,D:6.3%,F:2.0%,M:0.7%	248	232	16	5	2	90.5	S:81.6%,D:8.9%,F:2.2%,M:7.3%	863	778	85	21	70
40	***Amphimedon queenslandica***	AMQU	Porifera	18	Demospongiae	Ensembl	22	43615	Y	Y	92.5	S:88.2%,D:4.3%,F:7.1%,M:0.4%	236	225	11	18	1	89.9	S:84.4%,D:5.5%,F:4.2%,M:5.9%	857	805	52	40	57
41	***Tethya wilhelma***	TEWI	Porifera	18	Demospongiae	Lab souece	14	26225	Y	Y	67.5	S:65.1%,D:2.4%,F:16.9%,M:15.6%	172	166	6	43	40	66.1	S:63.9%,D:2.2%,F:13.6%,M:20.3%	631	610	21	130	193
42	***Oscarella carmela***	OSCA	Porifera	18	Homoscleromorpha	compagen	4.6	11152	Y	Y	45.1	S:44.3%,D:0.8%,F:22.4%,M:32.5%	115	113	2	57	83	41.3	S:41.0%,D:0.3%,F:14.5%,M:44.2%	394	391	3	138	422
43	*Meara stichopi*	MEST	Xenacoelomorpha	19	Acoelomorpha	figshare	20	130113	Y	N	35.3	S:34.9%,D:0.4%,F:28.6%,M:36.1%	90	89	1	73	92	34.1	S:33.6%,D:0.5%,F:22.5%,M:43.4%	326	321	5	215	413
44	*Nemertoderma westbladi*	NEWE	Xenacoelomorpha	19	Acoelomorpha	figshare	17	80966	Y	N	62	S:60.8%,D:1.2%,F:24.7%,M:13.3%	158	155	3	63	34	64.2	S:62.7%,D:1.5%,F:15.4%,M:20.4%	612	598	14	147	195
45	*Pseudaphanostoma variabilis*	PSVA	Xenacoelomorpha	19	Acoelomorpha	figshare	21	115245	Y	N	34.9	S:33.7%,D:1.2%,F:38.0%,M:27.1%	89	86	3	97	69	35.7	S:34.8%,D:0.9%,F:24.8%,M:39.5%	341	332	9	237	376
46	*Symsagittifera roscoffensis*	SYRO	Xenacoelomorpha	19	Acoelomorpha	figshare	24	113993	Y	N	45.1	S:43.9%,D:1.2%,F:28.2%,M:26.7%	115	112	3	72	68	45.7	S:43.6%,D:2.1%,F:19.0%,M:35.3%	436	416	20	181	337
47	*Xenoturbella bocki*	XEBO	Xenacoelomorpha	19	Xenoturbellidae	figshare	8.7	24134	N	Y	44.3	S:43.9%,D:0.4%,F:29.4%,M:26.3%	113	112	1	75	67	46.5	S:45.8%,D:0.7%,F:22.3%,M:31.2%	444	437	7	213	297
	MIN BUSCO										*3.1*						*0*	*5.2*						*0*
	MAX BUSCO										*100*						*157*	*100*						*606*
	AVERAGE BUSCO										*78.4*						*27.4*	*70.0*						*204.9*
	MEADIAN BUSCO										*87.9*						*10*	*74.7*						*193*
