# Supplementary material for: Exploring genome gene content and morphological analysis to test recalcitrant nodes in the animal phylogeny
Source: PLoS One. 2023 Mar 23;18(3):e0282444. doi: 10.1371/journal.pone.0282444 (PMC10035847; doi:10.1371/journal.pone.0282444)
Supplement: S2 Table — (PDF) [file pone.0282444.s016.pdf]

| |
|---|
| ,,,,,,,,Runs parameters:,,,,,,,,,,,,,,,,effsize,effsize,effsize,effsize,effsize,effsize,effsize,effsize,effsize,effsize,rel_diff,rel_diff,rel_diff,rel_diff,rel_diff,rel_diff,rel_diff,rel_diff,rel_diff,rel_diff,,,Tree,Collapsed tree,Collapsed tree short format,Smallest pp value in tree,plase of smallest pp,The main topologies found,,,,,,,,,,,,,,,,,,,,,,,,,,,,,,,,,,,,,,,,,,,,,,,,,,,,,,,,,,,,,,,,,,,, |
| Type,File name in Table 4,E-value,I-value,nchar,File name in repository ,SP #,Cahnis,First name used,,,,,,,,,,,,,,,,maxdiff,meandiff,Prior,alpha,gamma_rates[1],gamma_rates[2],gamma_rates[3],gamma_rates[4],mu,pi[1],pi[2],rec_mu,Prior,alpha,gamma_rates[1],gamma_rates[2],gamma_rates[3],gamma_rates[4],mu,pi[1],pi[2],rec_mu,maxdiff,Smallest ESS value,,,,,,"(PO,(CT,(PL,((XEBO,(PR,(CH,AM))),CN)));","(PO,(CT,(PL,((AC,(PR,(CH,AM))),CN)));","(PO,( |
| Homogroups,41,1.00E-12,1.5,24545,cluster.41_Species_homogroups_Diamond.1e-12.all_vs_all.txt.1.5.mcl.table.fasta.nexus,41,,,cluster.41_Species_homogroups_Diamond.1e-12.all_vs_all.txt.1.5.mcl.table.fasta_run_1.trees.tree,:,80001,trees,cluster.41_Species_homogroups_Diamond.1e-12.all_vs_all.txt.1.5.mcl.table.fasta_run_2.trees.tree,:,80001,trees,cluster.41_Species_homogroups_Diamond.1e-12.all_vs_all.txt.1.5.mcl.table.fasta_run_3.trees.tree,: |
| Homogroups,44,1.00E-12,1.5,27784,cluster.44_Species_homogroups_Diamond.1e-12.all_vs_all.txt.1.5.mcl.table.fasta.nexus,44,,,cluster.44_Species_homogroups_Diamond.1e-12.all_vs_all.txt.1.5.mcl.table.fasta_run_1.trees.tree,:,80001,trees,cluster.44_Species_homogroups_Diamond.1e-12.all_vs_all.txt.1.5.mcl.table.fasta_run_2.trees.tree,:,80001,trees,cluster.44_Species_homogroups_Diamond.1e-12.all_vs_all.txt.1.5.mcl.table.fasta_run_3.trees.tree,: |
| Homogroups,47,1.00E-12,1.5,28679,cluster.47_Species_homogroups_Diamond.1e-12.all_vs_all.txt.1.5.mcl.table.fasta.nexus,47,,,cluster.47_Species_homogroups_Diamond.1e-12.all_vs_all.txt.1.5.mcl.table.fasta_run_1.trees.tree,:,80001,trees,cluster.47_Species_homogroups_Diamond.1e-12.all_vs_all.txt.1.5.mcl.table.fasta_run_2.trees.tree,:,80001,trees,cluster.47_Species_homogroups_Diamond.1e-12.all_vs_all.txt.1.5.mcl.table.fasta_run_3.trees.tree,: |
| Homogroups,41,1.00E-09,1.5,24996,cluster.41_Species_homogroups_Diamond.1e-9.all_vs_all.txt.1.5.mcl.table.fasta.nexus,41,,,cluster.41_Species_homogroups_Diamond.1e-9.all_vs_all.txt.1.5.mcl.table.fasta_run_1.trees.tree,:,80001,trees,cluster.41_Species_homogroups_Diamond.1e-9.all_vs_all.txt.1.5.mcl.table.fasta_run_2.trees.tree,:,80001,trees,cluster.41_Species_homogroups_Diamond.1e-9.all_vs_all.txt.1.5.mcl.table.fasta_run_3.trees.tree,:,8000 |
| Homogroups,44,1.00E-09,1.5,28599,cluster.44_Species_homogroups_Diamond.1e-9.all_vs_all.txt.1.5.mcl.table.fasta.nexus,44,1_2_3,,cluster.44_Species_homogroups_Diamond.1e-9.all_vs_all.txt.1.5.mcl.table.fasta_run_1.trees.tree,:,80001,trees,cluster.44_Species_homogroups_Diamond.1e-9.all_vs_all.txt.1.5.mcl.table.fasta_run_2.trees.tree,:,80001,trees,cluster.44_Species_homogroups_Diamond.1e-9.all_vs_all.txt.1.5.mcl.table.fasta_run_3.trees.tree |
| Homogroups,47,1.00E-09,1.5,29656,cluster.47_Species_homogroups_Diamond.1e-9.all_vs_all.txt.1.5.mcl.table.fasta.nexus,47,,,cluster.47_Species_homogroups_Diamond.1e-9.all_vs_all.txt.1.5.mcl.table.fasta_run_1.trees.tree,:,80001,trees,cluster.47_Species_homogroups_Diamond.1e-9.all_vs_all.txt.1.5.mcl.table.fasta_run_2.trees.tree,:,80001,trees,cluster.47_Species_homogroups_Diamond.1e-9.all_vs_all.txt.1.5.mcl.table.fasta_run_3.trees.tree,:,8000 |
| Homogroups,41,1.00E-05,1.5,25934,cluster.41_Species_homogroups_Diamond.1e-5.all_vs_all.txt.1.5.mcl.table.fasta.nexus,41,,,cluster.41_Species_homogroups_Diamond.1e-5.all_vs_all.txt.1.5.mcl.table.fasta_run_1.trees.tree,:,80001,trees,cluster.41_Species_homogroups_Diamond.1e-5.all_vs_all.txt.1.5.mcl.table.fasta_run_2.trees.tree,:,80001,trees,cluster.41_Species_homogroups_Diamond.1e-5.all_vs_all.txt.1.5.mcl.table.fasta_run_3.trees.tree,:,8000 |
| Homogroups,44,1.00E-05,1.5,30267,cluster.44_Species_homogroups_Diamond.1e-5.all_vs_all.txt.1.5.mcl.table.fasta.nexus,44,,,cluster.44_Species_homogroups_Diamond.1e-5.all_vs_all.txt.1.5.mcl.table.fasta_run_1.trees.tree,:,80001,trees,cluster.44_Species_homogroups_Diamond.1e-5.all_vs_all.txt.1.5.mcl.table.fasta_run_2.trees.tree,:,80001,trees,cluster.44_Species_homogroups_Diamond.1e-5.all_vs_all.txt.1.5.mcl.table.fasta_run_3.trees.tree,:,8000 |
| Homogroups,47,1.00E-05,1.5,31634,cluster.47_Species_homogroups_Diamond.1e-5.all_vs_all.txt.1.5.mcl.table.fasta.nexus,47,,,cluster.47_Species_homogroups_Diamond.1e-5.all_vs_all.txt.1.5.mcl.table.fasta_run_1.trees.tree,:,80001,trees,cluster.47_Species_homogroups_Diamond.1e-5.all_vs_all.txt.1.5.mcl.table.fasta_run_2.trees.tree,:,80001,trees,cluster.47_Species_homogroups_Diamond.1e-5.all_vs_all.txt.1.5.mcl.table.fasta_run_3.trees.tree,:,8000 |
| Homogroups,OpiXen-homo-disAb41,1.00E-03,1.5,26575,cluster.All_ntax41_homogroups_Diamond_1e-3_all_vs_all.txt.1.5.mcl.table.fasta.nexus,41,,A1ntax41,Homogroups_A_1_ntax41_run_1.trees.tree,80001,Homogroups_A_1_ntax41_run_2.trees.tree,80001,Homogroups_A_1_ntax41_run_3.trees.tree,80001,Homogroups_A_1_ntax41_run_4.trees.tree,80001,0.06,0.000672826,693,693,0.0132921,1315,0.00961638,1298,0.00943689,1356,0.0107764,1 |
| Homogroups,OpiAco-homo-disAb44,1.00E-03,1.5,31284,cluster.All_ntax44_homogroups_Diamond_1e-3_all_vs_all.txt.1.5.mcl.table.fasta.nexus,44,,A1ntax44,/home/kjurave/revBayes_run/data/ZIP/Homogroups_A_1_ntax44,,,,,,,,,,,,,,,,,,,,,,,,,,,,,,,,,,,,,,,,,,,,,,,,,,,,,,,,,,,,,,,,,,,,, |
| Homogroups,Opi-homo-disAb47,1.00E-03,1.5,32795,cluster.All_ntax47_homogroups_Diamond_1e-3_all_vs_all.txt.1.5.mcl.table.fasta.nexus,47,1_3_4,A1ntax47,Homogroups_A_ntax47_run_1.trees.tree,80001,Homogroups_A_ntax47_run_3.trees.tree,80001,Homogroups_A_ntax47_run_4.trees.tree,80001,,,0.0310746,0.000242307,767,767,0.00112049,1113,0.00992693,1099,0.00990654,1188,0.0102909,1280,0.0105405,1190,0.0102893,80001,0,1039,0 |
| Homogroups,HolXen-homo-disAb41,1.00E-03,1.5,24618,Homogroups_A2ntax35.nexus,35,,A2ntax35,,,,,,,,,,,,,,,,,,,,,,,,,,,,,,,,,,,,,,,,,,,,,,,,,,,,,,,,,,,,,,,,,,,,,,,,,,,,,,,,,,,,,,,,,,,,,,,,,,,,,,,,,,, |
| Homogroups,HolAco-homo-disAb38,1.00E-03,1.5,29318,cluster.All_A2ntax38_homogroups_Diamond_1e-3_all_vs_all.txt.1.5.mcl.table.fasta.nexus,38,,A2ntax38,Folder4ntax38/Homogroups_A_2_ntax38.nexus,,,,,,,,,,,,,,,,,,,,,,,,,,,,,,,,,,,,,,,,,,,,,,,,,,,,,,,,,,,,,,,,,,,,,,,,,,,,,,,,,,,,,,,,,,,,,, |
| Homogroups,Hol-homo-disAb41,1.00E-03,1.5,30816,cluster.All_ntaxA2ntax41_homogroups_Diamond_1e-3_all_vs_all.txt.1.5.mcl.table.fasta.nexus,41,,A2ntax41,Homogroups_A_2_ntax41_run_1.trees.tree,80001,Homogroups_A_2_ntax41_run_2.trees.tree,80001,Homogroups_A_2_ntax41_run_3.trees.tree,80001,Homogroups_A_2_ntax41_run_4.trees.tree,80001,0,0.000130988,680,680,0.0157064,1841,0.0329858,1820,0.0329712,1973,0.0331053,212 |
| Homogroups,ChoXen-homo-disAb33,1.00E-03,1.5,24302,cluster.All_A3ntax33_homogroups_Diamond_1e-3_all_vs_all.txt.1.5.mcl.table.fasta.nexus,33,,A3ntax33,,,homogroups_A3ntax33_run_1.trees.tree,:,80001,trees,homogroups_A3ntax33_run_2.trees.tree,:,80001,trees,homogroups_A3ntax33_run_3.trees.tree,:,80001,trees,homogroups_A3ntax33_run_4.trees.tree,:,80001,trees,,,0.02,0.000384496,648,648,0.0165687,5497,0.0173967,5369,0.017405 |
| Homogroups,ChoAco-homo-disAb36,1.00E-03,1.5,29017,cluster.All_A3ntax36_homogroups_Diamond_1e-3_all_vs_all.txt.1.5.mcl.table.fasta.nexus,36,,A3ntax36,Homogroups_A3ntax36_run_1.trees.tree,80001,Homogroups_A3ntax36_run_2.trees.tree,80001,Homogroups_A3ntax36_run_3.trees.tree,80001,Homogroups_A3ntax36_run_4.trees.tree,80001,0,0.000175799,815,815,0.0113396,4205,0.0230605,4129,0.0229894,4670,0.0239819,5231,0.02461 |
| Homogroups,Cho-homo-disAb39,1.00E-03,1.5,30505,cluster.All_A3ntax39_homogroups_Diamond_1e-3_all_vs_all.txt.1.5.mcl.table.fasta.nexus,39,,A2ntax35,,/home/kjurave/revBayes_run/Folder2SecondStepEnded/A3ntax39/Done_Homogroups_A_3_ntax39_nexus,,,,,,,,,,,,,,,,,,,,,,,,,,,,,,,,,,,,,,,,,,,,,,,,,,,,,,,,,,,,,,,,,,,,,,,,,,,,,,,,,,,,,,,,,,, |
| Homogroups,OpiXen-homo-disP41,1.00E-03,1.5,30477,Homogroups_B4ntax41.nexus,41,,B4ntax41,Homogroups_B4ntax41_run_1.trees.tree,80001,Homogroups_B4ntax41_run_2.trees.tree,80001,Homogroups_B4ntax41_run_3.trees.tree,80001,Homogroups_B4ntax41_run_4.trees.tree,80001,0.06,0.000658933,754,754,0.0140228,1457,0.0228014,1431,0.022794,1541,0.0205681,1673,0.0188221,1545,0.0205909,80001,0,1903,0.0149379,1903,0.0149 |
| Homogroups,OpiAco-homo-disP44,1.00E-03,1.5,32774,A_1_ntax44.nexus,44,1_3_4,B4ntax44,Homogroups_B4ntax44_run_1.trees.tree,80001,Homogroups_B4ntax44_run_3.trees.tree,80001,Homogroups_B4ntax44_run_4.trees.tree,80001,,,0.384745,0.00309572,920,920,0.00072669,1049,0.00233579,1038,0.00234933,1101,0.00250395,1169,0.002594,1103,0.00250177,80001,0,1506,0.00121556,1506,0.00121441,80001,0,"""(ACDI:0.001637",NEVE: |
| Homogroups,HolXen-homo-disP35,1.00E-03,1.5,29994,table.C5ntax35.nexus,35,,B5ntax35,Homogroups_C5ntax35_run_1.trees.tree,80001,Homogroups_C5ntax35_run_2.trees.tree,80001,Homogroups_C5ntax35_run_3.trees.tree,80001,Homogroups_C5ntax35_run_4.trees.tree,80001,0.02,0.000311172,799,799,0.0111055,5063,0.0122139,5000,0.0121646,5589,0.0148785,6209,0.0166264,5594,0.0148597,80001,0,1596,0.000861401,1596,0.00086235 |
| Homogroups,HolXen-homo-disP38,1.00E-03,1.5,30992,Homogroups_B5ntax38.nexus,38,,B5ntax38,Homogroups_B5ntax38_run_1.trees.tree,80001,Homogroups_B5ntax38_run_2.trees.tree,80001,Homogroups_B5ntax38_run_3.trees.tree,80001,Homogroups_B5ntax38_run_4.trees.tree,80001,0.01,0.000164304,787,787,0.014825,3051,0.0254043,3023,0.0253922,3229,0.0254004,3431,0.0253155,3232,0.0254029,80001,0,916,0.0141101,916,0.014108, |
| Homogroups,HolAco-homo-disP41,1.00E-03,1.5,32203,Homogroups_B5ntax41.nexus,41,,B5ntax41,Homogroups_C5ntax41_run_1.trees.tree,80001,Homogroups_C5ntax41_run_2.trees.tree,80001,Homogroups_C5ntax41_run_3.trees.tree,80001,Homogroups_C5ntax41_run_4.trees.tree,80001,0.01,0.000220363,659,659,0.00252376,1434,0.0146799,1422,0.0146273,1491,0.0150327,1578,0.0152595,1492,0.0150405,80001,0,863,0.00951211,863,0.009 |
| Homogroups,Cho-homo-disP36,1.00E-03,1.5,30963,Homogroups_B6ntax36.nexus,36,,B6ntax36,Homogroups_B6ntax36_run_1.trees.tree,80001,Homogroups_B6ntax36_run_2.trees.tree,80001,Homogroups_B6ntax36_run_3.trees.tree,80001,Homogroups_B6ntax36_run_4.trees.tree,80001,0,0.000188164,704,704,0.00695533,3586,0.0131103,3545,0.0131229,3908,0.0133587,4283,0.0134405,3911,0.0133535,80001,0,1705,0.0119339,1705,0.0119349, |
| Homogroups,ChoAco-homo-disP33,1.00E-03,1.5,30055,Homogroups_C6ntax33.nexus,33,,C6ntax33,Homogroups_C6ntax33_run_1.trees.tree,80001,Homogroups_C6ntax33_run_2.trees.tree,80001,Homogroups_C6ntax33_run_3.trees.tree,80001,Homogroups_C6ntax33_run_4.trees.tree,80001,0.01,0.000161773,611,611,0.00828753,3576,0.00714959,3520,0.00715012,3875,0.00687355,4264,0.00673813,3883,0.00687038,80001,0,1264,0.0132144,12 |
| Homogroups,Cho-homo-disAb39,1.00E-03,1.5,32171,Homogroups_C6ntax39.nexus,39,,C6ntax39,Homogroups_C6ntax39_run_1.trees.tree,80001,Homogroups_C6ntax39_run_2.trees.tree,80001,Homogroups_C6ntax39_run_3.trees.tree,80001,Homogroups_C6ntax39_run_4.trees.tree,80001,0.01,0.000414463,665,665,0.0117082,1318,0.00428723,1302,0.00427206,1339,0.00705135,1408,0.00907734,1340,0.00702494,80001,0,1198,0.00706829,1197, |
| Homogroups,OpiXen-homo-neP38,1.00E-03,1.5,24904,cluster.All_ntaxC7ntax38_homogroups_Diamond_1e-3_all_vs_all.txt.1.5.mcl.table.fasta.nexus,38,,C7ntax38,Homogroups_C7ntax38_run_1.trees.tree,80001,Homogroups_C7ntax38_run_2.trees.tree,80001,Homogroups_C7ntax38_run_3.trees.tree,80001,Homogroups_C7ntax38_run_4.trees.tree,80001,0.01,0.000153306,666,666,0.012197,1272,0.00838956,1250,0.00857703,1364,0.00813666,1481 |
| Homogroups,OpiAco-homo-neP41,1.00E-03,1.5,29636,cluster.All_ntaxC7ntax41_homogroups_Diamond_1e-3_all_vs_all.txt.1.5.mcl.table.fasta.nexus,41,,C7ntax41,Homogroups_C_7_ntax41_run_1.trees.tree,80001,Homogroups_C_7_ntax41_run_2.trees.tree,80001,Homogroups_C_7_ntax41_run_3.trees.tree,80001,Homogroups_C_7_ntax41_run_4.trees.tree,80001,0.01,0.000111975,880,880,0.0106787,1067,0.0176915,1055,0.0176531,1140,0.017287 |
| Homogroups,Opi-homo-neAb44,1.00E-03,1.5,31119,cluster.All_ntaxC7ntax44_homogroups_Diamond_1e-3_all_vs_all.txt.1.5.mcl.table.fasta.nexus,44,2_4,C7ntax44,Homogroups_C_7_ntax44_run_2.trees.tree,80001,Homogroups_C_7_ntax44_run_4.trees.tree,80001,,,,,0.0165373,0.00025242,639,754,0.00462676,645,0.0201457,639,0.0202064,671,0.0189738,705,0.0179653,672,0.0189777,80001,0,726,0.00710146,726,0.00710398,80001,0,"""(ACDI:0. |
| Homogroups,HolXen-homo-disAb32,1.00E-03,1.5,22970,cluster.All_C8ntax32_homogroups_Diamond_1e-3_all_vs_all.txt.1.5.mcl.table.fasta.nexus,32,,C8ntax32,Homogroups_C_8_ntax32_run_1.trees.tree,80001,Homogroups_C_8_ntax32_run_2.trees.tree,80001,Homogroups_C_8_ntax32_run_3.trees.tree,80001,Homogroups_C_8_ntax32_run_4.trees.tree,80001,0.02,0.000216793,841,841,0.0155523,5743,0.0118177,5655,0.0121372,6514,0.0146872, |
| Homogroups,HolAco-homo-disAb35,1.00E-03,1.5,27684,cluster.All_C8ntax35_homogroups_Diamond_1e-3_all_vs_all.txt.1.5.mcl.table.fasta.nexus,35,,C8ntax35,Homogroups_C_8_ntax35_run_1.trees.tree,80001,Homogroups_C_8_ntax35_run_2.trees.tree,80001,Homogroups_C_8_ntax35_run_3.trees.tree,80001,Homogroups_C_8_ntax35_run_4.trees.tree,80001,0,9.21E-05,739,739,0.00562785,3669,0.0184337,3630,0.0184631,4018,0.0190654,4398,0 |
| Homogroups,Hol-homo-disAb38,1.00E-03,1.5,29134,cluster.All_C8ntax38_homogroups_Diamond_1e-3_all_vs_all.txt.1.5.mcl.table.fasta.nexus,38,,C8ntax38,Homogroupc_C8ntax38,,257,257,,0.0092723,0.0214417,0.000365879,,,,,,,,,,,,,,,,,,,,,,,"""(Acropora_digitifera:0.002679",Nematostella_vectensis:0.002753,(Hydra_magnipapillata:0.007809,(((Pleurobrachia_bachei:0.009073,Mnemiopsis_leidyi:0.00215)0.99:0.013124,((Trichoplax_adhaerens:0.002705 |
| Homogroups,ChoXen-homo-neAb30,1.00E-03,1.5,22662,cluster.All_C9ntax30_homogroups_Diamond_1e-3_all_vs_all.txt.1.5.mcl.table.fasta.nexus,30,,C9ntax30,Homogroups_C_9_ntax30_run_1.trees.tree,80001,Homogroups_C_9_ntax30_run_2.trees.tree,80001,Homogroups_C_9_ntax30_run_3.trees.tree,80001,Homogroups_C_9_ntax30_run_4.trees.tree,80001,0.01,9.30E-05,517,517,0.0158219,2041,0.00624827,2012,0.00638475,2208,0.00970058, |
| Homogroups,ChoAco-homo-neAb33,1.00E-03,1.5,27366,cluster.All_C9ntax33_homogroups_Diamond_1e-3_all_vs_all.txt.1.5.mcl.table.fasta.nexus,33,,C9ntax33,Homogroups_C_9_ntax33_run_1.trees.tree,80001,Homogroups_C_9_ntax33_run_2.trees.tree,80001,Homogroups_C_9_ntax33_run_3.trees.tree,80001,Homogroups_C_9_ntax33_run_4.trees.tree,80001,0.01,0.000215895,737,777,0.0107129,4267,0.00957921,4216,0.00957092,4594,0.009349 |
| Homogroups,Cho-homo-neAb36,1.00E-03,1.5,28828,cluster.All_C9ntax36_homogroups_Diamond_1e-3_all_vs_all.txt.1.5.mcl.table.fasta.nexus,36,,C9ntax36,Homogroup_C9ntax36_run_1.trees.tree,80001,Homogroup_C9ntax36_run_2.trees.tree,80001,Homogroup_C9ntax36_run_3.trees.tree,80001,Homogroup_C9ntax36_run_4.trees.tree,80001,0.0140998,0.000186844,630,630,0.0119971,1336,0.00909337,1319,0.00910524,1388,0.0104938,1479,0.01 |
| Homogroups,OpiAco-homo-neP38,1.00E-03,1.5,30280,Homogroups_D10ntax38.nexus,38,,D10ntax38,Homogroups_D10ntax38_run_1.trees.tree,80001,Homogroups_D10ntax38_run_2.trees.tree,80001,Homogroups_D10ntax38_run_3.trees.tree,80001,Homogroups_D10ntax38_run_4.trees.tree,80001,0.06,0.000412132,763,763,0.00507311,1120,0.00564531,1101,0.00575815,1188,0.00550146,1279,0.0052365,1192,0.00548099,80001,0,1122,0.009805 |
| Homogroups,OpiAco-homo-neP41,1.00E-03,1.5,31317,Homogroups_D10ntax41.nexus,41,1_2_3,D10ntax41,Homogroups_D10ntax41_run_1.trees.tree,80001,Homogroups_D10ntax41_run_2.trees.tree,80001,Homogroups_D10ntax41_run_3.trees.tree,80001,,,0.164785,0.00160532,702,798,0.0145775,708,0.0225605,702,0.0225261,721,0.0213203,750,0.0202771,722,0.0213441,80001,0,1305,0.00786048,1305,0.00785985,80001,0,"""(ACDI:0.001723", |
| Homogroups,Opi-homo-neP44,1.00E-03,1.5,32544,Homogroups_D10ntax44.nexus,44,,D10ntax44,Homogroups_D10ntax44_run_1.trees.tree,80001,Homogroups_D10ntax44_run_2.trees.tree,80001,Homogroups_D10ntax44_run_3.trees.tree,80001,Homogroups_D10ntax44_run_4.trees.tree,80001,0.0293496,0.000225794,570,681,0.0124635,574,0.0202505,570,0.0202519,591,0.0198767,621,0.0194457,592,0.019878,80001,0,1322,0.00631447,1322,0. |
| Homogroups,HolXen-homo-disP32,1.00E-03,1.5,29822,Homogroups_D11ntax32.nexus,32,,D11ntax32,Homogroups_D11ntax32_run_1.trees.tree,80001,Homogroups_D11ntax32_run_2.trees.tree,80001,Homogroups_D11ntax32_run_3.trees.tree,80001,Homogroups_D11ntax32_run_4.trees.tree,80001,0.02,0.000164593,617,617,0.0144462,4677,0.0115852,4595,0.0117251,5164,0.00981374,5687,0.00947658,5174,0.00981604,80001,0,801,0.0152357,80 |
| Homogroups,HolAco-homo-disP35,1.00E-03,1.5,30774,Homogroups_D11ntax35.nexus,35,,D11ntax35,Homogroups_D11ntax35_run_1.trees.tree,80001,Homogroups_D11ntax35_run_2.trees.tree,80001,Homogroups_D11ntax35_run_3.trees.tree,80001,Homogroups_D11ntax35_run_4.trees.tree,80001,0,0.000119108,721,721,0.00514701,2294,0.0146712,2274,0.0147181,2496,0.0152229,2726,0.0159048,2497,0.0151995,80001,0,1189,0.00801816,1189, |
| Homogroups,Hol-homo-disP38,1.00E-03,1.5,31974,Homogroups_D11ntax38.nexus,38,,D11ntax38,Homogroups_D11ntax38.nexus,,,,,,,,,,,,,,,,,,,,,,,,,,,,,,,,,,,,,,,,,,,,,,,,,,,,,,,,,,,,,,,,,,,,,,,,,,,,,,,,,,,,,,,,,,,,,,,,,,,,,,,, |
| Homogroups,ChoXen-homo-neP30,1.00E-03,1.5,29905,Homogroups_D12ntax30.nexus,30,,D12ntax30,Homogroups_D12ntax30_run_1.trees.tree,80001,Homogroups_D12ntax30_run_2.trees.tree,80001,Homogroups_D12ntax30_run_3.trees.tree,80001,Homogroups_D12ntax30_run_4.trees.tree,80001,0.02,0.000277237,672,672,0.013725,2067,0.0101239,2031,0.0100248,2228,0.0124731,2442,0.0139418,2234,0.0124654,80001,0,1303,0.0124988,130 |
| Homogroups,ChoAco-homo-neP33,1.00E-03,1.5,30766,Homogroups_D12ntax33.nexus,33,,D12ntax33,Homogroups_D12ntax33_run_1.trees.tree,80001,Homogroups_D12ntax33_run_2.trees.tree,80001,Homogroups_D12ntax33_run_3.trees.tree,80001,Homogroups_D12ntax33_run_4.trees.tree,80001,0.01,0.000139309,1000,1000,0.0101424,4358,0.000550526,4297,0.000541839,4755,0.00156437,5205,0.00238692,4760,0.00153941,80001,0,1362,0.0 |
| Homogroups,Cho-homo-neP36,1.00E-03,1.5,31964,Homogroups_D12ntax36.nexus,36,,D12ntax36,Homogroups_D12ntax36_run_1.trees.tree,80001,Homogroups_D12ntax36_run_2.trees.tree,80001,Homogroups_D12ntax36_run_3.trees.tree,80001,Homogroups_D12ntax36_run_4.trees.tree,80001,0.0145123,0.000169456,663,663,0.0129853,1935,0.0226373,1913,0.0225666,2061,0.0243841,2208,0.0255679,2064,0.0243782,80001,0,1019,0.014476,10 |
| Homogroups,41,1.00E-02,1.5,26978,cluster.41_Species_homogroups_Diamond.1e-2.all_vs_all.txt.1.5.mcl.table.fasta.nexus,41,,,cluster.41_Species_homogroups_Diamond.1e-2.all_vs_all.txt.1.5.mcl.table.fasta_run_1.trees.tree,:,80001,trees,cluster.41_Species_homogroups_Diamond.1e-2.all_vs_all.txt.1.5.mcl.table.fasta_run_2.trees.tree,:,80001,trees,cluster.41_Species_homogroups_Diamond.1e-2.all_vs_all.txt.1.5.mcl.table.fasta_run_3.trees.tree,:,8000 |
| Homogroups,44,1.00E-02,1.5,31891,cluster.44_Species_homogroups_Diamond.1e-2.all_vs_all.txt.1.5.mcl.table.fasta.nexus,44,,,cluster.44_Species_homogroups_Diamond.1e-2.all_vs_all.txt.1.5.mcl.table.fasta_run_1.trees.tree,:,80001,trees,cluster.44_Species_homogroups_Diamond.1e-2.all_vs_all.txt.1.5.mcl.table.fasta_run_2.trees.tree,:,80001,trees,cluster.44_Species_homogroups_Diamond.1e-2.all_vs_all.txt.1.5.mcl.table.fasta_run_3.trees.tree,:,8000 |
| Homogroups,47,1.00E-02,1.5,33506,cluster.47_Species_homogroups_Diamond.1e-2.all_vs_all.txt.1.5.mcl.table.fasta.nexus,47,,,cluster.47_Species_homogroups_Diamond.1e-2.all_vs_all.txt.1.5.mcl.table.fasta_run_1.trees.tree,:,80001,trees,cluster.47_Species_homogroups_Diamond.1e-2.all_vs_all.txt.1.5.mcl.table.fasta_run_2.trees.tree,:,80001,trees,cluster.47_Species_homogroups_Diamond.1e-2.all_vs_all.txt.1.5.mcl.table.fasta_run_3.trees.tree,:,8000 |
| Homogroups,41,1.00E-12,2,29332,cluster.41_Species_homogroups_Diamond.1e-12.all_vs_all.txt.2.mcl.table.fasta.nexus,41,,,cluster.41_Species_homogroups_Diamond.1e-12.all_vs_all.txt.2.mcl.table.fasta_run_1.trees.tree,:,80001,trees,cluster.41_Species_homogroups_Diamond.1e-12.all_vs_all.txt.2.mcl.table.fasta_run_2.trees.tree,:,80001,trees,cluster.41_Species_homogroups_Diamond.1e-12.all_vs_all.txt.2.mcl.table.fasta_run_3.trees.tree,:,80001,tre |
| Homogroups,44,1.00E-12,2,33188,cluster.44_Species_homogroups_Diamond.1e-12.all_vs_all.txt.2.mcl.table.fasta.nexus,44,,,cluster.44_Species_homogroups_Diamond.1e-12.all_vs_all.txt.2.mcl.table.fasta_run_1.trees.tree,:,80001,trees,cluster.44_Species_homogroups_Diamond.1e-12.all_vs_all.txt.2.mcl.table.fasta_run_2.trees.tree,:,80001,trees,cluster.44_Species_homogroups_Diamond.1e-12.all_vs_all.txt.2.mcl.table.fasta_run_3.trees.tree,:,80001,tre |
| Homogroups,47,1.00E-12,2,34487,cluster.47_Species_homogroups_Diamond.1e-12.all_vs_all.txt.2.mcl.table.fasta.nexus,47,1_3_4,,cluster.47_Species_homogroups_Diamond.1e-12.all_vs_all.txt.2.mcl.table.fasta_run_1.trees.tree,:,80001,trees,cluster.47_Species_homogroups_Diamond.1e-12.all_vs_all.txt.2.mcl.table.fasta_run_3.trees.tree,:,80001,trees,cluster.47_Species_homogroups_Diamond.1e-12.all_vs_all.txt.2.mcl.table.fasta_run_4.trees.tree,:,80 |
| Homogroups,41,1.00E-09,2,30103,cluster.41_Species_homogroups_Diamond.1e-9.all_vs_all.txt.2.mcl.table.fasta.nexus,41,,,cluster.41_Species_homogroups_Diamond.1e-9.all_vs_all.txt.2.mcl.table.fasta_run_1.trees.tree,:,80001,trees,cluster.41_Species_homogroups_Diamond.1e-9.all_vs_all.txt.2.mcl.table.fasta_run_2.trees.tree,:,80001,trees,cluster.41_Species_homogroups_Diamond.1e-9.all_vs_all.txt.2.mcl.table.fasta_run_3.trees.tree,:,80001,trees,c |
| Homogroups,44,1.00E-09,2,34517,cluster.44_Species_homogroups_Diamond.1e-9.all_vs_all.txt.2.mcl.table.fasta.nexus,44,,,cluster.44_Species_homogroups_Diamond.1e-9.all_vs_all.txt.2.mcl.table.fasta_run_1.trees.tree,:,80001,trees,cluster.44_Species_homogroups_Diamond.1e-9.all_vs_all.txt.2.mcl.table.fasta_run_2.trees.tree,:,80001,trees,cluster.44_Species_homogroups_Diamond.1e-9.all_vs_all.txt.2.mcl.table.fasta_run_3.trees.tree,:,80001,trees,c |
| Homogroups,47,1.00E-09,2,36011,cluster.47_Species_homogroups_Diamond.1e-9.all_vs_all.txt.2.mcl.table.fasta.nexus,47,,,cluster.47_Species_homogroups_Diamond.1e-9.all_vs_all.txt.2.mcl.table.fasta_run_1.trees.tree,:,80001,trees,cluster.47_Species_homogroups_Diamond.1e-9.all_vs_all.txt.2.mcl.table.fasta_run_2.trees.tree,:,80001,trees,cluster.47_Species_homogroups_Diamond.1e-9.all_vs_all.txt.2.mcl.table.fasta_run_3.trees.tree,:,80001,trees,cl |
| Homogroups,41,1.00E-05,2,31449,cluster.41_Species_homogroups_Diamond.1e-5.all_vs_all.txt.2.mcl.table.fasta.nexus,41,2_3_4,,cluster.41_Species_homogroups_Diamond.1e-5.all_vs_all.txt.2.mcl.table.fasta_run_2.trees.tree,:,80001,trees,cluster.41_Species_homogroups_Diamond.1e-5.all_vs_all.txt.2.mcl.table.fasta_run_3.trees.tree,:,80001,trees,cluster.41_Species_homogroups_Diamond.1e-5.all_vs_all.txt.2.mcl.table.fasta_run_4.trees.tree,:,80001,t |
| Homogroups,44,1.00E-05,2,36817,cluster.44_Species_homogroups_Diamond.1e-5.all_vs_all.txt.2.mcl.table.fasta.nexus,44,,,cluster.44_Species_homogroups_Diamond.1e-5.all_vs_all.txt.2.mcl.table.fasta_run_1.trees.tree,:,80001,trees,cluster.44_Species_homogroups_Diamond.1e-5.all_vs_all.txt.2.mcl.table.fasta_run_2.trees.tree,:,80001,trees,cluster.44_Species_homogroups_Diamond.1e-5.all_vs_all.txt.2.mcl.table.fasta_run_3.trees.tree,:,80001,trees,c |
| Homogroups,47,1.00E-05,2,38721,cluster.47_Species_homogroups_Diamond.1e-5.all_vs_all.txt.2.mcl.table.fasta.nexus,47,,,cluster.47_Species_homogroups_Diamond.1e-5.all_vs_all.txt.2.mcl.table.fasta_run_1.trees.tree,:,80001,trees,cluster.47_Species_homogroups_Diamond.1e-5.all_vs_all.txt.2.mcl.table.fasta_run_2.trees.tree,:,80001,trees,cluster.47_Species_homogroups_Diamond.1e-5.all_vs_all.txt.2.mcl.table.fasta_run_3.trees.tree,:,80001,trees,c |
| Homogroups,41,1.00E-02,2,32804,cluster.41_Species_homogroups_Diamond.1e-2.all_vs_all.txt.2.mcl.table.fasta.nexus,41,,,cluster.41_Species_homogroups_Diamond.1e-2.all_vs_all.txt.2.mcl.table.fasta_run_1.trees.tree,:,80001,trees,cluster.41_Species_homogroups_Diamond.1e-2.all_vs_all.txt.2.mcl.table.fasta_run_2.trees.tree,:,80001,trees,cluster.41_Species_homogroups_Diamond.1e-2.all_vs_all.txt.2.mcl.table.fasta_run_3.trees.tree,:,80001,trees,c |
| Homogroups,44,1.00E-02,2,38977,cluster.44_Species_homogroups_Diamond.1e-2.all_vs_all.txt.2.mcl.table.fasta.nexus,44,,,cluster.44_Species_homogroups_Diamond.1e-2.all_vs_all.txt.2.mcl.table.fasta_run_1.trees.tree,:,80001,trees,cluster.44_Species_homogroups_Diamond.1e-2.all_vs_all.txt.2.mcl.table.fasta_run_2.trees.tree,:,80001,trees,cluster.44_Species_homogroups_Diamond.1e-2.all_vs_all.txt.2.mcl.table.fasta_run_3.trees.tree,:,80001,trees,c |
| Homogroups,47,1.00E-02,2,41248,cluster.47_Species_homogroups_Diamond.1e-2.all_vs_all.txt.2.mcl.table.fasta.nexus,47,,,cluster.47_Species_homogroups_Diamond.1e-2.all_vs_all.txt.2.mcl.table.fasta_run_1.trees.tree,:,80001,trees,cluster.47_Species_homogroups_Diamond.1e-2.all_vs_all.txt.2.mcl.table.fasta_run_2.trees.tree,:,80001,trees,cluster.47_Species_homogroups_Diamond.1e-2.all_vs_all.txt.2.mcl.table.fasta_run_3.trees.tree,:,80001,trees,c |
| Homogroups,41,1.00E-12,2.5,32109,cluster.41_Species_homogroups_Diamond.1e-12.all_vs_all.txt.2.5.mcl.table.fasta.nexus,41,,,cluster.41_Species_homogroups_Diamond.1e-12.all_vs_all.txt.2.5.mcl.table.fasta_run_1.trees.tree,:,80001,trees,cluster.41_Species_homogroups_Diamond.1e-12.all_vs_all.txt.2.5.mcl.table.fasta_run_2.trees.tree,:,80001,trees,cluster.41_Species_homogroups_Diamond.1e-12.all_vs_all.txt.2.5.mcl.table.fasta_run_3.trees.tree,: |
| Homogroups,44,1.00E-12,2.5,36369,cluster.44_Species_homogroups_Diamond.1e-12.all_vs_all.txt.2.5.mcl.table.fasta.nexus,44,,,cluster.44_Species_homogroups_Diamond.1e-12.all_vs_all.txt.2.5.mcl.table.fasta_run_1.trees.tree,:,80001,trees,cluster.44_Species_homogroups_Diamond.1e-12.all_vs_all.txt.2.5.mcl.table.fasta_run_2.trees.tree,:,80001,trees,cluster.44_Species_homogroups_Diamond.1e-12.all_vs_all.txt.2.5.mcl.table.fasta_run_3.trees.tree,: |
| Homogroups,47,1.00E-12,2.5,37860,cluster.47_Species_homogroups_Diamond.1e-12.all_vs_all.txt.2.5.mcl.table.fasta.nexus,47,,,cluster.47_Species_homogroups_Diamond.1e-12.all_vs_all.txt.2.5.mcl.table.fasta_run_1.trees.tree,:,80001,trees,cluster.47_Species_homogroups_Diamond.1e-12.all_vs_all.txt.2.5.mcl.table.fasta_run_2.trees.tree,:,80001,trees,cluster.47_Species_homogroups_Diamond.1e-12.all_vs_all.txt.2.5.mcl.table.fasta_run_3.trees.tree,: |
| Homogroups,41,1.00E-09,2.5,32986,cluster.41_Species_homogroups_Diamond.1e-9.all_vs_all.txt.2.5.mcl.table.fasta.nexus,41,,,cluster.41_Species_homogroups_Diamond.1e-9.all_vs_all.txt.2.5.mcl.table.fasta_run_1.trees.tree,:,80001,trees,cluster.41_Species_homogroups_Diamond.1e-9.all_vs_all.txt.2.5.mcl.table.fasta_run_2.trees.tree,:,80001,trees,cluster.41_Species_homogroups_Diamond.1e-9.all_vs_all.txt.2.5.mcl.table.fasta_run_3.trees.tree,:,8000 |
| Homogroups,44,1.00E-09,2.5,37802,cluster.44_Species_homogroups_Diamond.1e-9.all_vs_all.txt.2.5.mcl.table.fasta.nexus,44,,,cluster.44_Species_homogroups_Diamond.1e-9.all_vs_all.txt.2.5.mcl.table.fasta_run_1.trees.tree,:,80001,trees,cluster.44_Species_homogroups_Diamond.1e-9.all_vs_all.txt.2.5.mcl.table.fasta_run_2.trees.tree,:,80001,trees,cluster.44_Species_homogroups_Diamond.1e-9.all_vs_all.txt.2.5.mcl.table.fasta_run_3.trees.tree,:,8000 |
| Homogroups,47,1.00E-09,2.5,39589,cluster.47_Species_homogroups_Diamond.1e-9.all_vs_all.txt.2.5.mcl.table.fasta.nexus,47,1_2_4,,cluster.47_Species_homogroups_Diamond.1e-9.all_vs_all.txt.2.5.mcl.table.fasta_run_1.trees.tree,:,80001,trees,cluster.47_Species_homogroups_Diamond.1e-9.all_vs_all.txt.2.5.mcl.table.fasta_run_2.trees.tree,:,80001,trees,cluster.47_Species_homogroups_Diamond.1e-9.all_vs_all.txt.2.5.mcl.table.fasta_run_4.trees.tree |
| Homogroups,41,1.00E-05,2.5,34553,cluster.41_Species_homogroups_Diamond.1e-5.all_vs_all.txt.2.5.mcl.table.fasta.nexus,41,,,cluster.41_Species_homogroups_Diamond.1e-5.all_vs_all.txt.2.5.mcl.table.fasta_run_1.trees.tree,:,80001,trees,cluster.41_Species_homogroups_Diamond.1e-5.all_vs_all.txt.2.5.mcl.table.fasta_run_2.trees.tree,:,80001,trees,cluster.41_Species_homogroups_Diamond.1e-5.all_vs_all.txt.2.5.mcl.table.fasta_run_3.trees.tree,:,8000 |
| Homogroups,44,1.00E-05,2.5,40465,cluster.44_Species_homogroups_Diamond.1e-5.all_vs_all.txt.2.5.mcl.table.fasta.nexus,44,,,cluster.44_Species_homogroups_Diamond.1e-5.all_vs_all.txt.2.5.mcl.table.fasta_run_1.trees.tree,:,80001,trees,cluster.44_Species_homogroups_Diamond.1e-5.all_vs_all.txt.2.5.mcl.table.fasta_run_2.trees.tree,:,80001,trees,cluster.44_Species_homogroups_Diamond.1e-5.all_vs_all.txt.2.5.mcl.table.fasta_run_3.trees.tree,:,8000 |
| Homogroups,47,1.00E-05,2.5,42746,cluster.47_Species_homogroups_Diamond.1e-5.all_vs_all.txt.2.5.mcl.table.fasta.nexus,47,1_4,,cluster.47_Species_homogroups_Diamond.1e-5.all_vs_all.txt.2.5.mcl.table.fasta_run_1.trees.tree,:,80001,trees,cluster.47_Species_homogroups_Diamond.1e-5.all_vs_all.txt.2.5.mcl.table.fasta_run_4.trees.tree,:,80001,trees,,,0.0689491,0.000929086,892,892,0.0033995,1471,0.000150888,1462,0.000142993,1606,0.00017 |
| Homogroups,41,1.00E-02,2.5,36015,Diamond_41sp_Xen_1e-2_2.5.nexus,41,,,cluster.41_Species_homogroups_Diamond.1e-2.all_vs_all.txt.2.5.mcl.table.fasta_run_1.trees.tree,:,80001,trees,cluster.41_Species_homogroups_Diamond.1e-2.all_vs_all.txt.2.5.mcl.table.fasta_run_2.trees.tree,:,80001,trees,cluster.41_Species_homogroups_Diamond.1e-2.all_vs_all.txt.2.5.mcl.table.fasta_run_3.trees.tree,:,80001,trees,cluster.41_Species_homogroups_Diamon |
| Homogroups,44,1.00E-02,2.5,42881,cluster.44_Species_homogroups_Diamond.1e-2.all_vs_all.txt.2.5.mcl.table.fasta.nexus,44,2_4,,cluster.44_Species_homogroups_Diamond.1e-2.all_vs_all.txt.2.5.mcl.table.fasta_run_2.trees.tree,:,80001,trees,cluster.44_Species_homogroups_Diamond.1e-2.all_vs_all.txt.2.5.mcl.table.fasta_run_4.trees.tree,:,80001,trees,,,0.0213122,0.000576875,734,859,0.00114393,2192,0.00902773,2184,0.00900957,2363,0.0091481 |
| Homogroups,47,1.00E-02,2.5,45532,cluster.47_Species_homogroups_Diamond.1e-2.all_vs_all.txt.2.5.mcl.table.fasta.nexus,47,,,cluster.47_Species_homogroups_Diamond.1e-2.all_vs_all.txt.2.5.mcl.table.fasta_run_1.trees.tree,:,80001,trees,cluster.47_Species_homogroups_Diamond.1e-2.all_vs_all.txt.2.5.mcl.table.fasta_run_2.trees.tree,:,80001,trees,cluster.47_Species_homogroups_Diamond.1e-2.all_vs_all.txt.2.5.mcl.table.fasta_run_3.trees.tree,:,8000 |
| Homogroups,41,1.00E-12,4,37121,cluster.41_Species_homogroups_Diamond.1e-12.all_vs_all.txt.4.mcl.table.fasta.nexus,41,,,cluster.41_Species_homogroups_Diamond.1e-12.all_vs_all.txt.4.mcl.table.fasta_run_1.trees.tree,:,80001,trees,cluster.41_Species_homogroups_Diamond.1e-12.all_vs_all.txt.4.mcl.table.fasta_run_2.trees.tree,:,80001,trees,cluster.41_Species_homogroups_Diamond.1e-12.all_vs_all.txt.4.mcl.table.fasta_run_3.trees.tree,:,80001,tre |
| Homogroups,44,1.00E-12,4,42071,cluster.44_Species_homogroups_Diamond.1e-12.all_vs_all.txt.4.mcl.table.fasta.nexus,44,1_2_3,,cluster.44_Species_homogroups_Diamond.1e-12.all_vs_all.txt.4.mcl.table.fasta_run_1.trees.tree,:,80001,trees,cluster.44_Species_homogroups_Diamond.1e-12.all_vs_all.txt.4.mcl.table.fasta_run_2.trees.tree,:,80001,trees,cluster.44_Species_homogroups_Diamond.1e-12.all_vs_all.txt.4.mcl.table.fasta_run_3.trees.tree,:,80 |
| Homogroups,47,1.00E-12,4,44138,cluster.47_Species_homogroups_Diamond.1e-12.all_vs_all.txt.4.mcl.table.fasta.nexus,47,1_2_3,,cluster.47_Species_homogroups_Diamond.1e-12.all_vs_all.txt.4.mcl.table.fasta_run_1.trees.tree,:,80001,trees,cluster.47_Species_homogroups_Diamond.1e-12.all_vs_all.txt.4.mcl.table.fasta_run_2.trees.tree,:,80001,trees,cluster.47_Species_homogroups_Diamond.1e-12.all_vs_all.txt.4.mcl.table.fasta_run_3.trees.tree,:,80 |
| Homogroups,41,1.00E-09,4,38154,cluster.41_Species_homogroups_Diamond.1e-9.all_vs_all.txt.4.mcl.table.fasta.nexus,41,,,cluster.41_Species_homogroups_Diamond.1e-9.all_vs_all.txt.4.mcl.table.fasta_run_1.trees.tree,:,80001,trees,cluster.41_Species_homogroups_Diamond.1e-9.all_vs_all.txt.4.mcl.table.fasta_run_2.trees.tree,:,80001,trees,cluster.41_Species_homogroups_Diamond.1e-9.all_vs_all.txt.4.mcl.table.fasta_run_3.trees.tree,:,80001,trees,c |
| Homogroups,44,1.00E-09,4,43763,cluster.44_Species_homogroups_Diamond.1e-9.all_vs_all.txt.4.mcl.table.fasta.nexus,44,3_4,,cluster.44_Species_homogroups_Diamond.1e-9.all_vs_all.txt.4.mcl.table.fasta_run_3.trees.tree,:,80001,trees,cluster.44_Species_homogroups_Diamond.1e-9.all_vs_all.txt.4.mcl.table.fasta_run_4.trees.tree,:,80001,trees,,,0.0131123,0.000247897,1160,1160,0.00220858,1945,0.0170461,1940,0.017176,2023,0.0175681,2132,0.0 |
| Homogroups,47,1.00E-09,4,46181,cluster.47_Species_homogroups_Diamond.1e-9.all_vs_all.txt.4.mcl.table.fasta.nexus,47,,,cluster.47_Species_homogroups_Diamond.1e-9.all_vs_all.txt.4.mcl.table.fasta_run_1.trees.tree,:,80001,trees,cluster.47_Species_homogroups_Diamond.1e-9.all_vs_all.txt.4.mcl.table.fasta_run_2.trees.tree,:,80001,trees,cluster.47_Species_homogroups_Diamond.1e-9.all_vs_all.txt.4.mcl.table.fasta_run_3.trees.tree,:,80001,trees,c |
| Homogroups,41,1.00E-05,4,40070,cluster.41_Species_homogroups_Diamond.1e-5.all_vs_all.txt.4.mcl.table.fasta.nexus,41,1_2_3,,cluster.41_Species_homogroups_Diamond.1e-5.all_vs_all.txt.4.mcl.table.fasta_run_1.trees.tree,:,80001,trees,cluster.41_Species_homogroups_Diamond.1e-5.all_vs_all.txt.4.mcl.table.fasta_run_2.trees.tree,:,80001,trees,cluster.41_Species_homogroups_Diamond.1e-5.all_vs_all.txt.4.mcl.table.fasta_run_3.trees.tree,:,80001,t |
| Homogroups,44,1.00E-05,4,46871,cluster.44_Species_homogroups_Diamond.1e-5.all_vs_all.txt.4.mcl.table.fasta.nexus,44,,,cluster.44_Species_homogroups_Diamond.1e-5.all_vs_all.txt.4.mcl.table.fasta_run_1.trees.tree,:,80001,trees,cluster.44_Species_homogroups_Diamond.1e-5.all_vs_all.txt.4.mcl.table.fasta_run_2.trees.tree,:,80001,trees,cluster.44_Species_homogroups_Diamond.1e-5.all_vs_all.txt.4.mcl.table.fasta_run_3.trees.tree,:,80001,trees,c |
| Homogroups,47,1.00E-05,4,49865,cluster.47_Species_homogroups_Diamond.1e-5.all_vs_all.txt.4.mcl.table.fasta.nexus,47,,,cluster.47_Species_homogroups_Diamond.1e-5.all_vs_all.txt.4.mcl.table.fasta_run_1.trees.tree,:,80001,trees,cluster.47_Species_homogroups_Diamond.1e-5.all_vs_all.txt.4.mcl.table.fasta_run_2.trees.tree,:,80001,trees,cluster.47_Species_homogroups_Diamond.1e-5.all_vs_all.txt.4.mcl.table.fasta_run_3.trees.tree,:,80001,trees,c |

```
Homogroups,41,1.00E-02,4,41673,cluster.41_Species_homogroups_Diamond.1e-2.all_vs_all.txt.4.mcl.table.fasta.nexus,41,,,cluster.41_Species_homogroups_Diamond.1e-2.all_vs_all.txt.4.mcl.table.fasta_run_1.trees.tree,:,80001,trees,cluster.41_Species_homogroups_Diamond.1e-2.all_vs_all.txt.4.mcl.table.fasta_run_2.trees.tree,:,80001,trees,cluster.41_Species_homogroups_Diamond.1e-2.all_vs_all.txt.4.mcl.table.fasta_run_3.trees.tree,:,80001,trees,c
Homogroups,44,1.00E-02,4,49635,cluster.44_Species_homogroups_Diamond.1e-2.all_vs_all.txt.4.mcl.table.fasta.nexus,44,,,cluster.44_Species_homogroups_Diamond.1e-2.all_vs_all.txt.4.mcl.table.fasta_run_1.trees.tree,:,80001,trees,cluster.44_Species_homogroups_Diamond.1e-2.all_vs_all.txt.4.mcl.table.fasta_run_2.trees.tree,:,80001,trees,cluster.44_Species_homogroups_Diamond.1e-2.all_vs_all.txt.4.mcl.table.fasta_run_3.trees.tree,:,80001,trees,c
Homogroups,47,1.00E-02,4,53090,cluster.47_Species_homogroups_Diamond.1e-2.all_vs_all.txt.4.mcl.table.fasta.nexus,47,,,cluster.47_Species_homogroups_Diamond.1e-2.all_vs_all.txt.4.mcl.table.fasta_run_1.trees.tree,:,80001,trees,cluster.47_Species_homogroups_Diamond.1e-2.all_vs_all.txt.4.mcl.table.fasta_run_2.trees.tree,:,80001,trees,cluster.47_Species_homogroups_Diamond.1e-2.all_vs_all.txt.4.mcl.table.fasta_run_3.trees.tree,:,80001,trees,c
Homogroups,41,1.00E-12,6,40738,cluster41_Species_homogroups_Diamond.1e-12.all_vs_all.txt.6.mcl.table.fasta.nexus,41,,,cluster41_homogroups_Diamond.1e-12.all_vs_all.txt.6.mcl.table.fasta_run_1.trees.tree,:,80001,trees,cluster41_homogroups_Diamond.1e-12.all_vs_all.txt.6.mcl.table.fasta_run_2.trees.tree,:,80001,trees,cluster41_homogroups_Diamond.1e-12.all_vs_all.txt.6.mcl.table.fasta_run_3.trees.tree,:,80001,trees,cluster41_homogroups_Di
Homogroups,44,1.00E-12,6,46123,cluster44_Species_homogroups_Diamond.1e-12.all_vs_all.txt.6.mcl.table.fasta.nexus,44,,,cluster44_homogroups_Diamond.1e-12.all_vs_all.txt.6.mcl.table.fasta_run_1.trees.tree,:,80001,trees,cluster44_homogroups_Diamond.1e-12.all_vs_all.txt.6.mcl.table.fasta_run_2.trees.tree,:,80001,trees,cluster44_homogroups_Diamond.1e-12.all_vs_all.txt.6.mcl.table.fasta_run_3.trees.tree,:,80001,trees,cluster44_homogroups_Di
Homogroups,47,1.00E-12,6,48738,cluster47_Species_homogroups_Diamond.1e-12.all_vs_all.txt.6.mcl.table.fasta.nexus,47,1_4,,cluster47_homogroups_Diamond.1e-2.all_vs_all.txt.6.mcl.table.fasta_run_1.trees.tree,:,80001,trees,cluster47_homogroups_Diamond.1e-2.all_vs_all.txt.6.mcl.table.fasta_run_4.trees.tree,:,80001,trees,,,0.0277747,0.0004464,754,754,0.00341444,12603,0.0396977,12808,0.0396798,16440,0.0397655,22146,0.0398117,16296,0
Homogroups,41,1.00E-09,6,41957,cluster41_Species_homogroups_Diamond.1e-9.all_vs_all.txt.6.mcl.table.fasta.nexus,41,,,cluster41_homogroups_Diamond.1e-9.all_vs_all.txt.6.mcl.table.fasta_run_1.trees.tree,:,80001,trees,cluster41_homogroups_Diamond.1e-9.all_vs_all.txt.6.mcl.table.fasta_run_2.trees.tree,:,80001,trees,cluster41_homogroups_Diamond.1e-9.all_vs_all.txt.6.mcl.table.fasta_run_3.trees.tree,:,80001,trees,cluster41_homogroups_Diamor
Homogroups,44,1.00E-09,6,48076,cluster44_Species_homogroups_Diamond.1e-9.all_vs_all.txt.6.mcl.table.fasta.nexus,44,,,cluster44_homogroups_Diamond.1e-9.all_vs_all.txt.6.mcl.table.fasta_run_1.trees.tree,:,80001,trees,cluster44_homogroups_Diamond.1e-9.all_vs_all.txt.6.mcl.table.fasta_run_2.trees.tree,:,80001,trees,cluster44_homogroups_Diamond.1e-9.all_vs_all.txt.6.mcl.table.fasta_run_3.trees.tree,:,80001,trees,cluster44_homogroups_Diamor
Homogroups,47,1.00E-09,6,51115,cluster47_Species_homogroups_Diamond.1e-9.all_vs_all.txt.6.mcl.table.fasta.nexus,47,,,cluster47_homogroups_Diamond.1e-9.all_vs_all.txt.6.mcl.table.fasta_run_1.trees.tree,:,80001,trees,cluster47_homogroups_Diamond.1e-9.all_vs_all.txt.6.mcl.table.fasta_run_2.trees.tree,:,80001,trees,cluster47_homogroups_Diamond.1e-9.all_vs_all.txt.6.mcl.table.fasta_run_3.trees.tree,:,80001,trees,cluster47_homogroups_Diamor
Homogroups,41,1.00E-05,6,43897,cluster41_Species_homogroups_Diamond.1e-5.all_vs_all.txt.6.mcl.table.fasta.nexus,41,,,cluster41_homogroups_Diamond.1e-5.all_vs_all.txt.6.mcl.table.fasta_run_1.trees.tree,:,80001,trees,cluster41_homogroups_Diamond.1e-5.all_vs_all.txt.6.mcl.table.fasta_run_2.trees.tree,:,80001,trees,cluster41_homogroups_Diamond.1e-5.all_vs_all.txt.6.mcl.table.fasta_run_3.trees.tree,:,80001,trees,cluster41_homogroups_Diamor
Homogroups,44,1.00E-05,6,51428,cluster44_Species_homogroups_Diamond.1e-5.all_vs_all.txt.6.mcl.table.fasta.nexus,44,,,cluster44_homogroups_Diamond.1e-5.all_vs_all.txt.6.mcl.table.fasta_run_1.trees.tree,:,80001,trees,cluster44_homogroups_Diamond.1e-5.all_vs_all.txt.6.mcl.table.fasta_run_2.trees.tree,:,80001,trees,cluster44_homogroups_Diamond.1e-5.all_vs_all.txt.6.mcl.table.fasta_run_3.trees.tree,:,80001,trees,cluster44_homogroups_Diamor
Homogroups,47,1.00E-05,6,55118,cluster47_Species_homogroups_Diamond.1e-5.all_vs_all.txt.6.mcl.table.fasta.nexus,47,,,cluster47_homogroups_Diamond.1e-5.all_vs_all.txt.6.mcl.table.fasta_run_1.trees.tree,:,80001,trees,cluster47_homogroups_Diamond.1e-5.all_vs_all.txt.6.mcl.table.fasta_run_2.trees.tree,:,80001,trees,cluster47_homogroups_Diamond.1e-5.all_vs_all.txt.6.mcl.table.fasta_run_3.trees.tree,:,80001,trees,cluster47_homogroups_Diamor
Homogroups,41,1.00E-02,6,45671,cluster41_Species_homogroups_Diamond.1e-2.all_vs_all.txt.6.mcl.table.fasta.nexus,41,,,cluster41_homogroups_Diamond.1e-2.all_vs_all.txt.6.mcl.table.fasta_run_1.trees.tree,:,80001,trees,cluster41_homogroups_Diamond.1e-2.all_vs_all.txt.6.mcl.table.fasta_run_2.trees.tree,:,80001,trees,cluster41_homogroups_Diamond.1e-2.all_vs_all.txt.6.mcl.table.fasta_run_3.trees.tree,:,80001,trees,cluster41_homogroups_Diamor
Homogroups,44,1.00E-02,6,54447,cluster44_Species_homogroups_Diamond.1e-2.all_vs_all.txt.6.mcl.table.fasta.nexus,44,,,cluster44_homogroups_Diamond.1e-2.all_vs_all.txt.6.mcl.table.fasta_run_1.trees.tree,:,80001,trees,cluster44_homogroups_Diamond.1e-2.all_vs_all.txt.6.mcl.table.fasta_run_2.trees.tree,:,80001,trees,cluster44_homogroups_Diamond.1e-2.all_vs_all.txt.6.mcl.table.fasta_run_3.trees.tree,:,80001,trees,cluster44_homogroups_Diamor
Homogroups,47,1.00E-02,6,58664,cluster47_Species_homogroups_Diamond.1e-2.all_vs_all.txt.6.mcl.table.fasta.nexus,47,1_4,,cluster47_homogroups_Diamond.1e-2.all_vs_all.txt.6.mcl.table.fasta_run_1.trees.tree,:,80001,trees,cluster47_homogroups_Diamond.1e-2.all_vs_all.txt.6.mcl.table.fasta_run_4.trees.tree,:,80001,trees,,,0.0277747,0.0004464,754,754,0.00341444,12603,0.0396977,12808,0.0396798,16440,0.0397655,22146,0.0398117,16296,0.0
Orthogroups,41,1.00E-12,1.5,26167,Orthogroups.Results_41.1e-12.I.1.5_Orthogroups.tsv.mcl.out.nexus,41,,,41.1e-12.I.1.5_Orthogroups.tsv.mcl.out_run_1.trees.tree,:,80001,trees,41.1e-12.I.1.5_Orthogroups.tsv.mcl.out_run_2.trees.tree,:,80001,trees,41.1e-12.I.1.5_Orthogroups.tsv.mcl.out_run_3.trees.tree,:,80001,trees,41.1e-12.I.1.5_Orthogroups.tsv.mcl.out_run_4.trees.tree,:,80001,trees,0.0180248,0.000348438,782,782,0.0120486,2823,0.00412309,2
Orthogroups,44,1.00E-12,1.5,32858,Orthogroups.Results_44.1e-12.I.1.5_Orthogroups.tsv.mcl.out.nexus,44,,,44.1e-12.I.1.5_Orthogroups.tsv.mcl.out_run_1.trees.tree,:,80001,trees,44.1e-12.I.1.5_Orthogroups.tsv.mcl.out_run_2.trees.tree,:,80001,trees,44.1e-12.I.1.5_Orthogroups.tsv.mcl.out_run_3.trees.tree,:,80001,trees,44.1e-12.I.1.5_Orthogroups.tsv.mcl.out_run_4.trees.tree,:,80001,trees,0.0301871,0.000329876,553,736,0.00201179,556,0.0116646,55
Orthogroups,47,1.00E-12,1.5,35584,Orthogroups.1e12.1_5.nexus,47,,,/home/kjurave/revBayes_run/data/Orthogroups.1e12.1_5,../../output/Orthogroups.1e12.1_5_run_1.trees.tree,:,80001,trees,../../output/Orthogroups.1e12.1_5_run_2.trees.tree,:,80001,trees,../../output/Orthogroups.1e12.1_5_run_3.trees.tree,:,80001,trees,../../output/Orthogroups.1e12.1_5_run_4.trees.tree,:,80001,trees,maxdiff,:,0.0594493,meandiff,:,0.000726095,bipartition,list,in,:,bpcor
Orthogroups,41,1.00E-09,1.5,26125,Orthogroups.Results_41.1e-9.I.1.5_Orthogroups.tsv.mcl.out.nexus,41,,,41.1e-9.I.1.5_Orthogroups.tsv.mcl.out_run_1.trees.tree,:,80001,trees,41.1e-9.I.1.5_Orthogroups.tsv.mcl.out_run_2.trees.tree,:,80001,trees,41.1e-9.I.1.5_Orthogroups.tsv.mcl.out_run_3.trees.tree,:,80001,trees,41.1e-9.I.1.5_Orthogroups.tsv.mcl.out_run_4.trees.tree,:,80001,trees,0.0200997,0.00032122,973,973,0.0150261,2837,0.00466841,2721,0.0
Orthogroups,44,1.00E-09,1.5,32852,Orthogroups.Results_44.1e-9.I.1.5_Orthogroups.tsv.mcl.out.nexus,44,,,44.1e-9.I.1.5_Orthogroups.tsv.mcl.out_run_1.trees.tree,:,80001,trees,44.1e-9.I.1.5_Orthogroups.tsv.mcl.out_run_2.trees.tree,:,80001,trees,44.1e-9.I.1.5_Orthogroups.tsv.mcl.out_run_3.trees.tree,:,80001,trees,44.1e-9.I.1.5_Orthogroups.tsv.mcl.out_run_4.trees.tree,:,80001,trees,0.0894489,0.0007712,519,737,0.0130458,527,0.013722,525,0.013722
Orthogroups,47,1.00E-09,1.5,35578,Orthogroups.Results_NEW_47.1e-9.I.1.5_Orthogroups.tsv.mcl.out.nexus,47,3_4,,gen.nexus,Orthogroups.Results_NEW_47.1e-9.I.1.5_Orthogroups.tsv.mcl.out_run_4.trees.tree,:,80001,trees,,,0.0110624,0.000649838,265,483,0.00101293,275,0.00289338,276,0.00302785,267,0.00144913,265,0.000245521,267,0.00144825,80001,0,334,0.00555687,334,0.00555627,80001,0,,,,,"""(ACDI:0.001176",NEVE:0.001245,(HYI
Orthogroups,41,1.00E-05,1.5,26066,Orthogroups.Results_41.1e-5.I.1.5_Orthogroups.tsv.mcl.out.nexus,41,,,41.1e-5.I.1.5_Orthogroups.tsv.mcl.out_run_1.trees.tree,:,80001,trees,41.1e-5.I.1.5_Orthogroups.tsv.mcl.out_run_2.trees.tree,:,80001,trees,41.1e-5.I.1.5_Orthogroups.tsv.mcl.out_run_3.trees.tree,:,80001,trees,41.1e-5.I.1.5_Orthogroups.tsv.mcl.out_run_4.trees.tree,:,80001,trees,0.0183998,0.000375541,780,780,0.00071676,1217,0.0119523,1203,0
Orthogroups,44,1.00E-05,1.5,32793,Orthogroups.Results_44.1e-5.I.1.5_Orthogroups.tsv.mcl.out.nexus,44,,,44.1e-5.I.1.5_Orthogroups.tsv.mcl.out_run_1.trees.tree,:,80001,trees,44.1e-5.I.1.5_Orthogroups.tsv.mcl.out_run_2.trees.tree,:,80001,trees,44.1e-5.I.1.5_Orthogroups.tsv.mcl.out_run_3.trees.tree,:,80001,trees,44.1e-5.I.1.5_Orthogroups.tsv.mcl.out_run_4.trees.tree,:,80001,trees,0.0282996,0.000554691,558,826,0.00549213,564,0.00463195,559,0.0
Orthogroups,47,1.00E-05,1.5,35502,Orthogroups.Results_NEW_47.1e-5.I.1.5_Orthogroups.tsv.mcl.out.nexus,47,1_2,,Orthogroups.Results_NEW_47.1e-5.I.1.5_Orthogroups.tsv.mcl.out_run_1.trees.tree,:,80001,trees,Orthogroups.Results_NEW_47.1e-5.I.1.5_Orthogroups.tsv.mcl.out_run_2.trees.tree,:,80001,trees,,,0.0236622,0.000498527,260,495,0.00687912,284,0.0101669,285,0.0103341,267,0.0103172,260,0.010086,267,0.0102852,80001,0,672,0.00
Orthogroups,OpiXen-ortho-disAb41,1.00E-03,1.5,26063,Orthogroups_A_1_ntax41.nexus,41,,A1ntax41,/home/kjurave/revBayes_run/Folder2SecondStepEnded/A1ntax41/Orthogroups_A_ntax41.nexus,,,,,,,,,,,,,,,,,,,,,,,,,,,,,,,,,,,,,,,,,,,,,,,,,,,,,,,,,,,,,,,,,,,,,,,,,,,,
Orthogroups,OpiAco-ortho-disAb44,1.00E-03,1.5,36596,Orthogroups.tsv.out.fasta.nexus,44,,A1ntax44,Orthogroups_47_ntax44_run_1.trees.tree,50001,Orthogroups_47_ntax44_run_2.trees.tree,50001,Orthogroups_47_ntax44_run_3.trees.tree,50001,Orthogroups_47_ntax44_run_4.trees.tree,50001,0.01,0.000188658,314,533,0.00718603,326,0.0106621,326,0.0110122,315,0.00879661,314,0.00874553,315,0.00881681,50001,0,619,0.0111018,619,0.011
Orthogroups,Cho-ortho-disAb47,1.00E-03,1.5,35455,Orthogroups_ntax47_base.nexus,47,,A1ntax47,/home/kjurave/revBayes_run/Folder2SecondStepEnded/A1ntax47/Orthogroups_ntax47_base.nexus,,,,,,,,,,,,,,,,,,,,,,,,,,,,,,,,,,,,,,,,,,,,,,,,,,,,,,,,,,,,,,,,,,,,,,,,,,,,
Orthogroups,HolXen-ortho-disAb35,1.00E-03,1.5,25104,Orthogroups.A2ntax35.nexus,35,,A2ntax35,Orthogroups.A2ntax35_run_1.trees.tree,:,80001,trees,Orthogroups.A2ntax35_run_2.trees.tree,:,80001,trees,Orthogroups.A2ntax35_run_3.trees.tree,:,80001,trees,Orthogroups.A2ntax35_run_4.trees.tree,:,80001,trees,,,,0.040612,0.000581549,782,797,0.0086546,4289,0.0265106,4173,0.0262468,4676,0.0275846,5144,0.0283382,4700,0.0276146,80001,
Orthogroups,HolAco-ortho-disAb38,1.00E-03,1.5,31885,A_srtaregy_2_ntax38.nexus,38,,A2ntax38,/home/kjurave/revBayes_run/data/ZIP/Done_Orthogroups_A_srtaregy_2_ntax38_nexus,,,,,,,,,,,,,,,,,,,,,,,,,,,,,,,,,,,,,,,,,,,,,,,,,,,,,,,,,,,,,,,,,,,,,,,,,,,,
Orthogroups,Hol-ortho-disAb41,1.00E-03,1.5,34495,Orthogroups.A2ntax41.nexus,41,,A2ntax41,Orthogroups.A2ntax41,../../output/Orthogroups.A2ntax41_run_1.trees.tree,:,80001,trees,../../output/Orthogroups.A2ntax41_run_2.trees.tree,:,80001,trees,../../output/Orthogroups.A2ntax41_run_3.trees.tree,:,80001,trees,../../output/Orthogroups.A2ntax41_run_4.trees.tree,:,80001,trees,,,,maxdiff,:,0.99655,meandiff,:,0.0203319,bipartition,list,in,:,bpcomp.bplist,co
Orthogroups,ChoAco-ortho-disAb33,1.00E-03,1.5,25050,Orthogroups_A3ntax33.nexus,33,1_3_4,A3ntax33,Orthogroups_A3ntax33_run_1.trees.tree,80001,Orthogroups_A3ntax33_run_3.trees.tree,80001,Orthogroups_A3ntax33_run_4.trees.tree,80001,,,0.07,0.00106701,826,826,0.00322892,2182,0.0135648,2134,0.0135004,2256,0.014077,2469,0.0141559,2267,0.0140821,80001,0,1967,0.0043251,1967,0.00432625,80001,0,"""(ACDI:0.002324",NEVE:(
Orthogroups,Cho-ortho-disAb36,1.00E-03,1.5,31786,A_srtaregy_3_ntax44_ntax36.nexus,36,,A3ntax36,Orthogroups_A3ntax36_run_1.trees.tree,80001,Orthogroups_A3ntax36_run_2.trees.tree,80001,Orthogroups_A3ntax36_run_3.trees.tree,80001,Orthogroups_A3ntax36_run_4.trees.tree,80001,0.03,0.000459234,682,682,0.019138,700,0.00442929,691,0.00424994,729,0.00601931,773,0.0072279,731,0.00602911,80001,0,740,0.0101357,740,0.010136
Orthogroups,Cho-ortho-disAb39,1.00E-03,1.5,34755,Orthogroups_47_ntax39.nexus,39,,A3ntax39,/home/kjurave/revBayes_run/Folder2SecondStepEnded/A3ntax39/Orthogroups_47_ntax39.nexus,,,,,,,,,,,,,,,,,,,,,,,,,,,,,,,,,,,,,,,,,,,,,,,,,,,,,,,,,,,,,,,,,,,,,,,,,,,,,
Orthogroups,OpiXen-ortho-disP41,1.00E-03,1.5,31067,table.B4ntax41.nexus,41,,B4ntax41,Orthogroups_47_ntax41_run_1.trees.tree,50001,Orthogroups_47_ntax41_run_2.trees.tree,50001,Orthogroups_47_ntax41_run_3.trees.tree,50001,Orthogroups_47_ntax41_run_4.trees.tree,50001,0.01,0.000201202,511,511,0.0115599,747,0.0118809,739,0.0119524,739,0.0120303,765,0.0118815,741,0.0120153,50001,0,580,0.0102761,580,0.0102789,50001,0,""""
Orthogroups,OpiAco-ortho-disP44,1.00E-03,1.5,32886,table.B4ntax44.nexus,44,1_3_4,B4ntax44,Outgroups_B4ntax44_run_1.trees.tree,80001,Outgroups_B4ntax44_run_3.trees.tree,80001,Outgroups_B4ntax44_run_4.trees.tree,80001,,,0.00188748,7.84E-05,535,723,0.00681974,543,0.00260274,540,0.00242183,535,0.00452828,550,0.00614179,536,0.00453926,80001,0,1389,0.00292936,1389,0.00293105,80001,0,"""(ACDI:0.001583",NEVE:0.001665
Orthogroups,HolXen-ortho-disP35,1.00E-03,1.5,30717,table.B5ntax35.nexus,35,,B5ntax35,table.B5ntax35_run_1.trees.tree,:,80001,trees,table.B5ntax35_run_2.trees.tree,:,80001,trees,table.B5ntax35_run_3.trees.tree,:,80001,trees,table.B5ntax35_run_4.trees.tree,:,80001,trees,0.0137873,0.00025244,893,893,0.0135552,3031,0.0163463,2963,0.016155,3288,0.0149208,3680,0.0140299,3303,0.014952,80001,0,1356,0.0137627,1356,0.0137625,80001,0,
Orthogroups,HolAco-ortho-disP38,1.00E-03,1.5,32469,table.B5ntax38.nexus,38,,B5ntax38,Orthogroups_B5ntax38_run_1.trees.tree,80001,Orthogroups_B5ntax38_run_2.trees.tree,80001,Orthogroups_B5ntax38_run_3.trees.tree,80001,Orthogroups_B5ntax38_run_4.trees.tree,80001,0.01,0.000280377,925,925,0.0127764,1077,0.0200543,1061,0.0200814,1127,0.0194112,1204,0.0187907,1130,0.0194135,80001,0,1081,0.0189266,1081,0.0189277,80001
Orthogroups,Hol-ortho-disP41,1.00E-03,1.5,35030,table.B5ntax41.nexus,41,,B5ntax41,/home/kjurave/revBayes_run/data/ZIP/Orthogroups_B5ntax41.nexus,,,,,,,,,,,,,,,,,,,,,,,,,,,,,,,,,,,,,,,,,,,,,,,,,,,,,,,,,,,,,,,,,,,,,,,,,,,,,,,,,,,,,,,,,,,,,
Orthogroups,ChoXen-ortho-disP33,1.00E-03,1.5,30751,table.B6ntax33.nexus,33,,B6ntax33,table.B6ntax33_run_1.trees.tree,:,80001,trees,table.B6ntax33_run_2.trees.tree,:,80001,trees,table.B6ntax33_run_3.trees.tree,:,80001,trees,table.B6ntax33_run_4.trees.tree,:,80001,trees,,,,,0.05,0.000579835,728,728,0.0101008,2904,0.0108077,2836,0.0108018,3077,0.0102728,3407,0.0119656,3093,0.0102709,80001,0,1409,0.01085,1409,0.0108518,80001,0,"""
Orthogroups,ChoAco-ortho-disP36,1.00E-03,1.5,32400,table.B6ntax36.nexus,36,,B6ntax36,Orthogroups_B6ntax36_run_1.trees.tree,80001,Orthogroups_B6ntax36_run_2.trees.tree,80001,Orthogroups_B6ntax36_run_3.trees.tree,80001,Orthogroups_B6ntax36_run_4.trees.tree,80001,0.03,0.000509356,776,776,0.00776437,1041,0.0189476,1026,0.0187696,1080,0.0196831,1143,0.0202134,1082,0.0197067,80001,0,797,0.0151405,797,0.0151414,80001
Orthogroups,Cho-ortho-disAb39,1.00E-03,1.5,34941,Orthogroups_B6ntax39.nexus,39,1_2_3,B6ntax39,Orthogroups_B6ntax39_run_1.trees.tree,80001,Orthogroups_B6ntax39_run_2.trees.tree,80001,Orthogroups_B6ntax39_run_3.trees.tree,80001,,,0.994925,0.0434136,312,447,0.639892,349,1.2191,347,1.19868,337,1.36068,338,1.47172,338,1.36266,80001,0,312,0.699744,312,0.699741,80001,0,"""(ACDI:0.002514",NEVE:0.002725,(HYMA:0.006584,
Orthogroups,OpiXen-ortho-neP38,1.00E-03,1.5,24683,OpiXen_ortho_neAb38_1e5_1.5.nexus,38,,C7ntax38,Orthogroups_C7ntax38_run_1.trees.tree,80001,Orthogroups_C7ntax38_run_2.trees.tree,80001,Orthogroups_C7ntax38_run_3.trees.tree,80001,Orthogroups_C7ntax38_run_4.trees.tree,80001,0.01,0.000141089,746,746,0.00988325,1013,0.0115898,993,0.0115984,1052,0.0113645,1138,0.013023,1057,0.0113617,80001,0,1084,0.00877324,1084,
Orthogroups,OpiAco-ortho-neP41,1.00E-03,1.5,26063,Orthogroups_C_7_ntax41.nexus,41,,C7ntax41,Orthogroups_C_7_ntax41_run_1.trees.tree,80001,Orthogroups_C_7_ntax41_run_2.trees.tree,80001,Orthogroups_C_7_ntax41_run_3.trees.tree,80001,Orthogroups_C_7_ntax41_run_4.trees.tree,80001,0.06,0.000703123,865,865,0.00549021,1414,0.0182616,1381,0.0177698,1493,0.0209001,1628,0.0227753,1501,0.020947,80001,0,1694,0.0104319,1
Orthogroups,Opi-ortho-neAb44,1.00E-03,1.5,34023,Orthogroups_C_7_ntax44.nexus,44,,C7ntax44,Orthogroups_C_7_ntax44_run_1.trees.tree,80001,Orthogroups_C_7_ntax44_run_2.trees.tree,80001,Orthogroups_C_7_ntax44_run_3.trees.tree,80001,Orthogroups_C_7_ntax44_run_4.trees.tree,80001,0.11,0.000870216,418,536,0.0143303,419,0.00314701,418,0.00307901,418,0.00310872,427,0.00315063,419,0.00312403,80001,0,541,0.00640004,541,
Orthogroups,HolXen-ortho-neAb32,1.00E-03,1.5,23716,Orthogroups_C8ntax32.nexus,32,,C8ntax32,Orthogroups_C8ntax32_run_1.trees.tree,80001,Orthogroups_C8ntax32_run_2.trees.tree,80001,Orthogroups_C8ntax32_run_3.trees.tree,80001,Orthogroups_C8ntax32_run_4.trees.tree,80001,0,6.71E-05,980,980,0.0130919,3790,0.0113917,3672,0.0113829,4203,0.0107041,4682,0.0102905,4224,0.0107181,80001,0,1105,0.0163655,1105,0.0163659,800
Orthogroups,HolAco-ortho-neAb35,1.00E-03,1.5,30435,Orthogroups_C8ntax35.nexus,35,,C8ntax35,Orthogroups_C8ntax35_run_1.trees.tree,80001,Orthogroups_C8ntax35_run_2.trees.tree,80001,Orthogroups_C8ntax35_run_3.trees.tree,80001,Orthogroups_C8ntax35_run_4.trees.tree,80001,0.01,0.000177518,623,623,0.0210661,785,0.0228059,777,0.0227295,827,0.0225171,885,0.0222323,828,0.0225276,80001,0,1347,0.0148842,1347,0.0148842,80
Orthogroups,Hol-ortho-neAb38,1.00E-03,1.5,33072,Orthogroups_C8ntax38.nexus,38,,C8ntax38,Orthogroups_C8ntax38/1_4,,435,493,,0.000907305,0.00514994,0.000375858,,,,,,,,,,,,,,,,,,,,,"""(Acropora_digitifera:0.003288",Nematostella_vectensis:0.003636,(Hydra_magnipapillata:0.009425,(((Trichoplax_adhaerens:0.004153,Hoilungia_hongkongensis:0.001662)0.99:0.007563,((Pleurobrachia_bachei:0.011127,Mnemiopsis_leidyi:0.003298)0.99:0.012461
Orthogroups,ChoXen-ortho-neAb30,1.00E-03,1.5,23703,Orthogroups_C_9_ntax30.nexus,30,,C9ntax30,Orthogroups_C_9_ntax30_run_1.trees.tree,80001,Orthogroups_C_9_ntax30_run_2.trees.tree,80001,Orthogroups_C_9_ntax30_run_3.trees.tree,80001,Orthogroups_C_9_ntax30_run_4.trees.tree,80001,0,0.000124078,586,586,0.0119409,1067,0.00349046,1045,0.0036822,1131,0.0029324,1212,0.00241584,1136,0.00290495,80001,0,699,0.00671704
Orthogroups,ChoAco-ortho-neAb33,1.00E-03,1.5,30411,Orthogroups_C_8_ntax33.nexus,33,,C9ntax33,Orthogroups_C_8_ntax33_run_1.trees.tree,80001,Orthogroups_C_8_ntax33_run_2.trees.tree,80001,Orthogroups_C_8_ntax33_run_3.trees.tree,80001,Orthogroups_C_8_ntax33_run_4.trees.tree,80001,0.01,0.000189175,545,545,0.00767087,654,0.0250733,646,0.0249887,691,0.0244827,740,0.0240357,692,0.0245058,80001,0,740,0.0129844,740,0.
Orthogroups,Cho-ortho-neAb36,1.00E-03,1.5,33051,Orthogroups_c9ntax36_V2.nexus,,,C9ntax36,Orthogroups_D12ntax36.nexus,,,,,,,,,,,,,,,,,,,,,,,,,,,,,,,,,,,,,,,,,,,,,,,,,,,,,,,,,,,,,,,,,,,,,,,,,,,,,,,,,,,,,,,,,,,,,,,,,,,,,,,,,,,,,,,,,,,,,,,,,,,,,,,,,,,,,,,,,,,,,,,
Orthogroups,OpiAco-ortho-neP38,1.00E-03,1.5,30911,Orthogroups_D10ntax38.nexus,38,,D10ntax38,Orthogroups_D10ntax38_run_1.trees.tree,80001,Orthogroups_D10ntax38_run_2.trees.tree,80001,Orthogroups_D10ntax38_run_3.trees.tree,80001,Orthogroups_D10ntax38_run_4.trees.tree,80001,0,0.000111884,826,844,0.0141849,1729,0.00849483,1663,0.00856768,1924,0.00842115,2182,0.00822161,1939,0.00839636,80001,0,826,0.00689499,826
Orthogroups,OpiAco-ortho-neP41,1.00E-03,1.5,32684,Orthogroups_D10ntax41.nexus,41,,D10ntax41,Orthogroups_D10ntax41_run_1.trees.tree,80001,Orthogroups_D10ntax41_run_2.trees.tree,80001,Orthogroups_D10ntax41_run_3.trees.tree,80001,Orthogroups_D10ntax41_run_4.trees.tree,80001,0,0.00013291,495,791,0.011827,499,0.0202164,495,0.0199852,509,0.0210869,534,0.0217452,510,0.0211143,80001,0,681,0.00864905,681,0.00864981,80
Orthogroups,Opi-ortho-neP44,1.00E-03,1.5,35239,Orthogroups_D10ntax44.nexus,44,,D10ntax44,/home/kjurave/revBayes_run/Folder2SecondStepEnded/D10ntax44/Orthogroups_D10ntax44.nexus,,,,,,,,,,,,,,,,,,,,,,,,,,,,,,,,,,,,,,,,,,,,,,,,,,,,,,,,,,,,,,,,,,,,,,,,,,,,,
Orthogroups,HolXen-ortho-neP32,1.00E-03,1.5,30572,Orthogroups_D11ntax32.nexus,32,,D11ntax32,Orthogroups_D11ntax32_run_1.trees.tree,80001,Orthogroups_D11ntax32_run_2.trees.tree,80001,Orthogroups_D11ntax32_run_3.trees.tree,80001,Orthogroups_D11ntax32_run_4.trees.tree,80001,0,8.70E-05,780,780,0.0200076,2241,0.0140776,2176,0.0140075,2441,0.0141845,2697,0.0144455,2455,0.0141952,80001,0,851,0.0133916,851,0.0133901,
Orthogroups,HolAco-ortho-neP35,1.00E-03,1.5,32276,Orthogroups_D11ntax35.nexus,35,,D11ntax35,Orthogroups_D11ntax35_run_1.trees.tree,80001,Orthogroups_D11ntax35_run_2.trees.tree,80001,Orthogroups_D11ntax35_run_3.trees.tree,80001,Orthogroups_D11ntax35_run_4.trees.tree,80001,0.01,0.000157159,817,817,0.0140753,865,0.0300589,854,0.0299847,901,0.0294506,963,0.0288449,903,0.0294725,80001,0,1455,0.00862869,1455,0.0086
Orthogroups,Hol-ortho-neP38,1.00E-03,1.5,34821,Orthogroups_D11ntax38.nexus,38,,D11ntax38,Orthogroups_D11ntax38.nexus,,,,,,,,,,,,,,,,,,,,,,,,,,,,,,,,,,,,,,,,,,,,,,,,,,,,,,,,,,,,,,,,,,,,,,,,,,,,,,,,,,,,,,,,,,,,,,,,,,,,,,,,,,,,,,,,,,,,,,,,,,,,,,,,,,,,,,,,,,,,,
Orthogroups,ChoXen-ortho-neP30,1.00E-03,1.5,30621,Orthogroups_D12ntax30.nexus,30,,D12ntax30,table.D12ntax30_run_1.trees.tree,:,80001,trees,table.D12ntax30_run_2.trees.tree,:,80001,trees,table.D12ntax30_run_3.trees.tree,:,80001,trees,table.D12ntax30_run_4.trees.tree,:,80001,trees,,,,,0,7.84E-05,608,608,0.0208049,1850,0.0122341,1792,0.0122325,1989,0.0130113,2175,0.013788,2002,0.013056,80001,0,1224,0.00584749,1224,0.00584618
Orthogroups,ChoAco-ortho-neP33,1.00E-03,1.5,32213,Orthogroups_D12ntax33.nexus,33,,D12ntax33,Orthogroups_D12ntax33_run_1.trees.tree,80001,Orthogroups_D12ntax33_run_2.trees.tree,80001,Orthogroups_D12ntax33_run_3.trees.tree,80001,Orthogroups_D12ntax33_run_4.trees.tree,80001,0.01,0.000143664,664,686,0.011481,941,0.00591181,924,0.00585976,1003,0.00609921,1084,0.00625019,1006,0.00610526,80001,0,664,0.00143914,664
Orthogroups,Cho-ortho-neP36,1.00E-03,1.5,34738,Orthogroups_D12ntax36.nexus,36,,D12ntax36,Orthogroups_D12ntax36_run_1.trees.tree,80001,Orthogroups_D12ntax36_run_2.trees.tree,80001,Orthogroups_D12ntax36_run_3.trees.tree,80001,Orthogroups_D12ntax36_run_4.trees.tree,80001,0.00632492,0.000236734,331,366,0.00731292,334,0.0081428,551,0.0104988,331,0.00872326,551,0.0104985,332,0.00872724,80001,0,551,0.0104988,551,0
Orthogroups,41,1.00E-02,1.5,26028,Orthogroups.Results_41.1e-2.I.1.5_Orthogroups.tsv.mcl.out.nexus,41,1_2_3,,,,,,,,,,,,,,,,,,,,,,,,,,,,,,,,,,,,,,,,,,,,,,,,,,,,,,,,,,,,,,,,,,,,,,,,,,,,,,,,,,,,,,,,,,,,,,,,,,,,,,,,,,,,,,,,,,,,,,,,,,,,,,,,,,,,,,,,,,,,,,,,,,,,,,,,,,,,,,,,,
Orthogroups,44,1.00E-02,1.5,32747,Orthogroups.Results_44.1e-2.I.1.5_Orthogroups.tsv.mcl.out.nexus,44,,,44.1e-2.I.1.5_Orthogroups.tsv.mcl.out_run_1.trees.tree,:,80001,trees,44.1e-2.I.1.5_Orthogroups.tsv.mcl.out_run_2.trees.tree,:,80001,trees,44.1e-2.I.1.5_Orthogroups.tsv.mcl.out_run_3.trees.tree,:,80001,trees,44.1e-2.I.1.5_Orthogroups.tsv.mcl.out_run_4.trees.tree,:,80001,trees,0.0933863,0.000813533,458,684,0.00421717,481,0.0134006,479,0.01
Orthogroups,47,1.00E-02,1.5,35446,Orthogroups.1e2.1_5.nexus,47,,,/home/kjurave/revBayes_run/data/Orthogroups.1e2.1_5,../../output/Orthogroups.1e2.1_5_run_1.trees.tree,:,80001,trees,../../output/Orthogroups.1e2.1_5_run_2.trees.tree,:,80001,trees,../../output/Orthogroups.1e2.1_5_run_3.trees.tree,:,77405,trees,../../output/Orthogroups.1e2.1_5_run_4.trees.tree,:,80001,trees,maxdiff,:,0.0284436,meandiff,:,0.000907072,bipartition,list,in,:,bpcomp.bplis
Orthogroups,41,1.00E-12,2,31695,Orthogroups.Results_41.1e-12.I.2_Orthogroups.tsv.mcl.out.nexus,41,,,41.1e-12.I.2_Orthogroups.tsv.mcl.out_run_1.trees.tree,:,80001,trees,41.1e-12.I.2_Orthogroups.tsv.mcl.out_run_2.trees.tree,:,80001,trees,41.1e-12.I.2_Orthogroups.tsv.mcl.out_run_3.trees.tree,:,80001,trees,41.1e-12.I.2_Orthogroups.tsv.mcl.out_run_4.trees.tree,:,80001,trees,0.0284996,0.00030888,848,848,0.0110859,2399,0.00869625,2351,0.00881
Orthogroups,44,1.00E-12,2,40378,Orthogroups.Results_44.1e-12.I.2_Orthogroups.tsv.mcl.out.nexus,44,,,44.1e-12.I.2_Orthogroups.tsv.mcl.out_run_1.trees.tree,:,80001,trees,44.1e-12.I.2_Orthogroups.tsv.mcl.out_run_2.trees.tree,:,80001,trees,44.1e-12.I.2_Orthogroups.tsv.mcl.out_run_3.trees.tree,:,80001,trees,44.1e-12.I.2_Orthogroups.tsv.mcl.out_run_4.trees.tree,:,80001,trees,0.0212372,0.000328922,592,709,0.00894498,612,0.0208502,606,0.020938
Orthogroups,47,1.00E-12,2,44852,Orthogroups.Results_NEW_47.1e-12.I.2_Orthogroups.tsv.mcl.out.nexus,47,,,Orthogroups.Results_NEW_47.1e-12.I.2_Orthogroups.tsv.mcl.out_run_1.trees.tree,:,80001,trees,Orthogroups.Results_NEW_47.1e-12.I.2_Orthogroups.tsv.mcl.out_run_2.trees.tree,:,80001,trees,Orthogroups.Results_NEW_47.1e-12.I.2_Orthogroups.tsv.mcl.out_run_3.trees.tree,:,80001,trees,Orthogroups.Results_NEW_47.1e-12.I.2_Orthogrou
Orthogroups,41,1.00E-09,2,31655,Orthogroups.Results_41.1e-9.I.2_Orthogroups.tsv.mcl.out.nexus,41,,,41.1e-9.I.2_Orthogroups.tsv.mcl.out_run_1.trees.tree,:,80001,trees,41.1e-9.I.2_Orthogroups.tsv.mcl.out_run_2.trees.tree,:,80001,trees,41.1e-9.I.2_Orthogroups.tsv.mcl.out_run_3.trees.tree,:,80001,trees,41.1e-9.I.2_Orthogroups.tsv.mcl.out_run_4.trees.tree,:,80001,trees,0.00573743,0.000103162,777,777,0.00840615,2162,0.0184455,2131,0.0188604,
Orthogroups,44,1.00E-09,2,40382,Orthogroups.Results_44.1e-9.I.2_Orthogroups.tsv.mcl.out.nexus,44,,,44.1e-9.I.2_Orthogroups.tsv.mcl.out_run_1.trees.tree,:,80001,trees,44.1e-9.I.2_Orthogroups.tsv.mcl.out_run_2.trees.tree,:,80001,trees,44.1e-9.I.2_Orthogroups.tsv.mcl.out_run_3.trees.tree,:,80001,trees,44.1e-9.I.2_Orthogroups.tsv.mcl.out_run_4.trees.tree,:,80001,trees,0.00369995,9.15E-05,632,920,0.00188207,704,0.0144025,698,0.0143811,730,0.0
Orthogroups,47,1.00E-09,2,44835,Orthogroups.Results_NEW_47.1e-9.I.2_Orthogroups.tsv.mcl.out.nexus,47,,,Orthogroups.Results_NEW_47.1e-9.I.2_Orthogroups.tsv.mcl.out_run_1.trees.tree,:,80001,trees,Orthogroups.Results_NEW_47.1e-9.I.2_Orthogroups.tsv.mcl.out_run_2.trees.tree,:,80001,trees,Orthogroups.Results_NEW_47.1e-9.I.2_Orthogroups.tsv.mcl.out_run_3.trees.tree,:,80001,trees,Orthogroups.Results_NEW_47.1e-9.I.2_Orthogroups.tsv.
Orthogroups,41,1.00E-05,2,31586,Orthogroups.Results_41.1e-5.I.2_Orthogroups.tsv.mcl.out.nexus,41,,,41.1e-5.I.2_Orthogroups.tsv.mcl.out_run_1.trees.tree,:,80001,trees,41.1e-5.I.2_Orthogroups.tsv.mcl.out_run_2.trees.tree,:,80001,trees,41.1e-5.I.2_Orthogroups.tsv.mcl.out_run_3.trees.tree,:,80001,trees,41.1e-5.I.2_Orthogroups.tsv.mcl.out_run_4.trees.tree,:,80001,trees,0.0380745,0.000329495,870,870,0.0027056,5681,0.0148147,5328,0.0149984,68
Orthogroups,44,1.00E-05,2,40341,Orthogroups.Results_44.1e-5.I.2_Orthogroups.tsv.mcl.out.nexus,44,,,44.1e-5.I.2_Orthogroups.tsv.mcl.out_run_1.trees.tree,:,80001,trees,44.1e-5.I.2_Orthogroups.tsv.mcl.out_run_2.trees.tree,:,80001,trees,44.1e-5.I.2_Orthogroups.tsv.mcl.out_run_3.trees.tree,:,80001,trees,44.1e-5.I.2_Orthogroups.tsv.mcl.out_run_4.trees.tree,:,80001,trees,0.00631242,0.000210855,641,748,0.00380621,647,0.00717021,641,0.00714298,
Orthogroups,47,1.00E-05,2,44808,Orthogroups.Results_NEW_47.1e-5.I.2_Orthogroups.tsv.mcl.out.nexus,47,,,Orthogroups.Results_NEW_47.1e-5.I.2_Orthogroups.tsv.mcl.out_run_1.trees.tree,:,80001,trees,Orthogroups.Results_NEW_47.1e-5.I.2_Orthogroups.tsv.mcl.out_run_2.trees.tree,:,80001,trees,Orthogroups.Results_NEW_47.1e-5.I.2_Orthogroups.tsv.mcl.out_run_3.trees.tree,:,80001,trees,Orthogroups.Results_NEW_47.1e-5.I.2_Orthogroups.tsv.
Orthogroups,41,1.00E-02,2,31573,Orthogroups.Results_41.1e-2.I.2_Orthogroups.tsv.mcl.out.nexus,41,,,41.1e-2.I.2_Orthogroups.tsv.mcl.out_run_1.trees.tree,:,80001,trees,41.1e-2.I.2_Orthogroups.tsv.mcl.out_run_2.trees.tree,:,80001,trees,41.1e-2.I.2_Orthogroups.tsv.mcl.out_run_3.trees.tree,:,80001,trees,41.1e-2.I.2_Orthogroups.tsv.mcl.out_run_4.trees.tree,:,80001,trees,0.0653492,0.000612521,770,770,0.00931935,3215,0.0307441,3148,0.0308607,3
Orthogroups,44,1.00E-02,2,40288,Orthogroups.Results_44.1e-2.I.2_Orthogroups.tsv.mcl.out.nexus,44,,,44.1e-2.I.2_Orthogroups.tsv.mcl.out_run_1.trees.tree,:,80001,trees,44.1e-2.I.2_Orthogroups.tsv.mcl.out_run_2.trees.tree,:,80001,trees,44.1e-2.I.2_Orthogroups.tsv.mcl.out_run_3.trees.tree,:,80001,trees,44.1e-2.I.2_Orthogroups.tsv.mcl.out_run_4.trees.tree,:,80001,trees,0.00474994,0.00017783,576,681,0.0131212,603,0.0113507,596,0.0111748,576,0
Orthogroups,47,1.00E-02,2,44774,Orthogroups.Results_NEW_47.1e-2.I.2_Orthogroups.tsv.mcl.out.nexus,47,1_4,,Orthogroups.Results_NEW_47.1e-2.I.2_Orthogroups.tsv.mcl.out_run_1.trees.tree,:,80001,trees,Orthogroups.Results_NEW_47.1e-2.I.2_Orthogroups.tsv.mcl.out_run_4.trees.tree,:,80001,trees,,,0.19211,0.00396962,257,456,0.00584429,281,0.032209,281,0.0318076,265,0.0384308,257,0.0438669,266,0.0384108,80001,0,689,0.00105732,68
Orthogroups,41,1.00E-12,2.5,34816,Orthogroups.Results_41.1e-12.I.2.5_Orthogroups.tsv.mcl.out.nexus,41,,,41.1e-12.I.2.5_Orthogroups.tsv.mcl.out_run_1.trees.tree,:,80001,trees,41.1e-12.I.2.5_Orthogroups.tsv.mcl.out_run_2.trees.tree,:,80001,trees,41.1e-12.I.2.5_Orthogroups.tsv.mcl.out_run_3.trees.tree,:,80001,trees,41.1e-12.I.2.5_Orthogroups.tsv.mcl.out_run_4.trees.tree,:,80001,trees,0.00102499,8.31E-05,926,926,0.0191205,19556,0.0176522,172
Orthogroups,44,1.00E-12,2.5,45115,Orthogroups.Results_44.1e-12.I.2.5_Orthogroups.tsv.mcl.out.nexus,44,,,44.1e-12.I.2.5_Orthogroups.tsv.mcl.out_run_1.trees.tree,:,80001,trees,44.1e-12.I.2.5_Orthogroups.tsv.mcl.out_run_2.trees.tree,:,80001,trees,44.1e-12.I.2.5_Orthogroups.tsv.mcl.out_run_3.trees.tree,:,80001,trees,44.1e-12.I.2.5_Orthogroups.tsv.mcl.out_run_4.trees.tree,:,80001,trees,0.00574993,0.0001428,612,622,0.0137232,622,0.0183955,616,
Orthogroups,47,1.00E-12,2.5,50843,Orthogroups.Results_NEW_47.1e-12.I.2.5_Orthogroups.tsv.mcl.out.nexus,47,,,Orthogroups.Results_NEW_47.1e-12.I.2.5_Orthogroups.tsv.mcl.out_run_1.trees.tree,:,80001,trees,Orthogroups.Results_NEW_47.1e-12.I.2.5_Orthogroups.tsv.mcl.out_run_2.trees.tree,:,80001,trees,Orthogroups.Results_NEW_47.1e-12.I.2.5_Orthogroups.tsv.mcl.out_run_3.trees.tree,:,80001,trees,Orthogroups.Results_NEW_47.1e-12.I.2.5_
Orthogroups,41,1.00E-09,2.5,34790,Orthogroups.Results_41.1e-9.I.2.5_Orthogroups.tsv.mcl.out.nexus,41,,,41.1e-9.I.2.5_Orthogroups.tsv.mcl.out_run_1.trees.tree,:,80001,trees,41.1e-9.I.2.5_Orthogroups.tsv.mcl.out_run_2.trees.tree,:,80001,trees,41.1e-9.I.2.5_Orthogroups.tsv.mcl.out_run_3.trees.tree,:,80001,trees,41.1e-9.I.2.5_Orthogroups.tsv.mcl.out_run_4.trees.tree,:,80001,trees,0.0262622,0.000236697,820,820,0.0132448,2211,0.00969459,2187,0
Orthogroups,44,1.00E-09,2.5,45111,Orthogroups.Results_44.1e-9.I.2.5_Orthogroups.tsv.mcl.out.nexus,44,,,44.1e-9.I.2.5_Orthogroups.tsv.mcl.out_run_1.trees.tree,:,80001,trees,44.1e-9.I.2.5_Orthogroups.tsv.mcl.out_run_2.trees.tree,:,80001,trees,44.1e-9.I.2.5_Orthogroups.tsv.mcl.out_run_3.trees.tree,:,80001,trees,44.1e-9.I.2.5_Orthogroups.tsv.mcl.out_run_4.trees.tree,:,80001,trees,0.00331246,0.000173309,868,868,0.00288827,972,0.0143545,956,0.0
Orthogroups,47,1.00E-09,2.5,50803,Orthogroups.Results_NEW_47.1e-9.I.2.5_Orthogroups.tsv.mcl.out.nexus,47,,,Orthogroups.Results_NEW_47.1e-9.I.2.5_Orthogroups.tsv.mcl.out_run_1.trees.tree,:,80001,trees,Orthogroups.Results_NEW_47.1e-9.I.2.5_Orthogroups.tsv.mcl.out_run_2.trees.tree,:,80001,trees,Orthogroups.Results_NEW_47.1e-9.I.2.5_Orthogroups.tsv.mcl.out_run_3.trees.tree,:,80001,trees,Orthogroups.Results_NEW_47.1e-9.I.2.5_Ortho
Orthogroups,41,1.00E-05,2.5,34789,Orthogroups.Results_41.1e-5.I.2.5_Orthogroups.tsv.mcl.out.nexus,41,,,41.1e-5.I.2.5_Orthogroups.tsv.mcl.out_run_1.trees.tree,:,80001,trees,41.1e-5.I.2.5_Orthogroups.tsv.mcl.out_run_2.trees.tree,:,80001,trees,41.1e-5.I.2.5_Orthogroups.tsv.mcl.out_run_3.trees.tree,:,80001,trees,41.1e-5.I.2.5_Orthogroups.tsv.mcl.out_run_4.trees.tree,:,80001,trees,0.00277497,9.42E-05,873,873,0.0034129,2595,0.00952088,2562,0.00
Orthogroups,44,1.00E-05,2.5,45124,Orthogroups.Results_44.1e-5.I.2.5_Orthogroups.tsv.mcl.out.nexus,44,,,44.1e-5.I.2.5_Orthogroups.tsv.mcl.out_run_1.trees.tree,:,80001,trees,44.1e-5.I.2.5_Orthogroups.tsv.mcl.out_run_2.trees.tree,:,80001,trees,44.1e-5.I.2.5_Orthogroups.tsv.mcl.out_run_3.trees.tree,:,80001,trees,44.1e-5.I.2.5_Orthogroups.tsv.mcl.out_run_4.trees.tree,:,80001,trees,0.00718741,0.000253992,764,764,0.00958848,810,0.0186576,799,0.0
```

Orthogroups,47,1.00E-05,2.5,50814,Orthogroups.1e5.2_5.nexus,47,,,/home/kjurave/revBayes_run/data/Orthogroups.1e5.2_5,../../output/Orthogroups.1e5.2_5_run_1.trees.tree,:,64334,trees,../../output/Orthogroups.1e5.2_5_run_2.trees.tree,:,64403,trees,../../output/Orthogroups.1e5.2_5_run_3.trees.tree,:,64672,trees,../../output/Orthogroups.1e5.2_5_run_4.trees.tree,:,71597,trees,maxdiff,:,0.00515829,meandiff,:,0.000184442,bipartition,list,in,:,bpcomp.bpl
Orthogroups,41,1.00E-02,2.5,34795,Orthogroups.Results_41.1e-2.I.2.5_Orthogroups.tsv.mcl.out.nexus,41,,,41.1e-2.I.2.5_Orthogroups.tsv.mcl.out_run_1.trees.tree,:,80001,trees,41.1e-2.I.2.5_Orthogroups.tsv.mcl.out_run_2.trees.tree,:,80001,trees,41.1e-2.I.2.5_Orthogroups.tsv.mcl.out_run_3.trees.tree,:,80001,trees,41.1e-2.I.2.5_Orthogroups.tsv.mcl.out_run_4.trees.tree,:,80001,trees,0.0534493,0.000525804,873,873,0.00563641,2353,0.010899,2321,0.0
Orthogroups,44,1.00E-02,2.5,45084,Orthogroups.Results_44.1e-2.I.2.5_Orthogroups.tsv.mcl.out.nexus,44,,,44.1e-2.I.2.5_Orthogroups.tsv.mcl.out_run_1.trees.tree,:,80001,trees,44.1e-2.I.2.5_Orthogroups.tsv.mcl.out_run_2.trees.tree,:,80001,trees,44.1e-2.I.2.5_Orthogroups.tsv.mcl.out_run_3.trees.tree,:,80001,trees,44.1e-2.I.2.5_Orthogroups.tsv.mcl.out_run_4.trees.tree,:,80001,trees,0.00432495,0.000173919,762,762,0.00802997,790,0.0319958,780,0.0
Orthogroups,47,1.00E-02,2.5,50785,Orthogroups.Results_NEW_47.1e-2.I.2.5_Orthogroups.tsv.mcl.out.nexus,47,,,Orthogroups.Results_NEW_47.1e-2.I.2.5_Orthogroups.tsv.mcl.out_run_1.trees.tree,:,80001,trees,Orthogroups.Results_NEW_47.1e-2.I.2.5_Orthogroups.tsv.mcl.out_run_2.trees.tree,:,80001,trees,Orthogroups.Results_NEW_47.1e-2.I.2.5_Orthogroups.tsv.mcl.out_run_3.trees.tree,:,80001,trees,Orthogroups.Results_NEW_47.1e-2.I.2.5_Ortho
Orthogroups,41,1.00E-12,4,40459,Orthogroups.Results_41.1e-12.I.4_Orthogroups.tsv.mcl.out.nexus,41,,,41.1e-12.I.4_Orthogroups.tsv.mcl.out_run_1.trees.tree,:,80001,trees,41.1e-12.I.4_Orthogroups.tsv.mcl.out_run_2.trees.tree,:,80001,trees,41.1e-12.I.4_Orthogroups.tsv.mcl.out_run_3.trees.tree,:,80001,trees,41.1e-12.I.4_Orthogroups.tsv.mcl.out_run_4.trees.tree,:,80001,trees,0.00172498,7.34E-05,927,927,0.00585944,6630,0.00329876,6482,0.00330
Orthogroups,44,1.00E-12,4,53373,Orthogroups.Results_44.1e-12.I.4_Orthogroups.tsv.mcl.out.nexus,44,,,44.1e-12.I.4_Orthogroups.tsv.mcl.out_run_1.trees.tree,:,80001,trees,44.1e-12.I.4_Orthogroups.tsv.mcl.out_run_2.trees.tree,:,80001,trees,44.1e-12.I.4_Orthogroups.tsv.mcl.out_run_3.trees.tree,:,80001,trees,44.1e-12.I.4_Orthogroups.tsv.mcl.out_run_4.trees.tree,:,80001,trees,0.00309996,9.22E-05,564,602,0.0107156,565,0.0161383,564,0.016103,57
Orthogroups,47,1.00E-12,4,61587,Orthogroups.Results_NEW_47.1e-12.I.4_Orthogroups.tsv.mcl.out.nexus,47,1_4,,Orthogroups.Results_NEW_47.1e-12.I.4_Orthogroups.tsv.mcl.out_run_1.trees.tree,:,80001,trees,Orthogroups.Results_NEW_47.1e-12.I.4_Orthogroups.tsv.mcl.out_run_4.trees.tree,:,80001,trees,,,0.00472494,0.000260904,281,380,0.00710328,286,0.0300776,284,0.0300449,281,0.0296363,283,0.0290313,282,0.0296579,80001,0,444,0.02
Orthogroups,41,1.00E-09,4,40449,Orthogroups.Results_41.1e-9.I.4_Orthogroups.tsv.mcl.out.nexus,41,,,41.1e-9.I.4_Orthogroups.tsv.mcl.out_run_1.trees.tree,:,80001,trees,41.1e-9.I.4_Orthogroups.tsv.mcl.out_run_2.trees.tree,:,80001,trees,41.1e-9.I.4_Orthogroups.tsv.mcl.out_run_3.trees.tree,:,80001,trees,41.1e-9.I.4_Orthogroups.tsv.mcl.out_run_4.trees.tree,:,80001,trees,0.00379995,9.34E-05,770,770,0.0068518,2555,0.00671521,2531,0.00671691,27
Orthogroups,44,1.00E-09,4,53306,Orthogroups.Results_44.1e-9.I.4_Orthogroups.tsv.mcl.out.nexus,44,,,44.1e-9.I.4_Orthogroups.tsv.mcl.out_run_1.trees.tree,:,80001,trees,44.1e-9.I.4_Orthogroups.tsv.mcl.out_run_2.trees.tree,:,80001,trees,44.1e-9.I.4_Orthogroups.tsv.mcl.out_run_3.trees.tree,:,80001,trees,44.1e-9.I.4_Orthogroups.tsv.mcl.out_run_4.trees.tree,:,80001,trees,0.00624992,9.49E-05,445,649,0.00533226,806,0.00805136,801,0.00802532,868
Orthogroups,47,1.00E-09,4,61521,Orthogroups.Results_NEW_47.1e-9.I.4_Orthogroups.tsv.mcl.out.nexus,47,1_3,,Orthogroups.Results_NEW_47.1e-9.I.4_Orthogroups.tsv.mcl.out_run_1.trees.tree,:,80001,trees,Orthogroups.Results_NEW_47.1e-9.I.4_Orthogroups.tsv.mcl.out_run_3.trees.tree,:,80001,trees,,,0.00518744,0.000181775,240,351,0.00992021,242,0.00465111,240,0.004586,240,0.0048639,244,0.00523964,240,0.00486834,80001,0,352,0.0067
Orthogroups,41,1.00E-05,4,40486,Orthogroups.Results_41.1e-5.I.4_Orthogroups.tsv.mcl.out.nexus,41,,,41.1e-5.I.4_Orthogroups.tsv.mcl.out_run_1.trees.tree,:,80001,trees,41.1e-5.I.4_Orthogroups.tsv.mcl.out_run_2.trees.tree,:,80001,trees,41.1e-5.I.4_Orthogroups.tsv.mcl.out_run_3.trees.tree,:,80001,trees,41.1e-5.I.4_Orthogroups.tsv.mcl.out_run_4.trees.tree,:,80001,trees,0.00876239,0.000115696,865,865,0.00999382,14687,0.0137145,14376,0.01353
Orthogroups,44,1.00E-05,4,53378,Orthogroups.Results_44.1e-5.I.4_Orthogroups.tsv.mcl.out.nexus,44,1_3_4,,44.1e-5.I.4_Orthogroups.tsv.mcl.out_run_1.trees.tree,:,80001,trees,44.1e-5.I.4_Orthogroups.tsv.mcl.out_run_3.trees.tree,:,80001,trees,44.1e-5.I.4_Orthogroups.tsv.mcl.out_run_4.trees.tree,:,80001,trees,,0.0942238,0.00103079,729,729,0.00142803,783,0.00303349,774,0.00316577,774,0.0036184,793,0.00397889,775,0.00359269,80001,0,1661
Orthogroups,47,1.00E-05,4,61578,Orthogroups.1e5.4.nexus,47,,,/home/kjurave/revBayes_run/data/Orthogroups.1e5.4,../../output/Orthogroups.1e5.4_run_1.trees.tree,:,65851,trees,../../output/Orthogroups.1e5.4_run_2.trees.tree,:,66237,trees,../../output/Orthogroups.1e5.4_run_3.trees.tree,:,77960,trees,../../output/Orthogroups.1e5.4_run_4.trees.tree,:,77267,trees,maxdiff,:,0.0187441,meandiff,:,0.000362225,bipartition,list,in,:,bpcomp.bplist,consensus,in,:,
Orthogroups,41,1.00E-02,4,40534,Orthogroups.Results_41.1e-2.I.4_Orthogroups.tsv.mcl.out.nexus,41,,,41.1e-2.I.4_Orthogroups.tsv.mcl.out_run_1.trees.tree,:,80001,trees,41.1e-2.I.4_Orthogroups.tsv.mcl.out_run_2.trees.tree,:,80001,trees,41.1e-2.I.4_Orthogroups.tsv.mcl.out_run_3.trees.tree,:,80001,trees,41.1e-2.I.4_Orthogroups.tsv.mcl.out_run_4.trees.tree,:,80001,trees,0.00133748,5.69E-05,707,707,0.0123809,3823,0.0101117,3785,0.0102243,4142,
Orthogroups,44,1.00E-02,4,53379,Orthogroups.Results_44.1e-2.I.4_Orthogroups.tsv.mcl.out.nexus,44,,,44.1e-2.I.4_Orthogroups.tsv.mcl.out_run_1.trees.tree,:,80001,trees,44.1e-2.I.4_Orthogroups.tsv.mcl.out_run_2.trees.tree,:,80001,trees,44.1e-2.I.4_Orthogroups.tsv.mcl.out_run_3.trees.tree,:,80001,trees,44.1e-2.I.4_Orthogroups.tsv.mcl.out_run_4.trees.tree,:,80001,trees,0.0158998,0.00030697,635,635,0.0103241,970,0.018086,962,0.0178754,978,0.0
Orthogroups,47,1.00E-02,4,61582,Orthogroups.Results_NEW_47.1e-2.I.4_Orthogroups.tsv.mcl.out.nexus,47,1_2_3,,Orthogroups.Results_NEW_47.1e-2.I.4_Orthogroups.tsv.mcl.out_run_1.trees.tree,:,80001,trees,Orthogroups.Results_NEW_47.1e-2.I.4_Orthogroups.tsv.mcl.out_run_2.trees.tree,:,80001,trees,Orthogroups.Results_NEW_47.1e-2.I.4_Orthogroups.tsv.mcl.out_run_3.trees.tree,:,80001,trees,,0.00294996,9.45E-05,265,265,0.000554936,297,0
Orthogroups,41,1.00E-12,6,44609,Orthogroups.Results_41.1e-12.I.6_Orthogroups.tsv.mcl.out.nexus,41,,,41.1e-12.I.6_Orthogroups.tsv.mcl.out_run_1.trees.tree,:,80001,trees,41.1e-12.I.6_Orthogroups.tsv.mcl.out_run_2.trees.tree,:,80001,trees,41.1e-12.I.6_Orthogroups.tsv.mcl.out_run_3.trees.tree,:,80001,trees,41.1e-12.I.6_Orthogroups.tsv.mcl.out_run_4.trees.tree,:,80001,trees,0.00114999,7.94E-05,681,681,0.0165961,4125,0.0274184,4108,0.0274038
Orthogroups,44,1.00E-12,6,58827,Orthogroups.Results_44.1e-12.I.6_Orthogroups.tsv.mcl.out.nexus,44,,,44.1e-12.I.6_Orthogroups.tsv.mcl.out_run_1.trees.tree,:,80001,trees,44.1e-12.I.6_Orthogroups.tsv.mcl.out_run_2.trees.tree,:,80001,trees,44.1e-12.I.6_Orthogroups.tsv.mcl.out_run_3.trees.tree,:,80001,trees,44.1e-12.I.6_Orthogroups.tsv.mcl.out_run_4.trees.tree,:,80001,trees,0.0131248,0.000174274,615,636,0.00614423,619,0.0267108,615,0.02665
Orthogroups,47,1.00E-12,6,68755,Orthogroups.1e12.6.nexus,47,1_2,,/home/kjurave/revBayes_run/data/Orthogroups.1e12.6/1_2,../../../output/Orthogroups.1e12.6_run_1.trees.tree,:,54076,trees,../../../output/Orthogroups.1e12.6_run_2.trees.tree,:,59028,trees,maxdiff,:,0.00532125,meandiff,:,0.000231251,bipartition,list,in,:,bpcomp.bplist,consensus,in,:,bpcomp.con.tre,name,effsize,rel_diff,Prior,195,0.000342254,alpha,163,0.033884,gamma_rates[1],161,0.
Orthogroups,41,1.00E-09,6,44634,Orthogroups.Results_41.1e-9.I.6_Orthogroups.tsv.mcl.out.nexus,41,,,41.1e-9.I.6_Orthogroups.tsv.mcl.out_run_1.trees.tree,:,80001,trees,41.1e-9.I.6_Orthogroups.tsv.mcl.out_run_2.trees.tree,:,80001,trees,41.1e-9.I.6_Orthogroups.tsv.mcl.out_run_3.trees.tree,:,80001,trees,41.1e-9.I.6_Orthogroups.tsv.mcl.out_run_4.trees.tree,:,80001,trees,0.429282,0.00277213,846,846,0.00157234,13039,0.0970543,12865,0.0979745,1
Orthogroups,44,1.00E-09,6,58839,Orthogroups.Results_44.1e-9.I.6_Orthogroups.tsv.mcl.out.nexus,44,,,44.1e-9.I.6_Orthogroups.tsv.mcl.out_run_1.trees.tree,:,80001,trees,44.1e-9.I.6_Orthogroups.tsv.mcl.out_run_2.trees.tree,:,80001,trees,44.1e-9.I.6_Orthogroups.tsv.mcl.out_run_3.trees.tree,:,80001,trees,44.1e-9.I.6_Orthogroups.tsv.mcl.out_run_4.trees.tree,:,80001,trees,0.0156248,0.000150601,520,606,0.00536582,541,0.0184989,533,0.018488,521,0
Orthogroups,47,1.00E-09,6,68771,Orthogroups.Results_NEW_47.1e-9.I.6_Orthogroups.tsv.mcl.out.nexus,47,2_3,,Orthogroups.Results_NEW_47.1e-9.I.6_Orthogroups.tsv.mcl.out_run_2.trees.tree,:,80001,trees,Orthogroups.Results_NEW_47.1e-9.I.6_Orthogroups.tsv.mcl.out_run_3.trees.tree,:,80001,trees,,,0.0077874,0.000471629,208,255,0.0292732,210,0.0593716,209,0.0588241,208,0.0571459,209,0.0549697,208,0.0572792,80001,0,344,0.0392715,
Orthogroups,41,1.00E-05,6,44722,Orthogroups.Results_41.1e-5.I.6_Orthogroups.tsv.mcl.out.nexus,41,,,41.1e-5.I.6_Orthogroups.tsv.mcl.out_run_1.trees.tree,:,80001,trees,41.1e-5.I.6_Orthogroups.tsv.mcl.out_run_2.trees.tree,:,80001,trees,41.1e-5.I.6_Orthogroups.tsv.mcl.out_run_3.trees.tree,:,80001,trees,41.1e-5.I.6_Orthogroups.tsv.mcl.out_run_4.trees.tree,:,80001,trees,0.00739991,0.00012083,854,966,0.00493235,11714,0.0171251,11600,0.0172147
Orthogroups,44,1.00E-05,6,58922,Orthogroups.Results_44.1e-5.I.6_Orthogroups.tsv.mcl.out.nexus,44,,,44.1e-5.I.6_Orthogroups.tsv.mcl.out_run_1.trees.tree,:,80001,trees,44.1e-5.I.6_Orthogroups.tsv.mcl.out_run_2.trees.tree,:,80001,trees,44.1e-5.I.6_Orthogroups.tsv.mcl.out_run_3.trees.tree,:,80001,trees,44.1e-5.I.6_Orthogroups.tsv.mcl.out_run_4.trees.tree,:,80001,trees,0.00662492,0.000138825,652,670,0.00595426,656,0.0161251,652,0.0157771,66
Orthogroups,47,1.00E-05,6,68810,Orthogroups.Results_NEW_47.1e-5.I.6_Orthogroups.tsv.mcl.out.nexus,47,1_4,,/home/kjurave/revBayes_run/data/ZIP/Orthogroups.Results_NEW_47.1e-5.I.6_Orthogroups.tsv.mcl.out.Oxy,,,,,,,,,,,,,,,,,,,,,,,,,,,,,"""(ACDI:0.001564",NEVE:0.001668,(HYMA:0.002822,(((TRAD:0.00237,HOHO:0.000995)0.99:0.002377,((PLBA:0.00355,MNLE:0.001866)0.99:0.003135,((OSCA:0.004333,(SYCI:0.002518,(TEWI:0.002039,AMQU:
Orthogroups,41,1.00E-02,6,44743,Orthogroups.Results_41.1e-2.I.6_Orthogroups.tsv.mcl.out.nexus,41,,,41.1e-2.I.6_Orthogroups.tsv.mcl.out_run_1.trees.tree,:,80001,trees,41.1e-2.I.6_Orthogroups.tsv.mcl.out_run_2.trees.tree,:,80001,trees,41.1e-2.I.6_Orthogroups.tsv.mcl.out_run_3.trees.tree,:,80001,trees,41.1e-2.I.6_Orthogroups.tsv.mcl.out_run_4.trees.tree,:,80001,trees,0.00076249,6.59E-05,829,829,0.00422472,26323,0.00771508,26231,0.0077899
Orthogroups,44,1.00E-02,6,58956,Orthogroups.Results_44.1e-2.I.6_Orthogroups.tsv.mcl.out.nexus,44,,,44.1e-2.I.6_Orthogroups.tsv.mcl.out_run_1.trees.tree,:,80001,trees,44.1e-2.I.6_Orthogroups.tsv.mcl.out_run_2.trees.tree,:,80001,trees,44.1e-2.I.6_Orthogroups.tsv.mcl.out_run_3.trees.tree,:,79934,trees,44.1e-2.I.6_Orthogroups.tsv.mcl.out_run_4.trees.tree,:,80001,trees,0.00176235,0.000102854,676,676,0.00941177,1230,0.021114,1225,0.0211711,12
Orthogroups,47,1.00E-02,6,68819,Orthogroups.1e2.6.nexus,47,3_4,,/home/kjurave/revBayes_run/data/Orthogroups.1e2.6/3_4,../../../output/Orthogroups.1e2.6_run_3.trees.tree,:,49878,trees,../../../output/Orthogroups.1e2.6_run_4.trees.tree,:,50168,trees,maxdiff,:,6.61E-05,meandiff,:,4.82E-06,bipartition,list,in,:,bpcomp.bplist,consensus,in,:,bpcomp.con.tre,name,effsize,rel_diff,Prior,209,0,alpha,215,0,gamma_rates[1],213,0,gamma_rates[2],212,0,gamma_
,,,,,,,,,,,,,,,,,,,,,,,,,,,,,,,,,,,,,,,,,,,,,,,,,,,,,,,,,,,,,,,,,,,,,,,,,,,,,,,,
,,,,,,,,,,,,,,,,,,,,,,,,,,,,,,,,,,,,,,,,,,,,,,,,,,,,,,,,,,,,,,,,,,,,,,,,,,,,,,,,
,,,,,,,,,,,,,,,,,,,,,,,,,,,,,,,,,,,,,,,,,,,,,,,,,,,,,,,,,,,,,,,,,,,,,,,,,,,,,,,,
,,,,,,,,,,,,,,,,,,,,,,,,,,,,,,,,,,,,,,,,,,,,,,,,,,,,,,,,,,,,,,,,,,,,,,,,,,,,,,,,
,,,,,,,,,,,,,,,,,,,,,,,,,,,,,,,,,,,,,,,,,,,,,,,,,,,,,,,,,,,,,,,,,,,,,,,,,,,,,,,,
,,,,,,,,,,,,,,,,,,,,,,,,,,,,,,,,,,,,,,,,,,,,,,,,,,,,,,,,,,,,,,,,,,,,,,,,,,,,,,,,
,,,,,,,,,,,,,,,,,,,,,,,,,,,,,,,,,,,,,,,,,,,,,,,,,,,,,,,,,,,,,,,,,,,,,,,,,,,,,,,,
,,,,,,,,,,,,,,,,,,,,,,,,,,,,,,,,,,,,,,,,,,,,,,,,,,,,,,,,,,,,,,,,,,,,,,,,,,,,,,,,
,,,,,,,,,,,,,,,,,,,,,,,,,,,,,,,,,,,,,,,,,,,,,,,,,,,,,,,,,,,,,,,,,,,,,,,,,,,,,,,,
,,,,,,,,,,,,,,,,,,,,,,,,,,,,,,,,,,,,,,,,,,,,,,,,,,,,,,,,,,,,,,,,,,,,,,,,,,,,,,,,
,,,,,,,,,,,,,,,,,,,,,,,,,,,,,,,,,,,,,,,,,,,,,,,,,,,,,,,,,,,,,,,,,,,,,,,,,,,,,,,,
,,,,,,,,,,,,,,,,,,,,,,,,,,,,,,,,,,,,,,,,,,,,,,,,,,,,,,,,,,,,,,,,,,,,,,,,,,,,,,,,
,,,,,,,,,,,,,,,,,,,,,,,,,,,,,,,,,,,,,,,,,,,,,,,,,,,,,,,,,,,,,,,,,,,,,,,,,,,,,,,,
,,,,,,,,,,,,,,,,,,,,,,,,,,,,,,,,,,,,,,,,,,,,,,,,,,,,,,,,,,,,,,,,,,,,,,,,,,,,,,,,
,,,,,,,,,,,,,,,,,,,,,,,,,,,,,,,,,,,,,,,,,,,,,,,,,,,,,,,,,,,,,,,,,,,,,,,,,,,,,,,,
,,,,,,,,,,,,,,,,,,,,,,,,,,,,,,,,,,,,,,,,,,,,,,,,,,,,,,,,,,,,,,,,,,,,,,,,,,,,,,,,
,,,,,,,,,,,,,,,,,,,,,,,,,,,,,,,,,,,,,,,,,,,,,,,,,,,,,,,,,,,,,,,,,,,,,,,,,,,,,,,,
,,,,,,,,,,,,,,,,,,,,,,,,,,,,,,,,,,,,,,,,,,,,,,,,,,,,,,,,,,,,,,,,,,,,,,,,,,,,,,,,
,,,,,,,,,,,,,,,,,,,,,,,,,,,,,,,,,,,,,,,,,,,,,,,,,,,,,,,,,,,,,,,,,,,,,,,,,,,,,,,,
,,,,,,,,,,,,,,,,,,,,,,,,,,,,,,,,,,,,,,,,,,,,,,,,,,,,,,,,,,,,,,,,,,,,,,,,,,,,,,,,
,,,,,,,,,,,,,,,,,,,,,,,,,,,,,,,,,,,,,,,,,,,,,,,,,,,,,,,,,,,,,,,,,,,,,,,,,,,,,,,,
,,,,,,,,,,,,,,,,,,,,,,,,,,,,,,,,,,,,,,,,,,,,,,,,,,,,,,,,,,,,,,,,,,,,,,,,,,,,,,,,
,,,,,,,,,,,,,,,,,,,,,,,,,,,,,,,,,,,,,,,,,,,,,,,,,,,,,,,,,,,,,,,,,,,,,,,,,,,,,,,,
,,,,,,,,,,,,,,,,,,,,,,,,,,,,,,,,,,,,,,,,,,,,,,,,,,,,,,,,,,,,,,,,,,,,,,,,,,,,,,,,
,,,,,,,,,,,,,,,,,,,,,,,,,,,,,,,,,,,,,,,,,,,,,,,,,,,,,,,,,,,,,,,,,,,,,,,,,,,,,,,,
,,,,,,,,,,,,,,,,,,,,,,,,,,,,,,,,,,,,,,,,,,,,,,,,,,,,,,,,,,,,,,,,,,,,,,,,,,,,,,,,
,,,,,,,,,,,,,,,,,,,,,,,,,,,,,,,,,,,,,,,,,,,,,,,,,,,,,,,,,,,,,,,,,,,,,,,,,,,,,,,,
,,,,,,,,,,,,,,,,,,,,,,,,,,,,,,,,,,,,,,,,,,,,,,,,,,,,,,,,,,,,,,,,,,,,,,,,,,,,,,,,
,,,,,,,,,,,,,,,,,,,,,,,,,,,,,,,,,,,,,,,,,,,,,,,,,,,,,,,,,,,,,,,,,,,,,,,,,,,,,,,,
,,,,,,,,,,,,,,,,,,,,,,,,,,,,,,,,,,,,,,,,,,,,,,,,,,,,,,,,,,,,,,,,,,,,,,,,,,,,,,,,
,,,,,,,,,,,,,,,,,,,,,,,,,,,,,,,,,,,,,,,,,,,,,,,,,,,,,,,,,,,,,,,,,,,,,,,,,,,,,,,,
,,,,,,,,,,,,,,,,,,,,,,,,,,,,,,,,,,,,,,,,,,,,,,,,,,,,,,,,,,,,,,,,,,,,,,,,,,,,,,,,
,,,,,,,,,,,,,,,,,,,,,,,,,,,,,,,,,,,,,,,,,,,,,,,,,,,,,,,,,,,,,,,,,,,,,,,,,,,,,,,,
,,,,,,,,,,,,,,,,,,,,,,,,,,,,,,,,,,,,,,,,,,,,,,,,,,,,,,,,,,,,,,,,,,,,,,,,,,,,,,,,
,,,,,,,,,,,,,,,,,,,,,,,,,,,,,,,,,,,,,,,,,,,,,,,,,,,,,,,,,,,,,,,,,,,,,,,,,,,,,,,,
,,,,,,,,,,,,,,,,,,,,,,,,,,,,,,,,,,,,,,,,,,,,,,,,,,,,,,,,,,,,,,,,,,,,,,,,,,,,,,,,
,,,,,,,,,,,,,,,,,,,,Choanoflagellata,Capsaspora/,Sphearoforma,Fungi,,,,,,,,,,,,,,,,,,,,,,,,,,,,,,,,,,,,,,,,,,,,,,,,,,,,,,,,,,,,,,,
,,,,,,,,,,,,,,,,Orthogroups,Homogroups,,,,,,,,,,,,,,,,,,,,,,,,,,,,,,,,,,,,,,,,,,,,,,,,,,,,,,,,,,,,,,,
,,,,,,,,,,,,,,OAbiXen-disAb41,,26063,26575,,,,,,,,,,,,,,,,,,,,,,,,,,,,,,,,,,,,,,,,,,,,,,,,,,,,,,,,,,,,,,
,,,,,,,,,,,,,,OAbiAco-disAb44,,35455,31284,,,,,,,Fungi+Capsaspora+Choanoflagellata,,,,,,,,,,,,,,,,,,,,,,,,,,,,,,,,,,,,,,,,,,,,,,,,,,,,,,,
,,,,,,,,,,,,,,OAbi-disAb47,,35455,32795,,,,,,,Capsaspora+Choanoflagellata,,,,,,,,,,,,,,,,,,,,,,,,,,,,,,,,,,,,,,,,,,,,,,,,,,,,,,,
,,,,,,,,,,,,,,HolXen-disAb33,,25104,25104,,,,,,,Choanoflagellata,,,,,,,,,,,,,,,,,,,,,,,,,,,,,,,,,,,,,,,,,,,,,,,,,,,,,,,,,
,,,,,,,,,,,,,,HolAco-disAb36,,31885,29318,,,,,,,,,,,,,,,,,,,,,,,,,,,,,,,,,,,,,,,,,,,,,,,,,,,,,,,,,,,,,
,,,,,,,,,,,,,,Hol-disAb39,,34495,30816,,,,,,,,,,,,,,,,,,,,,,,,,,,,,,,,,,,,,,,,,,,,,,,,,,,,,,,,,,,,,
,,,,,,,,,,,,,,ChoXen-disAb38,,25050,24302,,,,,,,,,,,,,,,,,,,,,,,,,,,,,,,,,,,,,,,,,,,,,,,,,,,,,,,,,,,,,
,,,,,,,,,,,,,,ChoAco-disAb35,,31786,29017,,,,,,,,,,,,,,,,,,,,,,,,,,,,,,,,,,,,,,,,,,,,,,,,,,,,,,,,,,,,,
,,,,,,,,,,,,,,Cho-disAb38,,34755,30505,,,,,,,,,,,H_41,O_41,H_44,O_44,H_47,O_47,,,,,,,,,,,,,,,,,,,,,,,,,,,,,,,,,,,,,,,,,,,,,,,,,,,,,
,,,,,,,,,,,,,,OpiXen-disP41,Pruned,matrix,31067,30477,,,,,,,,,,1.5,1.00E-12,24545,26167,27784,32858,28679,35584,,,,,,,,,,,,,,,,,,,,,,,,,,,,,,,,,,,,,,,,,,,,,,,,,,,,,
,,,,,,,,,,,,,,OpiAco-disP44,,32886,31557,,,,,,,,,,2,,29332,31695,33188,40378,34487,44852,,,,,,,,,,,,,,,,,,,,,,,,,,,,,,,,,,,,,,,,,,,,,,,,
,,,,,,,,,,,,,,HolXen-disP33,,30717,29994,,,,,,,,,,2.5,,32109,34816,36369,45115,37860,50843,,,,,,,,,,,,,,,,,,,,,,,,,,,,,,,,,,,,,,,,,,,,,,,,,,
,,,,,,,,,,,,,,HolAco-disP36,,32469,30992,,,,,,,,,,4,,37121,40459,42071,53373,44138,61587,,,,,,,,,,,,,,,,,,,,,,,,,,,,,,,,,,,,,,,,,,,,,,,,,
,,,,,,,,,,,,,,Hol-disP39,,34495,32203,,,,,,,,,,6,,40738,44609,46123,58827,48738,68755,,,,,,,,,,,,,,,,,,,,,,,,,,,,,,,,,,,,,,,,,,,,,,,
,,,,,,,,,,,,,,ChoXen-disP38,,30751,30055,,,,,,,,,,1.5,1.00E-09,24996,26028,28599,32747,29656,35446,,,,,,,,,,,,,,,,,,,,,,,,,,,,,,,,,,,,,,,,,,,,,,,,,,,,
,,,,,,,,,,,,,,ChoAco-disP35,,32400,30963,,,,,,,,,,2,,30103,31573,34517,40288,36011,44774,,,,,,,,,,,,,,,,,,,,,,,,,,,,,,,,,,,,,,,,,,,,,,,,,

| |
|---|
| ,,,,,,,,,,,,,,Cho-disP38,,34941,32171,,,,,,,,,2.5,,32986,34795,37802,45084,39589,50785,,,,,,,,,,,,,,,,,,,,,,,,,,,,,,,,,,,,, |
| ,,,,,,,,,,,,,,OpiXen-neAb35,No,long,branch,species,24683,24904,,,,,,,,,4,,38154,40534,43763,53379,46181,61582,,,,,,,,,,,,,,,,,,,,,,,,,,,,,,,,,,,,, |
| ,,,,,,,,,,,,,,OpiAc-neAb38,,26063,29636,,,,,,,,,6,,41957,44743,48076,58956,51115,68819,,,,,,,,,,,,,,,,,,,,,,,,,,,,,,,,,,,,, |
| ,,,,,,,,,,,,,,Opi-neAb41,,34023,31119,,,,,,,,,1.5,1.00E-05,25934,26066,30267,32793,31634,35502,,,,,,,,,,,,,,,,,,,,,,,,,,,,,,,,,,,,, |
| ,,,,,,,,,,,,,,HolXen-neAb38,,23716,22970,,,,,,,,,2,,31449,31586,36817,40341,38721,44808,,,,,,,,,,,,,,,,,,,,,,,,,,,,,,,,,,,,, |
| ,,,,,,,,,,,,,,HolAco-neAb41,,30435,27684,,,,,,,,,2.5,,34553,34789,40465,45124,42746,50814,,,,,,,,,,,,,,,,,,,,,,,,,,,,,,,,,,,,, |
| ,,,,,,,,,,,,,,Hol-neAb44,,33072,29134,,,,,,,,,4,,40070,40486,46871,53378,49865,61578,,,,,,,,,,,,,,,,,,,,,,,,,,,,,,,,,,,,, |
| ,,,,,,,,,,,,,,ChoXen-neAb30,,23703,22662,,,,,,,,,6,,43897,44722,51428,58922,55118,68810,,,,,,,,,,,,,,,,,,,,,,,,,,,,,,,,,,,,, |
| ,,,,,,,,,,,,,,ChoAco-neAb33,,30411,27366,,,,,,,,,1.5,0.01,26978,26125,31891,32852,33506,35578,,,,,,,,,,,,,,,,,,,,,,,,,,,,,,,,,,,,, |
| ,,,,,,,,,,,,,,Cho-neAb36,,34738,28828,,,,,,,,,2,,32804,31655,38977,40382,41248,44835,,,,,,,,,,,,,,,,,,,,,,,,,,,,,,,,,,,,, |
| ,,,,,,,,,,,,,,OpiXen-neP35,"""No",long,branch,species,,Pruned,"matrix""",30911,30280,,,,,,,,,2.5,,36015,34790,42881,45111,45532,50803,,,,,,,,,,,,,,,,,,,,,,,,,,,,,,,,,,,,, |
| ,,,,,,,,,,,,,,OpiAc-neP38,,32684,31317,,,,,,,,,4,,41673,40449,49635,53306,53090,61521,,,,,,,,,,,,,,,,,,,,,,,,,,,,,,,,,,,,, |
| ,,,,,,,,,,,,,,Opi-neP41,,35239,32544,,,,,,,,,6,,45671,44634,54447,58839,58664,68771,,,,,,,,,,,,,,,,,,,,,,,,,,,,,,,,,,,,, |
| ,,,,,,,,,,,,,,HolXen-neP38,,30572,29822,,,,,,,,,,,,,,,,,,,,,,,,,,,,,,,,,,,,,,,,,,,,,,,,,,,, |
| ,,,,,,,,,,,,,,HolAco-neP41,,32276,30774,,,,,,,,,,,,,,,,,,,,,,,,,,,,,,,,,,,,,,,,,,,,,,,,,,,, |
| ,,,,,,,,,,,,,,Hol-neP44,,34821,31974,,,,,,,,,,,,,,,,,,,,,,,,,,,,,,,,,,,,,,,,,,,,,,,,,,,, |
| ,,,,,,,,,,,,,,ChoXen-neP30,,30621,29905,,,,,,,,,,,,,,,,,,,,,,,,,,,,,,,,,,,,,,,,,,,,,,,,,,,, |
| ,,,,,,,,,,,,,,ChoAco-neP33,,32213,30766,,,,,,,,,,,,,,,,,,,,,,,,,,,,,,,,,,,,,,,,,,,,,,,,,,,, |
| ,,,,,,,,,,,,,,Cho-neP36,,34738,31964,,,,,,,,,,,,,,,,,,,,,,,,,,,,,,,,,,,,,,,,,,,,,,,,,,,, |
