# Supplementary material for: Exploring genome gene content and morphological analysis to test recalcitrant nodes in the animal phylogeny
Source: PLoS One. 2023 Mar 23;18(3):e0282444. doi: 10.1371/journal.pone.0282444 (PMC10035847; doi:10.1371/journal.pone.0282444)
Supplement: S3 Table — (PDF) [file pone.0282444.s017.pdf]

Table S3 | The summary of all datasets settings and results for run 2, summary of all the details and the most probable tree for each of the 190 datasets tested, count of the support for each of the unique topologies observed by the individual posterior trees.

,,

,,,,,,,,,,,,,,,,#of trees run,#of trees run,effsize,effsize,effsize,effsize,effsize,effsize,effsize,effsize,effsize,effsize,rel_diff,rel_diff,rel_diff,rel_diff,rel_diff,rel_diff,rel_diff,rel_diff,rel_diff,rel_diff,,,Tree,Collapsed tree,Collapsed tree short format,Smallest pp value in tree,plase of smallest pp,The main topologies found and support of each dataset count,,,,,,,,,,,,,,,,,,,,,,,,,,,,

Homogroups/Orthogroups,Species sample,File name in Table 4,#,SP # (see Genome gene content datasets protocol.pdf in repository),File name in repository (Use $find *Text-in-cell*),E-value,I-value,nchar,Run1,,Run2,,Run3,,Run4,,maxdiff,meandiff,Prior,alpha,gamma_rates[1],gamma_rates[2],gamma_rates[3],gamma_rates[4],mu,pi[1],pi[2],rec_mu,Prior,alpha,gamma_rates[1],gamma_rates[2],gamma_rates[3],gamma_rates[4],mu,pi[1],pi[2],rec_mu,maxd

Ortho,Opi,Opi-ortho-disAb,1,47,Opi-ortho-disAb,1.00E-03,1.5,35455,,,,,,,,,,,,,,,,,,,,,,,,,,,,,,"(ACDI:0.001338,NEVE:0.001413,(HYMA:0.003049,(((TRAD:0.001728,HOHO:0.000719)0.99:0.002705,((PLBA:0.003774,MNLE:0.001359)0.99:0.003999,(((SYCI:0.002491,OSCA:0.005023)0.87:9.1e-05,(TEWI:0.001985,AMQU:0.001501)0.99:0.001174)0.99:0.000263,((SARO:0.001999,MOBR:0.003614)0.99:0.002256,(CAOW:0.004588,(SPAR:0.005023,(SPPU:0.002

Ortho,OpiXen,OpiXen-ortho-disAb,2,41,OpiXen_ortho_disAb41,1.00E-03,1.5,26063,,,,,,,,,,,,,,,,,,,,,,,,,,,,,,"(ACDI:0.001369,NEVE:0.001557,(HYMA:0.00396,(((TRAD:0.001879,HOHO:0.000694)0.99:0.003516,((PLBA:0.004527,MNLE:0.001399)0.99:0.005616,(((SYCI:0.00275,OSCA:0.00676)0.83:0.000157,(TEWI:0.00238,AMQU:0.001672)0.99:0.001711)0.99:0.00039,((SARO:0.002229,MOBR:0.004492)0.99:0.003255,(CAOW:0.005951,(SPAR:0.006888,(S

Ortho,OpiAco,OpiAco-ortho-disAb,3,44,OpiAco_ortho_disAb44,1.00E-03,1.5,32774,,,,,,,,,,,,,,,,,,,,,,,,,,,,,,"(ACDI:0.001645,NEVE:0.001757,(HYMA:0.00402,(((TRAD:0.002107,HOHO:0.000792)0.99:0.00368,((PLBA:0.004835,MNLE:0.001601)0.99:0.005494,(((SYCI:0.003101,OSCA:0.006833)0.97:0.000153,(TEWI:0.002534,AMQU:0.001872)0.99:0.001632)0.99:0.000365,((SARO:0.002465,MOBR:0.004738)0.99:0.003135,(CAOW:0.006131,(SPAR:0.00683

Ortho,Opi,Opi-ortho-neAb,4,44,Opi-ortho-neAb,1.00E-03,1.5,34022,,,,,,,,,,,,,,,,,,,,,,,,,,,,,,"(ACDI:0.001557,NEVE:0.001639,(HYMA:0.003582,(((TRAD:0.001978,HOHO:0.000847)0.99:0.003176,((PLBA:0.004347,MNLE:0.001501)0.99:0.004847,(((SYCI:0.002912,OSCA:0.005957)0.87:0.000105,(TEWI:0.002299,AMQU:0.001739)0.99:0.001395)0.99:0.000303,((SARO:0.002314,MOBR:0.004237)0.99:0.002636,(CAOW:0.005327,(SPAR:0.005811,(SPPU:0.00

Ortho,OpiXen,OpiXen-ortho-neAb,5,38,OpiXen-ortho-neAb,1.00E-03,1.5,24683,,,,,,,,,,,,,,,,,,,,,,,,,,,,,,"(ACDI:0.001573,NEVE:0.001751,(HYMA:0.00447,(((TRAD:0.002172,HOHO:0.000766)0.99:0.003957,((PLBA:0.00522,MNLE:0.001663)0.99:0.006291,((OSCA:0.007674,(SYCI:0.00315,(TEWI:0.00268,AMQU:0.001866)0.99:0.001912)0.76:0.000174)0.99:0.000404,((SARO:0.002547,MOBR:0.005125)0.99:0.003658,(CAOW:0.006784,(SPAR:0.007743,(SPI

Ortho,OpiAco,OpiAco-ortho-neAb,6,41,OpiAco_ortho_neAb,1.00E-03,1.5,31361,,,,,,,,,,,,,,,,,,,,,,,,,,,,,,"(ACDI:0.001975,NEVE:0.002014,(HYMA:0.004868,(((TRAD:0.002455,HOHO:0.000957)0.99:0.004414,((PLBA:0.005741,MNLE:0.001864)0.99:0.006603,(((SYCI:0.00372,OSCA:0.008216)0.96:0.000162,(TEWI:0.00303,AMQU:0.002244)0.99:0.001982)0.99:0.000432,((SARO:0.002927,MOBR:0.005647)0.99:0.00375,(CAOW:0.007296,(SPAR:0.00812,(SP

Ortho,Hol,Hol-ortho-disAb,7,41,Hol_ortho_disAb,1.00E-03,1.5,34495,,,,,,,,,,,,,,,,,,,,,,,,,,,,,,"(ACDI:0.002407,NEVE:0.002625,(HYMA:0.006483,(((TRAD:0.003137,HOHO:0.00127)0.99:0.005279,((PLBA:0.007811,MNLE:0.002443)0.99:0.008546,(((SARO:0.003595,MOBR:0.007101)0.99:0.004481,(SPAR:0.011252,CAOW:0.008035)0.99:0.002247)0.99:0.002167,(OSCA:0.010732,(SYCI:0.004898,(TEWI:0.004001,AMQU:0.002975)0.99:0.002407)0.92:0.0002

Ortho,HolXen,HolXen-ortho-disAb,8,35,HolXen_ortho_disAb,1.00E-03,1.5,25104,,,,,,,,,,,,,,,,,,,,,,,,,,,,,,"(ACDI:0.002108,NEVE:0.002365,(HYMA:0.006154,(((TRAD:0.002851,HOHO:0.00103)0.99:0.00535,((PLBA:0.007359,MNLE:0.002209)0.99:0.008642,(((SARO:0.003381,MOBR:0.006863)0.99:0.0048,(SPAR:0.011503,CAOW:0.008251)0.99:0.002368)0.99:0.002297,(OSCA:0.01067,(SYCI:0.004344,(TEWI:0.003694,AMQU:0.00259)0.99:0.002553)0.94:0

Ortho,HolAco,HolAco-ortho-disAb,9,38,HolAco_ortho_disAb,1.00E-03,1.5,31885,,,,,,,,,,,,,,,,,,,,,,,,,,,,,,"(ACDI:0.002675,NEVE:0.002779,(HYMA:0.006958,(((TRAD:0.00342,HOHO:0.001351)0.99:0.006092,((PLBA:0.008804,MNLE:0.002729)0.99:0.009487,(((SARO:0.003997,MOBR:0.007838)0.99:0.005249,(SPAR:0.012746,CAOW:0.009305)0.99:0.002518)0.99:0.002422,(OSCA:0.012035,(SYCI:0.005311,(TEWI:0.004341,AMQU:0.003201)0.99:0.002781)

Ortho,Hol,Hol-ortho-neAb,10,38,Hol_ortho_neAb,1.00E-03,1.5,33072,,,,,,,,,,,,,,,,,,,,,,,,,,,,,,"(ACDI:0.003286,NEVE:0.003626,(HYMA:0.009405,(((TRAD:0.004159,HOHO:0.001665)0.99:0.007559,((PLBA:0.011137,MNLE:0.003318)0.99:0.012399,(((SARO:0.004758,MOBR:0.009984)0.99:0.006465,(SPAR:0.016193,CAOW:0.011127)0.99:0.003399)0.99:0.003208,(OSCA:0.015771,(SYCI:0.006861,(TEWI:0.005593,AMQU:0.004137)0.99:0.003536)0.95:0.000

Ortho,HolXen,HolXen-ortho-neAb,11,32,HolXen_ortho-neAb,1.00E-03,1.5,23716,,,,,,,,,,,,,,,,,,,,,,,,,,,,,,"(ACDI:0.002628,NEVE:0.002885,(HYMA:0.007839,(((TRAD:0.003557,HOHO:0.001293)0.99:0.006862,((PLBA:0.009428,MNLE:0.002644)0.99:0.011051,(((SARO:0.004226,MOBR:0.008792)0.99:0.00614,(SPAR:0.014663,CAOW:0.01029)0.99:0.003107)0.99:0.003,(OSCA:0.013744,(SYCI:0.005488,(TEWI:0.004723,AMQU:0.003288)0.99:0.003235)0.99:

Ortho,HolAco,HolAco-ortho-neAb,12,35,HolAco_ortho_neAb,1.00E-03,1.5,30435,,,,,,,,,,,,,,,,,,,,,,,,,,,,,,"(ACDI:0.003552,NEVE:0.003602,(HYMA:0.009553,(((TRAD:0.004377,HOHO:0.00176)0.99:0.008268,((PLBA:0.011852,MNLE:0.003449)0.99:0.013042,(((SARO:0.005237,MOBR:0.010559)0.99:0.007105,(SPAR:0.017423,CAOW:0.012369)0.99:0.003556)0.99:0.003345,(OSCA:0.016589,(SYCI:0.007121,(TEWI:0.00582,AMQU:0.004252)0.99:0.003817)0

Ortho,Cho,Cho-ortho-disAb,13,39,Cho-ortho-disAb,1.00E-03,1.5,34448,,,,,,,,,,,,,,,,,,,,,,,,,,,,,,"(ACDI:0.002667,NEVE:0.002961,(HYMA:0.007383,(((TRAD:0.003566,HOHO:0.001432)0.99:0.005978,((PLBA:0.009006,MNLE:0.00277)0.99:0.009885,((SARO:0.003988,MOBR:0.008372)0.99:0.007918,(OSCA:0.012408,(SYCI:0.005527,(TEWI:0.004547,AMQU:0.003374)0.99:0.002749)0.98:0.000244)0.99:0.000326)0.99:0.000607)0.99:0.00015)0.99:0.000249,(

Ortho,ChoXen,ChoXen-ortho-disAb,14,33,ChoXen_ortho_disAb,1.00E-03,1.5,25050,,,,,,,,,,,,,,,,,,,,,,,,,,,,,,"(ACDI:0.002358,NEVE:0.002569,(HYMA:0.006903,(((TRAD:0.003178,HOHO:0.001144)0.99:0.005983,((PLBA:0.008727,MNLE:0.002562)0.99:0.009737,((SARO:0.00358,MOBR:0.007749)0.99:0.008359,(OSCA:0.011992,(SYCI:0.004833,(TEWI:0.00418,AMQU:0.002879)0.99:0.002857)0.99:0.000309)0.99:0.000313)0.99:0.000666)0.69:9.6e-05)0.99:

Ortho,ChoAco,ChoAco-ortho-disAb,15,36,ChoAco_ortho_disAb,1.00E-03,1.5,31786,,,,,,,,,,,,,,,,,,,,,,,,,,,,,,"(ACDI:0.003027,NEVE:0.003161,(HYMA:0.008076,(((TRAD:0.003916,HOHO:0.001528)0.99:0.006952,((PLBA:0.010198,MNLE:0.003004)0.99:0.011026,((SARO:0.004372,MOBR:0.009276)0.99:0.00918,(OSCA:0.013988,(SYCI:0.006004,(TEWI:0.004988,AMQU:0.003667)0.99:0.00315)0.93:0.000267)0.99:0.000368)0.99:0.000668)0.98:0.000146)0.9

Ortho,Cho,Cho-ortho-neAb,16,36,Cho_ortho_neAb,1.00E-03,1.5,33051,,,,,,,,,,,,,,,,,,,,,,,,,,,,,,"(ACDI:0.003878,NEVE:0.004297,(HYMA:0.011416,(((TRAD:0.004867,HOHO:0.001936)0.99:0.009118,((PLBA:0.013701,MNLE:0.003896)0.99:0.015217,((SARO:0.005571,MOBR:0.012234)0.99:0.012227,(OSCA:0.019374,(SYCI:0.008172,(TEWI:0.006765,AMQU:0.004941)0.99:0.004282)0.98:0.000346)0.99:0.000491)0.99:0.000886)0.99:0.000268)0.99:0.0004

Ortho,ChoXen,ChoXen-ortho-neAb,17,30,ChoXen_ortho_neAb,1.00E-03,1.5,23703,,,,,,,,,,,,,,,,,,,,,,,,,,,,,,"(ACDI:0.003081,NEVE:0.003371,(HYMA:0.009249,(((TRAD:0.004171,HOHO:0.001518)0.99:0.008115,((PLBA:0.01146,MNLE:0.003125)0.99:0.013327,((SARO:0.004783,MOBR:0.010503)0.99:0.011211,(OSCA:0.016477,(SYCI:0.00643,(TEWI:0.005621,AMQU:0.003796)0.99:0.003822)0.99:0.000381)0.99:0.000423)0.99:0.000802)0.98:0.000189)0.99

Ortho,ChoAco,ChoAco-ortho-neAb,18,33,ChoAco_ortho_neAb,1.00E-03,1.5,30411,,,,,,,,,,,,,,,,,,,,,,,,,,,,,,"(ACDI:0.004222,NEVE:0.004284,(HYMA:0.011684,(((TRAD:0.005179,HOHO:0.002056)0.99:0.009933,((PLBA:0.014683,MNLE:0.004165)0.99:0.016063,((SARO:0.006079,MOBR:0.013082)0.99:0.013312,(OSCA:0.020409,(SYCI:0.008523,(TEWI:0.007124,AMQU:0.00516)0.99:0.004654)0.99:0.000377)0.99:0.000536)0.99:0.000914)0.95:0.000194)0.9

Ortho,OpiXen,OpiXen-ortho-disP,19,41,OpiXen_ortho_disP,1.00E-03,1.5,31067,,,,,,,,,,,,,,,,,,,,,,,,,,,,,,"(ACDI:0.001325,NEVE:0.001496,(HYMA:0.003881,(((TRAD:0.001762,HOHO:0.000703)0.99:0.003467,((PLBA:0.004502,MNLE:0.001353)0.99:0.005539,(((SYCI:0.002753,OSCA:0.006424)0.99:0.000162,(TEWI:0.002289,AMQU:0.001672)0.99:0.001602)0.99:0.000392,((SARO:0.00223,MOBR:0.004434)0.99:0.003063,(CAOW:0.005701,(SPAR:0.006687,(

Ortho,OpiAco,OpiAco-ortho-disP,20,44,OpiAco-ortho-disP,1.00E-03,1.5,32886,,,,,,,,,,,,,,,,,,,,,,,,,,,,,,"(ACDI:0.001648,NEVE:0.001699,(HYMA:0.004086,(((TRAD:0.002062,HOHO:0.000845)0.99:0.003701,((PLBA:0.004983,MNLE:0.001624)0.99:0.005743,(((SYCI:0.003145,OSCA:0.006783)0.97:0.000149,(TEWI:0.002584,AMQU:0.001903)0.99:0.001693)0.99:0.000385,((SARO:0.002492,MOBR:0.004734)0.99:0.003142,(CAOW:0.006109,(SPAR:0.00699,(S

Ortho,Opi,Opi-ortho-neP,21,44,Opi-ortho-neP,1.00E-03,1.5,35239,,,,,,,,,,,,,,,,,,,,,,,,,,,,,,"(ACDI:0.001616,NEVE:0.001695,(HYMA:0.003743,(((TRAD:0.00205,HOHO:0.000863)0.99:0.0033,((PLBA:0.004473,MNLE:0.001539)0.99:0.004986,(((SYCI:0.002996,OSCA:0.006192)0.72:0.000106,(TEWI:0.002397,AMQU:0.001785)0.99:0.001434)0.99:0.000313,((SARO:0.002378,MOBR:0.004352)0.99:0.002719,(CAOW:0.005465,(SPAR:0.006005,(SPPU:0.002985

Ortho,OpiXen,OpiXen-ortho-neP,22,38,OpiXen-ortho-neP,1.00E-03,1.5,30911,,,,,,,,,,,,,,,,,,,,,,,,,,,,,,"(ACDI:0.001509,NEVE:0.001738,(HYMA:0.004485,(((TRAD:0.002105,HOHO:0.000817)0.99:0.004016,((PLBA:0.005199,MNLE:0.001501)0.99:0.006604,(((SYCI:0.003185,OSCA:0.007773)0.97:0.000186,(TEWI:0.002614,AMQU:0.001888)0.99:0.001891)0.99:0.000459,((SARO:0.002521,MOBR:0.005154)0.99:0.003638,(CAOW:0.006734,(SPAR:0.00788,(S

Ortho,OpiAco,OpiAco-ortho-neP,23,41,OpiAco-ortho-neP,1.00E-03,1.5,32684,,,,,,,,,,,,,,,,,,,,,,,,,,,,,,"(ACDI:0.001896,NEVE:0.001984,(HYMA:0.004825,(((TRAD:0.002386,HOHO:0.000959)0.99:0.004363,((PLBA:0.005814,MNLE:0.001827)0.99:0.006811,(((SYCI:0.003708,OSCA:0.008121)0.91:0.000166,(TEWI:0.003014,AMQU:0.002241)0.99:0.001972)0.99:0.000454,((SARO:0.002919,MOBR:0.005627)0.99:0.003724,(CAOW:0.007172,(SPAR:0.00825,(SP

Ortho,Hol,Hol-ortho-disP,24,41,Hol-ortho-disP,1.00E-03,1.5,35030,,,,,,,,,,,,,,,,,,,,,,,,,,,,,,"(ACDI:0.002375,NEVE:0.002608,(HYMA:0.006334,(((TRAD:0.003124,HOHO:0.001248)0.99:0.005189,((PLBA:0.007587,MNLE:0.002444)0.99:0.008293,(((SARO:0.003635,MOBR:0.007018)0.99:0.004341,(SPAR:0.010937,CAOW:0.007857)0.99:0.002404)0.99:0.002201,((SYCI:0.004751,OSCA:0.01037)0.64:0.000185,(TEWI:0.003876,AMQU:0.002916)0.99:0.002381

Ortho,HolXen,HolXen-ortho-disP,25,35,HolXen-ortho-disP,1.00E-03,1.5,30717,,,,,,,,,,,,,,,,,,,,,,,,,,,,,,"(ACDI:0.001978,NEVE:0.002218,(HYMA:0.005957,(((TRAD:0.002681,HOHO:0.001092)0.99:0.005157,((PLBA:0.007131,MNLE:0.002101)0.99:0.008518,(((SARO:0.003297,MOBR:0.006768)0.99:0.004523,(SPAR:0.011105,CAOW:0.007606)0.99:0.002587)0.99:0.00225,((SYCI:0.004182,OSCA:0.010149)0.98:0.000228,(TEWI:0.003492,AMQU:0.002548)0.9

Ortho,HolAco,HolAco-ortho-disP,26,38,HolAco-ortho-disP,1.00E-03,1.5,32469,,,,,,,,,,,,,,,,,,,,,,,,,,,,,,"(ACDI:0.00245,NEVE:0.002579,(HYMA:0.006476,(((TRAD:0.003121,HOHO:0.001253)0.99:0.005624,((PLBA:0.007965,MNLE:0.0025)0.99:0.009094,(((SARO:0.003941,MOBR:0.007488)0.99:0.004771,(SPAR:0.012086,CAOW:0.008558)0.99:0.002539)0.99:0.002271,((SYCI:0.004946,OSCA:0.01106)0.94:0.000213,(TEWI:0.004019,AMQU:0.003008)0.99:0.

Ortho,Hol,Hol-ortho-neP38,27,38,Hol-ortho-neP38,1.00E-03,1.5,34821,,,,,,,,,,,,,,,,,,,,,,,,,,,,,,"(ACDI:0.003222,NEVE:0.003557,(HYMA:0.009109,(((TRAD:0.004152,HOHO:0.00165)0.99:0.007337,((PLBA:0.010748,MNLE:0.003263)0.99:0.011955,(((SARO:0.004856,MOBR:0.009736)0.99:0.006134,(SPAR:0.015599,CAOW:0.010771)0.99:0.00356)0.99:0.003196,(OSCA:0.015169,(SYCI:0.006635,(TEWI:0.005441,AMQU:0.004036)0.99:0.003425)0.71:0.0002

Ortho,HolXen,HolXen-ortho-neP,28,32,HolXen-ortho-neP,1.00E-03,1.5,30572,,,,,,,,,,,,,,,,,,,,,,,,,,,,,,"(ACDI:0.002505,NEVE:0.002798,(HYMA:0.007689,(((TRAD:0.003335,HOHO:0.001319)0.99:0.006663,((PLBA:0.009186,MNLE:0.002576)0.99:0.011116,(((SARO:0.004196,MOBR:0.008636)0.99:0.005835,(SPAR:0.014475,CAOW:0.009705)0.99:0.003349)0.99:0.002909,((SYCI:0.005307,OSCA:0.013238)0.95:0.000281,(TEWI:0.004457,AMQU:0.003164)0.9

Ortho,HolAco,HolAco-ortho-neP,29,35,HolAco-ortho-neP,1.00E-03,1.5,32276,,,,,,,,,,,,,,,,,,,,,,,,,,,,,,"(ACDI:0.0032,NEVE:0.003353,(HYMA:0.008699,(((TRAD:0.003984,HOHO:0.00163)0.99:0.007457,((PLBA:0.010619,MNLE:0.003196)0.99:0.012136,(((SARO:0.004874,MOBR:0.009823)0.99:0.006384,(SPAR:0.016029,CAOW:0.011079)0.99:0.003509)0.99:0.003102,((SYCI:0.006534,OSCA:0.014894)0.67:0.000252,(TEWI:0.005328,AMQU:0.003906)0.99:0

Ortho,Cho,Cho-ortho-disP,30,39,Cho-ortho-disP39,1.00E-03,1.5,34449,,,,,,,,,,,,,,,,,,,,,,,,,,,,,,"(ACDI:0.002694,NEVE:0.002961,(HYMA:0.007398,(((TRAD:0.003523,HOHO:0.001404)0.99:0.006001,((PLBA:0.009072,MNLE:0.002822)0.99:0.009844,((SARO:0.004039,MOBR:0.008407)0.99:0.007948,(OSCA:0.012475,(SYCI:0.005525,(TEWI:0.004568,AMQU:0.003376)0.99:0.002754)0.98:0.000244)0.99:0.000328)0.99:0.000612)0.98:0.00015)0.99:0.00025,((

Ortho,ChoXen,ChoXen-ortho-disP,31,33,ChoXen-ortho-disP,1.00E-03,1.5,30751,,,,,,,,,,,,,,,,,,,,,,,,,,,,,,"(ACDI:0.002215,NEVE:0.002467,(HYMA:0.006596,(((TRAD:0.003003,HOHO:0.001134)0.99:0.005692,((PLBA:0.007979,MNLE:0.002324)0.99:0.009606,((SARO:0.003483,MOBR:0.007568)0.99:0.007788,((SYCI:0.004605,OSCA:0.011392)0.98:0.000271,(TEWI:0.003885,AMQU:0.002831)0.99:0.002718)0.99:0.000392)0.99:0.000616)0.99:0.000148)0.99:

Ortho,ChoAco,ChoAco-ortho-disP,32,36,ChoAco-ortho-disP,1.00E-03,1.5,32400,,,,,,,,,,,,,,,,,,,,,,,,,,,,,,"(ACDI:0.002749,NEVE:0.002889,(HYMA:0.007307,(((TRAD:0.003482,HOHO:0.001404)0.99:0.00625,((PLBA:0.009147,MNLE:0.002864)0.99:0.010219,((SARO:0.00418,MOBR:0.00855)0.99:0.008189,((SYCI:0.005508,OSCA:0.012462)0.83:0.000229,(TEWI:0.004594,AMQU:0.003371)0.99:0.00291)0.99:0.000381)0.99:0.00062)0.97:0.000137)0.99:0.0002

Ortho,Cho,Cho-ortho-neP,33,36,Cho-ortho-neP,1.00E-03,1.5,34738,,,,,,,,,,,,,,,,,,,,,,,,,,,,,,"(ACDI:0.003774,NEVE:0.004184,(HYMA:0.010979,(((TRAD:0.004844,HOHO:0.001908)0.99:0.008702,((PLBA:0.012997,MNLE:0.003816)0.99:0.014518,((SARO:0.005626,MOBR:0.011934)0.99:0.01166,(OSCA:0.0185,(SYCI:0.007896,(TEWI:0.006493,AMQU:0.004779)0.99:0.004076)0.89:0.000324)0.99:0.000466)0.99:0.000895)0.99:0.000242)0.99:0.000407,((((IXS

Ortho,ChoXen,ChoXen-ortho-neP,34,30,ChoXen-ortho-neP,1.00E-03,1.5,30621,,,,,,,,,,,,,,,,,,,,,,,,,,,,,,"(ACDI:0.002867,NEVE:0.003257,(HYMA:0.008882,(((TRAD:0.003843,HOHO:0.001451)0.99:0.007642,((PLBA:0.01083,MNLE:0.003059)0.99:0.012929,((SARO:0.004593,MOBR:0.010128)0.99:0.010517,((SYCI:0.006138,OSCA:0.015636)0.9:0.000328,(TEWI:0.00516,AMQU:0.003637)0.99:0.003671)0.99:0.000515)0.99:0.000825)0.99:0.000211)0.99:0.00

Ortho,ChoAco,ChoAco-ortho-neP,35,33,ChoAco-ortho-neP33,1.00E-03,1.5,32213,,,,,,,,,,,,,,,,,,,,,,,,,,,,,,"(ACDI:0.00375,NEVE:0.003903,(HYMA:0.010414,(((TRAD:0.004656,HOHO:0.001863)0.99:0.008732,((PLBA:0.01293,MNLE:0.003749)0.99:0.014547,((SARO:0.005651,MOBR:0.011936)0.99:0.011635,(OSCA:0.018024,(SYCI:0.007731,(TEWI:0.006313,AMQU:0.00466)0.99:0.004093)0.67:0.000302)0.99:0.000498)0.99:0.000876)0.98:0.000198)0.99:0.

Homo,Opi,Opi-homo-disAb,36,47,Opi-homo-disAb47,1.00E-03,1.5,32767,,,,,,,,,,,,,,,,,,,,,,,,,,,,,,"(ACDI:0.001593,NEVE:0.001639,(HYMA:0.004437,(((PLBA:0.004922,MNLE:0.001245)0.99:0.007328,((TRAD:0.001582,HOHO:0.000635)0.99:0.004263,((OSCA:0.007065,(SYCI:0.00345,(TEWI:0.002814,AMQU:0.001947)0.99:0.001918)0.99:0.0002)0.99:0.000321,((SPAR:0.008489,CAOW:0.005935)0.99:0.001642,((SARO:0.002818,MOBR:0.005072)0.99:0.00

Homo,OpiXen,OpiXen-homo-disAb,37,41,OpiXen-homo-disAb41,1.00E-03,1.5,26554,,,,,,,,,,,,,,,,,,,,,,,,,,,,,,"(ACDI:0.001684,NEVE:0.001688,(HYMA:0.004704,(((PLBA:0.005413,MNLE:0.001427)0.99:0.007765,((TRAD:0.001701,HOHO:0.000674)0.99:0.004667,((OSCA:0.007713,(SYCI:0.003664,(TEWI:0.003003,AMQU:0.002091)0.99:0.002025)0.85:0.000193)0.99:0.000361,((SPAR:0.009623,CAOW:0.006743)0.99:0.001736,((SARO:0.003172,MOBR:0.0

Homo,OpiAco,OpiAco-homo-disAb,38,44,OpiAco.BLAST_Results_dis.1e3_1.5.mcl.out.fasta.nexus,1.00E-03,1.5,31268,,,,,,,,,,,,,,,,,,,,,,,,,,,,,,"(ACDI:0.001498,NEVE:0.001516,(HYMA:0.004174,(((PLBA:0.004714,MNLE:0.001181)0.99:0.006913,((TRAD:0.001521,HOHO:0.000623)0.99:0.004067,((OSCA:0.006699,(SYCI:0.003229,(TEWI:0.002655,AMQU:0.001849)0.99:0.001811)0.7:0.000164)0.99:0.000331,((SPAR:0.008407,CAOW:0.005916)0.99:0.0015

Homo,Opi,Opi-homo-neAb,39,44,Opi-homo-neAb44,1.00E-03,1.5,31089,,,,,,,,,,,,,,,,,,,,,,,,,,,,,,"(ACDI:0.00177,NEVE:0.001824,(HYMA:0.004975,(((TRAD:0.001762,HOHO:0.000695)0.99:0.004869,((PLBA:0.005673,MNLE:0.001498)0.99:0.008139,((OSCA:0.008052,(SYCI:0.003898,(TEWI:0.003145,AMQU:0.002205)0.99:0.002126)0.99:0.000221)0.99:0.000352,((SPAR:0.009534,CAOW:0.006664)0.99:0.001849,((SARO:0.003122,MOBR:0.005623)0.99:0.0

Homo,OpiXen,Opi-homo-neAb,40,38,OpiXen-homo-neAb38,1.00E-03,1.5,24878,,,,,,,,,,,,,,,,,,,,,,,,,,,,,,"(ACDI:0.001687,NEVE:0.001705,(HYMA:0.004726,(((PLBA:0.005485,MNLE:0.001366)0.99:0.007734,((TRAD:0.001776,HOHO:0.000704)0.99:0.004598,((OSCA:0.007632,(SYCI:0.003639,(TEWI:0.00297,AMQU:0.002075)0.99:0.002024)0.84:0.000192)0.99:0.000361,((SPAR:0.009515,CAOW:0.006662)0.99:0.001713,((SARO:0.003166,MOBR:0.005658

Homo,OpiAco,OpiAco-homo-neAb,41,41,OpiAco-homo-neAb41,1.00E-03,1.5,29622,,,,,,,,,,,,,,,,,,,,,,,,,,,,,,"(ACDI:0.001507,NEVE:0.001572,(HYMA:0.004468,(((TRAD:0.001581,HOHO:0.000581)0.99:0.004451,((PLBA:0.005046,MNLE:0.001233)0.99:0.007425,((OSCA:0.007389,(SYCI:0.003467,(TEWI:0.002767,AMQU:0.001855)0.99:0.001877)0.99:0.000234)0.99:0.000312,((SPAR:0.009405,CAOW:0.006361)0.99:0.001795,((SARO:0.002879,MOBR:0.00

Homo,Hol,Hol-homo-disAb,42,41,Hol-homo-disAb41,1.00E-03,1.5,30818,,,,,,,,,,,,,,,,,,,,,,,,,,,,,,"(ACDI:0.002248,NEVE:0.002295,(HYMA:0.006474,(((PLBA:0.007548,MNLE:0.001833)0.99:0.010769,((TRAD:0.002264,HOHO:0.000852)0.99:0.006187,(((SARO:0.004055,MOBR:0.007378)0.99:0.00528,(SPAR:0.012506,CAOW:0.008276)0.99:0.002472)0.99:0.002332,(OSCA:0.010434,(SYCI:0.004997,(TEWI:0.004025,AMQU:0.002801)0.99:0.002724)0.99:0.0

Homo,HolXen,HolXen-homo-disAb,43,35,HolXen-homo-disAb,1.00E-03,1.5,24614,,,,,,,,,,,,,,,,,,,,,,,,,,,,,,"(ACDI:0.002674,NEVE:0.002601,(HYMA:0.007638,(((PLBA:0.009077,MNLE:0.00211)0.99:0.012942,((TRAD:0.002762,HOHO:0.001074)0.99:0.007349,(((SARO:0.004907,MOBR:0.009148)0.99:0.006496,(SPAR:0.015651,CAOW:0.010292)0.99:0.002925)0.99:0.00271,(OSCA:0.012657,(SYCI:0.005935,(TEWI:0.004813,AMQU:0.003325)0.99:0.003215

Homo,HolAco,HolAco-homo-disAb,44,38,HolAco-homo-SCMA_is_in_disAb,1.00E-03,1.5,29318,,,,,,,,,,,,,,,,,,,,,,,,,,,,,,"(ACDI:0.002207,NEVE:0.002232,(HYMA:0.006275,(((PLBA:0.007504,MNLE:0.001874)0.99:0.010445,((TRAD:0.002218,HOHO:0.000853)0.99:0.006021,(((SARO:0.00401,MOBR:0.007414)0.99:0.005334,(SPAR:0.012696,CAOW:0.00844)0.99:0.002393)0.99:0.00217,(OSCA:0.010294,(SYCI:0.004882,(TEWI:0.003943,AMQU:0.002789)0.9

Homo,Hol,Hol-homo-neAb,45,38,Hol-homo-neAb38-cluster.Hol-homo-neAb.BLAST_Results.1e3_1.5.mcl,1.00E-03,1.5,29135,,,,,,,,,,,,,,,,,,,,,,,,,,,,,,"(ACDI:0.002664,NEVE:0.002693,(HYMA:0.007862,(((PLBA:0.009028,MNLE:0.002087)0.99:0.013133,((TRAD:0.002642,HOHO:0.001066)0.99:0.007402,(((SARO:0.004786,MOBR:0.00884)0.99:0.00635,(SPAR:0.015001,CAOW:0.00992)0.99:0.003053)0.99:0.002871,(OSCA:0.012676,(SYCI:0.006006,(TEWI:

Homo,HolXen,HolXen-homo-neAb,46,32,HolXen.BLAST_Results_ne.1e3_1.5.mcl.out,1.00E-03,1.5,22970,,,,,,,,,,,,,,,,,,,,,,,,,,,,,,"(ACDI:0.00242,NEVE:0.002551,(HYMA:0.007653,(((PLBA:0.009005,MNLE:0.002025)0.99:0.013198,((TRAD:0.002639,HOHO:0.00096)0.99:0.007409,(((SARO:0.004775,MOBR:0.009173)0.99:0.006793,(SPAR:0.016293,CAOW:0.010485)0.99:0.003138)0.99:0.002969,(OSCA:0.012775,(SYCI:0.005822,(TEWI:0.00468,AMQU:0.

Homo,HolAco,HolAco-homo-neAb,47,35,cluster.HolXen-homo-neAb.BLAST_Results.1e3_1.5.mcl.table,1.00E-03,1.5,27684,,,,,,,,,,,,,,,,,,,,,,,,,,,,,,"(ACDI:0.002596,NEVE:0.002577,(HYMA:0.007555,(((PLBA:0.009015,MNLE:0.002124)0.99:0.012696,((TRAD:0.002669,HOHO:0.001016)0.99:0.007225,(((SARO:0.004838,MOBR:0.009018)0.99:0.006434,(SPAR:0.015499,CAOW:0.010222)0.99:0.002871)0.99:0.002646,(OSCA:0.012493,(SYCI:0.005858,(TEW

Homo,Cho,Cho-homo-disAb,48,39,Cho-homo-disAb,1.00E-03,1.5,34449,,,,,,,,,,,,,,,,,,,,,,,,,,,,,,"(ACDI:0.002535,NEVE:0.002562,(HYMA:0.007288,(((PLBA:0.008605,MNLE:0.002064)0.99:0.012349,((TRAD:0.002566,HOHO:0.00103)0.99:0.006885,((SARO:0.004532,MOBR:0.008499)0.99:0.009035,(OSCA:0.011935,(SYCI:0.00562,(TEWI:0.004566,AMQU:0.003187)0.99:0.003082)0.99:0.000323)0.99:0.000285)0.99:0.000604)0.99:0.000159)0.99:0.000313

Homo,ChoXen,ChoXen-homo-disAb,49,33,cluster.Aco.BLAST_Results.1e3_1.5,1.00E-03,1.5,24302,,,,,,,,,,,,,,,,,,,,,,,,,,,,,,"(ACDI:0.003084,NEVE:0.003014,(HYMA:0.00905,(((PLBA:0.010914,MNLE:0.002494)0.99:0.015389,((TRAD:0.003129,HOHO:0.001176)0.99:0.00865,((SARO:0.005741,MOBR:0.010981)0.99:0.011188,(OSCA:0.015169,(SYCI:0.006996,(TEWI:0.005694,AMQU:0.003918)0.99:0.003808)0.99:0.00036)0.99:0.000345)0.99:0.000721)0.96

Homo,ChoAco,ChoAco-homo-disAb,50,36,cluster.Xen.BLAST_Results.1e3_1.5.mcl.table.fasta.nexus,1.00E-03,1.5,29017,,,,,,,,,,,,,,,,,,,,,,,,,,,,,,"(ACDI:0.002459,NEVE:0.00244,(HYMA:0.007168,(((PLBA:0.008864,MNLE:0.00226)0.99:0.011897,((TRAD:0.002493,HOHO:0.000948)0.99:0.006783,((SARO:0.004736,MOBR:0.008728)0.99:0.00868,(OSCA:0.011752,(SYCI:0.005546,(TEWI:0.004491,AMQU:0.003138)0.99:0.00301)0.98:0.000277)0.99:0.00029

Homo,Cho,Cho-homo-neAb,51,36,cluster.Cho-homo-neAb.BLAST_Results.1e3_1.5,1.00E-03,1.5,28828,,,,,,,,,,,,,,,,,,,,,,,,,,,,,,"(ACDI:0.003118,NEVE:0.003144,(HYMA:0.009141,(((PLBA:0.010852,MNLE:0.002491)0.99:0.015514,((TRAD:0.003105,HOHO:0.001198)0.99:0.008634,((SARO:0.005648,MOBR:0.010704)0.99:0.011222,(OSCA:0.015165,(SYCI:0.007031,(TEWI:0.005715,AMQU:0.003884)0.99:0.00384)0.99:0.000423)0.99:0.00034)0.99:0.00076

Homo,ChoXen,ChoXen-homo-neAb,52,30,ChoXen-homo-disAb33,1.00E-03,1.5,22662,,,,,,,,,,,,,,,,,,,,,,,,,,,,,,"(ACDI:0.003099,NEVE:0.003017,(HYMA:0.00905,(((PLBA:0.01087,MNLE:0.002444)0.99:0.015402,((TRAD:0.003157,HOHO:0.001219)0.99:0.008615,((SARO:0.005782,MOBR:0.011058)0.99:0.011186,(OSCA:0.015229,(SYCI:0.007006,(TEWI:0.005689,AMQU:0.00391)0.99:0.003805)0.99:0.000365)0.99:0.000348)0.99:0.00073)0.97:0.000164)0.9

Homo,ChoAco,ChoAco-homo-neAb,53,33,cluster.ChoXen-homo-neAb.BLAST_Results.1e3_1.5.mcl.table.fasta.nexus,1.00E-03,1.5,27366,,,,,,,,,,,,,,,,,,,,,,,,,,,,,,"(ACDI:0.002944,NEVE:0.00305,(HYMA:0.009298,(((PLBA:0.011182,MNLE:0.002412)0.99:0.016357,((TRAD:0.003193,HOHO:0.001132)0.99:0.009028,((SARO:0.005744,MOBR:0.011362)0.99:0.012364,(OSCA:0.015871,(SYCI:0.007105,(TEWI:0.005717,AMQU:0.003807)0.99:0.00391)0.99:0.000

Homo,OpiXen,OpiXen-homo-disP,54,41,OpiXen-homo-disP41,1.00E-03,1.5,30446,,,,,,,,,,,,,,,,,,,,,,,,,,,,,,"(ACDI:0.001384,NEVE:0.001451,(HYMA:0.004098,(((PLBA:0.004732,MNLE:0.001196)0.99:0.006976,((TRAD:0.001447,HOHO:0.00057)0.99:0.003992,((OSCA:0.006581,(SYCI:0.003155,(TEWI:0.002517,AMQU:0.001727)0.99:0.001788)0.99:0.000212)0.99:0.000314,((SPAR:0.008526,CAOW:0.005813)0.99:0.001616,((SARO:0.002685,MOBR:0.00494

Homo,OpiAco,OpiAco-homo-disP,55,44,OpiAco-homo-disP44-slurm,1.00E-03,1.5,31532,,,,,,,,,,,,,,,,,,,,,,,,,,,,,,"(ACDI:0.001549,NEVE:0.001575,(HYMA:0.004314,(((PLBA:0.004991,MNLE:0.001315)0.99:0.007178,((TRAD:0.001558,HOHO:0.000605)0.99:0.004237,((OSCA:0.006978,(SYCI:0.00337,(TEWI:0.002753,AMQU:0.001893)0.99:0.001913)0.98:0.000196)0.99:0.000332,((SPAR:0.008714,CAOW:0.006077)0.99:0.001592,((SARO:0.002852,MOBR:0.

Homo,Opi,Opi-homo-neP,56,44,Opi-homo-neP44,1.00E-03,1.5,32514,,,,,,,,,,,,,,,,,,,,,,,,,,,,,,"(ACDI:0.001841,NEVE:0.001842,(HYMA:0.005015,((((PLBA:0.005628,MNLE:0.001366)0.99:0.008342,(TRAD:0.001801,HOHO:0.000765)0.99:0.004721)0.5:0.000162,((OSCA:0.008057,(SYCI:0.003891,(TEWI:0.00315,AMQU:0.002182)0.99:0.00216)0.99:0.000218)0.99:0.000368,((SPAR:0.00965,CAOW:0.006683)0.99:0.001894,((SARO:0.003165,MOBR:0.00573

Homo,OpiXen,OpiXen-homo-neP,57,38,OpiXen-homo-neP38,1.00E-03,1.5,30250,,,,,,,,,,,,,,,,,,,,,,,,,,,,,,"(ACDI:0.001516,NEVE:0.001613,(HYMA:0.00457,(((PLBA:0.005385,MNLE:0.001346)0.99:0.007779,(TRAD:0.001617,HOHO:0.000584)0.99:0.004473,((OSCA:0.007459,(SYCI:0.003526,(TEWI:0.002829,AMQU:0.001919)0.99:0.001986)0.99:0.000237)0.99:0.000352,((SPAR:0.009698,CAOW:0.00659)0.99:0.001779,((SARO:0.002954,MOBR:0.005527)

Homo,OpiAco,OpiAco-homo-neP,58,41,OpiAco-homo-neP41,1.00E-03,1.5,31291,,,,,,,,,,,,,,,,,,,,,,,,,,,,,,"(ACDI:0.001704,NEVE:0.00174,(HYMA:0.004829,(((PLBA:0.005643,MNLE:0.001365)0.99:0.008048,((TRAD:0.00174,HOHO:0.000687)0.99:0.004678,((OSCA:0.007881,(SYCI:0.003775,(TEWI:0.003095,AMQU:0.00213)0.99:0.002116)0.98:0.000208)0.99:0.000372,((SPAR:0.009952,CAOW:0.006904)0.99:0.001772,((SARO:0.003226,MOBR:0.005799)0

Homo,Hol,Hol-homo-disP,59,41,Hol-homo-disP41,1.00E-03,1.5,32356,,,,,,,,,,,,,,,,,,,,,,,,,,,,,,"(ACDI:0.002282,NEVE:0.002317,(HYMA:0.00653,(((PLBA:0.007512,MNLE:0.001769)0.99:0.010941,((TRAD:0.002265,HOHO:0.000884)0.99:0.006197,(((SARO:0.004107,MOBR:0.007514)0.99:0.005335,(SPAR:0.01246,CAOW:0.008326)0.99:0.002571)0.99:0.002375,(OSCA:0.010474,(SYCI:0.005065,(TEWI:0.004076,AMQU:0.002817)0.99:0.002781)0.99:0.0002

Homo,HolXen,HolXen-homo-disP,60,35,HolXen-homo-disP35,1.00E-03,1.5,30185,,,,,,,,,,,,,,,,,,,,,,,,,,,,,,"(ACDI:0.002034,NEVE:0.00214,(HYMA:0.006352,(((PLBA:0.007286,MNLE:0.001704)0.99:0.010804,((TRAD:0.002214,HOHO:0.000784)0.99:0.006142,(((SARO:0.003948,MOBR:0.007572)0.99:0.005568,(SPAR:0.013207,CAOW:0.008569)0.99:0.002586)0.99:0.002432,(OSCA:0.010337,(SYCI:0.004828,(TEWI:0.003901,AMQU:0.002632)0.99:0.00263

Homo,HolAco,HolAco-homo-disP,61,38,HolAco-homo-disP38,1.00E-03,1.5,31151,,,,,,,,,,,,,,,,,,,,,,,,,,,,,,"(ACDI:0.002252,NEVE:0.002269,(HYMA:0.006421,(((PLBA:0.007628,MNLE:0.001791)0.99:0.010972,((TRAD:0.002322,HOHO:0.000898)0.99:0.006223,(((SARO:0.004176,MOBR:0.00766)0.99:0.005526,(SPAR:0.013067,CAOW:0.008782)0.99:0.002504)0.99:0.002284,(OSCA:0.01054,(SYCI:0.005092,(TEWI:0.004111,AMQU:0.002821)0.99:0.002801)(

Homo,Hol,Hol-homo-neP,62,38,Hol_homo_ne38,1.00E-03,1.5,32132,,,,,,,,,,,,,,,,,,,,,,,,,,,,,,"(ACDI:0.002778,NEVE:0.002807,(HYMA:0.007987,(((PLBA:0.009354,MNLE:0.002259)0.99:0.013472,((TRAD:0.002729,HOHO:0.001051)0.99:0.007586,(((SARO:0.004901,MOBR:0.009118)0.99:0.00665,(SPAR:0.015425,CAOW:0.010171)0.99:0.003237)0.99:0.002959,(OSCA:0.013063,(SYCI:0.006214,(TEWI:0.004993,AMQU:0.003428)0.99:0.003431)0.99:0.0003

Homo,HolXen,HolXen-homo-neP,63,32,HolXen-homo-neP,1.00E-03,1.5,30021,,,,,,,,,,,,,,,,,,,,,,,,,,,,,,"(ACDI:0.002587,NEVE:0.002651,(HYMA:0.007957,(((PLBA:0.009497,MNLE:0.00214)0.99:0.014106,((TRAD:0.00273,HOHO:0.001057)0.99:0.007699,(((SARO:0.005051,MOBR:0.009645)0.99:0.007188,(SPAR:0.016912,CAOW:0.010936)0.99:0.003401)0.99:0.002982,(OSCA:0.013266,(SYCI:0.006141,(TEWI:0.004932,AMQU:0.003308)0.99:0.003433)0.9

Homo,HolAco,HolAco-homo-neP,64,32,table.64-HolAco-homo-neP,1.00E-03,1.5,30939,,,,,,,,,,,,,,,,,,,,,,,,,,,,,,"(ACDI:0.002515,NEVE:0.002586,(HYMA:0.007802,(((PLBA:0.009249,MNLE:0.00209)0.99:0.013444,((TRAD:0.002683,HOHO:0.000962)0.99:0.007608,(((SARO:0.00482,MOBR:0.009317)0.99:0.006976,(SPAR:0.016582,CAOW:0.01066)0.99:0.003221)0.99:0.003013,(OSCA:0.013,(SYCI:0.00593,(TEWI:0.00473,AMQU:0.003212)0.99:0.003236)0.9

Homo,Cho,Cho-homo-disP,65,39,Cho-homo-disP39-cluster.Opi.BLAST_Results.1e3_1.5.mcl,1.00E-03,1.5,30505,,,,,,,,,,,,,,,,,,,,,,,,,,,,,,"(ACDI:0.002486,NEVE:0.00251,(HYMA:0.007228,(((PLBA:0.00861,MNLE:0.002041)0.99:0.012208,((TRAD:0.002498,HOHO:0.000968)0.99:0.006794,((SARO:0.004559,MOBR:0.008573)0.99:0.008803,(OSCA:0.01169,(SYCI:0.005554,(TEWI:0.004543,AMQU:0.003142)0.99:0.003056)0.99:0.000323)0.99:0.000284)0.99:0

Homo,ChoXen,ChoXen-homo-disP,66,33,ChoXen-homo-disP33-table.HolXen_homo_dis33,1.00E-03,1.5,30018,,,,,,,,,,,,,,,,,,,,,,,,,,,,,,"(ACDI:0.002367,NEVE:0.002458,(HYMA:0.007239,(((PLBA:0.008841,MNLE:0.002044)0.99:0.012837,((TRAD:0.00248,HOHO:0.000909)0.99:0.006982,((SARO:0.004549,MOBR:0.008903)0.99:0.009497,(OSCA:0.012105,(SYCI:0.005573,(TEWI:0.004482,AMQU:0.00305)0.99:0.00313)0.99:0.000355)0.99:0.00033)0.99:0.00

Homo,ChoAco,ChoAco-homo-disP,67,36,ChoAco-homo-disP36,1.00E-03,1.5,30929,,,,,,,,,,,,,,,,,,,,,,,,,,,,,,"(ACDI:0.002511,NEVE:0.002531,(HYMA:0.007297,(((PLBA:0.008844,MNLE:0.00206)0.99:0.012587,((TRAD:0.002551,HOHO:0.001006)0.99:0.006961,((SARO:0.00471,MOBR:0.008939)0.99:0.009097,(OSCA:0.012031,(SYCI:0.005715,(TEWI:0.00466,AMQU:0.003206)0.99:0.003139)0.99:0.000307)0.99:0.000315)0.99:0.000596)0.94:0.000138)0.99

Homo,Cho,Cho-homo-neP,68,36,Cho-homo-neP36,1.00E-03,1.5,31935,,,,,,,,,,,,,,,,,,,,,,,,,,,,,,"(ACDI:0.003237,NEVE:0.003243,(HYMA:0.009429,(((PLBA:0.011107,MNLE:0.0025)0.99:0.016146,((TRAD:0.00317,HOHO:0.001255)0.99:0.00889,((SARO:0.005774,MOBR:0.011169)0.99:0.011715,(OSCA:0.015537,(SYCI:0.007285,(TEWI:0.0059,AMQU:0.004031)0.99:0.004025)0.99:0.000419)0.99:0.000381)0.99:0.000782)0.74:0.000156)0.99:0.000499,((XEB

Homo,ChoXen,ChoXen-homo-neP,69,30,ChoXen-homo-neP,1.00E-03,1.5,29871,,,,,,,,,,,,,,,,,,,,,,,,,,,,,,"(ACDI:0.003055,NEVE:0.003127,(HYMA:0.009486,(((PLBA:0.011448,MNLE:0.002461)0.99:0.017094,((TRAD:0.003245,HOHO:0.00122)0.99:0.009138,((SARO:0.005889,MOBR:0.011674)0.99:0.012623,(OSCA:0.016115,(SYCI:0.007287,(TEWI:0.005853,AMQU:0.003957)0.99:0.004109)0.99:0.000462)0.99:0.000405)0.99:0.000815)0.77:0.00016)0.99:0.

Homo,ChoAco,ChoAco-homo-neP,70,33,ChoAco-homo-neP,1.00E-03,1.5,30733,,,,,,,,,,,,,,,,,,,,,,,,,,,,,,"(ACDI:0.00235,NEVE:0.002452,(HYMA:0.007214,(((PLBA:0.008434,MNLE:0.001892)0.99:0.012454,((TRAD:0.002498,HOHO:0.000969)0.99:0.006919,((SARO:0.004455,MOBR:0.008686)0.99:0.009511,(OSCA:0.012024,(SYCI:0.005484,(TEWI:0.004431,AMQU:0.003101)0.99:0.002993)0.99:0.00037)0.99:0.000286)0.99:0.000606)0.86:0.000125)0.99:0.

Homo,Xen,Non,Non,41,Diamond_41sp_Xen_1e-12_Results_allVSall.txt.abc.1.5,1.00E-12,1.5,24545,out.Diamond_41sp_Xen_1e-12_Results_allVSall.txt.abc.I15_run_1.trees.tree,70001 trees,out.Diamond_41sp_Xen_1e-12_Results_allVSall.txt.abc.I15_run_2.trees.tree,70001 trees,out.Diamond_41sp_Xen_1e-12_Results_allVSall.txt.abc.I15_run_3.trees.tree,70001 trees,out.Diamond_41sp_Xen_1e-12_Results_allVSall.txt.abc.I15_run_4.trees.tree,70001

Homo,Xen,Non,Non,41,out.Diamond_41sp_Xen_1e-12_Results_allVSall.txt.abc.I20,1.00E-12,2,29332,out.Diamond_41sp_Xen_1e-12_Results_allVSall.txt.abc.I20_run_1.trees.tree,70001 trees,out.Diamond_41sp_Xen_1e-12_Results_allVSall.txt.abc.I20_run_2.trees.tree,70001 trees,out.Diamond_41sp_Xen_1e-12_Results_allVSall.txt.abc.I20_run_3.trees.tree,70001 trees,out.Diamond_41sp_Xen_1e-12_Results_allVSall.txt.abc.I20_run_4.trees.tree,7000

Homo,Xen,Non,Non,41,out.Diamond_41sp_Xen_1e-12_Results_allVSall.txt.abc.I25,1.00E-12,2.5,32109,out.Diamond_41sp_Xen_1e-12_Results_allVSall.txt.abc.I25_run_1.trees.tree,70001 trees,out.Diamond_41sp_Xen_1e-12_Results_allVSall.txt.abc.I25_run_2.trees.tree,70001 trees,out.Diamond_41sp_Xen_1e-12_Results_allVSall.txt.abc.I25_run_3.trees.tree,70001 trees,out.Diamond_41sp_Xen_1e-12_Results_allVSall.txt.abc.I25_run_4.trees.tree,70

Homo,Xen,Non,Non,41,out.Diamond_41sp_Xen_1e-12_Results_allVSall.txt.abc.I40,1.00E-12,4,37121,out.Diamond_41sp_Xen_1e-12_Results_allVSall.txt.abc.I40_run_1.trees.tree,70001 trees,out.Diamond_41sp_Xen_1e-12_Results_allVSall.txt.abc.I40_run_3.trees.tree,70001 trees,out.Diamond_41sp_Xen_1e-12_Results_allVSall.txt.abc.I40_run_4.trees.tree,70001 trees,,,0.0476993,0.00129607,805,2303,2302,2493,2727,2488,70001,3794,3794,70001

Homo,Xen,Non,Non,41,out.Diamond_41sp_Xen_1e-12_Results_allVSall.txt.abc.I60,1.00E-12,6,40738,out.Diamond_41sp_Xen_1e-12_Results_allVSall.txt.abc.I60_run_1.trees.tree,70001 trees,out.Diamond_41sp_Xen_1e-12_Results_allVSall.txt.abc.I60_run_2.trees.tree,70001 trees,out.Diamond_41sp_Xen_1e-12_Results_allVSall.txt.abc.I60_run_3.trees.tree,70001 trees,out.Diamond_41sp_Xen_1e-12_Results_allVSall.txt.abc.I60_run_4.trees.tree,7000

Homo,Xen,Non,Non,41,Diamond_41sp_Xen_1e-9_Results_allVSall.txt.abc.1.5.mcl.out.fasta:dimensions ntax=41 nchar=24997;,1.00E-09,1.5,24997,out.Diamond_41sp_Xen_1e-9_Results_allVSall.txt.abc.I15_run_1.trees.tree,70001 trees,out.Diamond_41sp_Xen_1e-9_Results_allVSall.txt.abc.I15_run_2.trees.tree,70001 trees,out.Diamond_41sp_Xen_1e-9_Results_allVSall.txt.abc.I15_run_3.trees.tree,70001 trees,out.Diamond_41sp_Xen_1e-9_Results_

Homo,Xen,Non,Non,41,out.Diamond_41sp_Xen_1e-9_Results_allVSall.txt.abc.I20,1.00E-09,2,30099,out.Diamond_41sp_Xen_1e-9_Results_allVSall.txt.abc.I20_run_1.trees.tree,70001 trees,out.Diamond_41sp_Xen_1e-9_Results_allVSall.txt.abc.I20_run_2.trees.tree,70001 trees,out.Diamond_41sp_Xen_1e-9_Results_allVSall.txt.abc.I20_run_3.trees.tree,70001 trees,out.Diamond_41sp_Xen_1e-9_Results_allVSall.txt.abc.I20_run_4.trees.tree,70001 tree

Homo,Xen,Non,Non,41,out.Diamond_41sp_Xen_1e-9_Results_allVSall.txt.abc.I25,1.00E-09,2.5,32985,../../output/out.Diamond_41sp_Xen_1e-9_Results_allVSall.txt.abc.I25_run_1.trees.tree,../../output/out.Diamond_41sp_Xen_1e-9_Results_allVSall.txt.abc.I25_run_2.trees.tree,../../output/out.Diamond_41sp_Xen_1e-9_Results_allVSall.txt.abc.I25_run_3.trees.tree,../../output/out.Diamond_41sp_Xen_1e-9_Results_allVSall.txt.abc.I25_run_4.trees.tree,,,,,0

Homo,Xen,Non,Non,41,Diamond_41sp_Xen_1e-9_Results_allVSall.txt.abc.1e9_M_4.mcl.table.fasta:dimensions ntax=41 nchar=38149;,1.00E-09,4,38149,out.Diamond_41sp_Xen_1e-9_Results_allVSall.txt.abc.I40_run_1.trees.tree,70001 trees,out.Diamond_41sp_Xen_1e-9_Results_allVSall.txt.abc.I40_run_2.trees.tree,70001 trees,out.Diamond_41sp_Xen_1e-9_Results_allVSall.txt.abc.I40_run_3.trees.tree,70001 trees,out.Diamond_41sp_Xen_1e-9_Re

Homo,Xen,Non,Non,41,Diamond_41sp_Xen_1e-9_Results_allVSall.txt.abc.1e9_M_6.mcl.table.fasta:dimensions ntax=41 nchar=41960;,1.00E-09,6,41960,,,,../../output/Diamond_41sp_Xen_1e-9_6_run_1.trees.tree,../../output/Diamond_41sp_Xen_1e-9_6_run_2.trees.tree,../../output/Diamond_41sp_Xen_1e-9_6_run_3.trees.tree,../../output/Diamond_41sp_Xen_1e-9_6_run_4.trees.tree,,,0.0019589,0.000105023,598,7863,7872,8630,9237,8616,50685,1067

Homo,Xen,Non,Non,41,Diamond_41sp_Xen_1e-5_Results_allVSall.txt.abc.1.5.mcl.out.fasta:dimensions ntax=41 nchar=25934;,1.00E-05,1.5,25934,out.Diamond_41sp_Xen_1e-5_Results_allVSall.txt.abc.I15_run_1.trees.tree,70001 trees,out.Diamond_41sp_Xen_1e-5_Results_allVSall.txt.abc.I15_run_2.trees.tree,70001 trees,out.Diamond_41sp_Xen_1e-5_Results_allVSall.txt.abc.I15_run_3.trees.tree,70001 trees,out.Diamond_41sp_Xen_1e-5_Results_

Homo,Xen,Non,Non,41,out.Diamond_41sp_Xen_1e-5_Results_allVSall.txt.abc.I20,1.00E-05,2,31449,../../../output/out.Diamond_41sp_Xen_1e-5_Results_allVSall.txt.abc.I20_run_1.trees.tree : 70001 trees,../../../output/out.Diamond_41sp_Xen_1e-5_Results_allVSall.txt.abc.I20_run_3.trees.tree : 70001 trees,../../../output/out.Diamond_41sp_Xen_1e-5_Results_allVSall.txt.abc.I20_run_4.trees.tree : 70001 trees,,,,,,0.0560421,0.000766485,805,25315,21398

Homo,Xen,Non,Non,41,out.Diamond_41sp_Xen_1e-5_Results_allVSall.txt.abc.I25,1.00E-05,2.5,34553,out.Diamond_41sp_Xen_1e-5_Results_allVSall.txt.abc.I25_run_1.trees.tree,70001 trees,out.Diamond_41sp_Xen_1e-5_Results_allVSall.txt.abc.I25_run_2.trees.tree,70001 trees,out.Diamond_41sp_Xen_1e-5_Results_allVSall.txt.abc.I25_run_3.trees.tree,70001 trees,out.Diamond_41sp_Xen_1e-5_Results_allVSall.txt.abc.I25_run_4.trees.tree,70001 tre

Homo,Xen,Non,Non,41,out.Diamond_41sp_Xen_1e-5_Results_allVSall.txt.abc.I40,1.00E-05,4,40070,out.Diamond_41sp_Xen_1e-5_Results_allVSall.txt.abc.I40_run_2.trees.tree,70001 trees,out.Diamond_41sp_Xen_1e-5_Results_allVSall.txt.abc.I40_run_4.trees.tree,70001 trees,,,,,0.00969986,0.000366279,781,5386,5429,6209,7219,6181,70001,2501,2501,70001,0.00100861,0.0039371,0.00419422,0.00503091,0.00563959,0.00498575,0,0.00580646,0

Homo,Xen,Non,Non,41,out.Diamond_41sp_Xen_1e-5_Results_allVSall.txt.abc.I60,1.00E-05,6,43897,out.Diamond_41sp_Xen_1e-5_Results_allVSall.txt.abc.I60_run_1.trees.tree,70001 trees,out.Diamond_41sp_Xen_1e-5_Results_allVSall.txt.abc.I60_run_2.trees.tree,70001 trees,out.Diamond_41sp_Xen_1e-5_Results_allVSall.txt.abc.I60_run_3.trees.tree,70001 trees,out.Diamond_41sp_Xen_1e-5_Results_allVSall.txt.abc.I60_run_4.trees.tree,70001 tree

Homo,Xen,Non,Non,41,Diamond_41sp_Xen_1e-2_Results_allVSall.txt.abc.1.5.mcl.out.fasta,1.00E-02,1.5,26978,out.Diamond_41sp_Xen_1e-2_Results_allVSall.txt.abc.I15_run_1.trees.tree,70001 trees,out.Diamond_41sp_Xen_1e-2_Results_allVSall.txt.abc.I15_run_2.trees.tree,70001 trees,out.Diamond_41sp_Xen_1e-2_Results_allVSall.txt.abc.I15_run_3.trees.tree,70001 trees,out.Diamond_41sp_Xen_1e-2_Results_allVSall.txt.abc.I15_run_4.trees.tree

Homo,Xen,Non,Non,41,out.Diamond_41sp_Xen_1e-2_Results_allVSall.txt.abc.I20,1.00E-02,2,32804,out.Diamond_41sp_Xen_1e-2_Results_allVSall.txt.abc.I20_run_1.trees.tree,70001 trees,out.Diamond_41sp_Xen_1e-2_Results_allVSall.txt.abc.I20_run_2.trees.tree,70001 trees,out.Diamond_41sp_Xen_1e-2_Results_allVSall.txt.abc.I20_run_3.trees.tree,70001 trees,out.Diamond_41sp_Xen_1e-2_Results_allVSall.txt.abc.I20_run_4.trees.tree,70001 tree

Homo,Xen,Non,Non,41,Diamond_41sp_Xen_1e-2_Results_allVSall.txt.abc.1e2_M_2.5.mcl.out.fasta,1.00E-02,2.5,36015,../../output/Diamond_41sp_Xen_1e-2_2.5_run_1.trees.tree : 48212 trees,../../output/Diamond_41sp_Xen_1e-2_2.5_run_2.trees.tree : 50521 trees,../../output/Diamond_41sp_Xen_1e-2_2.5_run_3.trees.tree : 45089 trees,../../output/Diamond_41sp_Xen_1e-2_2.5_run_4.trees.tree : 49560 trees,,,,,0.0341473,0.000469891,480,1855,1829

Homo,Xen,Non,Non,41,out.Diamond_41sp_Xen_1e-2_Results_allVSall.txt.abc.I40,1.00E-02,4,41673,out.Diamond_41sp_Xen_1e-2_Results_allVSall.txt.abc.I40_run_1.trees.tree,70001 trees,out.Diamond_41sp_Xen_1e-2_Results_allVSall.txt.abc.I40_run_2.trees.tree,70001 trees,out.Diamond_41sp_Xen_1e-2_Results_allVSall.txt.abc.I40_run_3.trees.tree,70001 trees,out.Diamond_41sp_Xen_1e-2_Results_allVSall.txt.abc.I40_run_4.trees.tree,70001 tree

Homo,Xen,Non,Non,41,Diamond_41sp_Xen_1e-2_Results_allVSall.txt.abc.1e2_M_6.mcl.table.fasta:dimensions ntax=41 nchar=45671;,1.00E-02,6,45671,../../output/Diamond_41sp_Xen_1e-2_6_run_1.trees.tree : 44841 trees,../../output/Diamond_41sp_Xen_1e-2_6_run_2.trees.tree : 48322 trees,../../output/Diamond_41sp_Xen_1e-2_6_run_3.trees.tree : 45363 trees,../../output/Diamond_41sp_Xen_1e-2_6_run_4.trees.tree : 45414 trees,,,,,0.0528351,0.0

Homo,Aco,Non,Non,44,Diamond_44sp_Aco_1e-12_Results_allVSall.txt.abc.1.5.mcl.out.fasta:dimensions ntax=44 nchar=27784;,1.00E-12,1.5,27784,out.Diamond_44sp_Aco_1e-12_Results_allVSall.txt.abc.I15_run_1.trees.tree,70001 trees,out.Diamond_44sp_Aco_1e-12_Results_allVSall.txt.abc.I15_run_2.trees.tree,70001 trees,out.Diamond_44sp_Aco_1e-12_Results_allVSall.txt.abc.I15_run_3.trees.tree,70001 trees,out.Diamond_44sp_Aco_1e-12_Res

Homo,Aco,Non,Non,44,out.Diamond_44sp_Aco_1e-12_Results_allVSall.txt.abc.I20,1.00E-12,2,33188,out.Diamond_44sp_Aco_1e-12_Results_allVSall.txt.abc.I20_run_1.trees.tree,70001 trees,out.Diamond_44sp_Aco_1e-12_Results_allVSall.txt.abc.I20_run_2.trees.tree,70001 trees,out.Diamond_44sp_Aco_1e-12_Results_allVSall.txt.abc.I20_run_3.trees.tree,70001 trees,out.Diamond_44sp_Aco_1e-12_Results_allVSall.txt.abc.I20_run_4.trees.tree,7000

Homo,Aco,Non,Non,44,out.Diamond_44sp_Aco_1e-12_Results_allVSall.txt.abc.I25,1.00E-12,2.5,36369,out.Diamond_44sp_Aco_1e-12_Results_allVSall.txt.abc.I25_run_1.trees.tree,70001 trees,out.Diamond_44sp_Aco_1e-12_Results_allVSall.txt.abc.I25_run_2.trees.tree,70001 trees,out.Diamond_44sp_Aco_1e-12_Results_allVSall.txt.abc.I25_run_3.trees.tree,70001 trees,out.Diamond_44sp_Aco_1e-12_Results_allVSall.txt.abc.I25_run_4.trees.tree,700

Homo,Aco,Non,Non,44,out.Diamond_44sp_Aco_1e-12_Results_allVSall.txt.abc.I40,1.00E-12,4,42071,out.Diamond_44sp_Aco_1e-12_Results_allVSall.txt.abc.I40_run_2.trees.tree,70001 trees,out.Diamond_44sp_Aco_1e-12_Results_allVSall.txt.abc.I40_run_4.trees.tree,70001 trees,,,,,0.276639,0.00350901,846,1036,1031,1020,1020,1021,70001,3500,3500,70001,0.00728499,0.0468094,0.0470158,0.0485735,0.0497299,0.0485217,0,0.00235502,0.002

Homo,Aco,Non,Non,44,out.Diamond_44sp_Aco_1e-12_Results_allVSall.txt.abc.I60,1.00E-12,6,46123,out.Diamond_44sp_Aco_1e-12_Results_allVSall.txt.abc.I60_run_1.trees.tree,70001 trees,out.Diamond_44sp_Aco_1e-12_Results_allVSall.txt.abc.I60_run_2.trees.tree,70001 trees,out.Diamond_44sp_Aco_1e-12_Results_allVSall.txt.abc.I60_run_3.trees.tree,70001 trees,out.Diamond_44sp_Aco_1e-12_Results_allVSall.txt.abc.I60_run_4.trees.tree,7000

Homo,Aco,Non,Non,44,Diamond_44sp_Aco_1e-9_Results_allVSall.txt.abc.1.5.mcl.out.fasta:dimensions ntax=44 nchar=28607;,1.00E-09,1.5,28607,out.Diamond_44sp_Aco_1e-9_Results_allVSall.txt.abc.I15_run_1.trees.tree,70001 trees,out.Diamond_44sp_Aco_1e-9_Results_allVSall.txt.abc.I15_run_2.trees.tree,70001 trees,out.Diamond_44sp_Aco_1e-9_Results_allVSall.txt.abc.I15_run_4.trees.tree,70001 trees,,,0.0229854,0.000371891,898,1325,1309

Homo,Aco,Non,Non,44,out.Diamond_44sp_Aco_1e-9_Results_allVSall.txt.abc.I20,1.00E-09,2,34517,out.Diamond_44sp_Aco_1e-9_Results_allVSall.txt.abc.I20_run_1.trees.tree,70001 trees,out.Diamond_44sp_Aco_1e-9_Results_allVSall.txt.abc.I20_run_2.trees.tree,70001 trees,out.Diamond_44sp_Aco_1e-9_Results_allVSall.txt.abc.I20_run_3.trees.tree,70001 trees,out.Diamond_44sp_Aco_1e-9_Results_allVSall.txt.abc.I20_run_4.trees.tree,70001 tree

Homo,Aco,Non,Non,44,out.Diamond_44sp_Aco_1e-9_Results_allVSall.txt.abc.I25,1.00E-09,2.5,37807,out.Diamond_44sp_Aco_1e-9_Results_allVSall.txt.abc.I25_run_1.trees.tree,70001 trees,out.Diamond_44sp_Aco_1e-9_Results_allVSall.txt.abc.I25_run_2.trees.tree,70001 trees,out.Diamond_44sp_Aco_1e-9_Results_allVSall.txt.abc.I25_run_3.trees.tree,70001 trees,out.Diamond_44sp_Aco_1e-9_Results_allVSall.txt.abc.I25_run_4.trees.tree,70001 tre

Homo,Aco,Non,Non,44,out.Diamond_44sp_Aco_1e-9_Results_allVSall.txt.abc.I40,1.00E-09,4,43763,out.Diamond_44sp_Aco_1e-9_Results_allVSall.txt.abc.I40_run_1.trees.tree,70001 trees,out.Diamond_44sp_Aco_1e-9_Results_allVSall.txt.abc.I40_run_2.trees.tree,70001 trees,out.Diamond_44sp_Aco_1e-9_Results_allVSall.txt.abc.I40_run_3.trees.tree,70001 trees,out.Diamond_44sp_Aco_1e-9_Results_allVSall.txt.abc.I40_run_4.trees.tree,70001 trees

Homo,Aco,Non,Non,44,out.Diamond_44sp_Aco_1e-9_Results_allVSall.txt.abc.I60,1.00E-09,6,48076,out.Diamond_44sp_Aco_1e-9_Results_allVSall.txt.abc.I60_run_1.trees.tree,70001 trees,out.Diamond_44sp_Aco_1e-9_Results_allVSall.txt.abc.I60_run_2.trees.tree,70001 trees,out.Diamond_44sp_Aco_1e-9_Results_allVSall.txt.abc.I60_run_3.trees.tree,70001 trees,out.Diamond_44sp_Aco_1e-9_Results_allVSall.txt.abc.I60_run_4.trees.tree,70001 trees

Homo,Aco,Non,Non,44,Diamond_44sp_Aco_1e-5_Results_allVSall.txt.abc.1.5.mcl.out.fasta:dimensions ntax=44 nchar=30267;,1.00E-05,1.5,30267,out.Diamond_44sp_Aco_1e-2_Results_allVSall.txt.abc.I15_run_1.trees.tree,70001 trees,out.Diamond_44sp_Aco_1e-2_Results_allVSall.txt.abc.I15_run_2.trees.tree,70001 trees,out.Diamond_44sp_Aco_1e-2_Results_allVSall.txt.abc.I15_run_3.trees.tree,70001 trees,out.Diamond_44sp_Aco_1e-2_Results_a

Homo,Aco,Non,Non,44,out.Diamond_44sp_Aco_1e-5_Results_allVSall.txt.abc.I20,1.00E-05,2,36817,out.Diamond_44sp_Aco_1e-2_Results_allVSall.txt.abc.I20_run_1.trees.tree,70001 trees,out.Diamond_44sp_Aco_1e-2_Results_allVSall.txt.abc.I20_run_2.trees.tree,70001 trees,out.Diamond_44sp_Aco_1e-2_Results_allVSall.txt.abc.I20_run_3.trees.tree,70001 trees,out.Diamond_44sp_Aco_1e-2_Results_allVSall.txt.abc.I20_run_4.trees.tree,70001 trees

Homo,Aco,Non,Non,44,out.Diamond_44sp_Aco_1e-5_Results_allVSall.txt.abc.I25,1.00E-05,2.5,40465,../../output/out.Diamond_44sp_Aco_1e-5_Results_allVSall.txt.abc.I25_run_1.trees.tree,../../output/out.Diamond_44sp_Aco_1e-5_Results_allVSall.txt.abc.I25_run_2.trees.tree,../../output/out.Diamond_44sp_Aco_1e-5_Results_allVSall.txt.abc.I25_run_3.trees.tree,../../output/out.Diamond_44sp_Aco_1e-5_Results_allVSall.txt.abc.I25_run_4.trees.tree,,,,,0.

Homo,Aco,Non,Non,44,out.Diamond_44sp_Aco_1e-5_Results_allVSall.txt.abc.I40,1.00E-05,4,46871,out.Diamond_44sp_Aco_1e-2_Results_allVSall.txt.abc.I40_run_1.trees.tree,70001 trees,out.Diamond_44sp_Aco_1e-2_Results_allVSall.txt.abc.I40_run_2.trees.tree,70001 trees,out.Diamond_44sp_Aco_1e-2_Results_allVSall.txt.abc.I40_run_3.trees.tree,70001 trees,out.Diamond_44sp_Aco_1e-2_Results_allVSall.txt.abc.I40_run_4.trees.tree,70001 trees

Homo,Aco,Non,Non,44,out.Diamond_44sp_Aco_1e-5_Results_allVSall.txt.abc.I60,1.00E-05,6,51428,out.Diamond_44sp_Aco_1e-2_Results_allVSall.txt.abc.I60_run_1.trees.tree,70001 trees,out.Diamond_44sp_Aco_1e-2_Results_allVSall.txt.abc.I60_run_2.trees.tree,70001 trees,out.Diamond_44sp_Aco_1e-2_Results_allVSall.txt.abc.I60_run_3.trees.tree,70001 trees,out.Diamond_44sp_Aco_1e-2_Results_allVSall.txt.abc.I60_run_4.trees.tree,70001 trees

Homo,Aco,Non,Non,44,Diamond_44sp_Aco_1e-2_Results_allVSall.txt.abc.1.5.mcl.out.fasta:dimensions ntax=44 nchar=31891;,1.00E-02,1.5,31891,out.Diamond_44sp_Aco_1e-5_Results_allVSall.txt.abc.I15_run_1.trees.tree,70001 trees,out.Diamond_44sp_Aco_1e-5_Results_allVSall.txt.abc.I15_run_2.trees.tree,70001 trees,out.Diamond_44sp_Aco_1e-5_Results_allVSall.txt.abc.I15_run_3.trees.tree,70001 trees,out.Diamond_44sp_Aco_1e-5_Results_a

Homo,Aco,Non,Non,44,out.Diamond_44sp_Aco_1e-2_Results_allVSall.txt.abc.I20,1.00E-02,2,38977,../../output/out.Diamond_44sp_Aco_1e-2_Results_allVSall.txt.abc.I20_run_1.trees.tree,../../output/out.Diamond_44sp_Aco_1e-2_Results_allVSall.txt.abc.I20_run_2.trees.tree,../../output/out.Diamond_44sp_Aco_1e-2_Results_allVSall.txt.abc.I20_run_3.trees.tree,../../output/out.Diamond_44sp_Aco_1e-2_Results_allVSall.txt.abc.I20_run_4.trees.tree,,,,,0.05

Homo,Aco,Non,Non,44,out.Diamond_44sp_Aco_1e-2_Results_allVSall.txt.abc.I25,1.00E-02,2.5,42881,out.Diamond_44sp_Aco_1e-5_Results_allVSall.txt.abc.I25_run_1.trees.tree,70001 trees,out.Diamond_44sp_Aco_1e-5_Results_allVSall.txt.abc.I25_run_2.trees.tree,70001 trees,out.Diamond_44sp_Aco_1e-5_Results_allVSall.txt.abc.I25_run_3.trees.tree,70001 trees,out.Diamond_44sp_Aco_1e-5_Results_allVSall.txt.abc.I25_run_4.trees.tree,70001 tre

Homo,Aco,Non,Non,44,out.Diamond_44sp_Aco_1e-2_Results_allVSall.txt.abc.I40,1.00E-02,4,49635,out.Diamond_44sp_Aco_1e-5_Results_allVSall.txt.abc.I40_run_1.trees.tree,70001 trees,out.Diamond_44sp_Aco_1e-5_Results_allVSall.txt.abc.I40_run_2.trees.tree,70001 trees,out.Diamond_44sp_Aco_1e-5_Results_allVSall.txt.abc.I40_run_3.trees.tree,70001 trees,out.Diamond_44sp_Aco_1e-5_Results_allVSall.txt.abc.I40_run_4.trees.tree,70001 trees

Homo,Aco,Non,Non,44,out.Diamond_44sp_Aco_1e-2_Results_allVSall.txt.abc.I60,1.00E-02,6,54447,out.Diamond_44sp_Aco_1e-5_Results_allVSall.txt.abc.I60_run_1.trees.tree,70001 trees,out.Diamond_44sp_Aco_1e-5_Results_allVSall.txt.abc.I60_run_2.trees.tree,70001 trees,out.Diamond_44sp_Aco_1e-5_Results_allVSall.txt.abc.I60_run_3.trees.tree,70001 trees,out.Diamond_44sp_Aco_1e-5_Results_allVSall.txt.abc.I60_run_4.trees.tree,70001 trees

Homo,Opi,Non,Non,47,Diamond_47sp_Opi_1e-12_Results_allVSall.txt.abc.1.5.mcl.out.fasta:dimensions ntax=47 nchar=28679;,1.00E-12,1.5,28679,Diamond_47sp_Opi_1e-12_Results_allVSall.txt.abc.1.5.mcl.out.fasta_run_1.trees.tree,100001 trees,Diamond_47sp_Opi_1e-12_Results_allVSall.txt.abc.1.5.mcl.out.fasta_run_2.trees.tree,100001 trees,Diamond_47sp_Opi_1e-12_Results_allVSall.txt.abc.1.5.mcl.out.fasta_run_3.trees.tree,100001 trees,Diam

Homo,Opi,Non,Non,47,Diamond_47sp_Opi_1e-12_Results_allVSall.txt.abc.2.mcl.out.fasta:dimensions ntax=47 nchar=34487;,1.00E-12,2,34487,out.Diamond_47sp_Opi_1e-12_Results_allVSall.txt.abc.I20_run_2.trees.tree,70001 trees,out.Diamond_47sp_Opi_1e-12_Results_allVSall.txt.abc.I20_run_3.trees.tree,70001 trees,out.Diamond_47sp_Opi_1e-12_Results_allVSall.txt.abc.I20_run_4.trees.tree,70001 trees,,,0.0760132,0.000676674,890,949,946,1

Homo,Opi,Non,Non,47,Diamond_47sp_Opi_1e-12_Results_allVSall.txt.abc.2.5.mcl.out.fasta:dimensions ntax=47 nchar=37860;,1.00E-12,2.5,37860,Diamond_47sp_Opi_1e-12_Results_allVSall.txt.abc.2.5.mcl.out.fasta_run_1.trees.tree,82174 trees,Diamond_47sp_Opi_1e-12_Results_allVSall.txt.abc.2.5.mcl.out.fasta_run_2.trees.tree,78042 trees,Diamond_47sp_Opi_1e-12_Results_allVSall.txt.abc.2.5.mcl.out.fasta_run_3.trees.tree,96699 trees,Diamond

Homo,Opi,Non,Non,47,Diamond_47sp_Opi_1e-12_Results_allVSall.txt.abc.4.mcl.out.fasta:dimensions ntax=47 nchar=44138;,1.00E-12,4,44138,out.Diamond_47sp_Opi_1e-12_Results_allVSall.txt.abc.I40_run_1.trees.tree,70001 trees,out.Diamond_47sp_Opi_1e-12_Results_allVSall.txt.abc.I40_run_2.trees.tree,70001 trees,out.Diamond_47sp_Opi_1e-12_Results_allVSall.txt.abc.I40_run_3.trees.tree,70001 trees,out.Diamond_47sp_Opi_1e-12_Results_a

Homo,Opi,Non,Non,47,Diamond_47sp_Opi_1e-12_Results_allVSall.txt.abc.6.mcl.out.fasta:dimensions ntax=47 nchar=48738;,1.00E-12,6,48738,out.Diamond_47sp_Opi_1e-12_Results_allVSall.txt.abc.I60_run_1.trees.tree,70001 trees,out.Diamond_47sp_Opi_1e-12_Results_allVSall.txt.abc.I60_run_2.trees.tree,70001 trees,out.Diamond_47sp_Opi_1e-12_Results_allVSall.txt.abc.I60_run_3.trees.tree,70001 trees,out.Diamond_47sp_Opi_1e-12_Results_a

Homo,Opi,Non,Non,47,Diamond_47sp_Opi_1e-9_Results_allVSall.txt.abc.1.5.mcl.out.fasta:dimensions ntax=47 nchar=29659;,1.00E-09,1.5,29659,Diamond_47sp_Opi_1e-9_Results_allVSall.txt.abc.1.5.mcl.out.fasta_run_1.trees.tree,93534 trees,Diamond_47sp_Opi_1e-9_Results_allVSall.txt.abc.1.5.mcl.out.fasta_run_2.trees.tree,97986 trees,Diamond_47sp_Opi_1e-9_Results_allVSall.txt.abc.1.5.mcl.out.fasta_run_3.trees.tree,87507 trees,Diamond_47s

Homo,Opi,Non,Non,47,Diamond_47sp_Opi_1e-9_Results_allVSall.txt.abc.2.mcl.out.fasta:dimensions ntax=47 nchar=36008;,1.00E-09,2,36008,out.Diamond_47sp_Opi_1e-9_Results_allVSall.txt.abc.I20_run_1.trees.tree,70001 trees,out.Diamond_47sp_Opi_1e-9_Results_allVSall.txt.abc.I20_run_2.trees.tree,70001 trees,out.Diamond_47sp_Opi_1e-9_Results_allVSall.txt.abc.I20_run_3.trees.tree,70001 trees,out.Diamond_47sp_Opi_1e-9_Results_allVSal

Homo,Opi,Non,Non,47,Diamond_47sp_Opi_1e-9_Results_allVSall.txt.abc.2.5.mcl.out.fasta:dimensions ntax=47 nchar=39591;,1.00E-09,2.5,39591,Diamond_47sp_Opi_1e-9_Results_allVSall.txt.abc.2.5.mcl.out.fasta_run_1.trees.tree,75450 trees,Diamond_47sp_Opi_1e-9_Results_allVSall.txt.abc.2.5.mcl.out.fasta_run_2.trees.tree,75413 trees,Diamond_47sp_Opi_1e-9_Results_allVSall.txt.abc.2.5.mcl.out.fasta_run_3.trees.tree,76800 trees,Diamond_47s

Homo,Opi,Non,Non,47,Diamond_47sp_Opi_1e-9_Results_allVSall.txt.abc.4.mcl.out.fasta:dimensions ntax=47 nchar=46175;,1.00E-09,4,46175,out.Diamond_47sp_Opi_1e-9_Results_allVSall.txt.abc.I40_run_1.trees.tree,70001 trees,out.Diamond_47sp_Opi_1e-9_Results_allVSall.txt.abc.I40_run_2.trees.tree,70001 trees,out.Diamond_47sp_Opi_1e-9_Results_allVSall.txt.abc.I40_run_3.trees.tree,70001 trees,out.Diamond_47sp_Opi_1e-9_Results_allVSal

Homo,Opi,Non,Non,47,Diamond_47sp_Opi_1e-9_Results_allVSall.txt.abc.6.mcl.out.fasta:dimensions ntax=47 nchar=51122;,1.00E-09,6,51122,out.Diamond_47sp_Opi_1e-9_Results_allVSall.txt.abc.I60_run_1.trees.tree,70001 trees,out.Diamond_47sp_Opi_1e-9_Results_allVSall.txt.abc.I60_run_2.trees.tree,70001 trees,out.Diamond_47sp_Opi_1e-9_Results_allVSall.txt.abc.I60_run_3.trees.tree,70001 trees,out.Diamond_47sp_Opi_1e-9_Results_allVSall

Homo,Opi,Non,Non,47,Diamond_47sp_Opi_1e-5_Results_allVSall.txt.abc.1.5.mcl.out.fasta:dimensions ntax=47 nchar=31634;,1.00E-05,1.5,31634,Diamond_47sp_Opi_1e-5_Results_allVSall.txt.abc.1.5.mcl.out.fasta_run_1.trees.tree,81835 trees,Diamond_47sp_Opi_1e-5_Results_allVSall.txt.abc.1.5.mcl.out.fasta_run_2.trees.tree,78073 trees,Diamond_47sp_Opi_1e-5_Results_allVSall.txt.abc.1.5.mcl.out.fasta_run_3.trees.tree,82008 trees,Diamond_47s

Homo,Opi,Non,Non,47,Diamond_47sp_Opi_1e-5_Results_allVSall.txt.abc.2.mcl.out.fasta:dimensions ntax=47 nchar=38721;,1.00E-05,2,38721,out.Diamond_47sp_Opi_1e-5_Results_allVSall.txt.abc.I20_run_1.trees.tree,70001 trees,out.Diamond_47sp_Opi_1e-5_Results_allVSall.txt.abc.I20_run_2.trees.tree,70001 trees,out.Diamond_47sp_Opi_1e-5_Results_allVSall.txt.abc.I20_run_3.trees.tree,70001 trees,out.Diamond_47sp_Opi_1e-5_Results_allVSall

Homo,Opi,Non,Non,47,Diamond_47sp_Opi_1e-5_Results_allVSall.txt.abc.2.5.mcl.out.fasta:dimensions ntax=47 nchar=42746;,1.00E-05,2.5,42746,Diamond_47sp_Opi_1e-5_Results_allVSall.txt.abc.2.5.mcl.out.fasta_run_1.trees.tree,70619 trees,Diamond_47sp_Opi_1e-5_Results_allVSall.txt.abc.2.5.mcl.out.fasta_run_2.trees.tree,71171 trees,Diamond_47sp_Opi_1e-5_Results_allVSall.txt.abc.2.5.mcl.out.fasta_run_3.trees.tree,71296 trees,Diamond_47s

Homo,Opi,Non,Non,47,Diamond_47sp_Opi_1e-5_Results_allVSall.txt.abc.4.mcl.out.fasta:dimensions ntax=47 nchar=49865;,1.00E-05,4,49865,out.Diamond_47sp_Opi_1e-5_Results_allVSall.txt.abc.I40_run_2.trees.tree,70001 trees,out.Diamond_47sp_Opi_1e-5_Results_allVSall.txt.abc.I40_run_3.trees.tree,70001 trees,,,,,0.00819988,0.00026065,724,1012,1014,1081,1158,1079,70001,1580,1580,70001,0.000866509,0.0045971,0.00453285,0.00426457

Homo,Opi,Non,Non,47,Diamond_47sp_Opi_1e-5_Results_allVSall.txt.abc.6.mcl.out.fasta:dimensions ntax=47 nchar=55118;,1.00E-05,6,55118,out.Diamond_47sp_Opi_1e-5_Results_allVSall.txt.abc.I60_run_1.trees.tree,70001 trees,out.Diamond_47sp_Opi_1e-5_Results_allVSall.txt.abc.I60_run_2.trees.tree,70001 trees,out.Diamond_47sp_Opi_1e-5_Results_allVSall.txt.abc.I60_run_3.trees.tree,70001 trees,out.Diamond_47sp_Opi_1e-5_Results_allVSall

Homo,Opi,Non,Non,47,Diamond_47sp_Opi_1e-2_Results_allVSall.txt.abc.1.5.mcl.out.fasta:dimensions ntax=47 nchar=33506;,1.00E-02,1.5,33506,Diamond_47sp_Opi_1e-2_Results_allVSall.txt.abc.1.5.mcl.out.fasta_run_1.trees.tree,71563 trees,Diamond_47sp_Opi_1e-2_Results_allVSall.txt.abc.1.5.mcl.out.fasta_run_2.trees.tree,75017 trees,Diamond_47sp_Opi_1e-2_Results_allVSall.txt.abc.1.5.mcl.out.fasta_run_3.trees.tree,88653 trees,Diamond_47s

Homo,Opi,Non,Non,47,Diamond_47sp_Opi_1e-2_Results_allVSall.txt.abc.2.mcl.out.fasta:dimensions ntax=47 nchar=41248;,1.00E-02,2,41248,out.Diamond_47sp_Opi_1e-2_Results_allVSall.txt.abc.I20_run_1.trees.tree,70001 trees,out.Diamond_47sp_Opi_1e-2_Results_allVSall.txt.abc.I20_run_2.trees.tree,70001 trees,out.Diamond_47sp_Opi_1e-2_Results_allVSall.txt.abc.I20_run_3.trees.tree,70001 trees,out.Diamond_47sp_Opi_1e-2_Results_allVSall

Homo,Opi,Non,Non,47,Diamond_47sp_Opi_1e-2_Results_allVSall.txt.abc.2.5.mcl.out.fasta:dimensions ntax=47 nchar=45532;,1.00E-02,2.5,45532,Diamond_47sp_Opi_1e-2_Results_allVSall.txt.abc.2.5.mcl.out.fasta_run_1.trees.tree,69176 trees,Diamond_47sp_Opi_1e-2_Results_allVSall.txt.abc.2.5.mcl.out.fasta_run_2.trees.tree,65064 trees,Diamond_47sp_Opi_1e-2_Results_allVSall.txt.abc.2.5.mcl.out.fasta_run_3.trees.tree,71355 trees,Diamond_47s

Homo,Opi,Non,Non,47,Diamond_47sp_Opi_1e-2_Results_allVSall.txt.abc.4.mcl.out.fasta:dimensions ntax=47 nchar=53090;,1.00E-02,4,53090,../../output/Results_1e-2._47sp_4.mcl.out.fasta_run_1.trees.tree,../../output/Results_1e-2._47sp_4.mcl.out.fasta_run_2.trees.tree,../../output/Results_1e-2._47sp_4.mcl.out.fasta_run_3.trees.tree,../../output/Results_1e-2._47sp_4.mcl.out.fasta_run_4.trees.tree,,,,,0.0179283,0.000363992,299,314,313,323,341,323,

Ortho,Xen,Non,Non,41,Results_1e-12._41sp_1.5,1.00E-12,1.5,28377,../../output/Results_1e-12._41sp_1.5_run_1.trees.tree : 64544 trees,../../output/Results_1e-12._41sp_1.5_run_2.trees.tree : 51938 trees,../../output/Results_1e-12._41sp_1.5_run_3.trees.tree : 51837 trees,../../output/Results_1e-12._41sp_1.5_run_4.trees.tree : 55289 trees,,,,,0.0968941,0.000698049,655,2044,2018,2094,2201,2099,51884,1416,1416,51884,0.0130712,0.0323874,0.032

Ortho,Xen,Non,Non,41,Results_1e-5._41sp_1.5,1.00E-05,1.5,28993,../output/Results_1e-5._41sp_1.5_run_1.trees.tree,../output/Results_1e-5._41sp_1.5_run_2.trees.tree,../output/Results_1e-5._41sp_1.5_run_3.trees.tree,../output/Results_1e-5._41sp_1.5_run_4.trees.tree,,,,,0.00448232,0.000164589,806,1834,1813,1868,1964,1872,72420,2126,2126,72420,0.00498449,0.0190094,0.0190459,0.0181747,0.0189805,0.0181785,0,0.00731524,2126,0,,806

Ortho,Xen,Non,Non,41,Results_1e-12._41sp_2.mcl.out.fasta,1.00E-12,2,33387,Results_1e-12._41sp_2.mcl.out.fasta_run_1.trees.tree,70001 trees,Results_1e-12._41sp_2.mcl.out.fasta_run_2.trees.tree,70001 trees,Results_1e-12._41sp_2.mcl.out.fasta_run_3.trees.tree,70001 trees,Results_1e-12._41sp_2.mcl.out.fasta_run_4.trees.tree,70001 trees,0.00534278,0.000130572,833,2756,2711,2887,3147,2891,70001,3128,3128,70001,0.00548813,0.01773

Ortho,Xen,Non,Non,41,Results_1e-12._41sp_2.5.mcl.out.fasta,1.00E-12,2.5,36290,Results_1e-12._41sp_2.5.mcl.out.fasta_run_1.trees.tree,70001 trees,Results_1e-12._41sp_2.5.mcl.out.fasta_run_2.trees.tree,70001 trees,Results_1e-12._41sp_2.5.mcl.out.fasta_run_3.trees.tree,70001 trees,Results_1e-12._41sp_2.5.mcl.out.fasta_run_4.trees.tree,70001 trees,0.00324281,0.0000667504,974,10442,10774,8663,7612,8632,70001,988,988,70001,0.0076

Ortho,Xen,Non,Non,41,Results_1e-12._41sp_4.mcl.out.fasta,1.00E-12,4,42110,Results_1e-12._41sp_4.mcl.out.fasta_run_1.trees.tree,70001 trees,Results_1e-12._41sp_4.mcl.out.fasta_run_2.trees.tree,70001 trees,Results_1e-12._41sp_4.mcl.out.fasta_run_3.trees.tree,70001 trees,Results_1e-12._41sp_4.mcl.out.fasta_run_4.trees.tree,70001 trees,0.0237997,0.000336286,861,31358,31529,27359,25274,27373,70001,2193,2193,70001,0.0174177,0.03

Ortho,Xen,Non,Non,41,Results_1e-12._41sp_6.mcl.out.fasta,1.00E-12,6,46402,Results_1e-12._41sp_6.mcl.out.fasta_run_1.trees.tree,70001 trees,Results_1e-12._41sp_6.mcl.out.fasta_run_2.trees.tree,70001 trees,Results_1e-12._41sp_6.mcl.out.fasta_run_3.trees.tree,70001 trees,Results_1e-12._41sp_6.mcl.out.fasta_run_4.trees.tree,70001 trees,0.00945701,0.000168592,869,20786,20918,22475,23191,22372,70001,2900,2900,70001,0.00917093,0.

Ortho,Xen,Non,Non,41,Diamond_44sp_Aco_1e-9_Results_allVSall.txt.abc.1.5.mcl.out.fasta:dimensions ntax=44 nchar=28607;,1.00E-09,1.5,28607,../output/Results_1e-9._41sp_1.5_run_1.trees.tree,../output/Results_1e-9._41sp_1.5_run_2.trees.tree,../output/Results_1e-9._41sp_1.5_run_3.trees.tree,../output/Results_1e-9._41sp_1.5_run_4.trees.tree,,,,,0.0216008,0.0004012,343,644,640,616,623,618,25724,886,886,25724,0.0102393,0.0112483,0.011

Ortho,Xen,Non,Non,41,Results_1e-9._41sp_2,1.00E-09,2,34099,../../output/Results_1e-9._41sp_2_run_1.trees.tree : 22982 trees,../../output/Results_1e-9._41sp_2_run_2.trees.tree : 20900 trees,../../output/Results_1e-9._41sp_2_run_3.trees.tree : 26775 trees,../../output/Results_1e-9._41sp_2_run_4.trees.tree : 23041 trees,,,,,0.0145844,0.000389869,253,787,782,777,794,779,20908,571,571,20908,0.0138338,0.0164482,0.0162579,0.0177855,0.018869

Ortho,Xen,Non,Non,41,Results_1e-9._41sp_2.5,1.00E-09,2.5,37238,../output/Results_1e-9._41sp_2.5_run_1.trees.tree,../output/Results_1e-9._41sp_2.5_run_2.trees.tree,../output/Results_1e-9._41sp_2.5_run_3.trees.tree,../output/Results_1e-9._41sp_2.5_run_4.trees.tree,,,,,0.0102465,0.000377261,244,1282,1266,1359,1482,1361,21997,692,692,21997,0.0122405,0.0473756,0.0473213,0.0493825,0.0505381,0.0493573,0,0.0228711,692,0,,244,"(ACDI:

Ortho,Xen,Non,Non,41,Results_1e-9._41sp_4,1.00E-09,4,43348,../output/Results_1e-9._41sp_4_run_1.trees.tree,../output/Results_1e-9._41sp_4_run_2.trees.tree,../output/Results_1e-9._41sp_4_run_3.trees.tree,../output/Results_1e-9._41sp_4_run_4.trees.tree,,,,,0.212352,0.00174669,250,9627,9548,8579,9531,8588,20606,959,959,20606,0.00862455,0.0221751,0.0222803,0.0250886,0.0272337,0.0250353,0,0.0188257,959,0,,250,"(ACDI:0.001397,NE

Ortho,Xen,Non,Non,41,Results_1e-9._41sp_6,1.00E-09,6,47860,../output/Results_1e-9._41sp_6_run_1.trees.tree,../output/Results_1e-9._41sp_6_run_2.trees.tree,../output/Results_1e-9._41sp_6_run_3.trees.tree,../output/Results_1e-9._41sp_6_run_4.trees.tree,,,,,0.00928093,0.000364359,249,1877,1904,2104,2597,2087,20912,462,462,20912,0.0108838,0.0632318,0.0630009,0.0602234,0.0568846,0.0602924,0,0.0330561,462,0,,249,"(ACDI:0.001635

Ortho,Xen,Non,Non,41,Results_1e-5._41sp_2,1.00E-05,2,35194,../../output/Results_1e-5._41sp_2_run_1.trees.tree : 50674 trees,../../output/Results_1e-5._41sp_2_run_2.trees.tree : 78869 trees,../../output/Results_1e-5._41sp_2_run_3.trees.tree : 66886 trees,../../output/Results_1e-5._41sp_2_run_4.trees.tree : 69718 trees,,,,,0.0263657,0.000371728,611,5814,5628,7458,10052,7496,50732,1494,1494,50732,0.0152806,0.0241139,0.0240921,0.0253433

Ortho,Xen,Non,Non,41,Results_1e-5._41sp_2.5,1.00E-05,2.5,38630,../output/Results_1e-5._41sp_2.5_run_1.trees.tree,../output/Results_1e-5._41sp_2.5_run_2.trees.tree,../output/Results_1e-5._41sp_2.5_run_3.trees.tree,../output/Results_1e-5._41sp_2.5_run_4.trees.tree,,,,,0.405216,0.00273128,745,10405,10388,9830,9655,9837,63410,2510,2510,63410,0.00783669,0.0391818,0.0390404,0.0386391,0.0378771,0.0386752,0,0.0188868,2510,0,,745,"(

Ortho,Xen,Non,Non,41,Results_1e-5._41sp_4,1.00E-05,4,45343,../output/Results_1e-5._41sp_4_run_1.trees.tree,../output/Results_1e-5._41sp_4_run_2.trees.tree,../output/Results_1e-5._41sp_4_run_3.trees.tree,../output/Results_1e-5._41sp_4_run_4.trees.tree,,,,,0.0106715,0.000214198,743,16777,17063,24187,36008,23946,65998,2314,2314,65998,0.00821585,0.00755801,0.00783912,0.009908,0.0115294,0.00984479,0,0.00709257,2314,0,,743,"(AC

Ortho,Xen,Non,Non,41,Results_1e-5._41sp_6,1.00E-05,6,50013,../output/Results_1e-5._41sp_6_run_1.trees.tree,../output/Results_1e-5._41sp_6_run_2.trees.tree,../output/Results_1e-5._41sp_6_run_3.trees.tree,../output/Results_1e-5._41sp_6_run_4.trees.tree,,,,,0.009716,0.00025847,468,23126,23098,11551,6431,11699,40249,1092,1092,40249,0.00538502,0.0267826,0.0268134,0.0273434,0.0275295,0.0273362,0,0.0114382,1092,0,,468,"(ACDI:0

Ortho,Xen,Non,Non,41,Results_1e-2._41sp_1.5,1.00E-02,1.5,29593,../output/Results_1e-2._41sp_1.5_run_1.trees.tree,../output/Results_1e-2._41sp_1.5_run_2.trees.tree,../output/Results_1e-2._41sp_1.5_run_3.trees.tree,../output/Results_1e-2._41sp_1.5_run_4.trees.tree,,,,,0.0292204,0.000293754,539,1264,1252,1263,1318,1266,53085,1004,1004,53085,0.000717753,0.0158121,0.0156048,0.0182788,0.0199498,0.0182846,0,0.00719826,1004,0,,539

Ortho,Xen,Non,Non,41,Results_1e-2._41sp_2.mcl.out.fasta,1.00E-02,2,36289,Results_1e-2._41sp_2.mcl.out.fasta_run_1.trees.tree,70001 trees,Results_1e-2._41sp_2.mcl.out.fasta_run_2.trees.tree,70001 trees,,,,70001 trees,0.00807131,0.000138446,727,5971,5866,6784,7792,6794,70001,1604,1604,70001,0.0191508,0.0234684,0.02341,0.0236311,0.0236146,0.0236388,0,0.00239169,0.00239193,0,,727,"(ACDI:0.002019,NEVE:0.002117,(HYMA:0.00

Ortho,Xen,Non,Non,41,Results_1e-2._41sp_2.5.mcl.out.fasta,1.00E-02,2.5,40139,Results_1e-2._41sp_2.5.mcl.out.fasta_run_2.trees.tree,70001 trees,Results_1e-2._41sp_2.5.mcl.out.fasta_run_3.trees.tree,70001 trees,,,,,0.0206426,0.000362479,903,32129,32196,30495,29422,30479,70001,640,640,70001,0.00300884,0.00923475,0.00914184,0.0100071,0.0106551,0.0100175,0,0.00194385,0.00193905,0,,640,"(ACDI:0.002271,NEVE:0.002367,(HYMA:

Ortho,Xen,Non,Non,41,Results_1e-2._41sp_4.mcl.out.fasta,1.00E-02,4,47102,Results_1e-2._41sp_4.mcl.out.fasta_run_1.trees.tree,70001 trees,Results_1e-2._41sp_4.mcl.out.fasta_run_2.trees.tree,70001 trees,Results_1e-2._41sp_4.mcl.out.fasta_run_3.trees.tree,70001 trees,Results_1e-2._41sp_4.mcl.out.fasta_run_4.trees.tree,70001 trees,0.012157,0.000197054,846,14164,14264,12780,11777,12774,70001,1836,1836,70001,0.00947777,0.0204119,

Ortho,Xen,Non,Non,41,Results_1e-2._41sp_6.mcl.out.fasta,1.00E-02,6,52029,Results_1e-2._41sp_6.mcl.out.fasta_run_1.trees.tree,70001 trees,Results_1e-2._41sp_6.mcl.out.fasta_run_2.trees.tree,70001 trees,Results_1e-2._41sp_6.mcl.out.fasta_run_3.trees.tree,70001 trees,Results_1e-2._41sp_6.mcl.out.fasta_run_4.trees.tree,70001 trees,0.0108998,0.000152656,806,54625,54600,54000,33446,54293,70001,2109,2109,70001,0.0167104,0.0151384,

Ortho,Aco,Non,Non,44,Results_1e-12._44sp_1.5.mcl.out.fasta,1.00E-12,1.5,32723,Results_1e-12._44sp_1.5.mcl.out.fasta_run_1.trees.tree,60001 trees,output/Results_1e-12._44sp_1.5.mcl.out.fasta_run_2.trees.tree,60001 trees,output/Results_1e-12._44sp_1.5.mcl.out.fasta_run_3.trees.tree,60001 trees,output/Results_1e-12._44sp_1.5.mcl.out.fasta_run_4.trees.tree,60001 trees,0.0576324,0.000513379,803,955,947,980,1024,981,60001,1272,1272,60

Ortho,Aco,Non,Non,44,Results_1e-12._44sp_2.mcl.out.fasta,1.00E-12,2,38820,Results_1e-12._44sp_2.mcl.out.fasta_run_1.trees.tree,70001 trees,Results_1e-12._44sp_2.mcl.out.fasta_run_2.trees.tree,70001 trees,Results_1e-12._44sp_2.mcl.out.fasta_run_3.trees.tree,70001 trees,Results_1e-12._44sp_2.mcl.out.fasta_run_4.trees.tree,70001 trees,0.00677133,0.000113867,900,2123,2103,2220,2375,2221,70001,2543,2544,70001,0.00481658,0.01782

Ortho,Aco,Non,Non,44,Results_1e-12._44sp_2.5.mcl.out.fasta,1.00E-12,2.5,42309,Results_1e-12._44sp_2.5.mcl.out.fasta_run_1.trees.tree,70001 trees,Results_1e-12._44sp_2.5.mcl.out.fasta_run_2.trees.tree,70001 trees,Results_1e-12._44sp_2.5.mcl.out.fasta_run_3.trees.tree,70001 trees,Results_1e-12._44sp_2.5.mcl.out.fasta_run_4.trees.tree,70001 trees,0.0076856,0.000130788,920,3740,3721,4040,4384,4038,70001,2089,2089,70001,0.0161422,

Ortho,Aco,Non,Non,44,Results_1e-12._44sp_4.mcl.out.fasta,1.00E-12,4,49354,Results_1e-12._44sp_4.mcl.out.fasta_run_1.trees.tree,70001 trees,Results_1e-12._44sp_4.mcl.out.fasta_run_2.trees.tree,70001 trees,Results_1e-12._44sp_4.mcl.out.fasta_run_3.trees.tree,70001 trees,Results_1e-12._44sp_4.mcl.out.fasta_run_4.trees.tree,70001 trees,0.0010857,0.0000707166,778,66149,65915,51025,30753,52233,70001,1776,1776,70001,0.00553469,0.

Ortho,Aco,Non,Non,44,Results_1e-12._44sp_6.mcl.out.fasta,1.00E-12,6,54324,Results_1e-12._44sp_6.mcl.out.fasta_run_1.trees.tree,70001 trees,Results_1e-12._44sp_6.mcl.out.fasta_run_2.trees.tree,70001 trees,Results_1e-12._44sp_6.mcl.out.fasta_run_3.trees.tree,70001 trees,Results_1e-12._44sp_6.mcl.out.fasta_run_4.trees.tree,70001 trees,0.0167855,0.000236373,878,23353,23495,21261,16105,21478,70001,3344,3344,70001,0.00818513,0.0

Ortho,Aco,Non,Non,44,Results_1e-9._44sp_1.5,1.00E-09,1.5,33692,Results_1e-9._44sp_1.5.mcl.out.fasta_run_1.trees.tree,6001 trees,Results_1e-9._44sp_1.5.mcl.out.fasta_run_2.trees.tree,6001 trees,Results_1e-9._44sp_1.5.mcl.out.fasta_run_3.trees.tree,6001 trees,Results_1e-9._44sp_1.5.mcl.out.fasta_run_4.trees.tree,6001 trees,0.130978,0.000867756,649,588,586,574,578,574,6001,3270,3270,6001,0.00742602,0.025387,0.0253357,0.0252261,0

Ortho,Aco,Non,Non,44,Results_1e-9._44sp_2,1.00E-09,2,40442,Results_1e-9._44sp_2.mcl.out.fasta_run_1.trees.tree,70001 trees,Results_1e-9._44sp_2.mcl.out.fasta_run_2.trees.tree,70001 trees,Results_1e-9._44sp_2.mcl.out.fasta_run_3.trees.tree,70001 trees,Results_1e-9._44sp_2.mcl.out.fasta_run_4.trees.tree,70001 trees,0.0663562,0.000480422,948,4488,4301,6059,8901,6102,70001,2277,2277,70001,0.00652319,0.0125977,0.0125727,0.0110

Ortho,Aco,Non,Non,44,Results_1e-9._44sp_2.5.mcl.out.fasta,1.00E-09,2.5,44383,Results_1e-9._44sp_2.5.mcl.out.fasta_run_1.trees.tree,70001 trees,Results_1e-9._44sp_2.5.mcl.out.fasta_run_2.trees.tree,70001 trees,Results_1e-9._44sp_2.5.mcl.out.fasta_run_3.trees.tree,70001 trees,Results_1e-9._44sp_2.5.mcl.out.fasta_run_4.trees.tree,70001 trees,0.00304281,0.000112907,827,2552,2512,2650,2835,2652,70001,1991,1991,70001,0.00837649,0.03

Ortho,Aco,Non,Non,44,Results_1e-9._44sp_4,1.00E-09,4,51836,Results_1e-9._44sp_4.mcl.out.fasta_run_1.trees.tree,70001 trees,Results_1e-9._44sp_4.mcl.out.fasta_run_2.trees.tree,70001 trees,Results_1e-9._44sp_4.mcl.out.fasta_run_3.trees.tree,70001 trees,Results_1e-9._44sp_4.mcl.out.fasta_run_4.trees.tree,70001 trees,0.0846559,0.000664415,905,4384,4374,4690,5208,4677,70001,2538,2538,70001,0.00610932,0.00555329,0.0054749,0.004

Ortho,Aco,Non,Non,44,Results_1e-9._44sp_6,1.00E-09,6,57173,Results_1e-9._44sp_6.mcl.out.fasta_run_1.trees.tree,70001 trees,Results_1e-9._44sp_6.mcl.out.fasta_run_2.trees.tree,70001 trees,Results_1e-9._44sp_6.mcl.out.fasta_run_3.trees.tree,70001 trees,Results_1e-9._44sp_6.mcl.out.fasta_run_4.trees.tree,70001 trees,0.0148712,0.000174828,936,53239,53023,50120,41511,50234,70001,1751,1751,70001,0.00996446,0.0190714,0.0192467,0

Ortho,Aco,Non,Non,44,Results_1e-5._44sp_1.5.mcl.out.fasta,1.00E-05,1.5,35135,Results_1e-5._44sp_1.5.mcl.out.fasta_run_1.trees.tree,70001 trees,Results_1e-5._44sp_1.5.mcl.out.fasta_run_2.trees.tree,70001 trees,Results_1e-5._44sp_1.5.mcl.out.fasta_run_3.trees.tree,70001 trees,Results_1e-5._44sp_1.5.mcl.out.fasta_run_4.trees.tree,70001 trees,0.0111284,0.000176604,734,762,754,775,807,776,70001,1264,1264,70001,0.0078154,0.00961434,

Ortho,Aco,Non,Non,44,Results_1e-5._44sp_2.mcl.out.fasta,1.00E-05,2,43099,Results_1e-5._44sp_2.mcl.out.fasta_run_1.trees.tree,70001 trees,Results_1e-5._44sp_2.mcl.out.fasta_run_2.trees.tree,70001 trees,Results_1e-5._44sp_2.mcl.out.fasta_run_3.trees.tree,70001 trees,Results_1e-5._44sp_2.mcl.out.fasta_run_4.trees.tree,70001 trees,0.0258853,0.000225301,790,1011,998,1012,1051,1013,70001,1233,1233,70001,0.0153929,0.0315451,0.0307

Ortho,Aco,Non,Non,44,Results_1e-5._44sp_2.5.mcl.out.fasta,1.00E-05,2.5,47728,Results_1e-5._44sp_2.5.mcl.out.fasta_run_1.trees.tree,70001 trees,Results_1e-5._44sp_2.5.mcl.out.fasta_run_2.trees.tree,70001 trees,Results_1e-5._44sp_2.5.mcl.out.fasta_run_3.trees.tree,70001 trees,Results_1e-5._44sp_2.5.mcl.out.fasta_run_4.trees.tree,70001 trees,0.033171,0.000325559,807,1351,1342,1426,1526,1426,70001,1474,1474,70001,0.00358836,0.010

Ortho,Aco,Non,Non,44,Results_1e-5._44sp_4.mcl.out.fasta,1.00E-05,4,56208,Results_1e-5._44sp_4.mcl.out.fasta_run_1.trees.tree,70001 trees,Results_1e-5._44sp_4.mcl.out.fasta_run_2.trees.tree,70001 trees,Results_1e-5._44sp_4.mcl.out.fasta_run_3.trees.tree,70001 trees,Results_1e-5._44sp_4.mcl.out.fasta_run_4.trees.tree,70001 trees,0.0155998,0.00017349,924,15521,15283,17981,20142,18000,70001,1103,1103,70001,0.0118517,0.00551646,

Ortho,Aco,Non,Non,44,Results_1e-5._44sp_6.mcl.out.fasta,1.00E-05,6,61880,Results_1e-5._44sp_6.mcl.out.fasta_run_1.trees.tree,70001 trees,Results_1e-5._44sp_6.mcl.out.fasta_run_2.trees.tree,70001 trees,Results_1e-5._44sp_6.mcl.out.fasta_run_3.trees.tree,70001 trees,Results_1e-5._44sp_6.mcl.out.fasta_run_4.trees.tree,70001 trees,0.0239997,0.000219223,807,10482,10703,14397,20946,14239,70001,3046,3046,70001,0.00572558,0.013402

Ortho,Aco,Non,Non,44,Results_1e-9._47sp_1.5.mcl.out.fasta,1.00E-02,1.5,36269,../output/Results_1e-2._44sp_1.5_run_1.trees.tree,../output/Results_1e-2._44sp_1.5_run_2.trees.tree,../output/Results_1e-2._44sp_1.5_run_3.trees.tree,../output/Results_1e-2._44sp_1.5_run_4.trees.tree,,,,,0.244236,0.00243035,424,363,359,343,341,344,52747,926,926,52747,0.0020879,0.0337135,0.0337164,0.0342233,0.0342437,0.0342254,0,0.0151074,926,0,,341,"(A

Ortho,Aco,Non,Non,44,Results_1e-2._44sp_2,1.00E-02,2,45570,../../output/Results_1e-2._44sp_2_run_1.trees.tree : 38107 trees,../../output/Results_1e-2._44sp_2_run_2.trees.tree : 48661 trees,../../output/Results_1e-2._44sp_2_run_3.trees.tree : 48550 trees,../../output/Results_1e-2._44sp_2_run_4.trees.tree : 48541 trees,,,,,0.0143203,0.000199314,375,394,379,339,323,341,38129,1334,1334,38129,0.000459183,0.00555813,0.00633104,0.00868728,

Ortho,Aco,Non,Non,44,Results_1e-2._44sp_2.5,1.00E-02,2.5,50824,../output/Results_1e-2._44sp_2.5_run_1.trees.tree,../output/Results_1e-2._44sp_2.5_run_2.trees.tree,../output/Results_1e-2._44sp_2.5_run_3.trees.tree,../output/Results_1e-2._44sp_2.5_run_4.trees.tree,,,,,0.0894013,0.00074558,392,456,452,468,492,469,39478,529,529,39478,0.00753442,0.0248179,0.0246369,0.0230759,0.0237212,0.023137,0,0.0238062,529,0,,392,"(ACDI:0.0027

Ortho,Aco,Non,Non,44,Results_1e-2._44sp_4,1.00E-02,4,60021,../output/Results_1e-2._44sp_4_run_1.trees.tree,../output/Results_1e-2._44sp_4_run_2.trees.tree,../output/Results_1e-2._44sp_4_run_3.trees.tree,../output/Results_1e-2._44sp_4_run_4.trees.tree,,,,,0.00529689,0.000176809,384,972,964,1057,1190,1056,36562,1085,1085,36562,0.0128463,0.0258479,0.0258715,0.0260275,0.0259704,0.0260383,0,0.0191064,1085,0,,384,"(ACDI:0.00331

Ortho,Aco,Non,Non,44,Results_1e-2._44sp_6,1.00E-02,6,66075,../output/Results_1e-2._44sp_6_run_1.trees.tree,../output/Results_1e-2._44sp_6_run_2.trees.tree,../output/Results_1e-2._44sp_6_run_3.trees.tree,../output/Results_1e-2._44sp_6_run_4.trees.tree,,,,,0.00393596,0.000111131,462,6997,7003,7868,7290,7888,39393,1484,1484,39393,0.00781052,0.0183667,0.0184637,0.0185937,0.0185107,0.0185838,0,0.00985529,1484,0,,462,"(ACDI:0.00

Ortho,Opi,Non,Non,47,Results_1e-12._47sp_1.5.mcl.out.fasta,1.00E-12,1.5,34864,Results_1e-12._47sp_1.5.mcl.out.fasta_run_1.trees.tree,60001 trees,Results_1e-12._47sp_1.5.mcl.out.fasta_run_2.trees.tree,60001 trees,Results_1e-12._47sp_1.5.mcl.out.fasta_run_3.trees.tree,60001 trees,Results_1e-12._47sp_1.5.mcl.out.fasta_run_4.trees.tree,60001 trees,0.0861986,0.000614093,750,486,482,491,509,492,60001,1088,1088,60001,0.00408631,0.029

Ortho,Opi,Non,Non,47,Results_1e-12._47sp_2.5.mcl.out.fasta,1.00E-12,2,42034,Results_1e-12._47sp_2.mcl.out.fasta_run_1.trees.tree,60001 trees,Results_1e-12._47sp_2.mcl.out.fasta_run_2.trees.tree,60001 trees,Results_1e-12._47sp_2.mcl.out.fasta_run_3.trees.tree,60001 trees,Results_1e-12._47sp_2.mcl.out.fasta_run_4.trees.tree,60001 trees,0.0468492,0.00043945,722,521,515,524,544,524,60001,833,833,60001,0.0122459,0.0209681,0.02081

Ortho,Opi,Non,Non,47,Results_1e-12._47sp_2.mcl.out.fasta,1.00E-12,2.5,46272,Results_1e-12._47sp_2.5.mcl.out.fasta_run_1.trees.tree,60001 trees,Results_1e-12._47sp_2.5.mcl.out.fasta_run_2.trees.tree,60001 trees,Results_1e-12._47sp_2.5.mcl.out.fasta_run_3.trees.tree,60001 trees,Results_1e-12._47sp_2.5.mcl.out.fasta_run_4.trees.tree,60001 trees,0.0037166,0.000171268,675,466,464,469,481,469,60001,1435,1435,60001,0.00954254,0.0153

Ortho,Opi,Non,Non,47,Results_1e-12._47sp_4.mcl.out.fasta,1.00E-12,4,54954,Results_1e-12._47sp_4.mcl.out.fasta_run_1.trees.tree,58758 trees,Results_1e-12._47sp_4.mcl.out.fasta_run_2.trees.tree,47857 trees,Results_1e-12._47sp_4.mcl.out.fasta_run_3.trees.tree,59084 trees,Results_1e-12._47sp_4.mcl.out.fasta_run_4.trees.tree,54699 trees,0.0113757,0.000225169,490,920,923,1039,1204,1035,47937,1043,1043,47937,0.0111212,0.0218603,0.0

Ortho,Opi,Non,Non,47,Results_1e-12._47sp_6.mcl.out.fasta,1.00E-12,6,61366,Results_1e-12._47sp_6.mcl.out.fasta_run_1.trees.tree,70001 trees,Results_1e-12._47sp_6.mcl.out.fasta_run_2.trees.tree,70001 trees,Results_1e-12._47sp_6.mcl.out.fasta_run_3.trees.tree,70001 trees,Results_1e-12._47sp_6.mcl.out.fasta_run_4.trees.tree,70001 trees,0.0126855,0.000253753,688,1641,1672,2188,3164,2166,70001,1137,1137,70001,0.01139,0.0358215,0.0

Ortho,Opi,Non,Non,47,Results_1e-9._47sp_1.5.mcl.out.fasta,1.00E-09,1.5,36269,Results_1e-9._47sp_1.5.mcl.out.fasta_run_1.trees.tree,60001 trees,Results_1e-9._47sp_1.5.mcl.out.fasta_run_2.trees.tree,60001 trees,Results_1e-9._47sp_1.5.mcl.out.fasta_run_3.trees.tree,60001 trees,Results_1e-9._47sp_1.5.mcl.out.fasta_run_4.trees.tree,60001 trees,0.151781,0.000897764,724,429,425,415,419,416,60001,878,878,60001,0.00573719,0.0167208,0.01

Ortho,Opi,Non,Non,47,Results_1e-9._47sp_2.5.mcl.out.fasta,1.00E-09,2,44448,Results_1e-9._47sp_2.mcl.out.fasta_run_1.trees.tree,Results_1e-9._47sp_2.mcl.out.fasta_run_2.trees.tree,Results_1e-9._47sp_2.mcl.out.fasta_run_3.trees.tree,Results_1e-9._47sp_2.mcl.out.fasta_run_4.trees.tree,,,,,0.125998,0.00094235,716,596,590,619,656,620,60001,1195,1195,60001,0.010287,0.0235478,0.0233774,0.0202729,0.0175936,0.0203556,0,0.00646697,0.0

Ortho,Opi,Non,Non,47,Results_1e-9._47sp_2.mcl.out.fasta,1.00E-09,2.5,49447,output/Results_1e-9._47sp_2.5.mcl.out.fasta_run_1.trees.tree,60001 trees,output/Results_1e-9._47sp_2.5.mcl.out.fasta_run_2.trees.tree,60001 trees,output/Results_1e-9._47sp_2.5.mcl.out.fasta_run_3.trees.tree,60001 trees,output/Results_1e-9._47sp_2.5.mcl.out.fasta_run_4.trees.tree,60001 trees,0.0158331,0.000197125,580,473,468,461,465,462,60001,1206,1206,6000

Ortho,Opi,Non,Non,47,Results_1e-9._47sp_4.mcl.out.fasta,1.00E-09,4,58854,Results_1e-9._47sp_4.mcl.out.fasta_run_1.trees.tree,60001 trees,Results_1e-9._47sp_4.mcl.out.fasta_run_2.trees.tree,60001 trees,Results_1e-9._47sp_4.mcl.out.fasta_run_3.trees.tree,60001 trees,Results_1e-9._47sp_4.mcl.out.fasta_run_4.trees.tree,60001 trees,0.134231,0.00118906,529,470,468,476,491,476,60001,922,922,60001,0.00961862,0.0102306,0.0103252,0.011

Ortho,Opi,Non,Non,47,Results_1e-9._47sp_6.mcl.out.fasta,1.00E-09,6,65725,Results_1e-9._47sp_6.mcl.out.fasta_run_1.trees.tree,60001 trees,Results_1e-9._47sp_6.mcl.out.fasta_run_2.trees.tree,60001 trees,Results_1e-9._47sp_6.mcl.out.fasta_run_3.trees.tree,60001 trees,,,0.0562324,0.000734336,453,376,376,382,393,381,60001,1694,1694,60001,0.00650365,0.0065416,0.00628601,0.00612754,0.00650223,0.00610985,0,0.00639193,0.00639069

Ortho,Opi,Non,Non,47,Results_1e-5._47sp_1.5_1.mcl.out.fasta,1.00E-05,1.5,38234,Results_1e-5._47sp_1.5_1.mcl.out.fasta_run_1.trees.tree,60001 trees,Results_1e-5._47sp_1.5_1.mcl.out.fasta_run_2.trees.tree,60001 trees,Results_1e-5._47sp_1.5_1.mcl.out.fasta_run_3.trees.tree,60001 trees,Results_1e-5._47sp_1.5_1.mcl.out.fasta_run_4.trees.tree,60001 trees,0.0082832,0.000280686,509,337,334,324,323,324,60001,880,880,60001,0.0156459,0.0

Ortho,Opi,Non,Non,47,Results_1e-5._47sp_2.5.mcl.out.fasta,1.00E-05,2,48036,../../output/Results_1e-5._44sp_2.mcl.out.fasta_run_1.trees.tree,../../output/Results_1e-5._44sp_2.mcl.out.fasta_run_2.trees.tree,/output/Results_1e-5._44sp_2.mcl.out.fasta_run_3.trees.tree,/output/Results_1e-5._44sp_2.mcl.out.fasta_run_4.trees.tree,,,,,0.0258853,0.000225301,790,1011,998,1012,1051,1013,70001,1233,1233,70001,0.0153929,0.0315451,0.0307753,0.026

Ortho,Opi,Non,Non,47,Results_1e-5._47sp_2.mcl.out.fasta,1.00E-05,2.5,54142,output/Results_1e-5._47sp_2.5.mcl.out.fasta_run_1.trees.tree,60001 trees,output/Results_1e-5._47sp_2.5.mcl.out.fasta_run_4.trees.tree,60001 trees,output/Results_1e-5._47sp_2.5.mcl.out.fasta_run_3.trees.tree,60001 trees,output/Results_1e-5._47sp_2.5.mcl.out.fasta_run_2.trees.tree,60001 trees,0.197597,0.00145024,443,449,446,451,466,451,60001,605,605,60001,0.0

Ortho,Opi,Non,Non,47,Results_1e-5._47sp_4.mcl.out.fasta,1.00E-05,4,65212,Results_1e-5._47sp_4.mcl.out.fasta_run_1.trees.tree,60001 trees,Results_1e-5._47sp_4.mcl.out.fasta_run_2.trees.tree,60001 trees,Results_1e-5._47sp_4.mcl.out.fasta_run_3.trees.tree,60001 trees,Results_1e-5._47sp_4.mcl.out.fasta_run_4.trees.tree,60001 trees,0.00428326,0.000214123,425,373,372,380,395,380,60001,845,845,60001,0.00634129,0.0157474,0.0155447,0

Ortho,Opi,Non,Non,47,Results_1e-5._47sp_6.mcl.out.fasta,1.00E-05,6,72637,Results_1e-5._47sp_6.mcl.out.fasta_run_1.trees.tree,76060 trees,Results_1e-5._47sp_6.mcl.out.fasta_run_2.trees.tree,71735 trees,Results_1e-5._47sp_6.mcl.out.fasta_run_3.trees.tree,76445 trees,Results_1e-5._47sp_6.mcl.out.fasta_run_4.trees.tree,70891 trees,0.00677195,0.000159919,465,348,347,365,399,364,70891,879,879,70891,0.00587602,0.0237804,0.023685,0.0

Ortho,Opi,Non,Non,47,Results_1e-2._47sp_1.5_1.mcl.out.fasta,1.00E-02,1.5,40216,Results_1e-2._47sp_1.5_1.mcl.out.fasta_run_1.trees.tree,8001 trees,Results_1e-2._47sp_1.5_1.mcl.out.fasta_run_2.trees.tree,6994 trees,Results_1e-2._47sp_1.5_1.mcl.out.fasta_run_3.trees.tree,7002 trees,Results_1e-2._47sp_1.5_1.mcl.out.fasta_run_4.trees.tree,6974 trees,0.039887,0.000478329,416,324,322,310,309,311,6976,1566,1566,6976,0.00596492,0.01568

Ortho,Opi,Non,Non,47,Results_1e-2._47sp_2.5.mcl.out.fasta,1.00E-02,2,51418,Results_1e-2._47sp_2.mcl.out.fasta_run_1.trees.tree : 60001 trees,Results_1e-2._47sp_2.mcl.out.fasta_run_2.trees.tree : 60001 trees,Results_1e-2._47sp_2.mcl.out.fasta_run_3.trees.tree : 60001 trees,Results_1e-2._47sp_2.mcl.out.fasta_run_4.trees.tree : 60001 trees,,,,,0.019183,0.000450799,356,328,326,334,345,334,60001,474,474,60001,0.00519087,0.00374447,0.00

Ortho,Opi,Non,Non,47,Results_1e-2._47sp_2.mcl.out.fasta,1.00E-02,2.5,58248,Results_1e-2._47sp_2.5.mcl.out.fasta_run_1.trees.tree,70001 trees,Results_1e-2._47sp_2.5.mcl.out.fasta_run_2.trees.tree,70001 trees,Results_1e-2._47sp_2.5.mcl.out.fasta_run_3.trees.tree,70001 trees,Results_1e-2._47sp_2.5.mcl.out.fasta_run_4.trees.tree,70001 trees,0.047485,0.000567011,341,274,273,274,279,274,70001,207,207,70001,0.00971725,0.0124057,0.012

Ortho,Opi,Non,Non,47,Results_1e-2._47sp_4.mcl.out.fasta,1.00E-02,4,70371,Results_1e-2._47sp_4.mcl.out.fasta_run_1.trees.tree,70001 trees,Results_1e-2._47sp_4.mcl.out.fasta_run_2.trees.tree,70001 trees,Results_1e-2._47sp_4.mcl.out.fasta_run_3.trees.tree,70001 trees,Results_1e-2._47sp_4.mcl.out.fasta_run_4.trees.tree,70001 trees,0.0179283,0.000363992,299,314,313,323,341,323,70001,472,472,70001,0.00361237,0.0343321,0.0332068,0.0

Homo,Opi,Non,Non,47,Diamond_47sp_Opi_1e-2_Results_allVSall.txt.abc.6.mcl.out.fasta,1.00E-02,6,58664,out.Diamond_47sp_Opi_1e-2_Results_allVSall.txt.abc.I60_run_1.trees.tree,70001 trees,out.Diamond_47sp_Opi_1e-2_Results_allVSall.txt.abc.I60_run_2.trees.tree,70001 trees,out.Diamond_47sp_Opi_1e-2_Results_allVSall.txt.abc.I60_run_3.trees.tree,70001 trees,out.Diamond_47sp_Opi_1e-2_Results_allVSall.txt.abc.I60_run_4.trees.tree,70001

Ortho,Opi,Non,Non,47,Results_1e-2._47sp_6.mcl.out.fasta,1.00E-02,6,78371,Results_1e-2._47sp_6.mcl.out.fasta_run_1.trees.tree,70001 trees,Results_1e-2._47sp_6.mcl.out.fasta_run_2.trees.tree,70001 trees,Results_1e-2._47sp_6.mcl.out.fasta_run_3.trees.tree,70001 trees,Results_1e-2._47sp_6.mcl.out.fasta_run_4.trees.tree,70001 trees,0.0069999,0.000175378,212,170,169,168,170,169,70001,366,366,70001,0.00379123,0.000416962,0.00034230

,,SUM,,,30,20,7,6,1,2,7,8,1,1,1,6,7,1,7,4,1,4,3,2,7,15,5,9,4,2,8,1,6,3,4,6,1

,,%SUM,,,15.79,10.53,3.68,3.16,0.53,1.05,3.68,4.21,0.53,0.53,0.53,3.16,3.68,0.53,3.68,2.11,0.53,2.11,1.58,1.05,3.68,7.89,2.63,4.74,2.11,1.05,4.21,0.53,3.16,1.58,2.11,3.16,0.53

,,Porifera sister ,,,169,,,,,,,169,,169,,,,,,,,169,,,,,,,,,,,,

,,% Porifera sister ,,,88.9,11.1,,,,,,88.9,,88.9,,,,,,,,88.9,,,,,,,,,,,,

,,From PS CT Second (No Coelenterata),,,93,,,,,,Non resolved,3,CT-PL Sister,21,,,,,,,"PL first, before CT",52,,,,,,,,,,,,

,,% From PS CT Second (No Coelenterata),,,55.0,,,,,,,1.8,,12.4,,,,,,,,30.8,,,,,,,,,,,,

,,From PS - Nephrozoa,,,122.0,,,,,,,,,,,,,,,,,,,,,,,,,,,,,,,,

,,% From PS - Nephrozoa,,,72.2,,,,,,,,,,,,,,,,,,,,,,,,,,,,,,,,

,,Nephrozoa from CT Second,,,81.0,,,,,,,,,,,,,,,,,,,,,,,,,,,,,,,,

,,% Nephrozoa from CT Second,,,87.1,,,,,,,,,,,,,,,,,,,,,,,,,,,,,,,,

,,From PS - D. Monopyletic,,,145,,,,,,,,,,,,,,,,,,,,,,,,,,,,,,,,

,,% From PS - D. Monopyletic,,,85.8,,,,,,,,,,,,,,,,,,,,,,,,,,,,,,,,

,,From CT Second Nephrozoa - D. Monopyletic,,,73,,,,,,,,,,,,,,,,,,,,,,,,,,,,,,,,

,,% From CT Second Nephrozoa - D. Monopyletic,,,90.1,,,,,,,,,,,,,,,,,,,,,,,,,,,,,,,,

,,,

,,SUM Homo,,,8,1,1,0,0,1,0,2,1,1,0,4,7,1,7,0,0,0,1,0,7,10,5,6,4,0,8,1,5,3,4,5,1

,,%SUM Homo,,,8.42,1.05,1.05,0.00,0.00,1.05,0.00,2.11,1.05,1.05,0.00,4.21,7.37,1.05,7.37,0.00,0.00,0.00,1.05,0.00,7.37,10.53,5.26,6.32,4.21,0.00,8.42,1.05,5.26,3.16,4.21,5.26,1.05

,,Porifera sister ,,,75,,,,,,,75,,75,,,,,,,,75,,,,,,,,,,,,

,,% Porifera sister ,,,78.9,,,,,,,78.9,,78.9,,,,,,,,78.9,,,,,,,,,,,,

,,From PS CT Second (No Coelenterata),,,14,,,,,,Non resolved,2,CT-PL Sister,19,,,,,,,"PL first, before CT",40,,,,,,,,,,,,

,,% From PS CT Second (No Coelenterata),,,18.7,,,,,,,2.1,,20.0,,,,,,,,42.1,,,,,,,,,,,,

,,From PS - Nephrozoa,,,48.0,,,,,,,,,,,,,,,,,,,,,,,,,,,,,,,,

,,% From PS - Nephrozoa,,,64.0,,,,,,,,,,,,,,,,,,,,,,,,,,,,,,,,

,,Nephrozoa from CT Second,,,13.0,,,,,,,,,,,,,,,,,,,,,,,,,,,,,,,,

,,% Nephrozoa from CT Second,,,92.9,,,,,,,,,,,,,,,,,,,,,,,,,,,,,,,,

,,From PS - D. Monopyletic,,,74,,,,,,,,,,,,,,,,,,,,,,,,,,,,,,,,

,,% From PS - D. Monopyletic,,,98.7,,,,,,,,,,,,,,,,,,,,,,,,,,,,,,,,

,,From CT Second Nephrozoa - D. Monopyletic,,,13,,,,,,,,,,,,,,,,,,,,,,,,,,,,,,,,

,,% From CT Second Nephrozoa - D. Monopyletic,,,100.0,,,,,,,,,,,,,,,,,,,,,,,,,,,,,,,,

,,,

,,SUM Ortho,,,22,19,6,6,1,1,7,6,0,0,1,2,0,0,0,4,1,4,2,2,0,5,0,3,0,2,0,0,1,0,0,1,0

,,%SUM Ortho,,,23.16,20.00,6.32,6.32,1.05,1.05,7.37,6.32,0.00,0.00,1.05,2.11,0.00,0.00,0.00,4.21,1.05,4.21,2.11,2.11,0.00,5.26,0.00,3.16,0.00,2.11,0.00,0.00,1.05,0.00,0.00,1.05,0.00

,,Porifera sister ,,,94,,,,,,,94,,94,,,,,,,,40,,,,,,,,,,,,

,,% Porifera sister ,,,98.9,,,,,,,98.9,,98.9,,,,,,,,42.1,,,,,,,,,,,,

,,From PS CT Second (No Coelenterata),,,79,,,,,,Non resolved,1,CT-PL Sister,2,,,,,,,"PL first, before CT",12,,,,,,,,,,,,

,,% From PS CT Second (No Coelenterata),,,84.0,,,,,,,1.1,,2.1,,,,,,,,12.6,,,,,,,,,,,,

,,From PS - Nephrozoa,,,74.0,,,,,,,,,,,,,,,,,,,,,,,,,,,,,,,,

,,% From PS - Nephrozoa,,,78.7,,,,,,,,,,,,,,,,,,,,,,,,,,,,,,,,

,,Nephrozoa from CT Second,,,68.0,,,,,,,,,,,,,,,,,,,,,,,,,,,,,,,,

,,% Nephrozoa from CT Second,,,86.1,,,,,,,,,,,,,,,,,,,,,,,,,,,,,,,,

,,From PS - D. Monopyletic,,,71,,,,,,,,,,,,,,,,,,,,,,,,,,,,,,,,

,,% From PS - D. Monopyletic,,,75.5,,,,,,,,,,,,,,,,,,,,,,,,,,,,,,,,

,,From CT Second Nephrozoa - D. Monopyletic,,,60,,,,,,,,,,,,,,,,,,,,,,,,,,,,,,,,

,,% From CT Second Nephrozoa - D. Monopyletic,,,88.2,,,,,,,,,,,,,,,,,,,,,,,,,,,,,,,,

,,,

,,Sum E-val Low,,,18,8,0,0,0,0,6,5,0,0,0,4,4,0,5,0,0,0,0,1,0,1,0,0,0,0,0,1,2,1,2,3,0

,,%SUM E-val Low,,,30.00,13.33,0.00,0.00,0.00,0.00,10.00,8.33,0.00,0.00,0.00,6.67,6.67,0.00,8.33,0.00,0.00,0.00,0.00,1.67,0.00,1.67,0.00,0.00,0.00,0.00,0.00,1.67,3.33,1.67,3.33,5.00,0.00

,,Porifera sister ,,,52,,,,,,,,,,,,,,,,,,,,,,,,,,,,,,,,

,,% Porifera sister ,,,86.7,,,,,,,,,,,,,,,,,,,,,,,,,,,,,,,,

,,From PS CT Second (No Coelenterata),,,37,,,,,,,,,,,,,,,,,,,,,,,,,,,,,,,,

,,% From PS CT Second (No Coelenterata),,,71.2,,,,,,,,,,,,,,,,,,,,,,,,,,,,,,,,

,,From PS - Nephrozoa,,,46.0,,,,,,,,,,,,,,,,,,,,,,,,,,,,,,,,

,,% From PS - Nephrozoa,,88.5,,,,,,,,,,,,,,,,,,,,,,,,,,,,,,
,,Nephrozoa from CT Second,,37.0,,,,,,,,,,,,,,,,,,,,,,,,,,,,,,
,,% Nephrozoa from CT Second,,100.0,,,,,,,,,,,,,,,,,,,,,,,,,,,,,,
,,From PS - D. Monopyletic,,45,,,,,,,,,,,,,,,,,,,,,,,,,,,,,,
,,% From PS - D. Monopyletic,,86.5,,,,,,,,,,,,,,,,,,,,,,,,,,,,,,
,,From CT Second Nephrozoa - D. Monopyletic,,31,,,,,,,,,,,,,,,,,,,,,,,,,,,,,,
,,% From CT Second Nephrozoa - D. Monopyletic,,83.8,,,,,,,,,,,,,,,,,,,,,,,,,,,,,,
,,
,,
,,Sum E-val High,,6,6,0,0,0,0,1,2,1,0,1,2,3,0,2,0,1,0,3,1,0,7,0,5,0,2,3,0,4,2,2,3,1
,,%SUM E-val High,,10.00,,,,,,,,,,,,,,,,,,,,,,,,,,,,,,
,,Porifera sister ,,46,,,,,,,,,,,,,,,,,,,,,,,,,,,,,,
,,% Porifera sister ,,76.7,,,,,,,,,,,,,,,,,,,,,,,,,,,,,,
,,From PS CT Second (No Coelenterata),,19,,,,,,,,,,,,,,,,,,,,,,,,,,,,,,
,,% From PS CT Second (No Coelenterata),,41.3,,,,,,,,,,,,,,,,,,,,,,,,,,,,,,
,,From PS - Nephrozoa,,32.0,,,,,,,,,,,,,,,,,,,,,,,,,,,,,,
,,% From PS - Nephrozoa,,69.6,,,,,,,,,,,,,,,,,,,,,,,,,,,,,,
,,Nephrozoa from CT Second,,15.0,,,,,,,,,,,,,,,,,,,,,,,,,,,,,,
,,% Nephrozoa from CT Second,,78.9,,,,,,,,,,,,,,,,,,,,,,,,,,,,,,
,,From PS - D. Monopyletic,,38,,,,,,,,,,,,,,,,,,,,,,,,,,,,,,
,,% From PS - D. Monopyletic,,82.6,,,,,,,,,,,,,,,,,,,,,,,,,,,,,,
,,From CT Second Nephrozoa - D. Monopyletic,,14,,,,,,,,,,,,,,,,,,,,,,,,,,,,,,
,,% From CT Second Nephrozoa - D. Monopyletic,,93.3,,,,,,,,,,,,,,,,,,,,,,,,,,,,,,
,,
,,Sum I val Low,,17,11,0,0,0,0,4,7,1,0,1,5,7,0,6,0,0,0,0,0,0,5,0,2,0,2,3,0,0,0,0,0,1
,,%SUM I val Low,,23.61,,,,,,,,,,,,,,,,,,,,,,,,,,,,,,
,,Porifera sister ,,71,,,,,,,,,,,,,,,,,,,,,,,,,,,,,,
,,% Porifera sister ,,98.6,,,,,,,,,,,,,,,,,,,,,,,,,,,,,,
,,From PS CT Second (No Coelenterata),,39,,,,,,,,,,,,,,,,,,,,,,,,,,,,,,
,,% From PS CT Second (No Coelenterata),,54.9,,,,,,,,,,,,,,,,,,,,,,,,,,,,,,
,,From PS - Nephrozoa,,61.0,,,,,,,,,,,,,,,,,,,,,,,,,,,,,,
,,% From PS - Nephrozoa,,85.9,,,,,,,,,,,,,,,,,,,,,,,,,,,,,,
,,Nephrozoa from CT Second,,39.0,,,,,,,,,,,,,,,,,,,,,,,,,,,,,,
,,% Nephrozoa from CT Second,,100.0,,,,,,,,,,,,,,,,,,,,,,,,,,,,,,
,,From PS - D. Monopyletic,,65,,,,,,,,,,,,,,,,,,,,,,,,,,,,,,
,,% From PS - D. Monopyletic,,91.5,,,,,,,,,,,,,,,,,,,,,,,,,,,,,,
,,From CT Second Nephrozoa - D. Monopyletic,,35,,,,,,,,,,,,,,,,,,,,,,,,,,,,,,
,,% From CT Second Nephrozoa - D. Monopyletic,,89.7,,,,,,,,,,,,,,,,,,,,,,,,,,,,,,
,,
,,
,,
,,Sum I val High,,7,3,0,0,0,0,3,0,0,0,0,1,0,0,1,0,1,0,3,2,0,4,0,3,0,0,0,1,6,3,4,6,0
,,%SUM I val High,,14.58,,,,,,,,,,,,,,,,,,,,,,,,,,,,,,
,,Porifera sister ,,28,,,,,,,,,,,,,,,,,,,,,,,,,,,,,,
,,% Porifera sister ,,58.3,,,,,,,,,,,,,,,,,,,,,,,,,,,,,,
,,From PS CT Second (No Coelenterata),,17,,,,,,,,,,,,,,,,,,,,,,,,,,,,,,
,,% From PS CT Second (No Coelenterata),,60.7,,,,,,,,,,,,,,,,,,,,,,,,,,,,,,
,,From PS - Nephrozoa,,17.0,,,,,,,,,,,,,,,,,,,,,,,,,,,,,,
,,% From PS - Nephrozoa,,60.7,,,,,,,,,,,,,,,,,,,,,,,,,,,,,,
,,Nephrozoa from CT Second,,13.0,,,,,,,,,,,,,,,,,,,,,,,,,,,,,,
,,% Nephrozoa from CT Second,,76.5,,,,,,,,,,,,,,,,,,,,,,,,,,,,,,
,,From PS - D. Monopyletic,,19,,,,,,,,,,,,,,,,,,,,,,,,,,,,,,
,,% From PS - D. Monopyletic,,67.9,,,,,,,,,,,,,,,,,,,,,,,,,,,,,,
,,From CT Second Nephrozoa - D. Monopyletic,,10,,,,,,,,,,,,,,,,,,,,,,,,,,,,,,
,,% From CT Second Nephrozoa - D. Monopyletic,,76.9,,,,,,,,,,,,,,,,,,,,,,,,,,,,,,
,,
,,Dis,,4,4,0,0,0,0,0,1,0,0,0,0,0,0,0,0,0,0,2,0,0,3,6,0,3,0,0,4,0,0,0,0,0,0
,,%SUM I val High,,14.81,,,,,,,,,,,,,,,,,,,,,,,,,,,,,,
,,Porifera sister ,,27,,,,,,,,,,,,,,,,,,,,,,,,,,,,,,
,,% Porifera sister ,,100.0,,,,,,,,,,,,,,,,,,,,,,,,,,,,,,
,,From PS CT Second (No Coelenterata),,11,,,,,,,,,,,,,,,,,,,,,,,,,,,,,,
,,% From PS CT Second (No Coelenterata),,40.7,,,,,,,,,,,,,,,,,,,,,,,,,,,,,,
,,From PS - Nephrozoa,,16.0,,,,,,,,,,,,,,,,,,,,,,,,,,,,,,
,,% From PS - Nephrozoa,,59.3,,,,,,,,,,,,,,,,,,,,,,,,,,,,,,
,,Nephrozoa from CT Second,,9.0,,,,,,,,,,,,,,,,,,,,,,,,,,,,,,
,,% Nephrozoa from CT Second,,81.8,,,,,,,,,,,,,,,,,,,,,,,,,,,,,,
,,From PS - D. Monopyletic,,25,,,,,,,,,,,,,,,,,,,,,,,,,,,,,,
,,% From PS - D. Monopyletic,,92.6,,,,,,,,,,,,,,,,,,,,,,,,,,,,,,
,,From CT Second Nephrozoa - D. Monopyletic,,9,,,,,,,,,,,,,,,,,,,,,,,,,,,,,,
,,% From CT Second Nephrozoa - D. Monopyletic,,100.0,,,,,,,,,,,,,,,,,,,,,,,,,,,,,,
,,
,,Ne,,1,1,7,6,1,2,0,0,0,1,0,0,0,1,0,4,0,0,0,0,4,0,5,1,4,0,0,0,0,0,0,0,0
,,%SUM I val High,,2.63,,,,,,,,,,,,,,,,,,,,,,,,,,,,,,
,,Porifera sister ,,38,,,,,,,,,,,,,,,,,,,,,,,,,,,,,,
,,% Porifera sister ,,100.0,,,,,,,,,,,,,,,,,,,,,,,,,,,,,,
,,From PS CT Second (No Coelenterata),,22,,,,,,,,,,,,,,,,,,,,,,,,,,,,,,
,,% From PS CT Second (No Coelenterata),,57.9,,,,,,,,,,,,,,,,,,,,,,,,,,,,,,
,,From PS - Nephrozoa,,25.0,,,,,,,,,,,,,,,,,,,,,,,,,,,,,,
,,% From PS - Nephrozoa,,65.8,,,,,,,,,,,,,,,,,,,,,,,,,,,,,,
,,Nephrozoa from CT Second,,18.0,,,,,,,,,,,,,,,,,,,,,,,,,,,,,,
,,% Nephrozoa from CT Second,,81.8,,,,,,,,,,,,,,,,,,,,,,,,,,,,,,
,,From PS - D. Monopyletic,,33,,,,,,,,,,,,,,,,,,,,,,,,,,,,,,

,,% From PS - D. Monopyletic,,,86.8,,,,,,,,,,,,,,,,,,,,,,,,,,,,,,
,,From CT Second Nephrozoa - D. Monopyletic,,,17,,,,,,,,,,,,,,,,,,,,,,,,,,,,,,
,,% From CT Second Nephrozoa - D. Monopyletic,,,94.4,,,,,,,,,,,,,,,,,,,,,,,,,,,,,,
,,,
,,Opi,,,2,2,3,2,1,2,0,1,0,1,0,0,0,1,0,0,0,0,0,0,3,2,0,1,0,0,2,0,0,0,0,0,0
,,%SUM Opi,,,8.70,,,,,,,8.70,,8.70,,,,,,,,8.70,,,,,,,,,,,,
,,Porifera sister ,,,23,,,,,,,10,,1,,,,,,,,8,,,,,,,,,,,,
,,% Porifera sister ,,,100.0,,,,,,,43.5,,4.3,,,,,,,,34.8,,,,,,,,,,,,
,,From PS CT Second (No Coelenterata),,,13,,,,,,,1,,1,,,,,,,,8,,,,,,,,,,,,
,,% From PS CT Second (No Coelenterata),,,56.5,,,,,,,4.3,,4.3,,,,,,,,34.8,,,,,,,,,,,,
,,From PS - Nephrozoa,,,18.0,,,,,,,,,,,,,,,,,,,,,,,,,,,,,,
,,% From PS - Nephrozoa,,,78.3,,,,,,,,,,,,,,,,,,,,,,,,,,,,,,
,,Nephrozoa from CT Second,,,13.0,,,,,,,,,,,,,,,,,,,,,,,,,,,,,,
,,% Nephrozoa from CT Second,,,100.0,,,,,,,,,,,,,,,,,,,,,,,,,,,,,,
,,From PS - D. Monopyletic,,,22,,,,,,,,,,,,,,,,,,,,,,,,,,,,,,
,,% From PS - D. Monopyletic,,,95.7,,,,,,,,,,,,,,,,,,,,,,,,,,,,,,
,,From CT Second Nephrozoa - D. Monopyletic,,,12,,,,,,,,,,,,,,,,,,,,,,,,,,,,,,
,,% From CT Second Nephrozoa - D. Monopyletic,,,92.3,,,,,,,,,,,,,,,,,,,,,,,,,,,,,,
,,,
,,Hol,,,2,2,2,2,0,0,0,0,0,0,0,0,0,0,0,2,0,2,0,0,2,2,3,1,2,0,2,0,0,0,0,0,0
,,%SUM Hol,,,8.33,,,,,,,8.33,,0.00,,,,,,,,8.33,,,,,,,,,,,,
,,Porifera sister ,,,24,,,,,,,16,,0,,,,,,,,12,,,,,,,,,,,,
,,% Porifera sister ,,,100.0,,,,,,,66.7,,0.0,,,,,,,,50.0,,,,,,,,,,,,
,,From PS CT Second (No Coelenterata),,,12,,,,,,,0,,0,,,,,,,,12,,,,,,,,,,,,
,,% From PS CT Second (No Coelenterata),,,50.0,,,,,,,0.0,,0.0,,,,,,,,50.0,,,,,,,,,,,,
,,From PS - Nephrozoa,,,13.0,,,,,,,,,,,,,,,,,,,,,,,,,,,,,,
,,% From PS - Nephrozoa,,,54.2,,,,,,,,,,,,,,,,,,,,,,,,,,,,,,
,,Nephrozoa from CT Second,,,8.0,,,,,,,,,,,,,,,,,,,,,,,,,,,,,,
,,% Nephrozoa from CT Second,,,66.7,,,,,,,,,,,,,,,,,,,,,,,,,,,,,,
,,From PS - D. Monopyletic,,,20,,,,,,,,,,,,,,,,,,,,,,,,,,,,,,
,,% From PS - D. Monopyletic,,,83.3,,,,,,,,,,,,,,,,,,,,,,,,,,,,,,
,,From CT Second Nephrozoa - D. Monopyletic,,,8,,,,,,,,,,,,,,,,,,,,,,,,,,,,,,
,,% From CT Second Nephrozoa - D. Monopyletic,,,100.0,,,,,,,,,,,,,,,,,,,,,,,,,,,,,,
,,,
,,Cho,,,2,2,2,2,0,0,0,0,0,0,0,0,0,0,0,2,0,2,0,0,2,2,2,2,2,0,2,0,0,0,0,0,0
,,%SUM Cho,,,8.33,,,,,,,8.33,,8.33,,,,,,,,8.33,,,,,,,,,,,,
,,Porifera sister ,,,24,,,,,,,16,,0,,,,,,,,12,,,,,,,,,,,,
,,% Porifera sister ,,,100.0,,,,,,,66.7,,0.0,,,,,,,,50.0,,,,,,,,,,,,
,,From PS CT Second (No Coelenterata),,,12,,,,,,,0,,0,,,,,,,,12,,,,,,,,,,,,
,,% From PS CT Second (No Coelenterata),,,50.0,,,,,,,0.0,,0.0,,,,,,,,50.0,,,,,,,,,,,,
,,From PS - Nephrozoa,,,14.0,,,,,,,,,,,,,,,,,,,,,,,,,,,,,,
,,% From PS - Nephrozoa,,,58.3,,,,,,,,,,,,,,,,,,,,,,,,,,,,,,
,,Nephrozoa from CT Second,,,8.0,,,,,,,,,,,,,,,,,,,,,,,,,,,,,,
,,% Nephrozoa from CT Second,,,66.7,,,,,,,,,,,,,,,,,,,,,,,,,,,,,,
,,From PS - D. Monopyletic,,,20,,,,,,,,,,,,,,,,,,,,,,,,,,,,,,
,,% From PS - D. Monopyletic,,,83.3,,,,,,,,,,,,,,,,,,,,,,,,,,,,,,
,,From CT Second Nephrozoa - D. Monopyletic,,,8,,,,,,,,,,,,,,,,,,,,,,,,,,,,,,
,,% From CT Second Nephrozoa - D. Monopyletic,,,100.0,,,,,,,,,,,,,,,,,,,,,,,,,,,,,,
,,,
,,Pruned,,,3,3,3,3,1,0,0,0,0,1,0,0,0,1,0,2,0,1,0,0,1,3,3,3,2,0,2,0,0,0,0,0,0
,,%SUM Pruned,,,9.38,,,,,,,,,,,,,,,,,,,,,,,,,,,,,,
,,Porifera sister ,,,32,,,,,,,,,,,,,,,,,,,,,,,,,,,,,,
,,% Porifera sister ,,,100.0,,,,,,,,,,,,,,,,,,,,,,,,,,,,,,
,,From PS CT Second (No Coelenterata),,,16,,,,,,,,,,,,,,,,,,,,,,,,,,,,,,
,,% From PS CT Second (No Coelenterata),,,50.0,,,,,,,,,,,,,,,,,,,,,,,,,,,,,,
,,From PS - Nephrozoa,,,22.0,,,,,,,,,,,,,,,,,,,,,,,,,,,,,,
,,% From PS - Nephrozoa,,,68.8,,,,,,,,,,,,,,,,,,,,,,,,,,,,,,
,,Nephrozoa from CT Second,,,13.0,,,,,,,,,,,,,,,,,,,,,,,,,,,,,,
,,% Nephrozoa from CT Second,,,81.3,,,,,,,,,,,,,,,,,,,,,,,,,,,,,,
,,From PS - D. Monopyletic,,,28,,,,,,,,,,,,,,,,,,,,,,,,,,,,,,
,,% From PS - D. Monopyletic,,,87.5,,,,,,,,,,,,,,,,,,,,,,,,,,,,,,
,,From CT Second Nephrozoa - D. Monopyletic,,,12,,,,,,,,,,,,,,,,,,,,,,,,,,,,,,
,,% From CT Second Nephrozoa - D. Monopyletic,,,92.3,,,,,,,,,,,,,,,,,,,,,,,,,,,,,,
,,,
,,Ab Initio,,,3,3,4,2,0,2,0,1,0,0,0,0,0,0,0,2,0,3,0,0,6,3,3,2,3,0,3,0,0,0,0,0,0
,,%SUM Ab initio,,,7.50,,,,,,,,,,,,,,,,,,,,,,,,,,,,,,
,,Porifera sister ,,,40,,,,,,,,,,,,,,,,,,,,,,,,,,,,,,
,,% Porifera sister ,,,100.0,,,,,,,,,,,,,,,,,,,,,,,,,,,,,,
,,From PS CT Second (No Coelenterata),,,20,,,,,,,,,,,,,,,,,,,,,,,,,,,,,,
,,% From PS CT Second (No Coelenterata),,,50.0,,,,,,,,,,,,,,,,,,,,,,,,,,,,,,
,,From PS - Nephrozoa,,,23.0,,,,,,,,,,,,,,,,,,,,,,,,,,,,,,
,,% From PS - Nephrozoa,,,57.5,,,,,,,,,,,,,,,,,,,,,,,,,,,,,,
,,Nephrozoa from CT Second,,,15.0,,,,,,,,,,,,,,,,,,,,,,,,,,,,,,
,,% Nephrozoa from CT Second,,,75.0,,,,,,,,,,,,,,,,,,,,,,,,,,,,,,
,,From PS - D. Monopyletic,,,35,,,,,,,,,,,,,,,,,,,,,,,,,,,,,,
,,% From PS - D. Monopyletic,,,87.5,,,,,,,,,,,,,,,,,,,,,,,,,,,,,,
,,From CT Second Nephrozoa - D. Monopyletic,,,15,,,,,,,,,,,,,,,,,,,,,,,,,,,,,,
,,% From CT Second Nephrozoa - D. Monopyletic,,,100.0,,,,,,,,,,,,,,,,,,,,,,,,,,,,,,
