# Supplementary material for: Exploring genome gene content and morphological analysis to test recalcitrant nodes in the animal phylogeny
Source: PLoS One. 2023 Mar 23;18(3):e0282444. doi: 10.1371/journal.pone.0282444 (PMC10035847; doi:10.1371/journal.pone.0282444)
Supplement: S4 Table — (PDF) [file pone.0282444.s018.pdf]

Supplementary Table 4 | Naming convention for LBA check, for additional information for the datasets see Supplementary Table 2.

**Long-Branch attraction tests**			**Ctenophora-sister vs Porifera-sister** (**Total n. of sp.**)	**Relationships of Xenacoelomorpha - taxon exclusion**	
**Outgroup Sapling**	**LBA effect from**	**Method**		**Xen**oturbella (**Total n. of sp.**)	**Aco**eloorpha (**Total n. of sp.**)
**Opi**sthokonta	**dis**tant outgroup	**Ab** initio	Opi-homo/ortho-disAb47	OpiXen-homo/ortho-disAb41	OpiAco-homo/ortho-disAb44
		**P**runing	-	OpiXen-homo/ortho-disP41	OpiAco-homo/ortho-disP44
	in-groups (**ne**ar group)	**Ab** initio	Opi-homo/ortho-neAb44	OpiXen-homo/ortho-neAb38	OpiAco-homo/ortho-neAb41
		**P**runing	Opi-homo/ortho-neP44	OpiXen-homo/ortho-neP38	OpiAco-homo/ortho-neP41
**Hol**ozoa	**dis**tant outgroup	**Ab** initio	Hol-homo/ortho-disAb41	HolXen-homo/ortho-disAb35	HolAco-homo/ortho-desAb38
		**P**runing	Hol-homo/ortho-disP41	HolXen-homo/ortho-disP35	HolAco-homo/ortho-disP38
	in-groups (**ne**ar group)	**Ab** initio	Hol-homo/ortho-neAb38	HolXen-homo/ortho-neAb32	HolAco-homo/ortho-neAb35
		**P**runing	Hol-homo/ortho-neP38	HolXen-homo/ortho-neP32	HolAco-homo/ortho-neP35
**Cho**anozoa	**dis**tant outgroup	**Ab** initio	Cho-homo/ortho-disAb39	ChoXen-homo/ortho-disAb33	ChoAco-homo/ortho-disAb36
		**P**runing	Cho-homo/ortho-disP39	ChoXen-homo/ortho-disP33	ChoAco-homo/ortho-disP36
	in-groups (**ne**ar group)	**Ab** initio	Cho-homo/ortho-neAb36	ChoXen-homo/ortho-neAb30	ChoAco-homo/ortho-neAb33
		**P**runing	Cho-homo/ortho-neP36	ChoXen-homo/ortho-neP30	ChoAco-homo/ortho-neP33
