# Supplementary material for: Exploring genome gene content and morphological analysis to test recalcitrant nodes in the animal phylogeny
Source: PLoS One. 2023 Mar 23;18(3):e0282444. doi: 10.1371/journal.pone.0282444 (PMC10035847; doi:10.1371/journal.pone.0282444)
Supplement: S5 Table — (PDF) [file pone.0282444.s019.pdf]

Table S5 | Statistical hypothesis testing calculation results ,,,,,,,,,,,,,,,,,,,,,,,,,,,,,,
Data type,Species number,Dataset name,method,I_val,E_val,rep,n_samples,pp_Porifera,pp_Ctenophora,pp_Cnidaria,pp_Xenacoelomorpha,pp_Hemichordata,pp_Echinodermata,pp_Lophotrochozoa,pp_Ecdysozoa,pp_metazoans,pp_metazoans_ingroup,pp_Ctenophora_Sister,pp_Porifera_Sister,pp_Deuterostome,pp_Nephrozoa,pp_Xenambulacraria,bf_Porifera,bf_Xenacoelomorpha,bf_Deutorostome,bf_Porifera>5,bf_Porifera<-5,bf_Xenacoelomorpha>
Gene content,41,Xen,Homogroups,1.5,1.00E-02,1,70001,1,1,1,1,1,1,0.002247576,1,1,0.8567075,0,1,1,1,0,11.15625052,11.15625052,11.15625052,,,0,1,0,,
Gene content,41,Xen,Homogroups,1.5,1.00E-02,2,70001,1,1,1,1,1,1,0.001809489,1,1,0.8553932,0,1,1,1,0,11.15625052,11.15625052,11.15625052,,,0,1,0,,
Gene content,41,Xen,Homogroups,1.5,1.00E-02,3,70001,1,1,1,1,1,1,7.62E-05,1,1,0.8534123,0,1,1,1,0,11.15625052,11.15625052,11.15625052,,,0,1,0,,
Gene content,41,Xen,Homogroups,1.5,1.00E-02,4,70001,1,1,1,1,1,1,7.62E-05,1,1,0.8534123,0,1,1,1,0,11.15625052,11.15625052,11.15625052,,,0,1,0,,
Gene content,41,Xen,Homogroups,1.5,1.00E-05,1,70001,1,1,1,1,1,1,0.006171311,1,1,0.9672387,0,1,1,1,0,11.15625052,11.15625052,11.15625052,,,0,1,0,,
Gene content,41,Xen,Homogroups,1.5,1.00E-05,2,70001,1,1,1,1,1,1,0,1,1,0.959696,0,1,1,1,0,11.15625052,11.15625052,11.15625052,,,0,1,0,,
Gene content,41,Xen,Homogroups,1.5,1.00E-05,3,70001,1,1,1,1,1,1,0.0002285671,1,1,0.9659816,0,1,1,1,0,11.15625052,11.15625052,11.15625052,,,0,1,0,,
Gene content,41,Xen,Homogroups,1.5,1.00E-05,4,70001,1,1,1,1,1,1,0.007733186,1,1,0.9696768,0,1,1,1,0,11.15625052,11.15625052,11.15625052,,,0,1,0,,
Gene content,41,Xen,Homogroups,1.5,1.00E-09,1,70001,1,1,1,1,1,1,0.9602103,1,1,0.9890097,0,1,1,1,0,11.15625052,11.15625052,11.15625052,,,0,1,0,,
Gene content,41,Xen,Homogroups,1.5,1.00E-09,2,70001,1,1,1,1,1,1,0.9635055,1,1,0.9897335,0,1,1,1,0,11.15625052,11.15625052,11.15625052,,,0,1,0,,
Gene content,41,Xen,Homogroups,1.5,1.00E-09,3,70001,1,1,1,1,1,1,0.9635055,1,1,0.9897335,0,1,1,1,0,11.15625052,11.15625052,11.15625052,,,0,1,0,,
Gene content,41,Xen,Homogroups,1.5,1.00E-09,4,70001,1,1,1,1,1,1,0.9820765,1,1,0.9883431,0,1,1,1,0,11.15625052,11.15625052,11.15625052,,,0,1,0,,
Gene content,41,Xen,Homogroups,1.5,1.00E-12,1,70001,1,1,1,1,1,1,0.005390374,1,1,0.9940763,0,1,1,1,0,11.15625052,11.15625052,11.15625052,,,0,1,0,,
Gene content,41,Xen,Homogroups,1.5,1.00E-12,2,70001,1,1,1,1,1,1,0.01179025,1,1,0.9951049,0,1,1,1,0,11.15625052,11.15625052,11.15625052,,,0,1,0,,
Gene content,41,Xen,Homogroups,1.5,1.00E-12,3,70001,1,1,1,1,1,1,0.01159978,1,1,0.993162,0,1,1,1,0,11.15625052,11.15625052,11.15625052,,,0,1,0,,
Gene content,41,Xen,Homogroups,1.5,1.00E-12,4,70001,1,1,1,1,1,1,0.02649473,1,1,0.9932954,0,1,1,1,0,11.15625052,11.15625052,11.15625052,,,0,1,0,,
Gene content,41,Xen,Homogroups,2.0,1.00E-02,1,70001,1,1,1,1,1,1,0,1,1,0.0001142835,0,1,1,1,0,11.15625052,11.15625052,11.15625052,,,0,1,0,,
Gene content,41,Xen,Homogroups,2.0,1.00E-02,2,70001,1,1,1,1,1,1,3.81E-05,1,1,0.0001714253,0,1,1,1,0,11.15625052,11.15625052,11.15625052,,,0,1,0,,
Gene content,41,Xen,Homogroups,2.0,1.00E-02,3,70001,1,1,1,1,1,1,3.81E-05,1,1,0.0001714253,0,1,1,1,0,11.15625052,11.15625052,11.15625052,,,0,1,0,,
Gene content,41,Xen,Homogroups,2.0,1.00E-02,4,70001,1,1,1,1,1,1,0.01356165,1,1,1.90E-05,0,1,1,1,0,11.15625052,11.15625052,11.15625052,,,0,1,0,,
Gene content,41,Xen,Homogroups,2.0,1.00E-05,1,70001,1,1,1,1,1,1,0,1,1,0.007161768,0,1,1,1,0,11.15625052,11.15625052,11.15625052,,,0,1,0,,
Gene content,41,Xen,Homogroups,2.0,1.00E-05,2,70001,1,1,1,1,1,1,0,1,1,0.007180816,0,1,1,1,0,11.15625052,11.15625052,11.15625052,,,0,1,0,,
Gene content,41,Xen,Homogroups,2.0,1.00E-05,3,70001,1,1,1,1,1,1,0,1,1,0.006780823,0,1,1,1,0,11.15625052,11.15625052,11.15625052,,,0,1,0,,
Gene content,41,Xen,Homogroups,2.0,1.00E-05,4,70001,1,1,1,1,1,1,0,1,1,0.007866517,0,1,1,1,0,11.15625052,11.15625052,11.15625052,,,0,1,0,,
Gene content,41,Xen,Homogroups,2.0,1.00E-09,1,70001,1,1,1,1,1,1,1,1,1,0.2575951,0,1,1,1,0,11.15625052,11.15625052,11.15625052,,,0,1,0,,
Gene content,41,Xen,Homogroups,2.0,1.00E-09,2,70001,1,1,1,1,1,1,0.9953334,1,1,0.2360526,0,1,1,1,0,11.15625052,11.15625052,11.15625052,,,0,1,0,,
Gene content,41,Xen,Homogroups,2.0,1.00E-09,3,70001,1,1,1,1,1,1,0.9953334,1,1,0.2360526,0,1,1,1,0,11.15625052,11.15625052,11.15625052,,,0,1,0,,
Gene content,41,Xen,Homogroups,2.0,1.00E-09,4,70001,1,1,1,1,1,1,0.9999238,1,1,0.2687758,0,1,1,1,0,11.15625052,11.15625052,11.15625052,,,0,1,0,,
Gene content,41,Xen,Homogroups,2.0,1.00E-12,1,70001,1,1,1,1,1,1,1,1,1,0.0007047485,0,1,1,1,0,11.15625052,11.15625052,11.15625052,,,0,1,0,,
Gene content,41,Xen,Homogroups,2.0,1.00E-12,2,70001,1,1,1,1,1,1,1,1,1,0.000895221,0,1,1,1,0,11.15625052,11.15625052,11.15625052,,,0,1,0,,
Gene content,41,Xen,Homogroups,2.0,1.00E-12,3,70001,1,1,1,1,1,1,1,1,1,0.0002095198,0,1,1,1,0,11.15625052,11.15625052,11.15625052,,,0,1,0,,
Gene content,41,Xen,Homogroups,2.0,1.00E-12,4,70001,1,1,1,1,1,1,1,1,1,0.0002666616,0,1,1,1,0,11.15625052,11.15625052,11.15625052,,,0,1,0,,
Gene content,41,Xen,Homogroups,2.5,1.00E-02,1,100001,1,1,1,1,1,1,0,1,1,0.0001199984,0,1,1,1,0,11.51292546,11.51292546,11.51292546,,,0,1,0,,
Gene content,41,Xen,Homogroups,2.5,1.00E-02,2,100001,1,1,1,1,1,1,0,1,1,0.0001199984,0,1,1,1,0,11.51292546,11.51292546,11.51292546,,,0,1,0,,
Gene content,41,Xen,Homogroups,2.5,1.00E-02,3,100001,1,1,1,1,1,1,0.0001333316,1,1,8.00E-05,0,1,1,1,0,11.51292546,11.51292546,11.51292546,,,0,1,0,,
Gene content,41,Xen,Homogroups,2.5,1.00E-02,4,100001,1,1,1,1,1,1,0,1,1,1.33E-05,0,1,1,1,0,11.51292546,11.51292546,11.51292546,,,0,1,0,,
Gene content,41,Xen,Homogroups,2.5,1.00E-05,1,70001,1,1,1,1,1,1,0,1,1,0.001904726,0,1,1,1,0,11.15625052,11.15625052,11.15625052,,,0,1,0,,
Gene content,41,Xen,Homogroups,2.5,1.00E-05,2,70001,1,1,1,1,1,1,0,1,1,0.001276166,0,1,1,1,0,11.15625052,11.15625052,11.15625052,,,0,1,0,,
Gene content,41,Xen,Homogroups,2.5,1.00E-05,3,70001,1,1,1,1,1,1,0,1,1,0.001885678,0,1,1,1,0,11.15625052,11.15625052,11.15625052,,,0,1,0,,
Gene content,41,Xen,Homogroups,2.5,1.00E-05,4,70001,1,1,1,1,1,1,0,1,1,0.00118093,0,1,1,1,0,11.15625052,11.15625052,11.15625052,,,0,1,0,,
Gene content,41,Xen,Homogroups,2.5,1.00E-09,1,70001,1,1,1,1,1,1,0.9909716,1,1,0,0,1,1,1,0,11.15625052,11.15625052,11.15625052,,,0,1,0,,
Gene content,41,Xen,Homogroups,2.5,1.00E-09,2,70001,1,1,1,1,1,1,0.9856193,1,1,1.90E-05,0,1,1,1,0,11.15625052,11.15625052,11.15625052,,,0,1,0,,
Gene content,41,Xen,Homogroups,2.5,1.00E-09,3,70001,1,1,1,1,1,1,0.9856193,1,1,1.90E-05,0,1,1,1,0,11.15625052,11.15625052,11.15625052,,,0,1,0,,
Gene content,41,Xen,Homogroups,2.5,1.00E-09,4,70001,1,1,1,1,1,1,0.9644007,1,1,0,0,1,1,1,0,11.15625052,11.15625052,11.15625052,,,0,1,0,,
Gene content,41,Xen,Homogroups,2.5,1.00E-12,1,70001,1,1,1,1,1,1,0.03516124,1,1,0.0001142835,0,1,1,1,0,11.15625052,11.15625052,11.15625052,,,0,1,0,,
Gene content,41,Xen,Homogroups,2.5,1.00E-12,2,70001,1,1,1,1,1,1,0.04152302,1,1,0.0001142835,0,1,1,1,0,11.15625052,11.15625052,11.15625052,,,0,1,0,,
Gene content,41,Xen,Homogroups,2.5,1.00E-12,3,70001,1,1,1,1,1,1,0.007104627,1,1,1.90E-05,0,1,1,1,0,11.15625052,11.15625052,11.15625052,,,0,1,0,,
Gene content,41,Xen,Homogroups,2.5,1.00E-12,4,70001,1,1,1,1,1,1,0.007104627,1,1,1.90E-05,0,1,1,1,0,11.15625052,11.15625052,11.15625052,,,0,1,0,,
Gene content,41,Xen,Homogroups,4.0,1.00E-02,1,70001,1,1,1,1,1,1,5.71E-05,1,1,0,0,0,1,1,0,0,11.15625052,11.15625052,,,0,1,0,,
Gene content,41,Xen,Homogroups,4.0,1.00E-02,2,70001,1,1,1,1,1,1,0.0009333156,1,1,0,0,0,1,1,0,0,11.15625052,11.15625052,,,0,1,0,,
Gene content,41,Xen,Homogroups,4.0,1.00E-02,3,70001,1,1,1,1,1,1,0.02700901,1,1,0,0,0,1,1,0,0,11.15625052,11.15625052,,,0,1,0,,
Gene content,41,Xen,Homogroups,4.0,1.00E-02,4,70001,1,1,1,1,1,1,0.002171387,1,1,0,0,0,1,1,0,0,11.15625052,11.15625052,,,0,1,0,,
Gene content,41,Xen,Homogroups,4.0,1.00E-05,1,70001,1,1,1,1,1,1,0.1338641,1,1,0,0,0,1,1,0,0,11.15625052,11.15625052,,,0,1,0,,
Gene content,41,Xen,Homogroups,4.0,1.00E-05,2,70001,1,1,1,1,1,1,0.4668863,1,1,0,0,0,1,1,0,0,11.15625052,11.15625052,,,0,1,0,,
Gene content,41,Xen,Homogroups,4.0,1.00E-05,3,70001,1,1,1,1,1,1,0.5266947,1,1,0,0,0,1,1,0,0,11.15625052,11.15625052,,,0,1,0,,
Gene content,41,Xen,Homogroups,4.0,1.00E-05,4,70001,1,1,1,1,1,1,0.4318584,1,1,0,0,0,1,1,0,0,11.15625052,11.15625052,,,0,1,0,,
Gene content,41,Xen,Homogroups,4.0,1.00E-09,1,70001,1,1,1,1,1,1,1,1,1,0,0,0,1,1,0,0,11.15625052,11.15625052,,,0,1,0,,
Gene content,41,Xen,Homogroups,4.0,1.00E-09,2,70001,1,1,1,1,1,1,1,1,1,0,0,0,1,1,0,0,11.15625052,11.15625052,,,0,1,0,,
Gene content,41,Xen,Homogroups,4.0,1.00E-09,3,70001,1,1,1,1,1,1,1,1,1,0,0,0,1,1,0,0,11.15625052,11.15625052,,,0,1,0,,
Gene content,41,Xen,Homogroups,4.0,1.00E-09,4,70001,1,1,1,1,1,1,1,1,1,0,0,0,1,1,0,0,11.15625052,11.15625052,,,0,1,0,,
Gene content,41,Xen,Homogroups,4.0,1.00E-12,1,70001,1,1,1,1,1,1,1,1,1,0,0,0,1,1,0,0,11.15625052,11.15625052,,,0,1,0,,
Gene content,41,Xen,Homogroups,4.0,1.00E-12,2,70001,1,1,1,1,1,1,1,1,1,0,0,1,1,1,0,11.15625052,11.15625052,11.15625052,,,0,1,0,,
Gene content,41,Xen,Homogroups,4.0,1.00E-12,3,70001,1,1,1,1,1,1,1,1,1,0,0,0,1,1,0,0,11.15625052,11.15625052,,,0,1,0,,
Gene content,41,Xen,Homogroups,4.0,1.00E-12,4,70001,1,1,1,1,1,1,1,1,1,0,0,0,1,1,0,0,11.15625052,11.15625052,,,0,1,0,,
Gene content,41,Xen,Homogroups,6.0,1.00E-02,1,70001,1,1,1,1,1,1,0.9923811,1,1,0,0,0,0,0,1,0,-11.15625052,-11.15625052,,,0,0,1,,
Gene content,41,Xen,Homogroups,6.0,1.00E-02,2,70001,1,1,1,1,1,1,1,1,1,0,0,0,0,0,1,0,-11.15625052,-11.15625052,,,0,0,1,,
Gene content,41,Xen,Homogroups,6.0,1.00E-02,3,70001,1,1,1,1,1,1,1,1,1,0,0,0,0,0,1,0,-11.15625052,-11.15625052,,,0,0,1,,
Gene content,41,Xen,Homogroups,6.0,1.00E-02,4,70001,1,1,1,1,1,1,1,1,1,0,0,0,0,0,1,0,-11.15625052,-11.15625052,,,0,0,1,,
Gene content,41,Xen,Homogroups,6.0,1.00E-05,1,70001,1,1,1,1,1,1,1,1,1,0,0,0,0,0,1,0,-11.15625052,-11.15625052,,,0,0,1,,
Gene content,41,Xen,Homogroups,6.0,1.00E-05,2,70001,1,1,1,1,1,1,1,1,1,0,0,0,0,0,1,0,-11.15625052,-11.15625052,,,0,0,1,,
Gene content,41,Xen,Homogroups,6.0,1.00E-05,3,70001,1,1,1,1,1,1,1,1,1,0,0,0,0,0,1,0,-11.15625052,-11.15625052,,,0,0,1,,
Gene content,41,Xen,Homogroups,6.0,1.00E-05,4,70001,1,1,1,1,1,1,1,1,1,0,0,0,0,0,1,0,-11.15625052,-11.15625052,,,0,0,1,,
Gene content,41,Xen,Homogroups,6.0,1.00E-09,1,100001,1,1,1,1,1,1,1,1,1,0,0,0,5.33E-05,0.0001066652,0.9998933,0,-9.145708895,-9.838909072,,,0,0,1,,
Gene content,41,Xen,Homogroups,6.0,1.00E-09,2,100001,1,1,1,1,1,1,1,1,1,0,0,0,0.0003333289,0.0003599952,0.99964,0,-7.929059795,-8.006047483,,,0,0,1,,
Gene content,41,Xen,Homogroups,6.0,1.00E-09,3,100001,1,1,1,1,1,1,1,1,1,0,0,0,1.33E-05,5.33E-05,0.9999467,0,-9.838909105,-11.22524306,,,0,0,1,,
Gene content,41,Xen,Homogroups,6.0,1.00E-09,4,100001,1,1,1,1,1,1,1,1,1,0,0,0,1.33E-05,5.33E-05,0.9999467,0,-9.838909105,-11.22524306,,,0,0,1,,
Gene content,41,Xen,Homogroups,6.0,1.00E-12,1,70001,1,1,1,1,1,1,1,1,1,0,0,0,0,0,1,0,-11.15625052,-11.15625052,,,0,0,1,,
Gene content,41,Xen,Homogroups,6.0,1.00E-12,2,70001,1,1,1,1,1,1,1,1,1,0,0,0,0,0,1,0,-11.15625052,-11.15625052,,,0,0,1,,
Gene content,41,Xen,Homogroups,6.0,1.00E-12,3,70001,1,1,1,1,1,1,1,1,1,0,0,0,0,0,1,0,-11.15625052,-11.15625052,,,0,0,1,,
Gene content,41,Xen,Homogroups,6.0,1.00E-12,4,70001,1,1,1,1,1,1,1,1,1,0,0,0,0,0,1,0,-11.15625052,-11.15625052,,,0,0,1,,

Gene content,41,Xen,Orthogroups,1.5,1.00E-02,1,100001,1,1,1,1,1,1,0.1161718,1,1,0.99928,0,1,1,1,0,11.51292546,11.51292546,11.51292546,1,0,1,0,1,0,
Gene content,41,Xen,Orthogroups,1.5,1.00E-02,2,100001,1,1,1,1,1,1,0.07779896,1,1,0.9988667,0,1,1,1,0,11.51292546,11.51292546,11.51292546,1,0,1,0,1,0,
Gene content,41,Xen,Orthogroups,1.5,1.00E-02,3,100001,1,1,1,1,1,1,0.07779896,1,1,0.9988667,0,1,1,1,0,11.51292546,11.51292546,11.51292546,1,0,1,0,1,0,
Gene content,41,Xen,Orthogroups,1.5,1.00E-02,4,100001,1,1,1,1,1,1,0.07779896,1,1,0.9988667,0,1,1,1,0,11.51292546,11.51292546,11.51292546,1,0,1,0,1,0,
Gene content,41,Xen,Orthogroups,1.5,1.00E-05,1,100001,1,1,1,1,1,1,0,1,1,0.9994,0,1,1,1,0,11.51292546,11.51292546,11.51292546,1,0,1,0,1,0,
Gene content,41,Xen,Orthogroups,1.5,1.00E-05,2,100001,1,1,1,1,1,1,0,1,1,0.9993733,0,1,1,1,0,11.51292546,11.51292546,11.51292546,1,0,1,0,1,0,
Gene content,41,Xen,Orthogroups,1.5,1.00E-05,3,100001,1,1,1,1,1,1,0,1,1,0.9995733,0,1,1,1,0,11.51292546,11.51292546,11.51292546,1,0,1,0,1,0,
Gene content,41,Xen,Orthogroups,1.5,1.00E-05,4,100001,1,1,1,1,1,1,0.005826589,1,1,0.9994,0,1,1,1,0,11.51292546,11.51292546,11.51292546,1,0,1,0,1,0,
Gene content,41,Xen,Orthogroups,1.5,1.00E-09,1,92605,1,1,1,1,1,1,0.9947159,1,1,0.9997696,0,1,1,1,0,11.43608762,11.43608762,11.43608762,1,0,1,0,1,0,
Gene content,41,Xen,Orthogroups,1.5,1.00E-09,2,83266,1,1,1,1,1,1,0.9980304,1,1,0.9996477,0,1,1,1,0,11.32978357,11.32978357,11.32978357,1,0,1,0,1,0,
Gene content,41,Xen,Orthogroups,1.5,1.00E-09,3,80151,1,1,1,1,1,1,0.9997837,1,1,0.9999002,0,1,1,1,0,11.29165516,11.29165516,11.29165516,1,0,1,0,1,0,
Gene content,41,Xen,Orthogroups,1.5,1.00E-09,4,71939,1,1,1,1,1,1,0.9877861,1,1,0.9994254,0,1,1,1,0,11.18355992,11.18355992,11.18355992,1,0,1,0,1,0,
Gene content,41,Xen,Orthogroups,1.5,1.00E-12,1,100001,1,1,1,1,1,1,0.8437754,1,1,1,0,1,1,1,0,11.51292546,11.51292546,11.51292546,1,0,1,0,1,0,
Gene content,41,Xen,Orthogroups,1.5,1.00E-12,2,100001,1,1,1,1,1,1,0.9072412,1,1,1,0,1,1,1,0,11.51292546,11.51292546,11.51292546,1,0,1,0,1,0,
Gene content,41,Xen,Orthogroups,1.5,1.00E-12,3,100001,1,1,1,1,1,1,0.9072412,1,1,1,0,1,1,1,0,11.51292546,11.51292546,11.51292546,1,0,1,0,1,0,
Gene content,41,Xen,Orthogroups,1.5,1.00E-12,4,100001,1,1,1,1,1,1,0.8986814,1,1,1,0,1,1,1,0,11.51292546,11.51292546,11.51292546,1,0,1,0,1,0,
Gene content,41,Xen,Orthogroups,2.0,1.00E-02,1,70001,1,1,1,1,1,1,0,1,1,0.4827718,0,1,1,1,0,11.15625052,11.15625052,11.15625052,1,0,1,0,1,0,
Gene content,41,Xen,Orthogroups,2.0,1.00E-02,2,70001,1,1,1,1,1,1,0,1,1,0.4864288,0,1,1,1,0,11.15625052,11.15625052,11.15625052,1,0,1,0,1,0,
Gene content,41,Xen,Orthogroups,2.0,1.00E-02,3,70001,1,1,1,1,1,1,0,1,1,0.4875336,0,1,1,1,0,11.15625052,11.15625052,11.15625052,1,0,1,0,1,0,
Gene content,41,Xen,Orthogroups,2.0,1.00E-02,4,70001,1,1,1,1,1,1,0,1,1,0.4886764,0,1,1,1,0,11.15625052,11.15625052,11.15625052,1,0,1,0,1,0,
Gene content,41,Xen,Orthogroups,2.0,1.00E-05,1,100001,1,1,1,1,1,1,0,1,1,0.9925334,0,1,1,1,0,11.51292546,11.51292546,11.51292546,1,0,1,0,1,0,
Gene content,41,Xen,Orthogroups,2.0,1.00E-05,2,100001,1,1,1,1,1,1,0,1,1,0.9940401,0,1,1,1,0,11.51292546,11.51292546,11.51292546,1,0,1,0,1,0,
Gene content,41,Xen,Orthogroups,2.0,1.00E-05,3,100001,1,1,1,1,1,1,0,1,1,0.9942534,0,1,1,1,0,11.51292546,11.51292546,11.51292546,1,0,1,0,1,0,
Gene content,41,Xen,Orthogroups,2.0,1.00E-05,4,100001,1,1,1,1,1,1,0,1,1,0.9947201,0,1,1,1,0,11.51292546,11.51292546,11.51292546,1,0,1,0,1,0,
Gene content,41,Xen,Orthogroups,2.0,1.00E-09,1,64263,1,1,1,1,1,1,1,1,1,1,0,1,1,1,0,11.07072376,11.07072376,11.07072376,1,0,1,0,1,0,
Gene content,41,Xen,Orthogroups,2.0,1.00E-09,2,58474,1,1,1,1,1,1,1,1,1,1,0,1,1,1,0,10.97632039,10.97632039,10.97632039,1,0,1,0,1,0,
Gene content,41,Xen,Orthogroups,2.0,1.00E-09,3,74887,1,1,1,1,1,1,1,1,1,1,0,1,1,1,0,11.22372224,11.22372224,11.22372224,1,0,1,0,1,0,
Gene content,41,Xen,Orthogroups,2.0,1.00E-09,4,64452,1,1,1,1,1,1,1,1,1,1,0,1,1,1,0,11.07366052,11.07366052,11.07366052,1,0,1,0,1,0,
Gene content,41,Xen,Orthogroups,2.0,1.00E-12,1,70001,1,1,1,1,1,1,1,1,1,1,0,1,1,1,0,11.15625052,11.15625052,11.15625052,1,0,1,0,1,0,
Gene content,41,Xen,Orthogroups,2.0,1.00E-12,2,70001,1,1,1,1,1,1,1,1,1,1,0,1,1,1,0,11.15625052,11.15625052,11.15625052,1,0,1,0,1,0,
Gene content,41,Xen,Orthogroups,2.0,1.00E-12,3,70001,1,1,1,1,1,1,1,1,1,1,0,1,1,1,0,11.15625052,11.15625052,11.15625052,1,0,1,0,1,0,
Gene content,41,Xen,Orthogroups,2.0,1.00E-12,4,70001,1,1,1,1,1,1,1,1,1,1,0,1,1,1,0,11.15625052,11.15625052,11.15625052,1,0,1,0,1,0,
Gene content,41,Xen,Orthogroups,2.5,1.00E-02,1,70001,1,1,1,1,1,1,0.3178797,1,1,0.5909221,0,1,1,1,0,11.15625052,11.15625052,11.15625052,1,0,1,0,1,0,
Gene content,41,Xen,Orthogroups,2.5,1.00E-02,2,70001,1,1,1,1,1,1,0.2943563,1,1,0.579189,0,1,1,1,0,11.15625052,11.15625052,11.15625052,1,0,1,0,1,0,
Gene content,41,Xen,Orthogroups,2.5,1.00E-02,3,70001,1,1,1,1,1,1,0.3442411,1,1,0.5755509,0,1,1,1,0,11.15625052,11.15625052,11.15625052,1,0,1,0,1,0,
Gene content,41,Xen,Orthogroups,2.5,1.00E-02,4,70001,1,1,1,1,1,1,0.1758443,1,1,0.5842365,0,1,1,1,0,11.15625052,11.15625052,11.15625052,1,0,1,0,1,0,
Gene content,41,Xen,Orthogroups,2.5,1.00E-05,1,100001,1,1,1,1,1,1,0.5718457,1,1,0.99808,0,1,1,1,0,11.51292546,11.51292546,11.51292546,1,0,1,0,1,0,
Gene content,41,Xen,Orthogroups,2.5,1.00E-05,2,100001,1,1,1,1,1,1,0.5427794,1,1,0.9969734,0,1,1,1,0,11.51292546,11.51292546,11.51292546,1,0,1,0,1,0,
Gene content,41,Xen,Orthogroups,2.5,1.00E-05,3,100001,1,1,1,1,1,1,0.4949667,1,1,0.9972134,0,1,1,1,0,11.51292546,11.51292546,11.51292546,1,0,1,0,1,0,
Gene content,41,Xen,Orthogroups,2.5,1.00E-05,4,100001,1,1,1,1,1,1,0.3116758,1,1,0.9964667,0,1,1,1,0,11.51292546,11.51292546,11.51292546,1,0,1,0,1,0,
Gene content,41,Xen,Orthogroups,2.5,1.00E-09,1,78500,1,1,1,1,1,1,1,1,1,1,0,1,1,1,0,11.27084116,11.27084116,11.27084116,1,0,1,0,1,0,
Gene content,41,Xen,Orthogroups,2.5,1.00E-09,2,62067,1,1,1,1,1,1,1,1,1,1,0,1,1,1,0,11.03595361,11.03595361,11.03595361,1,0,1,0,1,0,
Gene content,41,Xen,Orthogroups,2.5,1.00E-09,3,61395,1,1,1,1,1,1,1,1,1,0.9999783,0,1,1,1,0,11.02506739,11.02506739,11.02506739,1,0,1,0,1,0,
Gene content,41,Xen,Orthogroups,2.5,1.00E-09,4,69600,1,1,1,1,1,1,1,1,1,0.9998659,0,1,1,1,0,11.15050548,11.15050548,11.15050548,1,0,1,0,1,0,
Gene content,41,Xen,Orthogroups,2.5,1.00E-12,1,70001,1,1,1,1,1,1,1,1,1,1,0,1,1,1,0,11.15625052,11.15625052,11.15625052,1,0,1,0,1,0,
Gene content,41,Xen,Orthogroups,2.5,1.00E-12,2,70001,1,1,1,1,1,1,1,1,1,1,0,1,1,1,0,11.15625052,11.15625052,11.15625052,1,0,1,0,1,0,
Gene content,41,Xen,Orthogroups,2.5,1.00E-12,3,70001,1,1,1,1,1,1,1,1,1,1,0,1,1,1,0,11.15625052,11.15625052,11.15625052,1,0,1,0,1,0,
Gene content,41,Xen,Orthogroups,2.5,1.00E-12,4,70001,1,1,1,1,1,1,1,1,1,1,0,1,1,1,0,11.15625052,11.15625052,11.15625052,1,0,1,0,1,0,
Gene content,41,Xen,Orthogroups,4.0,1.00E-02,1,70001,1,1,1,1,1,1,1,1,1,0.05057047,0,1,1,1,0,11.15625052,11.15625052,11.15625052,1,0,1,0,1,0,
Gene content,41,Xen,Orthogroups,4.0,1.00E-02,2,70001,1,1,1,1,1,1,1,1,1,0.05005619,0,1,1,1,0,11.15625052,11.15625052,11.15625052,1,0,1,0,1,0,
Gene content,41,Xen,Orthogroups,4.0,1.00E-02,3,70001,1,1,1,1,1,1,1,1,1,0.04712291,0,1,1,1,0,11.15625052,11.15625052,11.15625052,1,0,1,0,1,0,
Gene content,41,Xen,Orthogroups,4.0,1.00E-02,4,70001,1,1,1,1,1,1,1,1,1,0.04958001,0,1,1,1,0,11.15625052,11.15625052,11.15625052,1,0,1,0,1,0,
Gene content,41,Xen,Orthogroups,4.0,1.00E-05,1,100001,1,1,1,1,1,1,0.9991733,1,1,0.6958307,0,1,1,1,0,11.51292546,11.51292546,11.51292546,1,0,1,0,1,0,
Gene content,41,Xen,Orthogroups,4.0,1.00E-05,2,100001,1,1,1,1,1,1,1,1,1,0.6909375,0,1,1,1,0,11.51292546,11.51292546,11.51292546,1,0,1,0,1,0,
Gene content,41,Xen,Orthogroups,4.0,1.00E-05,3,100001,1,1,1,1,1,1,0.9999733,1,1,0.6742043,0,1,1,1,0,11.51292546,11.51292546,11.51292546,1,0,1,0,1,0,
Gene content,41,Xen,Orthogroups,4.0,1.00E-05,4,100001,1,1,1,1,1,1,1,1,1,0.6819376,0,1,1,1,0,11.51292546,11.51292546,11.51292546,1,0,1,0,1,0,
Gene content,41,Xen,Orthogroups,4.0,1.00E-09,1,74316,1,1,1,1,1,1,1,1,1,0.9547159,0,1,1,1,0,11.21606809,11.21606809,11.21606809,1,0,1,0,1,0,
Gene content,41,Xen,Orthogroups,4.0,1.00E-09,2,57603,1,1,1,1,1,1,1,1,1,0.9364396,0,1,1,1,0,10.96131257,10.96131257,10.96131257,1,0,1,0,1,0,
Gene content,41,Xen,Orthogroups,4.0,1.00E-09,3,71674,1,1,1,1,1,1,1,1,1,0.9523216,0,1,1,1,0,11.17986939,11.17986939,11.17986939,1,0,1,0,1,0,
Gene content,41,Xen,Orthogroups,4.0,1.00E-09,4,70565,1,1,1,1,1,1,1,1,1,0.952838,0,1,1,1,0,11.16427538,11.16427538,11.16427538,1,0,1,0,1,0,
Gene content,41,Xen,Orthogroups,4.0,1.00E-12,1,70001,1,1,1,1,1,1,1,1,1,0.9976572,0,1,1,1,0,11.15625052,11.15625052,11.15625052,1,0,1,0,1,0,
Gene content,41,Xen,Orthogroups,4.0,1.00E-12,2,70001,1,1,1,1,1,1,1,1,1,0.9970286,0,1,1,1,0,11.15625052,11.15625052,11.15625052,1,0,1,0,1,0,
Gene content,41,Xen,Orthogroups,4.0,1.00E-12,3,70001,1,1,1,1,1,1,1,1,1,0.9969905,0,1,1,1,0,11.15625052,11.15625052,11.15625052,1,0,1,0,1,0,
Gene content,41,Xen,Orthogroups,4.0,1.00E-12,4,70001,1,1,1,1,1,1,1,1,1,0.9975429,0,1,1,1,0,11.15625052,11.15625052,11.15625052,1,0,1,0,1,0,
Gene content,41,Xen,Orthogroups,6.0,1.00E-02,1,70001,1,1,1,1,1,1,1,1,1,0.2187958,0,1,1,1,0,11.15625052,11.15625052,11.15625052,1,0,1,0,1,0,
Gene content,41,Xen,Orthogroups,6.0,1.00E-02,2,70001,1,1,1,1,1,1,1,1,1,0.2128531,0,1,1,1,0,11.15625052,11.15625052,11.15625052,1,0,1,0,1,0,
Gene content,41,Xen,Orthogroups,6.0,1.00E-02,3,70001,1,1,1,1,1,1,1,1,1,0.2128531,0,1,1,1,0,11.15625052,11.15625052,11.15625052,1,0,1,0,1,0,
Gene content,41,Xen,Orthogroups,6.0,1.00E-02,4,70001,1,1,1,1,1,1,1,1,1,0.1999581,0,1,1,1,0,11.15625052,11.15625052,11.15625052,1,0,1,0,1,0,
Gene content,41,Xen,Orthogroups,6.0,1.00E-05,1,100001,1,1,1,1,1,1,1,1,1,0.124025,0,1,1,1,0,11.51292546,11.51292546,11.51292546,1,0,1,0,1,0,
Gene content,41,Xen,Orthogroups,6.0,1.00E-05,2,100001,1,1,1,1,1,1,1,1,1,0.1188651,0,1,1,1,0,11.51292546,11.51292546,11.51292546,1,0,1,0,1,0,
Gene content,41,Xen,Orthogroups,6.0,1.00E-05,3,100001,1,1,1,1,1,1,1,1,1,0.1188651,0,1,1,1,0,11.51292546,11.51292546,11.51292546,1,0,1,0,1,0,
Gene content,41,Xen,Orthogroups,6.0,1.00E-05,4,100001,1,1,1,1,1,1,1,1,1,0.1182118,0,1,1,1,0,11.51292546,11.51292546,11.51292546,1,0,1,0,1,0,
Gene content,41,Xen,Orthogroups,6.0,1.00E-09,1,58435,1,1,1,1,1,1,1,1,1,0.9866977,0,1,1,1,0,10.97565319,10.97565319,10.97565319,1,0,1,0,1,0,
Gene content,41,Xen,Orthogroups,6.0,1.00E-09,2,67914,1,1,1,1,1,1,1,1,1,0.9833909,0,1,1,1,0,11.12598275,11.12598275,11.12598275,1,0,1,0,1,0,
Gene content,41,Xen,Orthogroups,6.0,1.00E-09,3,67032,1,1,1,1,1,1,1,1,1,0.9829932,0,1,1,1,0,11.11291048,11.11291048,11.11291048,1,0,1,0,1,0,
Gene content,41,Xen,Orthogroups,6.0,1.00E-09,4,62774,1,1,1,1,1,1,1,1,1,0.9812876,0,1,1,1,0,11.04728032,11.04728032,11.04728032,1,0,1,0,1,0,
Gene content,41,Xen,Orthogroups,6.0,1.00E-12,1,70001,1,1,1,1,1,1,1,1,1,0.9828194,0,1,1,1,0,11.15625052,11.15625052,11.15625052,1,0,1,0,1,0,
Gene content,41,Xen,Orthogroups,6.0,1.00E-12,2,70001,1,1,1,1,1,1,1,1,1,0.9844384,0,1,1,1,0,11.15625052,11.15625052,11.15625052,1,0,1,0,1,0,
Gene content,41,Xen,Orthogroups,6.0,1.00E-12,3,70001,1,1,1,1,1,1,1,1,1,0.9825337,0,1,1,1,0,11.15625052,11.15625052,11.15625052,1,0,1,0,1,0,
Gene content,41,Xen,Orthogroups,6.0,1.00E-12,4,70001,1,1,1,1,1,1,1,1,1,0.983067,0,1,1,1,0,11.15625052,11.15625052,11.15625052,1,0,1,0,1,0,
Gene content,44,Aco,Homogroups,1.5,1.00E-02,1,70001,1,1,1,1,1,1,3.81E-05,1,1,0.3663359,0,1,1,1,0,11.15625052,11.15625052,11.15625052,1,0,1,0,1,0,
Gene content,44,Aco,Homogroups,1.5,1.00E-02,2,70001,1,1,1,1,1,1,0,1,1,0.3580694,0,1,1,1,0,11.15625052,11.15625052,11.15625052,1,0,1,0,1,0,

Gene content,44,Aco,Homogroups,1.5,1.00E-02,3,70001,1,1,1,1,1,1,1.90E-05,1,1,0.3738024,0,1,1,1,0,11.15625052,11.15625052,11.15625052,1,0,1,0,1,0,
Gene content,44,Aco,Homogroups,1.5,1.00E-02,4,70001,1,1,1,1,1,1,3.81E-05,1,1,0.3617074,0,1,1,1,0,11.15625052,11.15625052,11.15625052,1,0,1,0,1,0,
Gene content,44,Aco,Homogroups,1.5,1.00E-05,1,70001,1,1,1,1,1,1,0.002742805,1,1,0.3577265,0,1,1,1,0,11.15625052,11.15625052,11.15625052,1,0,1,0,1,0,
Gene content,44,Aco,Homogroups,1.5,1.00E-05,2,70001,1,1,1,1,1,1,0,1,1,0.3670406,0,1,1,1,0,11.15625052,11.15625052,11.15625052,1,0,1,0,1,0,
Gene content,44,Aco,Homogroups,1.5,1.00E-05,3,70001,1,1,1,1,1,1,0,1,1,0.3685454,0,1,1,1,0,11.15625052,11.15625052,11.15625052,1,0,1,0,1,0,
Gene content,44,Aco,Homogroups,1.5,1.00E-05,4,70001,1,1,1,1,1,1,0,1,1,0.3667549,0,1,1,1,0,11.15625052,11.15625052,11.15625052,1,0,1,0,1,0,
Gene content,44,Aco,Homogroups,1.5,1.00E-09,1,70001,1,1,1,1,1,1,0.6567875,1,1,0.826689,0,1,1,1,0,11.15625052,11.15625052,11.15625052,1,0,1,0,1,0,
Gene content,44,Aco,Homogroups,1.5,1.00E-09,2,70001,1,1,1,1,1,1,0.6201596,1,1,0.8255462,0,1,1,1,0,11.15625052,11.15625052,11.15625052,1,0,1,0,1,0,
Gene content,44,Aco,Homogroups,1.5,1.00E-09,3,70001,1,1,1,1,1,1,0.7086341,1,1,0.8307461,0,1,1,1,0,11.15625052,11.15625052,11.15625052,1,0,1,0,1,0,
Gene content,44,Aco,Homogroups,1.5,1.00E-09,4,70001,1,1,1,1,1,1,0.594941,1,1,0.8169368,0,1,1,1,0,11.15625052,11.15625052,11.15625052,1,0,1,0,1,0,
Gene content,44,Aco,Homogroups,1.5,1.00E-12,1,70001,1,1,1,1,1,1,0.00139045,1,1,0.8844212,0,1,1,1,0,11.15625052,11.15625052,11.15625052,1,0,1,0,1,0,
Gene content,44,Aco,Homogroups,1.5,1.00E-12,2,70001,1,1,1,1,1,1,5.71E-05,1,1,0.879488,0,1,1,1,0,11.15625052,11.15625052,11.15625052,1,0,1,0,1,0,
Gene content,44,Aco,Homogroups,1.5,1.00E-12,3,70001,1,1,1,1,1,1,5.71E-05,1,1,0.879488,0,1,1,1,0,11.15625052,11.15625052,11.15625052,1,0,1,0,1,0,
Gene content,44,Aco,Homogroups,1.5,1.00E-12,4,70001,1,1,1,1,1,1,0.001085694,1,1,0.8900402,0,1,1,1,0,11.15625052,11.15625052,11.15625052,1,0,1,0,1,0,
Gene content,44,Aco,Homogroups,2.0,1.00E-02,1,70001,1,1,1,1,1,1,0,1,1,0.000819032,0,1,1,1,0,11.15625052,11.15625052,11.15625052,1,0,1,0,1,0,
Gene content,44,Aco,Homogroups,2.0,1.00E-02,2,70001,1,1,1,1,1,1,0,1,1,0.0006285595,0,1,1,1,0,11.15625052,11.15625052,11.15625052,1,0,1,0,1,0,
Gene content,44,Aco,Homogroups,2.0,1.00E-02,3,70001,1,1,1,1,1,1,0,1,1,0.0006285595,0,1,1,1,0,11.15625052,11.15625052,11.15625052,1,0,1,0,1,0,
Gene content,44,Aco,Homogroups,2.0,1.00E-02,4,70001,1,1,1,1,1,1,0,1,1,0.0009714101,0,1,1,1,0,11.15625052,11.15625052,11.15625052,1,0,1,0,1,0,
Gene content,44,Aco,Homogroups,2.0,1.00E-05,1,70001,1,1,1,1,1,1,0,1,1,0.01009505,0,1,1,1,0,11.15625052,11.15625052,11.15625052,1,0,1,0,1,0,
Gene content,44,Aco,Homogroups,2.0,1.00E-05,2,70001,1,1,1,1,1,1,0,1,1,0.01062837,0,1,1,1,0,11.15625052,11.15625052,11.15625052,1,0,1,0,1,0,
Gene content,44,Aco,Homogroups,2.0,1.00E-05,3,70001,1,1,1,1,1,1,0,1,1,0.008914116,0,1,1,1,0,11.15625052,11.15625052,11.15625052,1,0,1,0,1,0,
Gene content,44,Aco,Homogroups,2.0,1.00E-05,4,70001,1,1,1,1,1,1,0,1,1,0.007599855,0,1,1,1,0,11.15625052,11.15625052,11.15625052,1,0,1,0,1,0,
Gene content,44,Aco,Homogroups,2.0,1.00E-09,1,70001,1,1,1,1,1,1,1,1,1,0.132188,0,1,1,1,0,11.15625052,11.15625052,11.15625052,1,0,1,0,1,0,
Gene content,44,Aco,Homogroups,2.0,1.00E-09,2,70001,1,1,1,1,1,1,0.9973143,1,1,0.1102265,0,1,1,1,0,11.15625052,11.15625052,11.15625052,1,0,1,0,1,0,
Gene content,44,Aco,Homogroups,2.0,1.00E-09,3,70001,1,1,1,1,1,1,0.999981,1,1,0.1219977,0,1,1,1,0,11.15625052,11.15625052,11.15625052,1,0,1,0,1,0,
Gene content,44,Aco,Homogroups,2.0,1.00E-09,4,70001,1,1,1,1,1,1,0.999981,1,1,0.1219977,0,1,1,1,0,11.15625052,11.15625052,11.15625052,1,0,1,0,1,0,
Gene content,44,Aco,Homogroups,2.0,1.00E-12,1,70001,1,1,1,1,1,1,0.9969143,1,1,0,0,1,1,1,0,11.15625052,11.15625052,11.15625052,1,0,1,0,1,0,
Gene content,44,Aco,Homogroups,2.0,1.00E-12,2,70001,1,1,1,1,1,1,0.9991048,1,1,0.0001142835,0,1,1,1,0,11.15625052,11.15625052,11.15625052,1,0,1,0,1,0,
Gene content,44,Aco,Homogroups,2.0,1.00E-12,3,70001,1,1,1,1,1,1,0.9865717,1,1,1.90E-05,0,1,1,1,0,11.15625052,11.15625052,11.15625052,1,0,1,0,1,0,
Gene content,44,Aco,Homogroups,2.0,1.00E-12,4,70001,1,1,1,1,1,1,1,1,1,5.71E-05,0,1,1,1,0,11.15625052,11.15625052,11.15625052,1,0,1,0,1,0,
Gene content,44,Aco,Homogroups,2.5,1.00E-02,1,70001,1,1,1,1,1,1,0,1,1,0,0,0.2006819,1,1,0,9.550230595,11.15625052,11.15625052,1,0,1,0,1,0,
Gene content,44,Aco,Homogroups,2.5,1.00E-02,2,70001,1,1,1,1,1,1,0,1,1,1.90E-05,0,0.7969562,1,1,0,10.92930925,11.15625052,11.15625052,1,0,1,0,1,0,
Gene content,44,Aco,Homogroups,2.5,1.00E-02,3,70001,1,1,1,1,1,1,0,1,1,0,0,0.2384145,1,1,0,9.722520283,11.15625052,11.15625052,1,0,1,0,1,0,
Gene content,44,Aco,Homogroups,2.5,1.00E-02,4,70001,1,1,1,1,1,1,0,1,1,0.0001142835,0,0.5713986,1,1,0,10.59659657,11.15625052,11.15625052,1,0,1,0,1,0,
Gene content,44,Aco,Homogroups,2.5,1.00E-05,1,70001,1,1,1,1,1,1,0,1,1,0.001028552,0,1,1,1,0,11.15625052,11.15625052,11.15625052,1,0,1,0,1,0,
Gene content,44,Aco,Homogroups,2.5,1.00E-05,2,70001,1,1,1,1,1,1,0,1,1,0.0005142759,0,1,1,1,0,11.15625052,11.15625052,11.15625052,1,0,1,0,1,0,
Gene content,44,Aco,Homogroups,2.5,1.00E-05,3,70001,1,1,1,1,1,1,0,1,1,0.0001904726,0,1,1,1,0,11.15625052,11.15625052,11.15625052,1,0,1,0,1,0,
Gene content,44,Aco,Homogroups,2.5,1.00E-05,4,70001,1,1,1,1,1,1,0,1,1,0.0008380793,0,1,1,1,0,11.15625052,11.15625052,11.15625052,1,0,1,0,1,0,
Gene content,44,Aco,Homogroups,2.5,1.00E-09,1,70001,1,1,1,1,1,1,0.9997905,1,1,3.81E-05,0,1,1,1,0,11.15625052,11.15625052,11.15625052,1,0,1,0,1,0,
Gene content,44,Aco,Homogroups,2.5,1.00E-09,2,70001,1,1,1,1,1,1,0.9894859,1,1,0.0001333308,0,1,1,1,0,11.15625052,11.15625052,11.15625052,1,0,1,0,1,0,
Gene content,44,Aco,Homogroups,2.5,1.00E-09,3,70001,1,1,1,1,1,1,0.9942096,1,1,0,0,1,1,1,0,11.15625052,11.15625052,11.15625052,1,0,1,0,1,0,
Gene content,44,Aco,Homogroups,2.5,1.00E-09,4,70001,1,1,1,1,1,1,0.9942096,1,1,0,0,1,1,1,0,11.15625052,11.15625052,11.15625052,1,0,1,0,1,0,
Gene content,44,Aco,Homogroups,2.5,1.00E-12,1,70001,1,1,1,1,1,1,0,1,1,3.81E-05,0,1,1,1,0,11.15625052,11.15625052,11.15625052,1,0,1,0,1,0,
Gene content,44,Aco,Homogroups,2.5,1.00E-12,2,70001,1,1,1,1,1,1,0.00506657,1,1,3.81E-05,0,1,1,1,0,11.15625052,11.15625052,11.15625052,1,0,1,0,1,0,
Gene content,44,Aco,Homogroups,2.5,1.00E-12,3,70001,1,1,1,1,1,1,0.001485686,1,1,7.62E-05,0,1,1,1,0,11.15625052,11.15625052,11.15625052,1,0,1,0,1,0,
Gene content,44,Aco,Homogroups,2.5,1.00E-12,4,70001,1,1,1,1,1,1,0,1,1,7.62E-05,0,1,1,1,0,11.15625052,11.15625052,11.15625052,1,0,1,0,1,0,
Gene content,44,Aco,Homogroups,4.0,1.00E-02,1,70001,1,1,1,1,1,1,0.9273919,1,1,0,0,0,1,1,0,0,11.15625052,11.15625052,0,0,1,0,1,0,
Gene content,44,Aco,Homogroups,4.0,1.00E-02,2,70001,1,1,1,1,1,1,0.8299651,1,1,0,0,0,1,1,0,0,11.15625052,11.15625052,0,0,1,0,1,0,
Gene content,44,Aco,Homogroups,4.0,1.00E-02,3,70001,1,1,1,1,1,1,0.8158511,1,1,0,0,0,1,1,0,0,11.15625052,11.15625052,0,0,1,0,1,0,
Gene content,44,Aco,Homogroups,4.0,1.00E-02,4,70001,1,1,1,1,1,1,0.8158511,1,1,0,0,0,1,1,0,0,11.15625052,11.15625052,0,0,1,0,1,0,
Gene content,44,Aco,Homogroups,4.0,1.00E-05,1,70001,1,1,1,1,1,1,0.9997333,1,1,0,0,0,1,1,0,0,11.15625052,11.15625052,0,0,1,0,1,0,
Gene content,44,Aco,Homogroups,4.0,1.00E-05,2,70001,1,1,1,1,1,1,0.9986286,1,1,0,0,0,1,1,0,0,11.15625052,11.15625052,0,0,1,0,1,0,
Gene content,44,Aco,Homogroups,4.0,1.00E-05,3,70001,1,1,1,1,1,1,0.9935049,1,1,0,0,0,1,1,0,0,11.15625052,11.15625052,0,0,1,0,1,0,
Gene content,44,Aco,Homogroups,4.0,1.00E-05,4,70001,1,1,1,1,1,1,0.995581,1,1,0,0,0,1,1,0,0,11.15625052,11.15625052,0,0,1,0,1,0,
Gene content,44,Aco,Homogroups,4.0,1.00E-09,1,70001,1,1,1,1,1,1,1,1,1,0,0,1,1,1,0,11.15625052,11.15625052,11.15625052,1,0,1,0,1,0,
Gene content,44,Aco,Homogroups,4.0,1.00E-09,2,70001,1,1,1,1,1,1,1,1,1,0,0,0.970972,1,1,0,11.12680716,11.15625052,11.15625052,1,0,1,0,1,0,
Gene content,44,Aco,Homogroups,4.0,1.00E-09,3,70001,1,1,1,1,1,1,1,1,1,0,0,1,1,1,0,11.15625052,11.15625052,11.15625052,1,0,1,0,1,0,
Gene content,44,Aco,Homogroups,4.0,1.00E-09,4,70001,1,1,1,1,1,1,1,1,1,0,0,1,1,1,0,11.15625052,11.15625052,11.15625052,1,0,1,0,1,0,
Gene content,44,Aco,Homogroups,4.0,1.00E-12,1,70001,1,1,1,1,1,1,0.999981,1,1,0,0,0,1,1,0,0,11.15625052,11.15625052,0,0,1,0,1,0,
Gene content,44,Aco,Homogroups,4.0,1.00E-12,2,70001,1,1,1,1,1,1,0.9803242,1,1,0,0,1,1,1,0,11.15625052,11.15625052,11.15625052,1,0,1,0,1,0,
Gene content,44,Aco,Homogroups,4.0,1.00E-12,3,70001,1,1,1,1,1,1,0.9991619,1,1,0,0,1,1,1,0,11.15625052,11.15625052,11.15625052,1,0,1,0,1,0,
Gene content,44,Aco,Homogroups,4.0,1.00E-12,4,70001,1,1,1,1,1,1,1,1,1,0,0,1,1,1,0,11.15625052,11.15625052,11.15625052,1,0,1,0,1,0,
Gene content,44,Aco,Homogroups,6.0,1.00E-02,1,70001,1,1,1,1,1,1,1,1,1,0,0,0,1,1,0,0,11.15625052,11.15625052,0,0,1,0,1,0,
Gene content,44,Aco,Homogroups,6.0,1.00E-02,2,70001,1,1,1,1,1,1,1,1,1,0,0,0,1,1,0,0,11.15625052,11.15625052,0,0,1,0,1,0,
Gene content,44,Aco,Homogroups,6.0,1.00E-02,3,70001,1,1,1,1,1,1,1,1,1,0,0,0,1,1,0,0,11.15625052,11.15625052,0,0,1,0,1,0,
Gene content,44,Aco,Homogroups,6.0,1.00E-02,4,70001,1,1,1,1,1,1,1,1,1,0,0,0,1,1,0,0,11.15625052,11.15625052,0,0,1,0,1,0,
Gene content,44,Aco,Homogroups,6.0,1.00E-05,1,70001,1,1,1,1,1,1,1,1,1,0,0,0,1,1,0,0,11.15625052,11.15625052,0,0,1,0,1,0,
Gene content,44,Aco,Homogroups,6.0,1.00E-05,2,70001,1,1,1,1,1,1,1,1,1,0,0,0,1,1,0,0,11.15625052,11.15625052,0,0,1,0,1,0,
Gene content,44,Aco,Homogroups,6.0,1.00E-05,3,70001,1,1,1,1,1,1,1,1,1,0,0,0,1,1,0,0,11.15625052,11.15625052,0,0,1,0,1,0,
Gene content,44,Aco,Homogroups,6.0,1.00E-05,4,70001,1,1,1,1,1,1,1,1,1,0,0,0,1,1,0,0,11.15625052,11.15625052,0,0,1,0,1,0,
Gene content,44,Aco,Homogroups,6.0,1.00E-09,1,70001,1,1,1,1,1,1,1,1,1,0,0,0,1,1,0,0,11.15625052,11.15625052,0,0,1,0,1,0,
Gene content,44,Aco,Homogroups,6.0,1.00E-09,2,70001,1,1,1,1,1,1,1,1,1,0,0,0,1,1,0,0,11.15625052,11.15625052,0,0,1,0,1,0,
Gene content,44,Aco,Homogroups,6.0,1.00E-09,3,70001,1,1,1,1,1,1,1,1,1,0,0,0,1,1,0,0,11.15625052,11.15625052,0,0,1,0,1,0,
Gene content,44,Aco,Homogroups,6.0,1.00E-09,4,70001,1,1,1,1,1,1,1,1,1,0,0,0,1,1,0,0,11.15625052,11.15625052,0,0,1,0,1,0,
Gene content,44,Aco,Homogroups,6.0,1.00E-12,1,70001,1,1,1,1,1,1,1,1,1,0,0,0,1,1,0,0,11.15625052,11.15625052,0,0,1,0,1,0,
Gene content,44,Aco,Homogroups,6.0,1.00E-12,2,70001,1,1,1,1,1,1,1,1,1,0,0,0,1,1,0,0,11.15625052,11.15625052,0,0,1,0,1,0,
Gene content,44,Aco,Homogroups,6.0,1.00E-12,3,70001,1,1,1,1,1,1,1,1,1,0,0,0,1,1,0,0,11.15625052,11.15625052,0,0,1,0,1,0,
Gene content,44,Aco,Homogroups,6.0,1.00E-12,4,70001,1,1,1,1,1,1,1,1,1,0,0,0,1,1,0,0,11.15625052,11.15625052,0,0,1,0,1,0,
Gene content,44,Aco,Orthogroups,1.5,1.00E-02,1,100001,1,1,1,1,1,1,0.2746497,1,1,0.99644,0,1,1,1,0,11.51292546,11.51292546,11.51292546,1,0,1,0,1,0,
Gene content,44,Aco,Orthogroups,1.5,1.00E-02,2,100001,1,1,1,1,1,1,0.2928228,1,1,0.99628,0,1,1,1,0,11.51292546,11.51292546,11.51292546,1,0,1,0,1,0,
Gene content,44,Aco,Orthogroups,1.5,1.00E-02,3,100001,1,1,1,1,1,1,0.2928228,1,1,0.99628,0,1,1,1,0,11.51292546,11.51292546,11.51292546,1,0,1,0,1,0,
Gene content,44,Aco,Orthogroups,1.5,1.00E-02,4,100001,1,1,1,1,1,1,0.2956227,1,1,0.9958267,0,1,1,1,0,11.51292546,11.51292546,11.51292546,1,0,1,0,1,0,

Gene content,44,Aco,Orthogroups,1.5,1.00E-05,1,70001,1,1,1,1,1,1,0.01681873,1,1,0.9971429,0,1,1,1,0,11.15625052,11.15625052,11.15625052,1,0,1,0,1,0,
Gene content,44,Aco,Orthogroups,1.5,1.00E-05,2,70001,1,1,1,1,1,1,0.01352355,1,1,0.9958477,0,1,1,1,0,11.15625052,11.15625052,11.15625052,1,0,1,0,1,0,
Gene content,44,Aco,Orthogroups,1.5,1.00E-05,3,70001,1,1,1,1,1,1,0.01352355,1,1,0.9958477,0,1,1,1,0,11.15625052,11.15625052,11.15625052,1,0,1,0,1,0,
Gene content,44,Aco,Orthogroups,1.5,1.00E-05,4,70001,1,1,1,1,1,1,0.003561837,1,1,0.9955048,0,1,1,1,0,11.15625052,11.15625052,11.15625052,1,0,1,0,1,0,
Gene content,44,Aco,Orthogroups,1.5,1.00E-09,1,6001,1,1,1,1,1,1,0.7478338,1,1,0.9993335,0,1,1,1,0,8.699514748,8.699514748,8.699514748,1,0,1,0,1,0,
Gene content,44,Aco,Orthogroups,1.5,1.00E-09,2,6001,1,1,1,1,1,1,0.6289713,1,1,0.9988891,0,1,1,1,0,8.699514748,8.699514748,8.699514748,1,0,1,0,1,0,
Gene content,44,Aco,Orthogroups,1.5,1.00E-09,3,6001,1,1,1,1,1,1,0.6289713,1,1,0.9988891,0,1,1,1,0,8.699514748,8.699514748,8.699514748,1,0,1,0,1,0,
Gene content,44,Aco,Orthogroups,1.5,1.00E-09,4,6001,1,1,1,1,1,1,0.6694068,1,1,0.9982226,0,1,1,1,0,8.699514748,8.699514748,8.699514748,1,0,1,0,1,0,
Gene content,44,Aco,Orthogroups,1.5,1.00E-12,1,60001,1,1,1,1,1,1,0.5018555,1,1,1,0,1,1,1,0,11.00209984,11.00209984,11.00209984,1,0,1,0,1,0,
Gene content,44,Aco,Orthogroups,1.5,1.00E-12,2,60001,1,1,1,1,1,1,0.5028333,1,1,1,0,1,1,1,0,11.00209984,11.00209984,11.00209984,1,0,1,0,1,0,
Gene content,44,Aco,Orthogroups,1.5,1.00E-12,3,60001,1,1,1,1,1,1,0.5684763,1,1,1,0,1,1,1,0,11.00209984,11.00209984,11.00209984,1,0,1,0,1,0,
Gene content,44,Aco,Orthogroups,1.5,1.00E-12,4,60001,1,1,1,1,1,1,0.5219884,1,1,1,0,1,1,1,0,11.00209984,11.00209984,11.00209984,1,0,1,0,1,0,
Gene content,44,Aco,Orthogroups,2.0,1.00E-02,1,100001,1,1,1,1,1,1,0.6315516,1,1,0.3609952,0,1,1,1,0,11.51292546,11.51292546,11.51292546,1,0,1,0,1,0,
Gene content,44,Aco,Orthogroups,2.0,1.00E-02,2,100001,1,1,1,1,1,1,0.7028573,1,1,0.3602885,0,1,1,1,0,11.51292546,11.51292546,11.51292546,1,0,1,0,1,0,
Gene content,44,Aco,Orthogroups,2.0,1.00E-02,3,100001,1,1,1,1,1,1,0.7028573,1,1,0.3602885,0,1,1,1,0,11.51292546,11.51292546,11.51292546,1,0,1,0,1,0,
Gene content,44,Aco,Orthogroups,2.0,1.00E-02,4,100001,1,1,1,1,1,1,0.7028573,1,1,0.3602885,0,1,1,1,0,11.51292546,11.51292546,11.51292546,1,0,1,0,1,0,
Gene content,44,Aco,Orthogroups,2.0,1.00E-05,1,70001,1,1,1,1,1,1,0,1,1,0.9972001,0,1,1,1,0,11.15625052,11.15625052,11.15625052,1,0,1,0,1,0,
Gene content,44,Aco,Orthogroups,2.0,1.00E-05,2,70001,1,1,1,1,1,1,0,1,1,0.995162,0,1,1,1,0,11.15625052,11.15625052,11.15625052,1,0,1,0,1,0,
Gene content,44,Aco,Orthogroups,2.0,1.00E-05,3,70001,1,1,1,1,1,1,0,1,1,0.9965524,0,1,1,1,0,11.15625052,11.15625052,11.15625052,1,0,1,0,1,0,
Gene content,44,Aco,Orthogroups,2.0,1.00E-05,4,70001,1,1,1,1,1,1,0,1,1,0.9965524,0,1,1,1,0,11.15625052,11.15625052,11.15625052,1,0,1,0,1,0,
Gene content,44,Aco,Orthogroups,2.0,1.00E-09,1,70001,1,1,1,1,1,1,0.8656597,1,1,0.9982096,0,1,1,1,0,11.15625052,11.15625052,11.15625052,1,0,1,0,1,0,
Gene content,44,Aco,Orthogroups,2.0,1.00E-09,2,70001,1,1,1,1,1,1,0.9285728,1,1,0.9976762,0,1,1,1,0,11.15625052,11.15625052,11.15625052,1,0,1,0,1,0,
Gene content,44,Aco,Orthogroups,2.0,1.00E-09,3,70001,1,1,1,1,1,1,0.9285728,1,1,0.9976762,0,1,1,1,0,11.15625052,11.15625052,11.15625052,1,0,1,0,1,0,
Gene content,44,Aco,Orthogroups,2.0,1.00E-09,4,70001,1,1,1,1,1,1,0.906878,1,1,0.9977143,0,1,1,1,0,11.15625052,11.15625052,11.15625052,1,0,1,0,1,0,
Gene content,44,Aco,Orthogroups,2.0,1.00E-12,1,70001,1,1,1,1,1,1,1,1,1,1,0,1,1,1,0,11.15625052,11.15625052,11.15625052,1,0,1,0,1,0,
Gene content,44,Aco,Orthogroups,2.0,1.00E-12,2,70001,1,1,1,1,1,1,0.999981,1,1,1,0,1,1,1,0,11.15625052,11.15625052,11.15625052,1,0,1,0,1,0,
Gene content,44,Aco,Orthogroups,2.0,1.00E-12,3,70001,1,1,1,1,1,1,0.999981,1,1,1,0,1,1,1,0,11.15625052,11.15625052,11.15625052,1,0,1,0,1,0,
Gene content,44,Aco,Orthogroups,2.0,1.00E-12,4,70001,1,1,1,1,1,1,1,1,1,1,0,1,1,1,0,11.15625052,11.15625052,11.15625052,1,0,1,0,1,0,
Gene content,44,Aco,Orthogroups,2.5,1.00E-02,1,100001,1,1,1,1,1,1,0.7808563,1,1,0.04710604,0,1,1,1,0,11.51292546,11.51292546,11.51292546,1,0,1,0,1,0,
Gene content,44,Aco,Orthogroups,2.5,1.00E-02,2,100001,1,1,1,1,1,1,0.8092559,1,1,0.04663938,0,1,1,1,0,11.51292546,11.51292546,11.51292546,1,0,1,0,1,0,
Gene content,44,Aco,Orthogroups,2.5,1.00E-02,3,100001,1,1,1,1,1,1,0.8800416,1,1,0.04458607,0,1,1,1,0,11.51292546,11.51292546,11.51292546,1,0,1,0,1,0,
Gene content,44,Aco,Orthogroups,2.5,1.00E-02,4,100001,1,1,1,1,1,1,0.9304276,1,1,0.04541273,0,1,1,1,0,11.51292546,11.51292546,11.51292546,1,0,1,0,1,0,
Gene content,44,Aco,Orthogroups,2.5,1.00E-05,1,70001,1,1,1,1,1,1,0.03942782,1,1,0.9729719,0,1,1,1,0,11.15625052,11.15625052,11.15625052,1,0,1,0,1,0,
Gene content,44,Aco,Orthogroups,2.5,1.00E-05,2,70001,1,1,1,1,1,1,0,1,1,0.9761719,0,1,1,1,0,11.15625052,11.15625052,11.15625052,1,0,1,0,1,0,
Gene content,44,Aco,Orthogroups,2.5,1.00E-05,3,70001,1,1,1,1,1,1,0,1,1,0.9761719,0,1,1,1,0,11.15625052,11.15625052,11.15625052,1,0,1,0,1,0,
Gene content,44,Aco,Orthogroups,2.5,1.00E-05,4,70001,1,1,1,1,1,1,0.02234243,1,1,0.9723624,0,1,1,1,0,11.15625052,11.15625052,11.15625052,1,0,1,0,1,0,
Gene content,44,Aco,Orthogroups,2.5,1.00E-09,1,55746,1,1,1,1,1,1,1,1,1,0.9995456,0,1,1,1,0,10.928543,10.928543,10.928543,1,0,1,0,1,0,
Gene content,44,Aco,Orthogroups,2.5,1.00E-09,2,56082,1,1,1,1,1,1,1,1,1,0.9994056,0,1,1,1,0,10.93455235,10.93455235,10.93455235,1,0,1,0,1,0,
Gene content,44,Aco,Orthogroups,2.5,1.00E-09,3,56989,1,1,1,1,1,1,1,1,1,0.9996257,0,1,1,1,0,10.950596,10.950596,10.950596,1,0,1,0,1,0,
Gene content,44,Aco,Orthogroups,2.5,1.00E-09,4,61499,1,1,1,1,1,1,1,1,1,0.9998699,0,1,1,1,0,11.02675993,11.02675993,11.02675993,1,0,1,0,1,0,
Gene content,44,Aco,Orthogroups,2.5,1.00E-12,1,70001,1,1,1,1,1,1,1,1,1,0.9999048,0,1,1,1,0,11.15625052,11.15625052,11.15625052,1,0,1,0,1,0,
Gene content,44,Aco,Orthogroups,2.5,1.00E-12,2,70001,1,1,1,1,1,1,1,1,1,0.9998286,0,1,1,1,0,11.15625052,11.15625052,11.15625052,1,0,1,0,1,0,
Gene content,44,Aco,Orthogroups,2.5,1.00E-12,3,70001,1,1,1,1,1,1,1,1,1,0.9998286,0,1,1,1,0,11.15625052,11.15625052,11.15625052,1,0,1,0,1,0,
Gene content,44,Aco,Orthogroups,2.5,1.00E-12,4,70001,1,1,1,1,1,1,1,1,1,0.9999619,0,1,1,1,0,11.15625052,11.15625052,11.15625052,1,0,1,0,1,0,
Gene content,44,Aco,Orthogroups,4.0,1.00E-02,1,100001,1,1,1,1,1,1,1,1,1,0.0004533273,0,1,1,1,0,11.51292546,11.51292546,11.51292546,1,0,1,0,1,0,
Gene content,44,Aco,Orthogroups,4.0,1.00E-02,2,100001,1,1,1,1,1,1,1,1,1,0.0003733284,0,1,1,1,0,11.51292546,11.51292546,11.51292546,1,0,1,0,1,0,
Gene content,44,Aco,Orthogroups,4.0,1.00E-02,3,100001,1,1,1,1,1,1,1,1,1,0.0004533273,0,1,1,1,0,11.51292546,11.51292546,11.51292546,1,0,1,0,1,0,
Gene content,44,Aco,Orthogroups,4.0,1.00E-02,4,100001,1,1,1,1,1,1,0.9970934,1,1,0.001066652,0,1,1,1,0,11.51292546,11.51292546,11.51292546,1,0,1,0,1,0,
Gene content,44,Aco,Orthogroups,4.0,1.00E-05,1,70001,1,1,1,1,1,1,0.9999429,1,1,0.6019314,0,1,1,1,0,11.15625052,11.15625052,11.15625052,1,0,1,0,1,0,
Gene content,44,Aco,Orthogroups,4.0,1.00E-05,2,70001,1,1,1,1,1,1,0.9977905,1,1,0.6213596,0,1,1,1,0,11.15625052,11.15625052,11.15625052,1,0,1,0,1,0,
Gene content,44,Aco,Orthogroups,4.0,1.00E-05,3,70001,1,1,1,1,1,1,0.9977524,1,1,0.6191311,0,1,1,1,0,11.15625052,11.15625052,11.15625052,1,0,1,0,1,0,
Gene content,44,Aco,Orthogroups,4.0,1.00E-05,4,70001,1,1,1,1,1,1,0.9969715,1,1,0.6152454,0,1,1,1,0,11.15625052,11.15625052,11.15625052,1,0,1,0,1,0,
Gene content,44,Aco,Orthogroups,4.0,1.00E-09,1,70001,1,1,1,1,1,1,1,1,1,0.846841,0,1,1,1,0,11.15625052,11.15625052,11.15625052,1,0,1,0,1,0,
Gene content,44,Aco,Orthogroups,4.0,1.00E-09,2,70001,1,1,1,1,1,1,1,1,1,0.8409935,0,1,1,1,0,11.15625052,11.15625052,11.15625052,1,0,1,0,1,0,
Gene content,44,Aco,Orthogroups,4.0,1.00E-09,3,70001,1,1,1,1,1,1,1,1,1,0.8409935,0,1,1,1,0,11.15625052,11.15625052,11.15625052,1,0,1,0,1,0,
Gene content,44,Aco,Orthogroups,4.0,1.00E-09,4,70001,1,1,1,1,1,1,1,1,1,0.8400602,0,1,1,1,0,11.15625052,11.15625052,11.15625052,1,0,1,0,1,0,
Gene content,44,Aco,Orthogroups,4.0,1.00E-12,1,70001,1,1,1,1,1,1,1,1,1,0.9886097,0,1,1,1,0,11.15625052,11.15625052,11.15625052,1,0,1,0,1,0,
Gene content,44,Aco,Orthogroups,4.0,1.00E-12,2,70001,1,1,1,1,1,1,1,1,1,0.9896954,0,1,1,1,0,11.15625052,11.15625052,11.15625052,1,0,1,0,1,0,
Gene content,44,Aco,Orthogroups,4.0,1.00E-12,3,70001,1,1,1,1,1,1,1,1,1,0.9896954,0,1,1,1,0,11.15625052,11.15625052,11.15625052,1,0,1,0,1,0,
Gene content,44,Aco,Orthogroups,4.0,1.00E-12,4,70001,1,1,1,1,1,1,1,1,1,0.9896954,0,1,1,1,0,11.15625052,11.15625052,11.15625052,1,0,1,0,1,0,
Gene content,44,Aco,Orthogroups,6.0,1.00E-02,1,100001,1,1,1,1,1,1,1,1,1,0.102612,0,1,1,1,0,11.51292546,11.51292546,11.51292546,1,0,1,0,1,0,
Gene content,44,Aco,Orthogroups,6.0,1.00E-02,2,100001,1,1,1,1,1,1,1,1,1,0.1012253,0,1,1,1,0,11.51292546,11.51292546,11.51292546,1,0,1,0,1,0,
Gene content,44,Aco,Orthogroups,6.0,1.00E-02,3,100001,1,1,1,1,1,1,1,1,1,0.09686538,0,1,1,1,0,11.51292546,11.51292546,11.51292546,1,0,1,0,1,0,
Gene content,44,Aco,Orthogroups,6.0,1.00E-02,4,100001,1,1,1,1,1,1,1,1,1,0.1057986,0,1,1,1,0,11.51292546,11.51292546,11.51292546,1,0,1,0,1,0,
Gene content,44,Aco,Orthogroups,6.0,1.00E-05,1,70001,1,1,1,1,1,1,1,1,1,0.2634045,0,1,1,1,0,11.15625052,11.15625052,11.15625052,1,0,1,0,1,0,
Gene content,44,Aco,Orthogroups,6.0,1.00E-05,2,70001,1,1,1,1,1,1,1,1,1,0.2706615,0,1,1,1,0,11.15625052,11.15625052,11.15625052,1,0,1,0,1,0,
Gene content,44,Aco,Orthogroups,6.0,1.00E-05,3,70001,1,1,1,1,1,1,1,1,1,0.27689,0,1,1,1,0,11.15625052,11.15625052,11.15625052,1,0,1,0,1,0,
Gene content,44,Aco,Orthogroups,6.0,1.00E-05,4,70001,1,1,1,1,1,1,1,1,1,0.2661854,0,1,1,1,0,11.15625052,11.15625052,11.15625052,1,0,1,0,1,0,
Gene content,44,Aco,Orthogroups,6.0,1.00E-09,1,70001,1,1,1,1,1,1,1,1,1,0.919792,0,1,1,1,0,11.15625052,11.15625052,11.15625052,1,0,1,0,1,0,
Gene content,44,Aco,Orthogroups,6.0,1.00E-09,2,70001,1,1,1,1,1,1,1,1,1,0.9033352,0,1,1,1,0,11.15625052,11.15625052,11.15625052,1,0,1,0,1,0,
Gene content,44,Aco,Orthogroups,6.0,1.00E-09,3,70001,1,1,1,1,1,1,1,1,1,0.906897,0,1,1,1,0,11.15625052,11.15625052,11.15625052,1,0,1,0,1,0,
Gene content,44,Aco,Orthogroups,6.0,1.00E-09,4,70001,1,1,1,1,1,1,1,1,1,0.9021162,0,1,1,1,0,11.15625052,11.15625052,11.15625052,1,0,1,0,1,0,
Gene content,44,Aco,Orthogroups,6.0,1.00E-12,1,70001,1,1,1,1,1,1,1,1,1,0.987086,0,1,1,1,0,11.15625052,11.15625052,11.15625052,1,0,1,0,1,0,
Gene content,44,Aco,Orthogroups,6.0,1.00E-12,2,70001,1,1,1,1,1,1,1,1,1,0.9921335,0,1,1,1,0,11.15625052,11.15625052,11.15625052,1,0,1,0,1,0,
Gene content,44,Aco,Orthogroups,6.0,1.00E-12,3,70001,1,1,1,1,1,1,1,1,1,0.9909906,0,1,1,1,0,11.15625052,11.15625052,11.15625052,1,0,1,0,1,0,
Gene content,44,Aco,Orthogroups,6.0,1.00E-12,4,70001,1,1,1,1,1,1,1,1,1,0.9911811,0,1,1,1,0,11.15625052,11.15625052,11.15625052,1,0,1,0,1,0,
Gene content,47,Opi,Homogroups,1.5,1.00E-02,1,71563,1,1,1,1,1,1,0,1,1,0.1744639,0,1,1,1,0,11.17831949,11.17831949,11.17831949,1,0,1,0,1,0,
Gene content,47,Opi,Homogroups,1.5,1.00E-02,2,75017,1,1,1,1,1,1,0,1,1,0.1654373,0,1,1,1,0,11.2254567,11.2254567,11.2254567,1,0,1,0,1,0,
Gene content,47,Opi,Homogroups,1.5,1.00E-02,3,88653,1,1,1,1,1,1,0,1,1,0.1764626,0,1,1,1,0,11.39247387,11.39247387,11.39247387,1,0,1,0,1,0,
Gene content,47,Opi,Homogroups,1.5,1.00E-02,4,88273,1,1,1,1,1,1,0,1,1,0.1767389,0,1,1,1,0,11.38817824,11.38817824,11.38817824,1,0,1,0,1,0,
Gene content,47,Opi,Homogroups,1.5,1.00E-05,1,81835,1,1,1,1,1,1,0,1,1,0.3315411,0,1,1,1,0,11.31244808,11.31244808,11.31244808,1,0,1,0,1,0,
Gene content,47,Opi,Homogroups,1.5,1.00E-05,2,78073,1,1,1,1,1,1,0,1,1,0.3434549,0,1,1,1,0,11.26538676,11.26538676,11.26538676,1,0,1,0,1,0,

Gene content,47,Opi,Homogroups,1.5,1.00E-05,3,82008,1,1,1,1,1,1,0,1,1,0.3438851,0,1,1,1,0,11.31455989,11.31455989,11.31455989,1,0,1,0,1,0,
Gene content,47,Opi,Homogroups,1.5,1.00E-05,4,95599,1,1,1,1,1,1,0,1,1,0.3446025,0,1,1,1,0,11.46790718,11.46790718,11.46790718,1,0,1,0,1,0,
Gene content,47,Opi,Homogroups,1.5,1.00E-09,1,93534,1,1,1,1,1,1,0.07851634,1,1,0.9594731,0,1,1,1,0,11.44606959,11.44606959,11.44606959,1,0,1,0,1,0,
Gene content,47,Opi,Homogroups,1.5,1.00E-09,2,97986,1,1,1,1,1,1,0.07552048,1,1,0.9642536,0,1,1,1,0,11.49256968,11.49256968,11.49256968,1,0,1,0,1,0,
Gene content,47,Opi,Homogroups,1.5,1.00E-09,3,87507,1,1,1,1,1,1,0.05445597,1,1,0.9570934,0,1,1,1,0,11.37946264,11.37946264,11.37946264,1,0,1,0,1,0,
Gene content,47,Opi,Homogroups,1.5,1.00E-09,4,94127,1,1,1,1,1,1,0.04459176,1,1,0.96191,0,1,1,1,0,11.45238959,11.45238959,11.45238959,1,0,1,0,1,0,
Gene content,47,Opi,Homogroups,1.5,1.00E-12,1,100001,1,1,1,1,1,1,0.0006799909,1,1,0.9681604,0,1,1,1,0,11.51292546,11.51292546,11.51292546,1,0,1,0,1,0,
Gene content,47,Opi,Homogroups,1.5,1.00E-12,2,100001,1,1,1,1,1,1,0,1,1,0.9676538,0,1,1,1,0,11.51292546,11.51292546,11.51292546,1,0,1,0,1,0,
Gene content,47,Opi,Homogroups,1.5,1.00E-12,3,100001,1,1,1,1,1,1,0,1,1,0.9622138,0,1,1,1,0,11.51292546,11.51292546,11.51292546,1,0,1,0,1,0,
Gene content,47,Opi,Homogroups,1.5,1.00E-12,4,100001,1,1,1,1,1,1,0,1,1,0.9720404,0,1,1,1,0,11.51292546,11.51292546,11.51292546,1,0,1,0,1,0,
Gene content,47,Opi,Homogroups,2.0,1.00E-02,1,70001,1,1,1,1,1,1,0,1,1,0.000819032,0,1,1,1,0,11.15625052,11.15625052,11.15625052,1,0,1,0,1,0,
Gene content,47,Opi,Homogroups,2.0,1.00E-02,2,70001,1,1,1,1,1,1,0,1,1,0.000819032,0,1,1,1,0,11.15625052,11.15625052,11.15625052,1,0,1,0,1,0,
Gene content,47,Opi,Homogroups,2.0,1.00E-02,3,70001,1,1,1,1,1,1,0,1,1,0.000819032,0,1,1,1,0,11.15625052,11.15625052,11.15625052,1,0,1,0,1,0,
Gene content,47,Opi,Homogroups,2.0,1.00E-02,4,70001,1,1,1,1,1,1,0,1,1,0.0007809375,0,1,1,1,0,11.15625052,11.15625052,11.15625052,1,0,1,0,1,0,
Gene content,47,Opi,Homogroups,2.0,1.00E-05,1,70001,1,1,1,1,1,1,0,1,1,0.02213291,0,1,1,1,0,11.15625052,11.15625052,11.15625052,1,0,1,0,1,0,
Gene content,47,Opi,Homogroups,2.0,1.00E-05,2,70001,1,1,1,1,1,1,0,1,1,0.01822822,0,1,1,1,0,11.15625052,11.15625052,11.15625052,1,0,1,0,1,0,
Gene content,47,Opi,Homogroups,2.0,1.00E-05,3,70001,1,1,1,1,1,1,0,1,1,0.01908535,0,1,1,1,0,11.15625052,11.15625052,11.15625052,1,0,1,0,1,0,
Gene content,47,Opi,Homogroups,2.0,1.00E-05,4,70001,1,1,1,1,1,1,0,1,1,0.01634255,0,1,1,1,0,11.15625052,11.15625052,11.15625052,1,0,1,0,1,0,
Gene content,47,Opi,Homogroups,2.0,1.00E-09,1,70001,1,1,1,1,1,1,1,1,1,0.1998248,0,1,1,1,0,11.15625052,11.15625052,11.15625052,1,0,1,0,1,0,
Gene content,47,Opi,Homogroups,2.0,1.00E-09,2,70001,1,1,1,1,1,1,1,1,1,0.1891964,0,1,1,1,0,11.15625052,11.15625052,11.15625052,1,0,1,0,1,0,
Gene content,47,Opi,Homogroups,2.0,1.00E-09,3,70001,1,1,1,1,1,1,0.9999619,1,1,0.202339,0,1,1,1,0,11.15625052,11.15625052,11.15625052,1,0,1,0,1,0,
Gene content,47,Opi,Homogroups,2.0,1.00E-09,4,70001,1,1,1,1,1,1,1,1,1,0.2204529,0,1,1,1,0,11.15625052,11.15625052,11.15625052,1,0,1,0,1,0,
Gene content,47,Opi,Homogroups,2.0,1.00E-12,1,70001,1,1,1,1,1,1,0.9269347,1,1,9.52E-05,0,1,1,1,0,11.15625052,11.15625052,11.15625052,1,0,1,0,1,0,
Gene content,47,Opi,Homogroups,2.0,1.00E-12,2,70001,1,1,1,1,1,1,0.6237595,1,1,0.0005904649,0,1,1,1,0,11.15625052,11.15625052,11.15625052,1,0,1,0,1,0,
Gene content,47,Opi,Homogroups,2.0,1.00E-12,3,70001,1,1,1,1,1,1,0.72351,1,1,0.001638064,0,1,1,1,0,11.15625052,11.15625052,11.15625052,1,0,1,0,1,0,
Gene content,47,Opi,Homogroups,2.0,1.00E-12,4,70001,1,1,1,1,1,1,0.77669,1,1,7.62E-05,0,1,1,1,0,11.15625052,11.15625052,11.15625052,1,0,1,0,1,0,
Gene content,47,Opi,Homogroups,2.5,1.00E-02,1,69176,1,1,1,1,1,1,0.004568058,1,1,0,0,0,1,1,0,0,11.1443948,11.1443948,0,0,1,0,1,0,
Gene content,47,Opi,Homogroups,2.5,1.00E-02,2,65064,1,1,1,1,1,1,0,1,1,0,0,0,1,1,0,0,11.08311131,11.08311131,0,0,1,0,1,0,
Gene content,47,Opi,Homogroups,2.5,1.00E-02,3,71355,1,1,1,1,1,1,0,1,1,0,0,0,1,1,0,0,11.17540868,11.17540868,0,0,1,0,1,0,
Gene content,47,Opi,Homogroups,2.5,1.00E-02,4,71406,1,1,1,1,1,1,0,1,1,0,0,0,1,1,0,0,11.17612317,11.17612317,0,0,1,0,1,0,
Gene content,47,Opi,Homogroups,2.5,1.00E-05,1,70619,1,1,1,1,1,1,0,1,1,0.0004531294,0,1,1,1,0,11.16504035,11.16504035,11.16504035,1,0,1,0,1,0,
Gene content,47,Opi,Homogroups,2.5,1.00E-05,2,71171,1,1,1,1,1,1,0,1,1,0.0002997433,0,1,1,1,0,11.17282666,11.17282666,11.17282666,1,0,1,0,1,0,
Gene content,47,Opi,Homogroups,2.5,1.00E-05,3,71296,1,1,1,1,1,1,0,1,1,0.000299222,0,1,1,1,0,11.17458148,11.17458148,11.17458148,1,0,1,0,1,0,
Gene content,47,Opi,Homogroups,2.5,1.00E-05,4,68123,1,1,1,1,1,1,0,1,1,0.0003131544,0,1,1,1,0,11.12905549,11.12905549,11.12905549,1,0,1,0,1,0,
Gene content,47,Opi,Homogroups,2.5,1.00E-09,1,75450,1,1,1,1,1,1,0.1198664,1,1,0.0002827455,0,1,1,1,0,11.23121221,11.23121221,11.23121221,1,0,1,0,1,0,
Gene content,47,Opi,Homogroups,2.5,1.00E-09,2,75413,1,1,1,1,1,1,0.1627475,1,1,0.0001944837,0,1,1,1,0,11.23072169,11.23072169,11.23072169,1,0,1,0,1,0,
Gene content,47,Opi,Homogroups,2.5,1.00E-09,3,76800,1,1,1,1,1,1,0.01230903,1,1,0.0002256944,0,1,1,1,0,11.2489469,11.2489469,11.2489469,1,0,1,0,1,0,
Gene content,47,Opi,Homogroups,2.5,1.00E-09,4,81078,1,1,1,1,1,1,0.1845286,1,1,0.0001315595,0,1,1,1,0,11.3031546,11.3031546,11.3031546,1,0,1,0,1,0,
Gene content,47,Opi,Homogroups,2.5,1.00E-12,1,82174,1,1,1,1,1,1,4.87E-05,1,1,0.006327984,0,1,1,1,0,11.31658206,11.31658206,11.31658206,1,0,1,0,1,0,
Gene content,47,Opi,Homogroups,2.5,1.00E-12,2,78042,1,1,1,1,1,1,0.01469282,1,1,0.00596255,0,1,1,1,0,11.26498961,11.26498961,11.26498961,1,0,1,0,1,0,
Gene content,47,Opi,Homogroups,2.5,1.00E-12,3,96699,1,1,1,1,1,1,0.02645984,1,1,0.004867287,0,1,1,1,0,11.479348,11.479348,11.479348,1,0,1,0,1,0,
Gene content,47,Opi,Homogroups,2.5,1.00E-12,4,96673,1,1,1,1,1,1,0.02646714,1,1,0.00486863,0,1,1,1,0,11.47907908,11.47907908,11.47907908,1,0,1,0,1,0,
Gene content,47,Opi,Homogroups,4.0,1.00E-02,1,70001,1,1,1,1,1,1,0.7018914,1,1,0,0,0,1,1,0,0,11.15625052,11.15625052,0,0,1,0,1,0,
Gene content,47,Opi,Homogroups,4.0,1.00E-02,2,70001,1,1,1,1,1,1,0.6411116,1,1,0,0,0,0.999981,1,0,0,11.15625052,10.87105258,0,0,1,0,1,0,
Gene content,47,Opi,Homogroups,4.0,1.00E-02,3,70001,1,1,1,1,1,1,0.4709434,1,1,0,0,0,1,1,0,0,11.15625052,11.15625052,0,0,1,0,1,0,
Gene content,47,Opi,Homogroups,4.0,1.00E-02,4,70001,1,1,1,1,1,1,0.5403516,1,1,0,0,0,1,1,0,0,11.15625052,11.15625052,0,0,1,0,1,0,
Gene content,47,Opi,Homogroups,4.0,1.00E-05,1,70001,1,1,1,1,1,1,0.5749224,1,1,0,0,0,0,1,0,0,11.15625052,-11.15625052,0,0,1,0,0,1,
Gene content,47,Opi,Homogroups,4.0,1.00E-05,2,70001,1,1,1,1,1,1,0.3926592,1,1,0,0,0,0,1,0,0,11.15625052,-11.15625052,0,0,1,0,0,1,
Gene content,47,Opi,Homogroups,4.0,1.00E-05,3,70001,1,1,1,1,1,1,0.4554389,1,1,0,0,0,0,1,0,0,11.15625052,-11.15625052,0,0,1,0,0,1,
Gene content,47,Opi,Homogroups,4.0,1.00E-05,4,70001,1,1,1,1,1,1,0.1298832,1,1,0,0,0,0,1,0,0,11.15625052,-11.15625052,0,0,1,0,0,1,
Gene content,47,Opi,Homogroups,4.0,1.00E-09,1,70001,1,1,1,1,1,1,0.9636578,1,1,0,0,0,0,1,0,0,11.15625052,-11.15625052,0,0,1,0,0,1,
Gene content,47,Opi,Homogroups,4.0,1.00E-09,2,70001,1,1,1,1,1,1,0.9840574,1,1,0,0,0,0,1,0,0,11.15625052,-11.15625052,0,0,1,0,0,1,
Gene content,47,Opi,Homogroups,4.0,1.00E-09,3,70001,1,1,1,1,1,1,0.999981,1,1,0,0,0,0,1,0,0,11.15625052,-11.15625052,0,0,1,0,0,1,
Gene content,47,Opi,Homogroups,4.0,1.00E-09,4,70001,1,1,1,1,1,1,1,1,1,0,0,0,0,1,0,0,11.15625052,-11.15625052,0,0,1,0,0,1,
Gene content,47,Opi,Homogroups,4.0,1.00E-12,1,70001,1,1,1,1,1,1,0.9955048,1,1,0,0,1,1,1,0,11.15625052,11.15625052,11.15625052,1,0,1,0,1,0,
Gene content,47,Opi,Homogroups,4.0,1.00E-12,2,70001,1,1,1,1,1,1,0.9788956,1,1,0,0,1,1,1,0,11.15625052,11.15625052,11.15625052,1,0,1,0,1,0,
Gene content,47,Opi,Homogroups,4.0,1.00E-12,3,70001,1,1,1,1,1,1,1,1,1,0,0,1,1,1,0,11.15625052,11.15625052,11.15625052,1,0,1,0,1,0,
Gene content,47,Opi,Homogroups,4.0,1.00E-12,4,70001,1,1,1,1,1,1,1,1,1,0,0,1,1,1,0,11.15625052,11.15625052,11.15625052,1,0,1,0,1,0,
Gene content,47,Opi,Homogroups,6.0,1.00E-02,1,70001,1,1,1,1,1,1,0.7019866,1,1,0,0,0,0,1,0,0,11.15625052,-11.15625052,0,0,1,0,0,1,
Gene content,47,Opi,Homogroups,6.0,1.00E-02,2,70001,1,1,1,1,1,1,0.7601569,1,1,0,0,0,0,1,0,0,11.15625052,-11.15625052,0,0,1,0,0,1,
Gene content,47,Opi,Homogroups,6.0,1.00E-02,3,70001,1,1,1,1,1,1,0.7986895,1,1,0,0,0,0,1,0,0,11.15625052,-11.15625052,0,0,1,0,0,1,
Gene content,47,Opi,Homogroups,6.0,1.00E-02,4,70001,1,1,1,1,1,1,0.7878136,1,1,0,0,0,0,1,0,0,11.15625052,-11.15625052,0,0,1,0,0,1,
Gene content,47,Opi,Homogroups,6.0,1.00E-05,1,70001,1,1,1,1,1,1,1,1,1,0,0,0,0,1,0,0,11.15625052,-11.15625052,0,0,1,0,0,1,
Gene content,47,Opi,Homogroups,6.0,1.00E-05,2,70001,1,1,1,1,1,1,1,1,1,0,0,0,0,1,0,0,11.15625052,-11.15625052,0,0,1,0,0,1,
Gene content,47,Opi,Homogroups,6.0,1.00E-05,3,70001,1,1,1,1,1,1,1,1,1,0,0,0,0,1,0,0,11.15625052,-11.15625052,0,0,1,0,0,1,
Gene content,47,Opi,Homogroups,6.0,1.00E-05,4,70001,1,1,1,1,1,1,1,1,1,0,0,0,0,1,0,0,11.15625052,-11.15625052,0,0,1,0,0,1,
Gene content,47,Opi,Homogroups,6.0,1.00E-09,1,70001,1,1,1,1,1,1,1,1,1,0,0,0,0,1,0,0,11.15625052,-11.15625052,0,0,1,0,0,1,
Gene content,47,Opi,Homogroups,6.0,1.00E-09,2,70001,1,1,1,1,1,1,1,1,1,0,0,0,0,1,0,0,11.15625052,-11.15625052,0,0,1,0,0,1,
Gene content,47,Opi,Homogroups,6.0,1.00E-09,3,70001,1,1,1,1,1,1,1,1,1,0,0,0,0,1,0,0,11.15625052,-11.15625052,0,0,1,0,0,1,
Gene content,47,Opi,Homogroups,6.0,1.00E-09,4,70001,1,1,1,1,1,1,1,1,1,0,0,0,0,1,0,0,11.15625052,-11.15625052,0,0,1,0,0,1,
Gene content,47,Opi,Homogroups,6.0,1.00E-12,1,70001,1,1,1,1,1,1,1,1,1,0,0,0,0,1,0,0,11.15625052,-11.15625052,0,0,1,0,0,1,
Gene content,47,Opi,Homogroups,6.0,1.00E-12,2,70001,1,1,1,1,1,1,1,1,1,0,0,0,0,1,0,0,11.15625052,-11.15625052,0,0,1,0,0,1,
Gene content,47,Opi,Homogroups,6.0,1.00E-12,3,70001,1,1,1,1,1,1,1,1,1,0,0,0,0,1,0,0,11.15625052,-11.15625052,0,0,1,0,0,1,
Gene content,47,Opi,Homogroups,6.0,1.00E-12,4,70001,1,1,1,1,1,1,1,1,1,0,0,0,0,1,0,0,11.15625052,-11.15625052,0,0,1,0,0,1,
Gene content,47,Opi,Orthogroups,1.5,1.00E-02,1,8001,1,1,1,1,1,1,0.1469755,1,1,0.9758374,0,1,1,1,0,8.987196821,8.987196821,8.987196821,1,0,1,0,1,0,
Gene content,47,Opi,Orthogroups,1.5,1.00E-02,2,6996,1,1,1,1,1,1,0.1576139,1,1,0.9801791,0,1,1,1,0,8.852950887,8.852950887,8.852950887,1,0,1,0,1,0,
Gene content,47,Opi,Orthogroups,1.5,1.00E-02,3,7004,1,1,1,1,1,1,0.1574338,1,1,0.9802018,0,1,1,1,0,8.854093908,8.854093908,8.854093908,1,0,1,0,1,0,
Gene content,47,Opi,Orthogroups,1.5,1.00E-02,4,6976,1,1,1,1,1,1,0.1142966,1,1,0.9808869,0,1,1,1,0,8.850087607,8.850087607,8.850087607,1,0,1,0,1,0,
Gene content,47,Opi,Orthogroups,1.5,1.00E-05,1,60001,1,1,1,1,1,1,0,1,1,0.9988667,0,1,1,1,0,11.00209984,11.00209984,11.00209984,1,0,1,0,1,0,
Gene content,47,Opi,Orthogroups,1.5,1.00E-05,2,60001,1,1,1,1,1,1,8.89E-05,1,1,0.9988222,0,1,1,1,0,11.00209984,11.00209984,11.00209984,1,0,1,0,1,0,
Gene content,47,Opi,Orthogroups,1.5,1.00E-05,3,60001,1,1,1,1,1,1,0.00475545,1,1,0.9986889,0,1,1,1,0,11.00209984,11.00209984,11.00209984,1,0,1,0,1,0,
Gene content,47,Opi,Orthogroups,1.5,1.00E-05,4,60001,1,1,1,1,1,1,0,1,1,0.9994667,0,1,1,1,0,11.00209984,11.00209984,11.00209984,1,0,1,0,1,0,

Gene content,47,Opi,Orthogroups,1.5,1.00E-09,1,60001,1,1,1,1,1,1,0.2867492,1,1,0.9999111,0,1,1,1,0,11.00209984,11.00209984,11.00209984,1,0,1,0,1,0,
Gene content,47,Opi,Orthogroups,1.5,1.00E-09,2,60001,1,1,1,1,1,1,0.1659963,1,1,0.9997111,0,1,1,1,0,11.00209984,11.00209984,11.00209984,1,0,1,0,1,0,
Gene content,47,Opi,Orthogroups,1.5,1.00E-09,3,60001,1,1,1,1,1,1,0.1659963,1,1,0.9997111,0,1,1,1,0,11.00209984,11.00209984,11.00209984,1,0,1,0,1,0,
Gene content,47,Opi,Orthogroups,1.5,1.00E-09,4,60001,1,1,1,1,1,1,0.1659963,1,1,0.9997111,0,1,1,1,0,11.00209984,11.00209984,11.00209984,1,0,1,0,1,0,
Gene content,47,Opi,Orthogroups,1.5,1.00E-12,1,60001,1,1,1,1,1,1,0.2622831,1,1,0.9997556,0,1,1,1,0,11.00209984,11.00209984,11.00209984,1,0,1,0,1,0,
Gene content,47,Opi,Orthogroups,1.5,1.00E-12,2,60001,1,1,1,1,1,1,0.2380836,1,1,0.9997333,0,1,1,1,0,11.00209984,11.00209984,11.00209984,1,0,1,0,1,0,
Gene content,47,Opi,Orthogroups,1.5,1.00E-12,3,60001,1,1,1,1,1,1,0.160352,1,1,0.9997778,0,1,1,1,0,11.00209984,11.00209984,11.00209984,1,0,1,0,1,0,
Gene content,47,Opi,Orthogroups,1.5,1.00E-12,4,60001,1,1,1,1,1,1,0.225795,1,1,0.9994889,0,1,1,1,0,11.00209984,11.00209984,11.00209984,1,0,1,0,1,0,
Gene content,47,Opi,Orthogroups,2.0,1.00E-02,1,60001,1,1,1,1,1,1,0.003399924,1,1,0.2923935,0,1,0,1,0,11.00209984,11.00209984,-11.00209984,1,0,1,0,0,1,
Gene content,47,Opi,Orthogroups,2.0,1.00E-02,2,60001,1,1,1,1,1,1,0.02164396,1,1,0.2839048,0,1,0,1,0,11.00209984,11.00209984,-11.00209984,1,0,1,0,0,1,
Gene content,47,Opi,Orthogroups,2.0,1.00E-02,3,60001,1,1,1,1,1,1,0.02164396,1,1,0.2839048,0,1,0,1,0,11.00209984,11.00209984,-11.00209984,1,0,1,0,0,1,
Gene content,47,Opi,Orthogroups,2.0,1.00E-02,4,60001,1,1,1,1,1,1,0.01424413,1,1,0.2995489,0,1,0,1,0,11.00209984,11.00209984,-11.00209984,1,0,1,0,0,1,
Gene content,47,Opi,Orthogroups,2.0,1.00E-05,1,60001,1,1,1,1,1,1,0,1,1,0.9590009,0,1,0,1,0,11.00209984,11.00209984,-11.00209984,1,0,1,0,0,1,
Gene content,47,Opi,Orthogroups,2.0,1.00E-05,2,60001,1,1,1,1,1,1,0,1,1,0.9737339,0,1,0,1,0,11.00209984,11.00209984,-11.00209984,1,0,1,0,0,1,
Gene content,47,Opi,Orthogroups,2.0,1.00E-05,3,60001,1,1,1,1,1,1,0,1,1,0.9634008,0,1,0,1,0,11.00209984,11.00209984,-11.00209984,1,0,1,0,0,1,
Gene content,47,Opi,Orthogroups,2.0,1.00E-05,4,60001,1,1,1,1,1,1,0,1,1,0.9692673,0,1,0,1,0,11.00209984,11.00209984,-11.00209984,1,0,1,0,0,1,
Gene content,47,Opi,Orthogroups,2.0,1.00E-09,1,60001,1,1,1,1,1,1,0.7814271,1,1,0.9992222,0,1,0,1,0,11.00209984,11.00209984,-11.00209984,1,0,1,0,0,1,
Gene content,47,Opi,Orthogroups,2.0,1.00E-09,2,60001,1,1,1,1,1,1,0.9404458,1,1,0.9995778,0,1,0,1,0,11.00209984,11.00209984,-11.00209984,1,0,1,0,0,1,
Gene content,47,Opi,Orthogroups,2.0,1.00E-09,3,60001,1,1,1,1,1,1,0.9404458,1,1,0.9995778,0,1,0,1,0,11.00209984,11.00209984,-11.00209984,1,0,1,0,0,1,
Gene content,47,Opi,Orthogroups,2.0,1.00E-09,4,60001,1,1,1,1,1,1,0.8593142,1,1,0.9988,0,1,0,1,0,11.00209984,11.00209984,-11.00209984,1,0,1,0,0,1,
Gene content,47,Opi,Orthogroups,2.0,1.00E-12,1,60001,1,1,1,1,1,1,0.9564454,1,1,0.9998444,0,1,1,1,0,11.00209984,11.00209984,11.00209984,1,0,1,0,1,0,
Gene content,47,Opi,Orthogroups,2.0,1.00E-12,2,60001,1,1,1,1,1,1,0.9969112,1,1,1,0,1,1,1,0,11.00209984,11.00209984,11.00209984,1,0,1,0,1,0,
Gene content,47,Opi,Orthogroups,2.0,1.00E-12,3,60001,1,1,1,1,1,1,0.9919113,1,1,1,0,1,1,1,0,11.00209984,11.00209984,11.00209984,1,0,1,0,1,0,
Gene content,47,Opi,Orthogroups,2.0,1.00E-12,4,60001,1,1,1,1,1,1,0.9597564,1,1,1,0,1,1,1,0,11.00209984,11.00209984,11.00209984,1,0,1,0,1,0,
Gene content,47,Opi,Orthogroups,2.5,1.00E-02,1,65812,1,1,1,1,1,1,0.2028809,1,1,0.1577625,0,1,0,1,0,11.09454228,11.09454228,-11.09454228,1,0,1,0,0,1,
Gene content,47,Opi,Orthogroups,2.5,1.00E-02,2,70211,1,1,1,1,1,1,0.1558138,1,1,0.1546364,0,1,0,1,0,11.15924603,11.15924603,-11.15924603,1,0,1,0,0,1,
Gene content,47,Opi,Orthogroups,2.5,1.00E-02,3,67909,1,1,1,1,1,1,0.2250452,1,1,0.1614309,0,1,0,1,0,11.12590913,11.12590913,-11.12590913,1,0,1,0,0,1,
Gene content,47,Opi,Orthogroups,2.5,1.00E-02,4,66951,1,1,1,1,1,1,0.1250448,1,1,0.1669056,0,1,0,1,0,11.11170135,11.11170135,-11.11170135,1,0,1,0,0,1,
Gene content,47,Opi,Orthogroups,2.5,1.00E-05,1,60001,1,1,1,1,1,1,0.006977623,1,1,0.9964223,0,1,0,1,0,11.00209984,11.00209984,-11.00209984,1,0,1,0,0,1,
Gene content,47,Opi,Orthogroups,2.5,1.00E-05,2,60001,1,1,1,1,1,1,0.003955468,1,1,0.9952223,0,1,0,1,0,11.00209984,11.00209984,-11.00209984,1,0,1,0,0,1,
Gene content,47,Opi,Orthogroups,2.5,1.00E-05,3,60001,1,1,1,1,1,1,0.0185107,1,1,0.9980667,0,1,0,1,0,11.00209984,11.00209984,-11.00209984,1,0,1,0,0,1,
Gene content,47,Opi,Orthogroups,2.5,1.00E-05,4,60001,1,1,1,1,1,1,0.01626631,1,1,0.9976223,0,1,0,1,0,11.00209984,11.00209984,-11.00209984,1,0,1,0,0,1,
Gene content,47,Opi,Orthogroups,2.5,1.00E-09,1,60001,1,1,1,1,1,1,0.9836004,1,1,0.9996445,0,1,0,1,0,11.00209984,11.00209984,-11.00209984,1,0,1,0,0,1,
Gene content,47,Opi,Orthogroups,2.5,1.00E-09,2,60001,1,1,1,1,1,1,0.9986889,1,1,0.9996445,0,1,0,1,0,11.00209984,11.00209984,-11.00209984,1,0,1,0,0,1,
Gene content,47,Opi,Orthogroups,2.5,1.00E-09,3,60001,1,1,1,1,1,1,0.9986889,1,1,0.9996445,0,1,0,1,0,11.00209984,11.00209984,-11.00209984,1,0,1,0,0,1,
Gene content,47,Opi,Orthogroups,2.5,1.00E-09,4,60001,1,1,1,1,1,1,0.9987334,1,1,0.9997778,0,1,0,1,0,11.00209984,11.00209984,-11.00209984,1,0,1,0,0,1,
Gene content,47,Opi,Orthogroups,2.5,1.00E-12,1,60001,1,1,1,1,1,1,1,1,1,0.9996,0,1,0,1,0,11.00209984,11.00209984,-11.00209984,1,0,1,0,0,1,
Gene content,47,Opi,Orthogroups,2.5,1.00E-12,2,60001,1,1,1,1,1,1,0.9996222,1,1,0.9999556,0,1,0,1,0,11.00209984,11.00209984,-11.00209984,1,0,1,0,0,1,
Gene content,47,Opi,Orthogroups,2.5,1.00E-12,3,60001,1,1,1,1,1,1,1,1,1,0.9999111,0,1,0,1,0,11.00209984,11.00209984,-11.00209984,1,0,1,0,0,1,
Gene content,47,Opi,Orthogroups,2.5,1.00E-12,4,60001,1,1,1,1,1,1,1,1,1,0.9999556,0,1,0,1,0,11.00209984,11.00209984,-11.00209984,1,0,1,0,0,1,
Gene content,47,Opi,Orthogroups,4.0,1.00E-02,1,70001,1,1,1,1,1,1,0.9334298,1,1,0.8708786,0,1,0,0,1,11.15625052,-11.15625052,-11.15625052,1,0,0,1,0,1,
Gene content,47,Opi,Orthogroups,4.0,1.00E-02,2,70001,1,1,1,1,1,1,0.9470867,1,1,0.8777928,0,1,0,0,1,11.15625052,-11.15625052,-11.15625052,1,0,0,1,0,1,
Gene content,47,Opi,Orthogroups,4.0,1.00E-02,3,70001,1,1,1,1,1,1,0.9283061,1,1,0.8720786,0,1,0,0,1,11.15625052,-11.15625052,-11.15625052,1,0,0,1,0,1,
Gene content,47,Opi,Orthogroups,4.0,1.00E-02,4,70001,1,1,1,1,1,1,0.9553532,1,1,0.8750119,0,1,0,0,1,11.15625052,-11.15625052,-11.15625052,1,0,0,1,0,1,
Gene content,47,Opi,Orthogroups,4.0,1.00E-05,1,60001,1,1,1,1,1,1,0.9981112,1,1,0.9832448,0,1,0,0,1,11.00209984,-11.00209984,-11.00209984,1,0,0,1,0,1,
Gene content,47,Opi,Orthogroups,4.0,1.00E-05,2,60001,1,1,1,1,1,1,0.9962223,1,1,0.9792005,0,1,0,0,1,11.00209984,-11.00209984,-11.00209984,1,0,0,1,0,1,
Gene content,47,Opi,Orthogroups,4.0,1.00E-05,3,60001,1,1,1,1,1,1,0.9981556,1,1,0.9790005,0,1,0,0,1,11.00209984,-11.00209984,-11.00209984,1,0,0,1,0,1,
Gene content,47,Opi,Orthogroups,4.0,1.00E-05,4,60001,1,1,1,1,1,1,0.9981556,1,1,0.9790005,0,1,0,0,1,11.00209984,-11.00209984,-11.00209984,1,0,0,1,0,1,
Gene content,47,Opi,Orthogroups,4.0,1.00E-09,1,60001,1,1,1,1,1,1,1,1,1,0.8694029,0,1,0,1,0,11.00209984,11.00209984,-11.00209984,1,0,1,0,0,1,
Gene content,47,Opi,Orthogroups,4.0,1.00E-09,2,60001,1,1,1,1,1,1,1,1,1,0.8795138,0,1,0,1,0,11.00209984,11.00209984,-11.00209984,1,0,1,0,0,1,
Gene content,47,Opi,Orthogroups,4.0,1.00E-09,3,60001,1,1,1,1,1,1,1,1,1,0.8836026,0,1,0,1,0,11.00209984,11.00209984,-11.00209984,1,0,1,0,0,1,
Gene content,47,Opi,Orthogroups,4.0,1.00E-09,4,60001,1,1,1,1,1,1,1,1,1,0.8836026,0,1,0,1,0,11.00209984,11.00209984,-11.00209984,1,0,1,0,0,1,
Gene content,47,Opi,Orthogroups,4.0,1.00E-12,1,58993,1,1,1,1,1,1,1,1,1,0.9986439,0,1,0,1,0,10.98515712,10.98515712,-10.98515712,1,0,1,0,0,1,
Gene content,47,Opi,Orthogroups,4.0,1.00E-12,2,48046,1,1,1,1,1,1,1,1,1,0.9983072,0,1,0,1,0,10.77989335,10.77989335,-10.77989335,1,0,1,0,0,1,
Gene content,47,Opi,Orthogroups,4.0,1.00E-12,3,59319,1,1,1,1,1,1,1,1,1,0.9988312,0,1,0,1,0,10.99066808,10.99066808,-10.99066808,1,0,1,0,0,1,
Gene content,47,Opi,Orthogroups,4.0,1.00E-12,4,54915,1,1,1,1,1,1,1,1,1,0.9983004,0,1,0,1,0,10.9135236,10.9135236,-10.9135236,1,0,1,0,0,1,
Gene content,47,Opi,Orthogroups,6.0,1.00E-02,1,70001,1,1,1,1,1,1,0.9993905,1,1,0.5114188,0,1,0,0,1,11.15625052,-11.15625052,-11.15625052,1,0,0,1,0,1,
Gene content,47,Opi,Orthogroups,6.0,1.00E-02,2,70001,1,1,1,1,1,1,0.9990476,1,1,0.4974,0,1,0,0,1,11.15625052,-11.15625052,-11.15625052,1,0,0,1,0,1,
Gene content,47,Opi,Orthogroups,6.0,1.00E-02,3,70001,1,1,1,1,1,1,0.9993143,1,1,0.5088284,0,1,0,0,1,11.15625052,-11.15625052,-11.15625052,1,0,0,1,0,1,
Gene content,47,Opi,Orthogroups,6.0,1.00E-02,4,70001,1,1,1,1,1,1,0.9993143,1,1,0.5088284,0,1,0,0,1,11.15625052,-11.15625052,-11.15625052,1,0,0,1,0,1,
Gene content,47,Opi,Orthogroups,6.0,1.00E-05,1,76060,1,1,1,1,1,1,1,1,1,0.1741082,0,1,0,0,1,11.23926463,-11.23926463,-11.23926463,1,0,0,1,0,1,
Gene content,47,Opi,Orthogroups,6.0,1.00E-05,2,71735,1,1,1,1,1,1,1,1,1,0.1721683,0,1,0,0,1,11.18072011,-11.18072011,-11.18072011,1,0,0,1,0,1,
Gene content,47,Opi,Orthogroups,6.0,1.00E-05,3,76445,1,1,1,1,1,1,1,1,1,0.1845502,0,1,0,0,1,11.24431373,-11.24431373,-11.24431373,1,0,0,1,0,1,
Gene content,47,Opi,Orthogroups,6.0,1.00E-05,4,70891,1,1,1,1,1,1,1,1,1,0.1790329,0,1,0,0,1,11.16888466,-11.16888466,-11.16888466,1,0,0,1,0,1,
Gene content,47,Opi,Orthogroups,6.0,1.00E-09,1,60001,1,1,1,1,1,1,1,1,1,0.3228817,0,1,0,0,1,11.00209984,-11.00209984,-11.00209984,1,0,0,1,0,1,
Gene content,47,Opi,Orthogroups,6.0,1.00E-09,2,60001,1,1,1,1,1,1,1,1,1,0.3195262,0,1,0,0,1,11.00209984,-11.00209984,-11.00209984,1,0,0,1,0,1,
Gene content,47,Opi,Orthogroups,6.0,1.00E-09,3,60001,1,1,1,1,1,1,1,1,1,0.3236373,0,1,0,0,1,11.00209984,-11.00209984,-11.00209984,1,0,0,1,0,1,
Gene content,47,Opi,Orthogroups,6.0,1.00E-09,4,60001,1,1,1,1,1,1,1,1,1,0.3174818,0,1,0,0,1,11.00209984,-11.00209984,-11.00209984,1,0,0,1,0,1,
Gene content,47,Opi,Orthogroups,6.0,1.00E-12,1,70001,1,1,1,1,1,1,1,1,1,0.9962858,0,1,0,1,0,11.15625052,11.15625052,-11.15625052,1,0,1,0,0,1,
Gene content,47,Opi,Orthogroups,6.0,1.00E-12,2,70001,1,1,1,1,1,1,1,1,1,0.997162,0,1,0,1,0,11.15625052,11.15625052,-11.15625052,1,0,1,0,0,1,
Gene content,47,Opi,Orthogroups,6.0,1.00E-12,3,70001,1,1,1,1,1,1,1,1,1,0.9916954,0,1,0,1,0,11.15625052,11.15625052,-11.15625052,1,0,1,0,0,1,
Gene content,47,Opi,Orthogroups,6.0,1.00E-12,4,70001,1,1,1,1,1,1,1,1,1,0.9966286,0,1,0,1,0,11.15625052,11.15625052,-11.15625052,1,0,1,0,0,1,
Gene content,36,ChoAco-dis,HomogroupsdisAb,1.5,1.00E-03,1,70001,1,1,1,1,1,1,0.0003238034,1,1,0.02087579,0,1,1,1,0,12.83589269,4.427140124,11.15625052,1,0,0,0,1,0,
Gene content,36,ChoAco-dis,HomogroupsdisAb,1.5,1.00E-03,2,70001,1,1,1,1,1,1,0.0001333308,1,1,0.02839946,0,1,1,1,0,12.83589269,4.427140124,11.15625052,1,0,0,0,1,0,
Gene content,36,ChoAco-dis,HomogroupsdisAb,1.5,1.00E-03,3,70001,1,1,1,1,1,1,0.0001142835,1,1,0.01908535,0,1,1,1,0,12.83589269,4.427140124,11.15625052,1,0,0,0,1,0,
Gene content,36,ChoAco-dis,HomogroupsdisAb,1.5,1.00E-03,4,70001,1,1,1,1,1,1,0.0002666616,1,1,0.02108531,0,1,1,1,0,12.83589269,4.427140124,11.15625052,1,0,0,0,1,0,
Gene content,36,ChoAco-dis,OrthogroupsdisAb,1.5,1.00E-03,1,40001,1,1,1,1,1,1,1,1,1,0.9838672,0,1,1,1,0,12.2762769,3.867524336,10.59663473,1,0,0,0,1,0,
Gene content,36,ChoAco-dis,OrthogroupsdisAb,1.5,1.00E-03,2,40001,1,1,1,1,1,1,0.9999667,1,1,0.9820339,0,1,1,1,0,12.2762769,3.867524336,10.59663473,1,0,0,0,1,0,
Gene content,36,ChoAco-dis,OrthogroupsdisAb,1.5,1.00E-03,3,40001,1,1,1,1,1,1,0.9999667,1,1,0.9820339,0,1,1,1,0,12.2762769,3.867524336,10.59663473,1,0,0,0,1,0,
Gene content,36,ChoAco-dis,OrthogroupsdisAb,1.5,1.00E-03,4,40001,1,1,1,1,1,1,0.9895003,1,1,0.9866338,0,1,1,1,0,12.2762769,3.867524336,10.59663473,1,0,0,0,1,0,
Gene content,36,ChoAco-dis,OrthogroupsdisP,1.5,1.00E-03,1,40001,1,1,1,1,1,1,0.9751675,1,1,0.9744342,0,1,1,1,0,12.2762769,3.867524336,10.59663473,1,0,0,0,1,0,
Gene content,36,ChoAco-dis,OrthogroupsdisP,1.5,1.00E-03,2,40001,1,1,1,1,1,1,0.9710343,1,1,0.978634,0,1,1,1,0,12.2762769,3.867524336,10.59663473,1,0,0,0,1,0,

Gene content,36,ChoAco-dis,OrthogroupsdisP,1.5,1.00E-03,3,40001,1,1,1,1,1,1,0.9963001,1,1,0.9735009,0,1,1,1,0,12.2762769,3.867524336,10.59663473,1,0,0,0,1,0,
Gene content,36,ChoAco-dis,OrthogroupsdisP,1.5,1.00E-03,4,40001,1,1,1,1,1,1,0.9963001,1,1,0.9735009,0,1,1,1,0,12.2762769,3.867524336,10.59663473,1,0,0,0,1,0,
Gene content,33,ChoAco-ne,HomogroupsneAb,1.5,1.00E-03,1,40001,1,1,1,1,1,1,1,1,1,0.01526616,0,1,1,1,0,13.06473426,5.479184471,10.59663473,1,0,1,0,1,0,
Gene content,33,ChoAco-ne,HomogroupsneAb,1.5,1.00E-03,2,40001,1,1,1,1,1,1,1,1,1,0.01326622,0,1,1,1,0,13.06473426,5.479184471,10.59663473,1,0,1,0,1,0,
Gene content,33,ChoAco-ne,HomogroupsneAb,1.5,1.00E-03,3,40001,1,1,1,1,1,1,1,1,1,0.009033032,0,1,1,1,0,13.06473426,5.479184471,10.59663473,1,0,1,0,1,0,
Gene content,33,ChoAco-ne,HomogroupsneAb,1.5,1.00E-03,4,40001,1,1,1,1,1,1,1,1,1,0.009033032,0,1,1,1,0,13.06473426,5.479184471,10.59663473,1,0,1,0,1,0,
Gene content,33,ChoAco-ne,HomogroupsneP,1.5,1.00E-03,1,60001,1,1,1,1,1,1,1,1,1,0.9545343,0,1,1,1,0,13.47019937,5.884649579,11.00209984,1,0,1,0,1,0,
Gene content,33,ChoAco-ne,HomogroupsneP,1.5,1.00E-03,2,60001,1,1,1,1,1,1,1,1,1,0.9650008,0,1,1,1,0,13.47019937,5.884649579,11.00209984,1,0,1,0,1,0,
Gene content,33,ChoAco-ne,HomogroupsneP,1.5,1.00E-03,3,60001,1,1,1,1,1,1,1,1,1,0.960712,0,1,1,1,0,13.47019937,5.884649579,11.00209984,1,0,1,0,1,0,
Gene content,33,ChoAco-ne,HomogroupsneP,1.5,1.00E-03,4,60001,1,1,1,1,1,1,1,1,1,0.9679118,0,1,1,1,0,13.47019937,5.884649579,11.00209984,1,0,1,0,1,0,
Gene content,33,ChoAco-ne,OrthogroupsneAb,1.5,1.00E-03,1,50001,1,1,1,1,1,1,1,1,1,0.9603211,0,1,1,1,0,13.28787782,5.702328022,10.81977828,1,0,1,0,1,0,
Gene content,33,ChoAco-ne,OrthogroupsneAb,1.5,1.00E-03,2,50001,1,1,1,1,1,1,1,1,1,0.9635743,0,1,1,1,0,13.28787782,5.702328022,10.81977828,1,0,1,0,1,0,
Gene content,33,ChoAco-ne,OrthogroupsneAb,1.5,1.00E-03,3,50001,1,1,1,1,1,1,1,1,1,0.9572011,0,1,1,1,0,13.28787782,5.702328022,10.81977828,1,0,1,0,1,0,
Gene content,33,ChoAco-ne,OrthogroupsneAb,1.5,1.00E-03,4,50001,1,1,1,1,1,1,1,1,1,0.9572011,0,1,1,1,0,13.28787782,5.702328022,10.81977828,1,0,1,0,1,0,
Gene content,33,ChoAco-ne,OrthogroupsneP,1.5,1.00E-03,1,60001,1,1,1,1,1,1,1,1,1,0.9850448,0,1,1,1,0,13.47019937,5.884649579,11.00209984,1,0,1,0,1,0,
Gene content,33,ChoAco-ne,OrthogroupsneP,1.5,1.00E-03,2,60001,1,1,1,1,1,1,1,1,1,0.9820671,0,1,1,1,0,13.47019937,5.884649579,11.00209984,1,0,1,0,1,0,
Gene content,33,ChoAco-ne,OrthogroupsneP,1.5,1.00E-03,3,60001,1,1,1,1,1,1,1,1,1,0.9814226,0,1,1,1,0,13.47019937,5.884649579,11.00209984,1,0,1,0,1,0,
Gene content,33,ChoAco-ne,OrthogroupsneP,1.5,1.00E-03,4,60001,1,1,1,1,1,1,1,1,1,0.9858003,0,1,1,1,0,13.47019937,5.884649579,11.00209984,1,0,1,0,1,0,
Gene content,39,Cho-dis,HomogroupsdisAb,1.5,1.00E-03,1,50001,0.9998133,1,1,1,1,1,0,1,1,0.003199915,0,1,1,1,0,7.035818245,15.20476359,10.81977828,1,0,1,0,1,0,
Gene content,39,Cho-dis,HomogroupsdisAb,1.5,1.00E-03,2,50001,0.9998667,1,1,1,1,1,0,1,1,0.002479934,0,1,1,1,0,7.035818245,15.20476359,10.81977828,1,0,1,0,1,0,
Gene content,39,Cho-dis,HomogroupsdisAb,1.5,1.00E-03,3,50001,1,1,1,1,1,1,0,1,1,0.002533266,0,1,1,1,0,7.035818245,15.20476359,10.81977828,1,0,1,0,1,0,
Gene content,39,Cho-dis,HomogroupsdisAb,1.5,1.00E-03,4,50001,0.9999733,1,1,1,1,1,0,1,1,0.002266606,0,1,1,1,0,7.035818245,15.20476359,10.81977828,1,0,1,0,1,0,
Gene content,39,Cho-dis,HomogroupsdisP,1.5,1.00E-03,1,60001,0.9999556,1,1,1,1,1,0,1,1,0.003333259,0,1,1,1,0,7.218139802,15.38708514,11.00209984,1,0,1,0,1,0,
Gene content,39,Cho-dis,HomogroupsdisP,1.5,1.00E-03,2,60001,0.9998222,1,1,1,1,1,0,1,1,0.00539988,0,1,1,1,0,7.218139802,15.38708514,11.00209984,1,0,1,0,1,0,
Gene content,39,Cho-dis,HomogroupsdisP,1.5,1.00E-03,3,60001,0.9998222,1,1,1,1,1,0,1,1,0.00539988,0,1,1,1,0,7.218139802,15.38708514,11.00209984,1,0,1,0,1,0,
Gene content,39,Cho-dis,HomogroupsdisP,1.5,1.00E-03,4,60001,0.9999333,1,1,1,1,1,0,1,1,0.00282216,0,1,1,1,0,7.218139802,15.38708514,11.00209984,1,0,1,0,1,0,
Gene content,39,Cho-dis,OrthogroupsdisAb,1.5,1.00E-03,1,100001,1,1,1,1,1,0,0.1608112,1,1,0.9910801,0,1,0,0,1,7.728965426,-7.127940163,-11.51292546,1,0,0,1,0,1,
Gene content,39,Cho-dis,OrthogroupsdisAb,1.5,1.00E-03,2,100001,1,1,1,1,1,0,0.2999693,1,1,0.9919334,0,1,0,0,1,7.728965426,-7.127940163,-11.51292546,1,0,0,1,0,1,
Gene content,39,Cho-dis,OrthogroupsdisAb,1.5,1.00E-03,3,100001,1,1,1,1,1,0,0.1615712,1,1,0.9926001,0,1,0,0,1,7.728965426,-7.127940163,-11.51292546,1,0,0,1,0,1,
Gene content,39,Cho-dis,OrthogroupsdisAb,1.5,1.00E-03,4,100001,1,1,1,1,1,0,0.1615712,1,1,0.9926001,0,1,0,0,1,7.728965426,-7.127940163,-11.51292546,1,0,0,1,0,1,
Gene content,39,Cho-dis,OrthogroupsdisP,1.5,1.00E-03,1,33369,0.9999201,1,1,1,1,0,0.4949055,1,1,0.9977225,0,1,0,0,1,6.631392597,-6.030367334,-10.41535264,1,0,0,1,0,1,
Gene content,39,Cho-dis,OrthogroupsdisP,1.5,1.00E-03,2,28092,1,1,1,1,1,0,0.3190944,1,1,0.9983388,0,1,0,0,1,6.45924448,-5.858219218,-10.24320452,1,0,0,1,0,1,
Gene content,39,Cho-dis,OrthogroupsdisP,1.5,1.00E-03,3,30189,1,1,1,1,1,0,0.282528,1,1,0.999205,0,1,0,0,1,6.531239734,-5.930214472,-10.31519977,1,0,0,1,0,1,
Gene content,39,Cho-dis,OrthogroupsdisP,1.5,1.00E-03,4,30574,1,1,1,1,1,0,0.3255418,1,1,0.9973398,0,1,0,0,1,6.543912506,-5.942887244,-10.32787255,1,0,0,1,0,1,
Gene content,36,Cho-ne,HomogroupsneAb,1.5,1.00E-03,1,40001,0.9992,1,1,1,1,1,1,1,1,0.0948635,0,1,1,1,0,4.925605045,13.55801213,10.59663473,0,0,1,0,1,0,
Gene content,36,Cho-ne,HomogroupsneAb,1.5,1.00E-03,2,40001,0.9994,1,1,1,1,1,1,1,1,0.08983034,0,1,1,1,0,4.925605045,13.55801213,10.59663473,0,0,1,0,1,0,
Gene content,36,Cho-ne,HomogroupsneAb,1.5,1.00E-03,3,40001,0.9992667,1,1,1,1,1,1,1,1,0.0978634,0,1,1,1,0,4.925605045,13.55801213,10.59663473,0,0,1,0,1,0,
Gene content,36,Cho-ne,HomogroupsneAb,1.5,1.00E-03,4,40001,0.9996667,1,1,1,1,1,1,1,1,0.09929669,0,1,1,1,0,4.925605045,13.55801213,10.59663473,0,0,1,0,1,0,
Gene content,36,Cho-ne,HomogroupsneP,1.5,1.00E-03,1,60001,0.9999556,1,1,1,1,1,1,1,0,0,0,0,1,0,0,-5.671029688,2.961377397,11.00209984,0,1,0,0,1,0,
Gene content,36,Cho-ne,HomogroupsneP,1.5,1.00E-03,2,50001,1,1,1,1,1,1,1,1,1,0.0612517,0,1,1,1,0,5.148748596,13.78115568,10.81977828,1,0,1,0,1,0,
Gene content,36,Cho-ne,HomogroupsneP,1.5,1.00E-03,3,50001,1,1,1,1,1,1,1,1,1,0.06562492,0,1,1,1,0,5.148748596,13.78115568,10.81977828,1,0,1,0,1,0,
Gene content,36,Cho-ne,HomogroupsneP,1.5,1.00E-03,4,50001,1,1,1,1,1,1,1,1,1,0.06045172,0,1,1,1,0,5.148748596,13.78115568,10.81977828,1,0,1,0,1,0,
Gene content,36,Cho-ne,OrthogroupsneAb,1.5,1.00E-03,1,80001,0.9999833,1,1,1,1,0,1,1,1,0.9993333,0,1,0,0,1,5.618752225,-8.328404516,-11.28978191,1,0,0,1,0,1,
Gene content,36,Cho-ne,OrthogroupsneAb,1.5,1.00E-03,2,80001,1,1,1,1,1,0,1,1,1,0.9992333,0,1,0,0,1,5.618752225,-8.328404516,-11.28978191,1,0,0,1,0,1,
Gene content,36,Cho-ne,OrthogroupsneAb,1.5,1.00E-03,3,80001,1,1,1,1,1,0,1,1,1,0.99915,0,1,0,0,1,5.618752225,-8.328404516,-11.28978191,1,0,0,1,0,1,
Gene content,36,Cho-ne,OrthogroupsneAb,1.5,1.00E-03,4,80001,1,1,1,1,1,0,1,1,1,0.9996167,0,1,0,0,1,5.618752225,-8.328404516,-11.28978191,1,0,0,1,0,1,
Gene content,36,Cho-ne,OrthogroupsneP,1.5,1.00E-03,1,80001,1,1,1,1,1,0,1,1,1,0.9979334,0,1,0,0,1,5.618752225,-8.328404516,-11.28978191,1,0,0,1,0,1,
Gene content,36,Cho-ne,OrthogroupsneP,1.5,1.00E-03,2,80001,1,1,1,1,1,0,1,1,1,0.9985834,0,1,0,0,1,5.618752225,-8.328404516,-11.28978191,1,0,0,1,0,1,
Gene content,36,Cho-ne,OrthogroupsneP,1.5,1.00E-03,3,80001,1,1,1,1,1,0,1,1,1,0.9994333,0,1,0,0,1,5.618752225,-8.328404516,-11.28978191,1,0,0,1,0,1,
Gene content,36,Cho-ne,OrthogroupsneP,1.5,1.00E-03,4,80001,1,1,1,1,1,0,1,1,1,0.99805,0,1,0,0,1,5.618752225,-8.328404516,-11.28978191,1,0,0,1,0,1,
Gene content,38,HolAco-dis,HomogroupsdisAb,1.5,1.00E-03,1,40001,1,1,1,1,1,1,0.005966468,1,1,0.00119996,0,1,1,1,0,11.96612198,18.28006787,10.59663473,1,0,1,0,1,0,
Gene content,38,HolAco-dis,HomogroupsdisAb,1.5,1.00E-03,2,40001,1,1,1,1,1,1,0.01119963,1,1,0.0008666378,0,1,1,1,0,11.96612198,18.28006787,10.59663473,1,0,1,0,1,0,
Gene content,38,HolAco-dis,HomogroupsdisAb,1.5,1.00E-03,3,40001,1,1,1,1,1,1,0.007866404,1,1,0.001033299,0,1,1,1,0,11.96612198,18.28006787,10.59663473,1,0,1,0,1,0,
Gene content,38,HolAco-dis,HomogroupsdisAb,1.5,1.00E-03,4,40001,1,1,1,1,1,1,0.002066598,1,1,0.0006999767,0,1,1,1,0,11.96612198,18.28006787,10.59663473,1,0,1,0,1,0,
Gene content,38,HolAco-dis,HomogroupsdisP,1.5,1.00E-03,1,50001,1,1,1,1,1,1,0.01050639,1,1,0.03101251,0,1,1,1,0,12.18926553,18.50321142,10.81977828,1,0,1,0,1,0,
Gene content,38,HolAco-dis,HomogroupsdisP,1.5,1.00E-03,2,50001,1,1,1,1,1,1,0.01554625,1,1,0.0274926,0,1,1,1,0,12.18926553,18.50321142,10.81977828,1,0,1,0,1,0,
Gene content,38,HolAco-dis,HomogroupsdisP,1.5,1.00E-03,3,50001,1,1,1,1,1,1,0.01594624,1,1,0.02493267,0,1,1,1,0,12.18926553,18.50321142,10.81977828,1,0,1,0,1,0,
Gene content,38,HolAco-dis,HomogroupsdisP,1.5,1.00E-03,4,50001,1,1,1,1,1,1,0.01213301,1,1,0.02626597,0,1,1,1,0,12.18926553,18.50321142,10.81977828,1,0,1,0,1,0,
Gene content,38,HolAco-dis,OrthogroupsdisAb,1.5,1.00E-03,1,40001,1,1,1,1,1,1,0.9995667,1,1,0.9985334,0,1,1,1,0,11.96612198,18.28006787,10.59663473,1,0,1,0,1,0,
Gene content,38,HolAco-dis,OrthogroupsdisAb,1.5,1.00E-03,2,40001,1,1,1,1,1,1,0.9929336,1,1,0.9975001,0,1,1,1,0,11.96612198,18.28006787,10.59663473,1,0,1,0,1,0,
Gene content,38,HolAco-dis,OrthogroupsdisAb,1.5,1.00E-03,3,40001,1,1,1,1,1,1,0.9929336,1,1,0.9975001,0,1,1,1,0,11.96612198,18.28006787,10.59663473,1,0,1,0,1,0,
Gene content,38,HolAco-dis,OrthogroupsdisAb,1.5,1.00E-03,4,40001,1,1,1,1,1,1,0.9738675,1,1,0.9996,0,1,1,1,0,11.96612198,18.28006787,10.59663473,1,0,1,0,1,0,
Gene content,38,HolAco-dis,OrthogroupsdisP,1.5,1.00E-03,1,40001,1,1,1,1,1,1,0.9988334,1,1,0.9932336,0,1,1,1,0,11.96612198,18.28006787,10.59663473,1,0,1,0,1,0,
Gene content,38,HolAco-dis,OrthogroupsdisP,1.5,1.00E-03,2,40001,1,1,1,1,1,1,0.9925669,1,1,0.9936335,0,1,1,1,0,11.96612198,18.28006787,10.59663473,1,0,1,0,1,0,
Gene content,38,HolAco-dis,OrthogroupsdisP,1.5,1.00E-03,3,40001,1,1,1,1,1,1,0.9925669,1,1,0.9936335,0,1,1,1,0,11.96612198,18.28006787,10.59663473,1,0,1,0,1,0,
Gene content,38,HolAco-dis,OrthogroupsdisP,1.5,1.00E-03,4,40001,1,1,1,1,1,1,0.9992334,1,1,0.9915003,0,1,1,1,0,11.96612198,18.28006787,10.59663473,1,0,1,0,1,0,
Gene content,35,HolAco-ne,HomogroupsneAb,1.5,1.00E-03,1,40001,1,1,1,1,1,1,1,1,1,0.00119996,0,1,1,1,0,7.892009193,16.81491951,10.59663473,1,0,1,0,1,0,
Gene content,35,HolAco-ne,HomogroupsneAb,1.5,1.00E-03,2,40001,1,1,1,1,1,1,1,1,1,0.00239992,0,1,1,1,0,7.892009193,16.81491951,10.59663473,1,0,1,0,1,0,
Gene content,35,HolAco-ne,HomogroupsneAb,1.5,1.00E-03,3,40001,1,1,1,1,1,1,1,1,1,0.001866604,0,1,1,1,0,7.892009193,16.81491951,10.59663473,1,0,1,0,1,0,
Gene content,35,HolAco-ne,HomogroupsneAb,1.5,1.00E-03,4,40001,1,1,1,1,1,1,1,1,1,0.001866604,0,1,1,1,0,7.892009193,16.81491951,10.59663473,1,0,1,0,1,0,
Gene content,35,HolAco-ne,HomogroupsneP,1.5,1.00E-03,1,100001,1,1,1,1,1,1,1,1,1,0.001826642,0,1,1,1,0,8.808299925,17.73121024,11.51292546,1,0,1,0,1,0,
Gene content,35,HolAco-ne,HomogroupsneP,1.5,1.00E-03,2,100001,1,1,1,1,1,1,1,1,1,0.001119985,0,1,1,1,0,8.808299925,17.73121024,11.51292546,1,0,1,0,1,0,
Gene content,35,HolAco-ne,HomogroupsneP,1.5,1.00E-03,3,100001,1,1,1,1,1,1,1,1,1,0.001039986,0,1,1,1,0,8.808299925,17.73121024,11.51292546,1,0,1,0,1,0,
Gene content,35,HolAco-ne,HomogroupsneP,1.5,1.00E-03,4,100001,1,1,1,1,1,1,1,1,1,0.001626645,0,1,1,1,0,8.808299925,17.73121024,11.51292546,1,0,1,0,1,0,
Gene content,35,HolAco-ne,OrthogroupsneAb,1.5,1.00E-03,1,60001,1,1,1,1,1,1,1,1,1,0.9936224,0,1,1,1,0,8.297474301,17.22038461,11.00209984,1,0,1,0,1,0,
Gene content,35,HolAco-ne,OrthogroupsneAb,1.5,1.00E-03,2,60001,1,1,1,1,1,1,1,1,1,0.9941557,0,1,1,1,0,8.297474301,17.22038461,11.00209984,1,0,1,0,1,0,
Gene content,35,HolAco-ne,OrthogroupsneAb,1.5,1.00E-03,3,60001,1,1,1,1,1,1,1,1,1,0.9922891,0,1,1,1,0,8.297474301,17.22038461,11.00209984,1,0,1,0,1,0,
Gene content,35,HolAco-ne,OrthogroupsneAb,1.5,1.00E-03,4,60001,1,1,1,1,1,1,1,1,1,0.993489,0,1,1,1,0,8.297474301,17.22038461,11.00209984,1,0,1,0,1,0,
Gene content,35,HolAco-ne,OrthogroupsneP,1.5,1.00E-03,1,40001,1,1,1,1,1,1,1,1,1,0.9973001,0,1,1,1,0,7.892009193,16.81491951,10.59663473,1,0,1,0,1,0,
Gene content,35,HolAco-ne,OrthogroupsneP,1.5,1.00E-03,2,40001,1,1,1,1,1,1,1,1,1,0.9980001,0,1,1,1,0,7.892009193,16.81491951,10.59663473,1,0,1,0,1,0,
Gene content,35,HolAco-ne,OrthogroupsneP,1.5,1.00E-03,3,40001,1,1,1,1,1,1,1,1,1,0.9980001,0,1,1,1,0,7.892009193,16.81491951,10.59663473,1,0,1,0,1,0,
Gene content,35,HolAco-ne,OrthogroupsneP,1.5,1.00E-03,4,40001,1,1,1,1,1,1,1,1,1,0.9977334,0,1,1,1,0,7.892009193,16.81491951,10.59663473,1,0,1,0,1,0,

Gene content,41,Hol-dis,HomogroupsdisAb,1.5,1.00E-03,1,40001,1,1,1,1,1,1,0,1,0,0,0,0,0,0,0,-3.106080331,5.231094455,-10.59663473,0,0,1,0,0,1,
Gene content,41,Hol-dis,HomogroupsdisAb,1.5,1.00E-03,2,40001,1,1,1,1,1,1,0,1,0,0,0,0,0,0,0,-3.106080331,5.231094455,-10.59663473,0,0,1,0,0,1,
Gene content,41,Hol-dis,HomogroupsdisAb,1.5,1.00E-03,3,40001,1,1,1,1,1,1,0.0007333089,1,0,0,0,0,0,0,0,-3.106080331,5.231094455,-10.59663473,0,0,1,0,0,1,
Gene content,41,Hol-dis,HomogroupsdisAb,1.5,1.00E-03,4,40001,1,1,1,1,1,1,0,1,0,0,0,0,0,0,0,-3.106080331,5.231094455,-10.59663473,0,0,1,0,0,1,
Gene content,41,Hol-dis,HomogroupsdisP,1.5,1.00E-03,1,50001,1,1,1,1,1,1,0,1,0,0,0,0,0,0,0,-3.106080331,5.231094455,-10.81977828,0,0,1,0,0,1,
Gene content,41,Hol-dis,HomogroupsdisP,1.5,1.00E-03,2,50001,1,1,1,1,1,1,0,1,0,0,0,0,0,0,0,-3.106080331,5.231094455,-10.81977828,0,0,1,0,0,1,
Gene content,41,Hol-dis,HomogroupsdisP,1.5,1.00E-03,3,50001,1,1,1,1,1,1,0.0001866617,1,0,0,0,0,0,0,0,-3.106080331,5.231094455,-10.81977828,0,0,1,0,0,1,
Gene content,41,Hol-dis,HomogroupsdisP,1.5,1.00E-03,4,50001,1,1,1,1,1,1,0,1,0,0,0,0,0,0,0,-3.106080331,5.231094455,-10.81977828,0,0,1,0,0,1,
Gene content,41,Hol-dis,OrthogroupsdisAb,1.5,1.00E-03,1,100001,1,1,1,1,1,1,0.9777603,1,0,0,0,0,0,0,0,-3.106080331,5.231094455,-11.51292546,0,0,1,0,0,1,
Gene content,41,Hol-dis,OrthogroupsdisAb,1.5,1.00E-03,2,100001,1,1,1,1,1,1,0.9868268,1,0,0,0,0,0,0,0,-3.106080331,5.231094455,-11.51292546,0,0,1,0,0,1,
Gene content,41,Hol-dis,OrthogroupsdisAb,1.5,1.00E-03,3,100001,1,1,1,1,1,1,0.99968,1,0,0,0,0,0,0,0,-3.106080331,5.231094455,-11.51292546,0,0,1,0,0,1,
Gene content,41,Hol-dis,OrthogroupsdisAb,1.5,1.00E-03,4,100001,1,1,1,1,1,1,0.9723737,1,0,0,0,0,0,0,0,-3.106080331,5.231094455,-11.51292546,0,0,1,0,0,1,
Gene content,41,Hol-dis,OrthogroupsdisP,1.5,1.00E-03,1,80001,1,1,1,1,1,0,0.9058016,1,1,0.9993833,0,1,0,0,1,8.183701583,-6.058687459,-11.28978191,1,0,0,1,0,1,
Gene content,41,Hol-dis,OrthogroupsdisP,1.5,1.00E-03,2,80001,1,1,1,1,1,0,0.9249846,1,1,0.9996667,0,1,0,0,1,8.183701583,-6.058687459,-11.28978191,1,0,0,1,0,1,
Gene content,41,Hol-dis,OrthogroupsdisP,1.5,1.00E-03,3,80001,1,1,1,1,1,0,0.9249846,1,1,0.9996667,0,1,0,0,1,8.183701583,-6.058687459,-11.28978191,1,0,0,1,0,1,
Gene content,41,Hol-dis,OrthogroupsdisP,1.5,1.00E-03,4,80001,1,1,1,1,1,0,0.9440176,1,1,0.9997333,0,1,0,0,1,8.183701583,-6.058687459,-11.28978191,1,0,0,1,0,1,
Gene content,38,Hol-ne,HomogroupsneAb,1.5,1.00E-03,1,40001,1,1,1,1,1,1,1,1,1,0.07876404,0,1,1,1,0,7.681222984,14.52963491,10.59663473,1,0,1,0,1,0,
Gene content,38,Hol-ne,HomogroupsneAb,1.5,1.00E-03,2,40001,1,1,1,1,1,1,1,1,1,0.07099763,0,1,1,1,0,7.681222984,14.52963491,10.59663473,1,0,1,0,1,0,
Gene content,38,Hol-ne,HomogroupsneAb,1.5,1.00E-03,3,40001,1,1,1,1,1,1,1,1,1,0.07099763,0,1,1,1,0,7.681222984,14.52963491,10.59663473,1,0,1,0,1,0,
Gene content,38,Hol-ne,HomogroupsneAb,1.5,1.00E-03,4,40001,1,1,1,1,1,1,1,1,1,0.07793074,0,1,1,1,0,7.681222984,14.52963491,10.59663473,1,0,1,0,1,0,
Gene content,38,Hol-ne,HomogroupsneP,1.5,1.00E-03,1,50001,1,1,1,1,1,1,1,1,1,0.06322498,0,1,1,1,0,7.904366535,14.75277846,10.81977828,1,0,1,0,1,0,
Gene content,38,Hol-ne,HomogroupsneP,1.5,1.00E-03,2,50001,1,1,1,1,1,1,1,1,1,0.06349164,0,1,1,1,0,7.904366535,14.75277846,10.81977828,1,0,1,0,1,0,
Gene content,38,Hol-ne,HomogroupsneP,1.5,1.00E-03,3,50001,1,1,1,1,1,1,1,1,1,0.05877177,0,1,1,1,0,7.904366535,14.75277846,10.81977828,1,0,1,0,1,0,
Gene content,38,Hol-ne,HomogroupsneP,1.5,1.00E-03,4,50001,1,1,1,1,1,1,1,1,1,0.05893176,0,1,1,1,0,7.904366535,14.75277846,10.81977828,1,0,1,0,1,0,
Gene content,38,Hol-ne,OrthogroupsneAb,1.5,1.00E-03,1,100001,1,1,1,1,1,0,1,1,1,0.9998,0,1,0,0,1,8.597513715,-7.579925287,-11.51292546,1,0,0,1,0,1,
Gene content,38,Hol-ne,OrthogroupsneAb,1.5,1.00E-03,2,100001,1,1,1,1,1,0,1,1,1,0.99968,0,1,0,0,1,8.597513715,-7.579925287,-11.51292546,1,0,0,1,0,1,
Gene content,38,Hol-ne,OrthogroupsneAb,1.5,1.00E-03,3,100001,1,1,1,1,1,0,1,1,1,0.99968,0,1,0,0,1,8.597513715,-7.579925287,-11.51292546,1,0,0,1,0,1,
Gene content,38,Hol-ne,OrthogroupsneAb,1.5,1.00E-03,4,100001,1,1,1,1,1,0,1,1,1,0.99984,0,1,0,0,1,8.597513715,-7.579925287,-11.51292546,1,0,0,1,0,1,
Gene content,38,Hol-ne,OrthogroupsneP,1.5,1.00E-03,1,80001,1,1,1,1,1,0,1,1,1,0.99975,0,1,0,0,1,8.374370164,-7.356781736,-11.28978191,1,0,0,1,0,1,
Gene content,38,Hol-ne,OrthogroupsneP,1.5,1.00E-03,2,80001,1,1,1,1,1,0,1,1,1,0.9997667,0,1,0,0,1,8.374370164,-7.356781736,-11.28978191,1,0,0,1,0,1,
Gene content,38,Hol-ne,OrthogroupsneP,1.5,1.00E-03,3,80001,1,1,1,1,1,0,1,1,1,0.9995333,0,1,0,0,1,8.374370164,-7.356781736,-11.28978191,1,0,0,1,0,1,
Gene content,38,Hol-ne,OrthogroupsneP,1.5,1.00E-03,4,80001,1,1,1,1,1,0,1,1,1,0.9997833,0,1,0,0,1,8.374370164,-7.356781736,-11.28978191,1,0,0,1,0,1,
Gene content,35,HolXen-dis,HomogroupsdisAb,1.5,1.00E-03,1,40001,1,1,1,1,1,1,1,0.3074231,1,1,0.04276524,0,1,1,1,0,8.550641125,21.7827722,10.59663473,1,0,1,0,1,0,
Gene content,35,HolXen-dis,HomogroupsdisAb,1.5,1.00E-03,2,40001,1,1,1,1,1,1,1,0.3944535,1,1,0.04236525,0,1,1,1,0,8.550641125,21.7827722,10.59663473,1,0,1,0,1,0,
Gene content,35,HolXen-dis,HomogroupsdisAb,1.5,1.00E-03,3,40001,1,1,1,1,1,1,1,0.329489,1,1,0.04326522,0,1,1,1,0,8.550641125,21.7827722,10.59663473,1,0,1,0,1,0,
Gene content,35,HolXen-dis,HomogroupsdisAb,1.5,1.00E-03,4,40001,1,1,1,1,1,1,1,0.2898237,1,1,0.04419853,0,1,1,1,0,8.550641125,21.7827722,10.59663473,1,0,1,0,1,0,
Gene content,35,HolXen-dis,HomogroupsdisP,1.5,1.00E-03,1,70001,1,1,1,1,1,1,1,0.01558066,1,1,0.07011295,0,1,1,1,0,9.110256913,22.34238798,11.15625052,1,0,1,0,1,0,
Gene content,35,HolXen-dis,HomogroupsdisP,1.5,1.00E-03,2,70001,1,1,1,1,1,1,1,0.01034266,1,1,0.07350336,0,1,1,1,0,9.110256913,22.34238798,11.15625052,1,0,1,0,1,0,
Gene content,35,HolXen-dis,HomogroupsdisP,1.5,1.00E-03,3,70001,1,1,1,1,1,1,1,0.02664711,1,1,0.07266528,0,1,1,1,0,9.110256913,22.34238798,11.15625052,1,0,1,0,1,0,
Gene content,35,HolXen-dis,HomogroupsdisP,1.5,1.00E-03,4,70001,1,1,1,1,1,1,1,0.02664711,1,1,0.07266528,0,1,1,1,0,9.110256913,22.34238798,11.15625052,1,0,1,0,1,0,
Gene content,35,HolXen-dis,OrthogroupsdisAb,1.5,1.00E-03,1,40001,1,1,1,1,1,1,1,0.9638012,1,1,0.9549348,0,1,1,1,0,8.550641125,21.7827722,10.59663473,1,0,1,0,1,0,
Gene content,35,HolXen-dis,OrthogroupsdisAb,1.5,1.00E-03,2,40001,1,1,1,1,1,1,1,0.9018366,1,1,0.9474351,0,1,1,1,0,8.550641125,21.7827722,10.59663473,1,0,1,0,1,0,
Gene content,35,HolXen-dis,OrthogroupsdisAb,1.5,1.00E-03,3,40001,1,1,1,1,1,1,1,0.9018366,1,1,0.9474351,0,1,1,1,0,8.550641125,21.7827722,10.59663473,1,0,1,0,1,0,
Gene content,35,HolXen-dis,OrthogroupsdisAb,1.5,1.00E-03,4,40001,1,1,1,1,1,1,1,0.9018366,1,1,0.9474351,0,1,1,1,0,8.550641125,21.7827722,10.59663473,1,0,1,0,1,0,
Gene content,35,HolXen-dis,OrthogroupsdisP,1.5,1.00E-03,1,40001,1,1,1,1,1,1,1,0.9904337,1,1,0.9987334,0,1,1,1,0,8.550641125,21.7827722,10.59663473,1,0,1,0,1,0,
Gene content,35,HolXen-dis,OrthogroupsdisP,1.5,1.00E-03,2,40001,1,1,1,1,1,1,1,0.9971668,1,1,0.9974668,0,1,1,1,0,8.550641125,21.7827722,10.59663473,1,0,1,0,1,0,
Gene content,35,HolXen-dis,OrthogroupsdisP,1.5,1.00E-03,3,40001,1,1,1,1,1,1,1,0.9943335,1,1,0.9976667,0,1,1,1,0,8.550641125,21.7827722,10.59663473,1,0,1,0,1,0,
Gene content,35,HolXen-dis,OrthogroupsdisP,1.5,1.00E-03,4,40001,1,1,1,1,1,1,1,0.9856338,1,1,0.9963335,0,1,1,1,0,8.550641125,21.7827722,10.59663473,1,0,1,0,1,0,
Gene content,32,HolXen-ne,HomogroupsneAb,1.5,1.00E-03,1,40001,1,1,1,1,1,1,1,1,1,1,0.1278957,0,1,1,1,0,8.806424451,19.7519702,10.59663473,1,0,1,0,1,0,
Gene content,32,HolXen-ne,HomogroupsneAb,1.5,1.00E-03,2,40001,1,1,1,1,1,1,1,1,1,1,0.1277624,0,1,1,1,0,8.806424451,19.7519702,10.59663473,1,0,1,0,1,0,
Gene content,32,HolXen-ne,HomogroupsneAb,1.5,1.00E-03,3,40001,1,1,1,1,1,1,1,1,1,1,0.1259625,0,1,1,1,0,8.806424451,19.7519702,10.59663473,1,0,1,0,1,0,
Gene content,32,HolXen-ne,HomogroupsneAb,1.5,1.00E-03,4,40001,1,1,1,1,1,1,1,1,1,1,0.1249958,0,1,1,1,0,8.806424451,19.7519702,10.59663473,1,0,1,0,1,0,
Gene content,32,HolXen-ne,HomogroupsneP,1.5,1.00E-03,1,50001,1,1,1,1,1,1,1,1,1,1,0.136343,0,1,1,1,0,9.029568002,19.97511375,10.81977828,1,0,1,0,1,0,
Gene content,32,HolXen-ne,HomogroupsneP,1.5,1.00E-03,2,50001,1,1,1,1,1,1,1,1,1,1,0.136263,0,1,1,1,0,9.029568002,19.97511375,10.81977828,1,0,1,0,1,0,
Gene content,32,HolXen-ne,HomogroupsneP,1.5,1.00E-03,3,50001,1,1,1,1,1,1,1,1,1,1,0.1431962,0,1,1,1,0,9.029568002,19.97511375,10.81977828,1,0,1,0,1,0,
Gene content,32,HolXen-ne,HomogroupsneP,1.5,1.00E-03,4,50001,1,1,1,1,1,1,1,1,1,1,0.1361297,0,1,1,1,0,9.029568002,19.97511375,10.81977828,1,0,1,0,1,0,
Gene content,32,HolXen-ne,OrthogroupsneAb,1.5,1.00E-03,1,40001,1,1,1,1,1,1,1,1,1,1,0.989967,0,1,1,1,0,8.806424451,19.7519702,10.59663473,1,0,1,0,1,0,
Gene content,32,HolXen-ne,OrthogroupsneAb,1.5,1.00E-03,2,40001,1,1,1,1,1,1,1,1,1,1,0.9916336,0,1,1,1,0,8.806424451,19.7519702,10.59663473,1,0,1,0,1,0,
Gene content,32,HolXen-ne,OrthogroupsneAb,1.5,1.00E-03,3,40001,1,1,1,1,1,1,1,1,1,1,0.991367,0,1,1,1,0,8.806424451,19.7519702,10.59663473,1,0,1,0,1,0,
Gene content,32,HolXen-ne,OrthogroupsneAb,1.5,1.00E-03,4,40001,1,1,1,1,1,1,1,1,1,1,0.9934002,0,1,1,1,0,8.806424451,19.7519702,10.59663473,1,0,1,0,1,0,
Gene content,32,HolXen-ne,OrthogroupsneP,1.5,1.00E-03,1,40001,1,1,1,1,1,1,1,1,1,1,0.9998333,0,1,1,1,0,8.806424451,19.7519702,10.59663473,1,0,1,0,1,0,
Gene content,32,HolXen-ne,OrthogroupsneP,1.5,1.00E-03,2,40001,1,1,1,1,1,1,1,1,1,1,0.9999,0,1,1,1,0,8.806424451,19.7519702,10.59663473,1,0,1,0,1,0,
Gene content,32,HolXen-ne,OrthogroupsneP,1.5,1.00E-03,3,40001,1,1,1,1,1,1,1,1,1,1,0.9996,0,1,1,1,0,8.806424451,19.7519702,10.59663473,1,0,1,0,1,0,
Gene content,32,HolXen-ne,OrthogroupsneP,1.5,1.00E-03,4,40001,1,1,1,1,1,1,1,1,1,1,0.9999333,0,1,1,1,0,8.806424451,19.7519702,10.59663473,1,0,1,0,1,0,
Gene content,44,OpiAco-dis,HomogroupsdisAb,1.5,1.00E-03,1,100001,1,1,1,1,1,1,1,0,1,1,0.1623845,0,1,1,1,0,10.73122489,21.63926924,11.51292546,1,0,1,0,1,0,
Gene content,44,OpiAco-dis,HomogroupsdisAb,1.5,1.00E-03,2,100001,1,1,1,1,1,1,1,0.0003866615,1,1,0.1633178,0,1,1,1,0,10.73122489,21.63926924,11.51292546,1,0,1,0,1,0,
Gene content,44,OpiAco-dis,HomogroupsdisAb,1.5,1.00E-03,3,100001,1,1,1,1,1,1,1,0.0003866615,1,1,0.1633178,0,1,1,1,0,10.73122489,21.63926924,11.51292546,1,0,1,0,1,0,
Gene content,44,OpiAco-dis,HomogroupsdisAb,1.5,1.00E-03,4,100001,1,1,1,1,1,1,1,0.0003999947,1,1,0.1576779,0,1,1,1,0,10.73122489,21.63926924,11.51292546,1,0,1,0,1,0,
Gene content,44,OpiAco-dis,HomogroupsdisP,1.5,1.00E-03,1,45735,1,1,1,1,1,1,1,0.002886129,1,1,0.1187394,0,1,1,1,0,9.948896705,20.85694106,10.73059728,1,0,1,0,1,0,
Gene content,44,OpiAco-dis,HomogroupsdisP,1.5,1.00E-03,2,46867,1,1,1,1,1,1,1,8.53E-05,1,1,0.1182328,0,1,1,1,0,9.973347167,20.88139152,10.75504774,1,0,1,0,1,0,
Gene content,44,OpiAco-dis,HomogroupsdisP,1.5,1.00E-03,3,47072,1,1,1,1,1,1,1,0.0002266032,1,1,0.1206945,0,1,1,1,0,9.977711801,20.88575616,10.75941238,1,0,1,0,1,0,
Gene content,44,OpiAco-dis,HomogroupsdisP,1.5,1.00E-03,4,45660,1,1,1,1,1,1,1,0.001547671,1,1,0.1193459,0,1,1,1,0,9.947255441,20.8552998,10.72895602,1,0,1,0,1,0,
Gene content,44,OpiAco-dis,OrthogroupsdisAb,1.5,1.00E-03,1,240004,1,1,1,1,1,1,1,0.8339972,1,1,0.9214291,0,1,1,1,0,11.60670612,22.51475048,12.3884067,1,0,1,0,1,0,
Gene content,44,OpiAco-dis,OrthogroupsdisAb,1.5,1.00E-03,2,60001,1,1,1,1,1,1,1,0.7555832,1,1,0.9156463,0,1,1,1,0,10.22039926,21.12844362,11.00209984,1,0,1,0,1,0,
Gene content,44,OpiAco-dis,OrthogroupsdisAb,1.5,1.00E-03,3,60001,1,1,1,1,1,1,1,0.7555832,1,1,0.9156463,0,1,1,1,0,10.22039926,21.12844362,11.00209984,1,0,1,0,1,0,
Gene content,44,OpiAco-dis,OrthogroupsdisAb,1.5,1.00E-03,4,60001,1,1,1,1,1,1,1,0.9003355,1,1,0.9282683,0,1,1,1,0,10.22039926,21.12844362,11.00209984,1,0,1,0,1,0,
Gene content,44,OpiAco-dis,OrthogroupsdisP,1.5,1.00E-03,1,40001,1,1,1,1,1,1,1,0.9999667,1,1,0.9975667,0,1,1,1,0,9.814934155,20.72297851,10.59663473,1,0,1,0,1,0,
Gene content,44,OpiAco-dis,OrthogroupsdisP,1.5,1.00E-03,2,40001,1,1,1,1,1,1,1,1,1,1,0.9977001,0,1,1,1,0,9.814934155,20.72297851,10.59663473,1,0,1,0,1,0,
Gene content,44,OpiAco-dis,OrthogroupsdisP,1.5,1.00E-03,3,40001,1,1,1,1,1,1,1,1,1,1,0.9967001,0,1,1,1,0,9.814934155,20.72297851,10.59663473,1,0,1,0,1,0,
Gene content,44,OpiAco-dis,OrthogroupsdisP,1.5,1.00E-03,4,40001,1,1,1,1,1,1,1,1,1,1,0.9967001,0,1,1,1,0,9.814934155,20.72297851,10.59663473,1,0,1,0,1,0,
Gene content,41,OpiAco-ne,HomogroupsneAb,1.5,1.00E-03,1,43501,1,1,1,1,1,1,1,1,1,1,0.2060933,0,1,1,1,0,10.00606117,19.65401138,10.68051622,1,0,1,0,1,0,
Gene content,41,OpiAco-ne,HomogroupsneAb,1.5,1.00E-03,2,49885,1,1,1,1,1,1,1,1,1,1,0.2138237,0,1,1,1,0,10.14300054,19.79095076,10.81745559,1,0,1,0,1,0,

Gene content,41,OpiAco-ne,HomogroupsneAb,1.5,1.00E-03,3,38794,1,1,1,1,1,1,1,1,1,0.2167308,0,1,1,1,0,9.891540049,19.53949026,10.5659951,1,0,1,0,1,0,
Gene content,41,OpiAco-ne,HomogroupsneAb,1.5,1.00E-03,4,38736,1,1,1,1,1,1,1,1,1,0.216646,0,1,1,1,0,9.890043816,19.53799403,10.56449886,1,0,1,0,1,0,
Gene content,41,OpiAco-ne,HomogroupsneP,1.5,1.00E-03,1,70001,1,1,1,1,1,1,1,1,1,0.0930268,0,1,1,1,0,10.48179547,20.12974569,11.15625052,1,0,1,0,1,0,
Gene content,41,OpiAco-ne,HomogroupsneP,1.5,1.00E-03,2,70001,1,1,1,1,1,1,1,1,1,0.0926649,0,1,1,1,0,10.48179547,20.12974569,11.15625052,1,0,1,0,1,0,
Gene content,41,OpiAco-ne,HomogroupsneP,1.5,1.00E-03,3,70001,1,1,1,1,1,1,1,1,1,0.08799832,0,1,1,1,0,10.48179547,20.12974569,11.15625052,1,0,1,0,1,0,
Gene content,41,OpiAco-ne,HomogroupsneP,1.5,1.00E-03,4,70001,1,1,1,1,1,1,1,1,1,0.09445534,0,1,1,1,0,10.48179547,20.12974569,11.15625052,1,0,1,0,1,0,
Gene content,41,OpiAco-ne,OrthogroupsneAb,1.5,1.00E-03,1,70001,1,1,1,1,1,1,1,1,1,0.9897335,0,1,1,1,0,10.48179547,20.12974569,11.15625052,1,0,1,0,1,0,
Gene content,41,OpiAco-ne,OrthogroupsneAb,1.5,1.00E-03,2,70001,1,1,1,1,1,1,1,1,1,0.9869907,0,1,1,1,0,10.48179547,20.12974569,11.15625052,1,0,1,0,1,0,
Gene content,41,OpiAco-ne,OrthogroupsneAb,1.5,1.00E-03,3,70001,1,1,1,1,1,1,1,1,1,0.9878669,0,1,1,1,0,10.48179547,20.12974569,11.15625052,1,0,1,0,1,0,
Gene content,41,OpiAco-ne,OrthogroupsneAb,1.5,1.00E-03,4,70001,1,1,1,1,1,1,1,1,1,0.9878669,0,1,1,1,0,10.48179547,20.12974569,11.15625052,1,0,1,0,1,0,
Gene content,41,OpiAco-ne,OrthogroupsneP,1.5,1.00E-03,1,60001,1,1,1,1,1,1,1,1,1,0.9984223,0,1,1,1,0,10.32764479,19.97559501,11.00209984,1,0,1,0,1,0,
Gene content,41,OpiAco-ne,OrthogroupsneP,1.5,1.00E-03,2,60001,1,1,1,1,1,1,1,1,1,0.9988,0,1,1,1,0,10.32764479,19.97559501,11.00209984,1,0,1,0,1,0,
Gene content,41,OpiAco-ne,OrthogroupsneP,1.5,1.00E-03,3,60001,1,1,1,1,1,1,1,1,1,0.9986445,0,1,1,1,0,10.32764479,19.97559501,11.00209984,1,0,1,0,1,0,
Gene content,41,OpiAco-ne,OrthogroupsneP,1.5,1.00E-03,4,60001,1,1,1,1,1,1,1,1,1,0.9986445,0,1,1,1,0,10.32764479,19.97559501,11.00209984,1,0,1,0,1,0,
Gene content,47,Opi-dis,HomogroupsdisAb,1.5,1.00E-03,1,60001,1,1,1,1,1,1,0,1,1,0.1132197,0,1,1,1,0,5.474942453,18.40784095,11.00209984,1,0,1,0,1,0,
Gene content,47,Opi-dis,HomogroupsdisAb,1.5,1.00E-03,2,60001,1,1,1,1,1,1,0,1,1,0.1107087,0,1,1,1,0,5.474942453,18.40784095,11.00209984,1,0,1,0,1,0,
Gene content,47,Opi-dis,HomogroupsdisAb,1.5,1.00E-03,3,60001,1,1,1,1,1,1,0,1,1,0.1148863,0,1,1,1,0,5.474942453,18.40784095,11.00209984,1,0,1,0,1,0,
Gene content,47,Opi-dis,HomogroupsdisAb,1.5,1.00E-03,4,60001,1,1,1,1,1,1,0,1,1,0.1131975,0,1,1,1,0,5.474942453,18.40784095,11.00209984,1,0,1,0,1,0,
Gene content,47,Opi-dis,OrthogroupsdisAb,1.5,1.00E-03,1,6001,1,1,1,1,1,1,0.9937792,1,1,0.9997778,0,1,1,1,0,3.17235736,16.10525586,8.699514748,0,0,1,0,1,0,
Gene content,47,Opi-dis,OrthogroupsdisAb,1.5,1.00E-03,2,6001,1,1,1,1,1,1,0.9997778,1,1,0.9995557,0,1,1,1,0,3.17235736,16.10525586,8.699514748,0,0,1,0,1,0,
Gene content,47,Opi-dis,OrthogroupsdisAb,1.5,1.00E-03,3,6001,1,1,1,1,1,1,1,1,1,0.9993335,0,1,1,1,0,3.17235736,16.10525586,8.699514748,0,0,1,0,1,0,
Gene content,47,Opi-dis,OrthogroupsdisAb,1.5,1.00E-03,4,6001,1,1,1,1,1,1,1,1,1,0.9995557,0,1,1,1,0,3.17235736,16.10525586,8.699514748,0,0,1,0,1,0,
Gene content,44,Opi-ne,HomogroupsneAb,1.5,1.00E-03,1,40001,1,1,1,1,1,1,1,1,1,0.5591147,0,1,1,1,0,5.525177133,16.97469038,10.59663473,1,0,1,0,1,0,
Gene content,44,Opi-ne,HomogroupsneAb,1.5,1.00E-03,2,40001,1,1,1,1,1,1,1,1,1,0.5479484,0,1,1,1,0,5.525177133,16.97469038,10.59663473,1,0,1,0,1,0,
Gene content,44,Opi-ne,HomogroupsneAb,1.5,1.00E-03,3,40001,1,1,1,1,1,1,1,1,1,0.5504817,0,1,1,1,0,5.525177133,16.97469038,10.59663473,1,0,1,0,1,0,
Gene content,44,Opi-ne,HomogroupsneAb,1.5,1.00E-03,4,40001,1,1,1,1,1,1,1,1,1,0.5242825,0,1,1,1,0,5.525177133,16.97469038,10.59663473,1,0,1,0,1,0,
Gene content,44,Opi-ne,HomogroupsneP,1.5,1.00E-03,1,64366,1,1,1,1,1,1,1,1,1,0.1534749,0,1,1,1,0,6.000867686,17.45038093,11.07232529,1,0,1,0,1,0,
Gene content,44,Opi-ne,HomogroupsneP,1.5,1.00E-03,2,53279,1,1,1,1,1,1,1,1,1,0.1499249,0,1,1,1,0,5.811821167,17.26133441,10.88327877,1,0,1,0,1,0,
Gene content,44,Opi-ne,HomogroupsneP,1.5,1.00E-03,3,57113,1,1,1,1,1,1,1,1,1,0.1566476,0,1,1,1,0,5.881311931,17.33082517,10.95276953,1,0,1,0,1,0,
Gene content,44,Opi-ne,HomogroupsneP,1.5,1.00E-03,4,62923,1,1,1,1,1,1,1,1,1,0.1474159,0,1,1,1,0,5.978193543,17.42770679,11.04965114,1,0,1,0,1,0,
Gene content,44,Opi-ne,OrthogroupsneAb,1.5,1.00E-03,1,100001,1,1,1,1,1,1,1,1,1,0.9996133,0,1,1,1,0,6.441467865,17.89098111,11.51292546,1,0,1,0,1,0,
Gene content,44,Opi-ne,OrthogroupsneAb,1.5,1.00E-03,2,100001,1,1,1,1,1,1,1,1,1,0.9996933,0,1,1,1,0,6.441467865,17.89098111,11.51292546,1,0,1,0,1,0,
Gene content,44,Opi-ne,OrthogroupsneAb,1.5,1.00E-03,3,100001,1,1,1,1,1,1,1,1,1,0.9996533,0,1,1,1,0,6.441467865,17.89098111,11.51292546,1,0,1,0,1,0,
Gene content,44,Opi-ne,OrthogroupsneAb,1.5,1.00E-03,4,100001,1,1,1,1,1,1,1,1,1,0.9995067,0,1,1,1,0,6.441467865,17.89098111,11.51292546,1,0,1,0,1,0,
Gene content,44,Opi-ne,OrthogroupsneP,1.5,1.00E-03,1,100001,1,1,1,1,1,0.9368942,1,1,1,0.99972,0,1,0,0.9368542,0.06314582,6.441467865,9.075136655,-11.51292546,1,0,1,0,0,1,
Gene content,44,Opi-ne,OrthogroupsneP,1.5,1.00E-03,2,100001,1,1,1,1,1,1,1,1,1,0.99992,0,1,1,1,0,6.441467865,17.89098111,11.51292546,1,0,1,0,1,0,
Gene content,44,Opi-ne,OrthogroupsneP,1.5,1.00E-03,3,100001,1,1,1,1,1,0.5815922,1,1,1,0.9997733,0,1,0.3975014,0.5815389,0.4184611,6.441467865,6.707149574,-0.4158868856,1,0,1,0,0,0,
Gene content,44,Opi-ne,OrthogroupsneP,1.5,1.00E-03,4,100001,1,1,1,1,1,0.9364275,1,1,1,0.99972,0,1,0.09634538,0.9363742,0.06362582,6.441467865,9.067051463,-2.238507786,1,0,1,0,0,0,
Gene content,41,OpiXen-dis,HomogroupsdisAb,1.5,1.00E-03,1,40001,1,1,1,1,1,1,0.01253292,1,1,0.6627112,0,1,1,1,0,9.574271837,25.11153969,10.59663473,1,0,1,0,1,0,
Gene content,41,OpiXen-dis,HomogroupsdisAb,1.5,1.00E-03,2,40001,1,1,1,1,1,1,0.03136562,1,1,0.6629779,0,1,1,1,0,9.574271837,25.11153969,10.59663473,1,0,1,0,1,0,
Gene content,41,OpiXen-dis,HomogroupsdisAb,1.5,1.00E-03,3,40001,1,1,1,1,1,1,0.01949935,1,1,0.6736442,0,1,1,1,0,9.574271837,25.11153969,10.59663473,1,0,1,0,1,0,
Gene content,41,OpiXen-dis,HomogroupsdisAb,1.5,1.00E-03,4,40001,1,1,1,1,1,1,0.00389987,1,1,0.6679777,0,1,1,1,0,9.574271837,25.11153969,10.59663473,1,0,1,0,1,0,
Gene content,41,OpiXen-dis,HomogroupsdisP,1.5,1.00E-03,1,47339,1,1,1,1,1,1,0.01664554,1,1,0.2822701,0,1,1,1,0,9.742705738,25.2799736,10.76506863,1,0,1,0,1,0,
Gene content,41,OpiXen-dis,HomogroupsdisP,1.5,1.00E-03,2,50001,1,1,1,1,1,1,0.01991947,1,1,0.2845524,0,1,1,1,0,9.797415388,25.33468324,10.81977828,1,0,1,0,1,0,
Gene content,41,OpiXen-dis,HomogroupsdisP,1.5,1.00E-03,3,42394,1,1,1,1,1,1,0.0123915,1,1,0.2744056,0,1,1,1,0,9.632375637,25.16964349,10.65473853,1,0,1,0,1,0,
Gene content,41,OpiXen-dis,HomogroupsdisP,1.5,1.00E-03,4,48242,1,1,1,1,1,1,0.004035156,1,1,0.2786745,0,1,1,1,0,9.761601665,25.29886952,10.78396456,1,0,1,0,1,0,
Gene content,41,OpiXen-dis,OrthogroupsdisAb,1.5,1.00E-03,1,40001,1,1,1,1,1,1,0.9500683,1,1,0.7683411,0,1,1,1,0,9.574271837,25.11153969,10.59663473,1,0,1,0,1,0,
Gene content,41,OpiXen-dis,OrthogroupsdisAb,1.5,1.00E-03,2,40001,1,1,1,1,1,1,0.9209693,1,1,0.7570414,0,1,1,1,0,9.574271837,25.11153969,10.59663473,1,0,1,0,1,0,
Gene content,41,OpiXen-dis,OrthogroupsdisAb,1.5,1.00E-03,3,40001,1,1,1,1,1,1,0.8540382,1,1,0.7763741,0,1,1,1,0,9.574271837,25.11153969,10.59663473,1,0,1,0,1,0,
Gene content,41,OpiXen-dis,OrthogroupsdisAb,1.5,1.00E-03,4,40001,1,1,1,1,1,1,0.9716343,1,1,0.7573748,0,1,1,1,0,9.574271837,25.11153969,10.59663473,1,0,1,0,1,0,
Gene content,41,OpiXen-dis,OrthogroupsdisP,1.5,1.00E-03,1,40001,1,1,1,1,1,1,0.9998333,1,1,0.9995667,0,1,1,1,0,9.574271837,25.11153969,10.59663473,1,0,1,0,1,0,
Gene content,41,OpiXen-dis,OrthogroupsdisP,1.5,1.00E-03,2,40001,1,1,1,1,1,1,0.9999333,1,1,0.9991334,0,1,1,1,0,9.574271837,25.11153969,10.59663473,1,0,1,0,1,0,
Gene content,41,OpiXen-dis,OrthogroupsdisP,1.5,1.00E-03,3,40001,1,1,1,1,1,1,0.9999333,1,1,0.9991334,0,1,1,1,0,9.574271837,25.11153969,10.59663473,1,0,1,0,1,0,
Gene content,41,OpiXen-dis,OrthogroupsdisP,1.5,1.00E-03,4,40001,1,1,1,1,1,1,1,1,1,0.9995333,0,1,1,1,0,9.574271837,25.11153969,10.59663473,1,0,1,0,1,0,
Gene content,38,OpiXen-ne,HomogroupsneAb,1.5,1.00E-03,1,40001,1,1,1,1,1,1,1,1,1,0.9546348,0,1,1,1,0,9.830055162,23.54839452,10.59663473,1,0,1,0,1,0,
Gene content,38,OpiXen-ne,HomogroupsneAb,1.5,1.00E-03,2,40001,1,1,1,1,1,1,1,1,1,0.9538349,0,1,1,1,0,9.830055162,23.54839452,10.59663473,1,0,1,0,1,0,
Gene content,38,OpiXen-ne,HomogroupsneAb,1.5,1.00E-03,3,40001,1,1,1,1,1,1,1,1,1,0.9646345,0,1,1,1,0,9.830055162,23.54839452,10.59663473,1,0,1,0,1,0,
Gene content,38,OpiXen-ne,HomogroupsneAb,1.5,1.00E-03,4,40001,1,1,1,1,1,1,1,1,1,0.9567348,0,1,1,1,0,9.830055162,23.54839452,10.59663473,1,0,1,0,1,0,
Gene content,38,OpiXen-ne,HomogroupsneP,1.5,1.00E-03,1,50001,1,1,1,1,1,1,1,1,1,0.4448148,0,1,1,1,0,10.05319871,23.77153807,10.81977828,1,0,1,0,1,0,
Gene content,38,OpiXen-ne,HomogroupsneP,1.5,1.00E-03,2,48345,1,1,1,1,1,1,1,1,1,0.4611545,0,1,1,1,0,10.01951783,23.73785719,10.7860974,1,0,1,0,1,0,
Gene content,38,OpiXen-ne,HomogroupsneP,1.5,1.00E-03,3,48829,1,1,1,1,1,1,1,1,1,0.4604336,0,1,1,1,0,10.02947963,23.74781899,10.7960592,1,0,1,0,1,0,
Gene content,38,OpiXen-ne,HomogroupsneP,1.5,1.00E-03,4,49331,1,1,1,1,1,1,1,1,1,0.429417,0,1,1,1,0,10.03970812,23.75804748,10.80628769,1,0,1,0,1,0,
Gene content,38,OpiXen-ne,OrthogroupsneAb,1.5,1.00E-03,1,40001,1,1,1,1,1,1,1,1,1,0.9731676,0,1,1,1,0,9.830055162,23.54839452,10.59663473,1,0,1,0,1,0,
Gene content,38,OpiXen-ne,OrthogroupsneAb,1.5,1.00E-03,2,40001,1,1,1,1,1,1,1,1,1,0.9755341,0,1,1,1,0,9.830055162,23.54839452,10.59663473,1,0,1,0,1,0,
Gene content,38,OpiXen-ne,OrthogroupsneAb,1.5,1.00E-03,3,40001,1,1,1,1,1,1,1,1,1,0.9733676,0,1,1,1,0,9.830055162,23.54839452,10.59663473,1,0,1,0,1,0,
Gene content,38,OpiXen-ne,OrthogroupsneAb,1.5,1.00E-03,4,40001,1,1,1,1,1,1,1,1,1,0.9784674,0,1,1,1,0,9.830055162,23.54839452,10.59663473,1,0,1,0,1,0,
Gene content,38,OpiXen-ne,OrthogroupsneP,1.5,1.00E-03,1,40001,1,1,1,1,1,1,1,1,1,1,0,1,1,1,0,9.830055162,23.54839452,10.59663473,1,0,1,0,1,0,
Gene content,38,OpiXen-ne,OrthogroupsneP,1.5,1.00E-03,2,40001,1,1,1,1,1,1,1,1,1,0.9999333,0,1,1,1,0,9.830055162,23.54839452,10.59663473,1,0,1,0,1,0,
Gene content,38,OpiXen-ne,OrthogroupsneP,1.5,1.00E-03,3,40001,1,1,1,1,1,1,1,1,1,0.9999333,0,1,1,1,0,9.830055162,23.54839452,10.59663473,1,0,1,0,1,0,
Gene content,38,OpiXen-ne,OrthogroupsneP,1.5,1.00E-03,4,40001,1,1,1,1,1,1,1,1,1,0.9999333,0,1,1,1,0,9.830055162,23.54839452,10.59663473,1,0,1,0,1,0,
Sum for support all,,,,,,,,,,,,,,,,,,,,,,,566,1,583,112,533,125,
Sum for support Cho,,,,,,,,,,,,,,,,,,,,,,,55,1,31,16,44,16,
Sum for support Hol,,,,,,,,,,,,,,,,,,,,,,,84,0,84,12,72,24,
Sum for support Opi,,,,,,,,,,,,,,,,,,,,,,,84,0,88,0,85,1,
,,,,,,,,,,,,,,,,,,,,,,,bf_Porifera>5,bf_Porifera<-5,bf_Xenacoelomorpha>5,bf_Xenacoelomorpha<-5,bf_Deutorostome>5,bf_Deutorostome<-5,
