# Supplementary material for: Exploring genome gene content and morphological analysis to test recalcitrant nodes in the animal phylogeny
Source: PLoS One. 2023 Mar 23;18(3):e0282444. doi: 10.1371/journal.pone.0282444 (PMC10035847; doi:10.1371/journal.pone.0282444)
Supplement: S6 Table — (PDF) [file pone.0282444.s020.pdf]

1.5 X 1e-2	2 X 1e-2	2.5 X 1e-2	4 X 1e-2	6 X 1e-2
1.5 X 1e-5	2 X 1e-5	2.5 X 1e-5	4 X 1e-5	6 X 1e-5
1.5 X 1e-9	2 X 1e-9	2.5 X 1e-9	4 X 1e-9	6 X 1e-9
1.5 X 1e-12	2 X 1e-12	2.5 X 1e-12	4 X 1e-12	6 X 1e-12

Supplementary Table 6: The 20 different parameter combinations for each dataset tested in Opi (47 taxa), Aco (44 taxa) and Xen (41 taxa) taxon samplings.
