# Supplementary material for: Exploring genome gene content and morphological analysis to test recalcitrant nodes in the animal phylogeny
Source: PLoS One. 2023 Mar 23;18(3):e0282444. doi: 10.1371/journal.pone.0282444 (PMC10035847; doi:10.1371/journal.pone.0282444)
Supplement: S7 Table — (PDF) [file pone.0282444.s021.pdf]

Outgroup sampling and method used		Opi (47 sp)	Aco (44 sp)	Xen (41 sp)
Opisthokonta (no reduced outgroup sampling)	Method A	Opi-A	OpiAco-A	OpiXen-A
	Method B	-	OpiAco-B	OpiXen-B
Holozoa	Method A	Hol-A	HolAco-A	HolXen-A
	Method B	Hol-B	HolAco-B	HolXen-B
Choanozoa	Method A	Cho-A	ChoAco-A	ChoXen-A
	Method B	Cho-B	ChoAco-B	ChoXen-B

Supplementary Table 7: The reduced outgroup sampling dataset designations.
