# Supplementary material for: Exploring genome gene content and morphological analysis to test recalcitrant nodes in the animal phylogeny
Source: PLoS One. 2023 Mar 23;18(3):e0282444. doi: 10.1371/journal.pone.0282444 (PMC10035847; doi:10.1371/journal.pone.0282444)
Supplement: S8 Table — (PDF) [file pone.0282444.s022.pdf]

Outgroup sampling and method used		**Opi-ne (44 sp)**	**Aco-ne (41 sp)**	**Xen-ne (38 sp)**
Opisthokonta (no reduced outgroup sampling)	Method A	Opi-neA	OpiAco-neA	OpiXen-neA
	Method B	Opi-neB	OpiAco-neB	OpiXen-neB
Holozoa	Method A	Hol-neA	HolAco-neA	HolXen-neA
	Method B	Hol-neB	HolAco-neB	HolXen-neB
Choanozoa	Method A	Cho-neA	ChoAco-neA	ChoXen-neA
	Method B	Cho-neB	ChoAco-neB	ChoXen-neB

Supplementary Table 8: The reduced outgroup and ingroup sampling performed according to the dataset naming list as presented in Supplementary Table 4.
