# Supplementary material for: Exploring genome gene content and morphological analysis to test recalcitrant nodes in the animal phylogeny
Source: PLoS One. 2023 Mar 23;18(3):e0282444. doi: 10.1371/journal.pone.0282444 (PMC10035847; doi:10.1371/journal.pone.0282444)
Supplement: S1 File — (PDF) [file pone.0282444.s023.pdf]

1. Supplementary Data 1 - Methodology used to construct the datasets

1.1. Gene content analysis

A Figure (All_graph.p.png/pdf) showing the full pipeline of dataset construction is available at https://github.com/PalMuc/triangulation/tree/main/Additional%20information

Data and code used to construct the datasets are available in the data repository at
https://github.com/PalMuc/triangulation/tree/main/data_matrices_gene_content
https://github.com/PalMuc/triangulation/tree/main/Code

1.2. Morphology

1.2.1. Character list construction

In addition to the three main morphological datasets that were combined [1–3], a few additional characters were added to improve the resolution of the outgroups: five characters common to all Fungi [4] and two synapomorphies present in both choanoflagellate taxa were included in the analysis [based on 5]. Additionally, three other important characters were noticed to be missing in the combined dataset and subsequently were added: Schizocoely (char. 108), which is known to be one of the most important synapomorphies of protostomes (at least for the taxa sampled in our set, since chaetognaths and other groups have a different mode akin to Deuterostome development) and two general characters for muscles and excretory organs (char. 55 and 106). Furthermore, three missing sponge synapomorphies were added: a general character for the presence of sclerocytes (char. 33), the presence of an osculum (char. 768) and their cellular totipotency (char. 769). A few changes were made to five characters (Supplement 4). The coding of several characters related to cilia and statoliths within Xenacoelomorpha (characters 92 and 95) were modified in order to reflect up to date interpretations based on comparative anatomy [6]. The resulting combined dataset contains 770 characters, that are mostly binary absence/presence, except for five characters that have three states.

Coding strategies

In order to evaluate the effect of different coding strategies for absent character states, two separate data matrices were produced. In the first coding strategy, all absent states are

interpreted as true absence (coded as 0; also known as non-additive coding), whereas the second coding strategy includes character dependencies based on the original reductive coding in Deline et al. [3], which distinguishes between absent and non-applicable. The non-additive coding is unrealistic because it does not respect the principle of character independence [7], but it is useful for our study because the taxonomic sample in our character matrix is very disparate and a significant proportion of the character states are inapplicable for many of the taxa. The outgroups, for example, are not animals, and most of our set is composed of animal characters, therefore they are scored as unknown for the majority of characters. By simplifying the scoring we can observe the general trends without the increased uncertainty.

1.2.2. Additional comments about the morphological topologies

Vertebrates appear in a polytomy with Cephalochordata and Urochordata, which is in agreement with the equivocal morphological support for the three possible topologies [8]. Resolving the internal relationships within Porifera requires better taxon sampling. The taxon sampling here was intentionally restricted to match the 47 species taxon sampling of the genome gene content dataset to make results comparable and avoid biases in unequal taxon composition. Consequently, class Hexactinellida was not represented here because there are no genomes available and it could also not be included in the gene content analyses. Phylogenomic analyses generally support a sister relationship between the Calcarea and Homoscleromorpha [9], whereas the gene content dataset supports either the phylogenomic topology or Calcarea + Demospongiae. The non-additive coded matrices support Homoscleromorpha + Demospongiae based on the presence of silica spicules (see Morphological trees in the data repository), whereas the reductive coded matrix finds sponges paraphyletic at the base of Metazoa.

These differences are likely influenced by the limitations of our morphological analyses, which are very sensitive to differences in intrinsic anatomical complexity. In the non-additive set, all the complexity of eumetazoans produces a clear distinction between Porifera and Placozoa in one clade and the rest of the animals in another clade. In contrast, the reductive coding leads to a major decrease in the percentage of characters that sponges and placozoans can be coded for, introducing significant uncertainty in the estimated tree (i.e., lower posterior probabilities). The fact that the morphological dataset includes both unicellular

organisms and vertebrates creates a major challenge for morphology since non-additive coding leads to very notable branch length gradients due to differences in character complexity/availability. This same issue is the source of the significantly lower node support across the whole tree when using the reductive-coded matrix. The analysis of different versions of the morphological dataset as it was being constructed showed that the relative position of ctenophores and cnidarians is dependent on coding hypotheses, but none of those analyses ever supported ctenophora-sister.
