# Supplementary material for: Exploring genome gene content and morphological analysis to test recalcitrant nodes in the animal phylogeny
Source: PLoS One. 2023 Mar 23;18(3):e0282444. doi: 10.1371/journal.pone.0282444 (PMC10035847; doi:10.1371/journal.pone.0282444)
Supplement: S2 File — (PDF) [file pone.0282444.s024.pdf]

1. Supplementary Data 2 - Analyses of the genome gene content datasets

1.1. Individual trees for each combination of parameters

In total, 380 phylogenies were generated, divided into two replicates (Run 1, Run 2), where the whole workflow from dataset construction to phylogenetic analysis was performed starting from the 47 proteomes.

Each of the 190 datasets were divided based on two main settings.

First, three taxon subgroups were created based on the number of species in each: Opisthokonta (Opi - 47 taxa), Acoelomorpha without *Xenoturbella bocki* (Aco - 44 taxa), and *Xenoturbella bocki* without Acoelomorpha (Xen - 41 taxa). Then, four different similarity (E) and five inflation (I) values (Supp. Tab. 6) were applied to analyse each of these subgroups, resulting in 20 datasets each (60 in total).

For each combination of parameters, homolog- and ortholog-based genome gene content prediction was carried out for dataset construction, resulting in 120 phylogenies in total (3 taxon samplings x 20 parameter combination (see Supp. Table 6) x 2 homologous and orthologous gene content).

1.5 X 1e-2	2 X 1e-2	2.5 X 1e-2	4 X 1e-2	6 X 1e-2
1.5 X 1e-5	2 X 1e-5	2.5 X 1e-5	4 X 1e-5	6 X 1e-5
1.5 X 1e-9	2 X 1e-9	2.5 X 1e-9	4 X 1e-9	6 X 1e-9
1.5 X 1e-12	2 X 1e-12	2.5 X 1e-12	4 X 1e-12	6 X 1e-12

Supplementary Table 6: The 20 different parameter combinations for each dataset tested in Opi (47 taxa), Aco (44 taxa) and Xen (41 taxa) taxon samplings.

1.2. Individual tree for each combination of taxa sampling

1.2.1. Outgroup reduction

Second, the default I- and E-value of 1.5 and 1e-3 (in MCL and DIAMOND) were applied as described before on the three different taxon samplings (Opi, Aco, and Xen). Additionally, the outgroup sampling was reduced for each of the three different taxon samplings (Opi, Aco, and Xen) by following the taxon sampling of: i) the complete taxon sampling; ii) Ichthyosporea + Choanoflagellata + Metazoa (= Holozoa; dataset prefix Holo), and iii)

Choanoflagellata + Metazoa (= Choanozoa; dataset prefix Cho) [10] , and two methodologies for dataset creation (Pruning and Ab Initio, here referred as A and B for simplicity) were applied to generate the final matrices.

This setting results in 17 new combinations. The Opi-B dataset was not possible to generate, because the Pruning method is deleting species from the final matrix to create a new matrix with fewer species, instead of rerunning all the pipeline, and this method can only create matrices with fewer than 47 taxa (the initial full Opi [47 species] dataset consisted of 47 taxa). For each combination described in the table, homologous and orthologous based gene content prediction was carried out to construct the presence/absence data matrix used for phylogenetic analyses. This resulted in 34 phylogenies (3 taxon samplings x 6 taxon samplings of outgroups and methodology combination x 2 homologous and orthologous predicted datasets, minus the 2 combinations which were not possible to generate).

The resulting combinations are:

Outgroup sampling and method used		Opi (47 sp)	Aco (44 sp)	Xen (41 sp)
Opisthokonta (no reduced outgroup sampling)	Method A	Opi-A	OpiAco-A	OpiXen-A
	Method B	-	OpiAco-B	OpiXen-B
Holozoa	Method A	Hol-A	HolAco-A	HolXen-A
	Method B	Hol-B	HolAco-B	HolXen-B
Choanozoa	Method A	Cho-A	ChoAco-A	ChoXen-A
	Method B	Cho-B	ChoAco-B	ChoXen-B

Supplementary Table 7: The reduced outgroup sampling dataset designations.

1.2.2. Outgroup and long branched ingroup reduction

The procedure described in the previous section was repeated for datasets without the ingroup species *Caenorhabditis elegans* (Nematoda), *Pristionchus pacificus* (Nematoda), and *Schistosoma mansoni* (Platyhelminthes) per reduced outgroup dataset. These are the excluded "near" long branch species (Suffix ne) in the datasets (see Supp. Table 8).

This step resulted in 36 additional phylogenies (3 taxon samplings x 6 taxon samplings of outgroups and ingroups with the methodology combination x 2 homologous and orthologous predicted datasets).

The dataset construction and phylogenetic analyses were performed twice, each time resulting in 190 phylogenies.

Outgroup sampling and method used		**Opi-ne (44 sp)**	**Aco-ne (41 sp)**	**Xen-ne (38 sp)**
Opisthokonta (no reduced outgroup sampling)	Method A	Opi-neA	OpiAco-neA	OpiXen-neA
	Method B	Opi-neB	OpiAco-neB	OpiXen-neB
Holozoa	Method A	Hol-neA	HolAco-neA	HolXen-neA
	Method B	Hol-neB	HolAco-neB	HolXen-neB
Choanozoa	Method A	Cho-neA	ChoAco-neA	ChoXen-neA
	Method B	Cho-neB	ChoAco-neB	ChoXen-neB

Supplementary Table 8: The reduced outgroup and ingroup sampling performed according to the dataset naming list as presented in Supplementary Table 4.

1.2.3. Supplementary results and discussion from both analyses

See Suppl. Tables 2 and 3 for all details in each run and Supp. Table 3 for the trends examination in the different individual trees, at https://github.com/PalMuc/triangulation/tree/main/Tables

The overall all topologies count and support was calculated as the percentage of individual posterior trees from the total number of trees with the same study case only for run 2 (Supp. Table 3). The graphical summary of these results is displayed in Supp. Fig. 1 (see also data repository for further details). Each part of Supp Fig. 1 is described and discussed in detail below.

1.2.3.1. Results and Discussion of Supplementary Figure 1-A

Results: I-values have a more significant effect than E-values on the number of predicted homo-/orthogroups (gene families).

Discussion: While it can be expected that many protein families are evolutionary related and evolved through processes of gene duplication, the exact composition (in terms of orthology

groups included) of a protein family can be difficult to identify. This is because the similarity of very distantly related paralogs can be minimal, and at some point, as we move backward in evolutionary history, whether two protein families should be merged into a single, larger, superfamily or not becomes difficult to decide. In software such as Orthofinder, the I-values are used to decide the extent to which orthogroups should be merged into a single homogroup (i.e., into the same family). With a larger I-value (high granularity), a large number of smaller (i.e., including fewer orthogroups) gene families are identified. Smaller I-value (low granularity), leads to the inference of less gene families which however include more orthogroups. Changes in E-values, differently, influence the number of sequences retained in each cluster (be that homo- or orthogroup). The smaller the E-value needed to accept an individual sequence as a member of a cluster, the smaller the number of sequences identified to belong to each cluster and the higher the number of singletons identified. Taxon inclusivity will also change with E-values, as higher E-values might fail to identify sequences from distantly related taxa as members of a given cluster. Combining the two parameters (specific choices of E- and I-values), as expected, resulted in homogroup datasets with variable numbers of characters, and their analyses resulted in a greater diversity of inferred trees. As expected, orthogroup-based datasets included more characters (homogroups generally include muliple orthogroups), and their analyses inferred less variable trees. We suggest that this result was to be expected as orthogroups can be partitioned across different homogroups (when changing the I-value), but when homogroups are atomized in their constituent orthogroups, the same set of orthogroups should be identified. Differences in orthogroup composition are driven by E-values rather than I-values. However, this does not necessarily mean that orthogroups are more reliable markers in genome gene content studies. Homo- and orthogroups have different strengths and weaknesses. Homogroups – if too high E-values are used in the context of low granularity analyses – might cluster orthogroups that are not homologous, introducing homoplasy in the data. However, homogroups accumulate losses more slowly than orthogroups. In this way, they are expected to be less homoplastic. Further studies will be needed to understand better what coding strategy is best in genome gene content studies. It can be hypothesised that homogroups inferred using optimal I- and E-values would be most reliable, but how to identify optimal values for these parameters need to be further investigated.

Given the current uncertainty on how best to assemble datasets for genome gene content analyses, we have here taken the approach of testing a large range of I-values,

E-values, and both ortho and homogroups. Results are mostly consistent with those in Fig. 2 suggesting the pattern described to be robust.

1.2.3.2. Results and Discussion of Supplementary Figure 1-B

Results: Matrices with the fewest taxon ingroup number (Xen datasets) show the highest variation in the ranges of predicted numbers of gene families for each treatment (e.g., Pruning method [P], outgroup reduction [Dis], and outgroup and ingroup reduction [Ne]). The largest variation was obtained for the Xen-Holozoa datasets, followed by the homogroups predicted datasets with the Ne treatment (Reduction of ingroups, species with long branches, and outgroups; see Methods). Also, the same treatment yields the largest variation of the predicted number of characters for orthogroup datasets. Both Opi and Aco taxon samplings show the same patterns of the predicted number of characters for all the treatments. The Aco-Choanozoa dataset shows smaller ranges of the predicted number of gene families for all treatments, while for the Opi-Choanozoa dataset the ranges are smaller and without outliers for the treatments of Pruning method (P), outgroup reduction (Dis), and outgroup and ingroup reduction (Ne).

Discussion: The results observed suggest that larger datasets (i.e., more outgroup taxa, larger taxon sample [Opi]) result in a more stable number of gene families in the different treatments. Homogroup-based datasets do not appear to show a distinct trend as well as datasets based on smaller taxon samplings (for both homo- and orthogroups). Together with the conclusions from Supp. Fig. 1A, the combination of dataset size and the type of dataset predicted (homo-/orthogroup) is crucial for the accurate prediction of gene families. Overall, applying the Pruning method shows a more stable range of predicted gene families compared to the *Ab initio* method, but this method can only be applied to reduce the species of interest from an initial fixed set of taxa. The initial set of taxa undergoes *de novo* prediction of gene families, which is a crucial step. Therefore, the secondary reduction (Pruning) has a less significant effect on the number of singletons. Thus, an initially well-balanced and large sample of taxa is crucial.

1.2.3.3. Results and Discussion of Supplementary Figure 1-C

Results: The majority of the MCMC trees supported the Porifera-sister hypothesis. Both data types support the Nephrozoa hypothesis and slightly differ in their support for the monophyly of Deuterostomia. Datasets produced with higher E-values have slightly lower support for

Porifera-sister (they support Placozoa-sister) and slightly higher support for the monophyly of Deuterostomia. Regarding the datasets produced using different I-values, all but Ctenophora-second have lower support datasets constructed with high I-values. The reduction of outgroups only (Dis) and ingroups with outgroups (Ne) showed the same trends in support of the three different hypotheses, i.e., Porifera-sister, Nephrozoa, and monophyletic Deuterostomia. The different outgroup samplings agree on the same overall trend, except the Opisthokonta outgroup sampling has slightly higher support for the Nephrozoa hypothesis than the other two outgroup samplings. Comparing the Pruned datasets to the *Ab initio* created datasets, the results show a similar trend and no difference apart from slightly higher support for the monophyly of Deuterostomia for the *Ab initio* datasets.

Discussion: The results observed suggest that the topology is shown in Figure 2 (main text) for genome gene content is stable and does not change considerably when different outgroups are used. The primary source of difference is the parameter settings used to generate the different types of data (homogroups vs. orthogroups, high vs. low E- and I-values).

1.3. Ortho- and Homogroups based trees for each combination

Supp. Figs 2–5 show the TPCTs for each combination of species (Opi, Aco and Xen) based on the different data types predicted (homogroups and orthogroups) and the different settings used as described in the sections above (Suppl. Data 1 I and Suppl. Data 2 I-II).
