# Supplementary material for: Exploring genome gene content and morphological analysis to test recalcitrant nodes in the animal phylogeny
Source: PLoS One. 2023 Mar 23;18(3):e0282444. doi: 10.1371/journal.pone.0282444 (PMC10035847; doi:10.1371/journal.pone.0282444)
Supplement: S3 File — (PDF) [file pone.0282444.s025.pdf]

1. Supplementary Data 3 - Character list of the morphological analyses

The full character list can be found here:
https://github.com/PalMuc/triangulation/blob/main/Morphology/morpho_character_list.txt

The full character matrices can be found in the data repository at
https://github.com/PalMuc/triangulation/tree/main/Morphology/data_matrices.
