# Supplementary material for: Exploring genome gene content and morphological analysis to test recalcitrant nodes in the animal phylogeny
Source: PLoS One. 2023 Mar 23;18(3):e0282444. doi: 10.1371/journal.pone.0282444 (PMC10035847; doi:10.1371/journal.pone.0282444)
Supplement: S4 File — (PDF) [file pone.0282444.s026.pdf]

1.Supplementary Data 4 - Total Posterior Consensus Tree (*TPCT*)

The total posterior consensus tree is similar in approach to model [11] and data [12] averaging. Our motivation is that we do not condition the specific gene content dataset. For example, we produced different gene content datasets depending on our choice of similarity (E-value) and granulation (I-value). However, we do not know what the "correct" E-value and I-value should be, and therefore average our results over all different datasets produced.

Mathematically, we want to compute the posterior probability of a phylogeny given our genome dataset summed over all E-values and I-values, for example using the gene content orthogroups dataset, which gives

$$P(Tree \mid Genome\ data) = \sum_{i}^{N}\sum_{e}^{M} P(Tree \mid gene\ content\ data_{i,e}) \times P(i) \times P(e),$$

were $P(e)$and $P(i)$ are our prior probabilities for the different E-values and I-values. Additionally, we assume that for a given E- and I-value we obtain a gene content dataset from the genome dataset with probability 1, and thus omit this probability. Furthermore, for the N I-values and ME-values, we assume an equal prior probability of $\frac{1}{N}$ and $\frac{1}{M}$ respectively. Thus, we can simplify our posterior probability to

$$P(Tree \mid Genome\ data) = \frac{1}{N \times M}\sum_{i}^{N}\sum_{e}^{M} P(Tree \mid gene\ content\ data_{i,e}),$$

where we can see that $P(Tree \mid gene\ content\ data_{i,e})$is our standard phylogenetic posterior probability which we estimated using MCMC sampling. Therefore, we can simply combine all posterior samples for the different MCMC simulations (assuming each MCMC simulation produced the exact same number of samples) with the different orthogroup datasets to compute the total posterior probability of a phylogeny, and similarly, the total posterior consensus tree averaged over all E-values and I-values.

The TPCT was computed both for orthogroup and homogroup datasets for the datasets testing the effect of different combinations of E-value and I-value, resulting in six TPCT in total (3

outgroup samplings and 2 method combinations). The summaries of the orthogroups and homogroups were performed separately and also combined the two types of the datasets into the phylogeny in Figure 2, genome gene content.
