# Supplementary material for: Exploring genome gene content and morphological analysis to test recalcitrant nodes in the animal phylogeny
Source: PLoS One. 2023 Mar 23;18(3):e0282444. doi: 10.1371/journal.pone.0282444 (PMC10035847; doi:10.1371/journal.pone.0282444)
Supplement: S5 File — (PDF) [file pone.0282444.s027.pdf]

1. Supplementary Data 5 - Hypothesis testing

We performed statistical hypothesis testing for three competing hypotheses: (1) Porifera-sister vs Ctenophora-sister, (2) Nephrozoa vs Xenambulacraria hypothesis, and (3) Deuterostome monophyly vs Deuterostome paraphyly. The statistical hypothesis tests provide statistical significance for the various hypotheses. The most commonly used approach in Bayesian statistics is to compute the Bayes factor (BF). BF represents the support in favor of the null model M_0 over the alternative model M_1.

$$BF(M_0, M_1) = \frac{Posterior(M_0)}{Posterior(M_1)} \div \frac{Prior(M_0)}{Prior(M_1)}$$

The BF is often computed as the log-BF instead to avoid numerical imprecision. Standard statistical practice [13], defines a log-BF of larger than 1 as substantial support, larger than 3 as strong support, and larger than 5 as decisive support. Note that [13]) defined significance thresholds for twice the ln(BF): 2ln(BF) > 2 as support, 2ln(BF) > 6 as strong support, and 2ln(BF) > 10 as very strong or decisive support, which are ultimately equivalent to our thresholds (multiplying both sites by a factor of 2). Also, note that positive values of ln(BF) are interpreted as support for the null model M0 and negative values of ln(BF) as support for the alternative model M1.

The Bayes factors have been used to test for topological hypotheses in two ways: (a) as the posterior odds ratio only and (b) as the traditional Bayes factors accounting for the prior odds. The traditional Bayes factors for testing monophyly hypotheses have been criticized as being overly supportive of clades being monophyletic because the prior heavily penalizes against monophyly [14]. For example, adding more species in a clade that is not of interest changes the prior probability of the focal clades, but most likely not the posterior probabilities. In that case, the change in prior probabilities changes the Bayes factors and thus our conclusions, without any actual additional evidence. However, adding or removing species in distant clades from the focal clades should not impact our prior belief about our specific hypothesis, and therefore posterior odds should be preferred over Bayes factors [14].

We obtained the posterior odds from the posterior samples of trees by computing the posterior probability of both null and alternative hypotheses. If the posterior probability was either 1.0 or 0.0, then we subtracted or added $\frac{1}{N}$, where N is the number of MCMC samples,

respectively, to avoid problems when computing the ratio. This approach is conservative (see [15]) and limits our power to compute statistical support only to a maximal precision of $posterior\ odds = N - 1$ or $posterior\ odds = \frac{1}{N-1}$ respectively.

For our first question (Porifera-sister vs Ctenophora-sister) we computed the posterior probability of the clade (Ecdysozoa + Lophotrochozoa + Chordata + Echinodermata + Hemichordata + Xenacoelomorpha + Cnidaria + Placozoa + Ctenophora) being monophyletic (null hypothesis; Porifera-sister) as well as the clade (Ecdysozoa + Lophotrochozoa + Chordata + Echinodermata + Hemichordata + Xenacoelomorpha + Cnidaria + Placozoa + Porifera) being monophyletic (alternative hypothesis; Ctenophora-sister). For our second question (Nephrozoa vs Xenambulacraria) we computed the posterior probability of the clade (Ecdysozoa + Lophotrochozoa + Chordata + Echinodermata + Hemichordata) being monophyletic (null hypothesis; Nephrozoa) as well as the clade (Xenacoelomorpha + Hemichordata + Echinodermata) being monophyletic (alternative hypothesis; Xenambulacraria). For our third question (Deuterostome monophyly vs Deuterostome paraphyly) we computed the posterior probability of the clade Deuterostome being monophyletic (null hypothesis; Deuterostome monophyly) and the clade Deuterostome being paraphyletic (alternative hypothesis; Deuterostome paraphyletic).

We computed the posterior odds separately for each E-value, I-value and MCMC replicate. The posterior probabilities were computed in RevBayes and the posterior odds and posterior odds in a custom R script available in the data repository https://github.com/PalMuc/triangulation/tree/main/Code
