# Supplementary material for: Exploring genome gene content and morphological analysis to test recalcitrant nodes in the animal phylogeny
Source: PLoS One. 2023 Mar 23;18(3):e0282444. doi: 10.1371/journal.pone.0282444 (PMC10035847; doi:10.1371/journal.pone.0282444)
Supplement: S6 File — (PDF) [file pone.0282444.s028.pdf]

1. Supplementary Data 6 - RevBayes scripts parameters

The code used for phylogenetic estimation from the genome gene content data matrices in this study is available in the data repository
https://github.com/PalMuc/triangulation/blob/main/Code/mcmc_gene_content_original.Rev

A full description of the different steps of the code can be found in Pett et al. [16].
The main assumptions of this gene content evolution model are that there are no absent (i.e., no invariant sites) and no singleton sites included. The gene content evolution model was a binary, continuous time reversible Markov chain with estimated stationary frequencies. Additionally, we assume that homogroups or orthogroups can evolve according to one of four discrete rate categories (modeled by the mean of the quartiles of a gamma distribution), which is identical to the among-site rate variation used in nucleotide substitution models. We performed a slight modification to the original model of Pett et al. [16] by fixing the hyperparameter of the branch length prior distribution to an expectation of 0.1, as standardly applied in phylogenetic analyses (see, for example, the program MrBayes [17]).
