# Supplementary material for: Exploring genome gene content and morphological analysis to test recalcitrant nodes in the animal phylogeny
Source: PLoS One. 2023 Mar 23;18(3):e0282444. doi: 10.1371/journal.pone.0282444 (PMC10035847; doi:10.1371/journal.pone.0282444)
Supplement: S7 File — (PDF) [file pone.0282444.s029.pdf]

1. References

1. Goloboff, P. A. *et al.* Phylogenetic analysis of 73 060 taxa corroborates major eukaryotic groups. *Cladistics* vol. 25 211–230 (2009).
2. Peterson, K. J. & Eernisse, D. J. Animal phylogeny and the ancestry of bilaterians: inferences from morphology and 18S rDNA gene sequences. *Evol. Dev.* **3**, 170–205 (2001).
3. Deline, B. *et al.* Evolution of metazoan morphological disparity. *Proc. Natl. Acad. Sci. U. S. A.* **115**, E8909–E8918 (2018).
4. Hibbett, D. S. *et al.* A higher-level phylogenetic classification of the Fungi. *Mycol. Res.* **111**, 509–547 (2007).
5. Carr, M., Leadbeater, B. S. C., Hassan, R., Nelson, M. & Baldauf, S. L. Molecular phylogeny of choanoflagellates, the sister group to Metazoa. *Proc. Natl. Acad. Sci. U. S. A.* **105**, 16641–16646 (2008).
6. Haszprunar, G. Review of data for a morphological look on Xenacoelomorpha (Bilateria incertae sedis). *Org. Divers. Evol.* **16**, 363–389 (2016).
7. Brazeau, M. D. Problematic character coding methods in morphology and their effects. *Biol. J. Linn. Soc. Lond.* **104**, 489–498 (2011).
8. Stach, T. Chordate phylogeny and evolution: a not so simple three-taxon problem. *J. Zool.* **276**, 117–141 (2008).
9. Simion, P. *et al.* A Large and Consistent Phylogenomic Dataset Supports Sponges as the Sister Group to All Other Animals. *Curr. Biol.* **27**, 958–967 (2017).
10. Torruella, G. *et al.* Phylogenetic relationships within the Opisthokonta based on phylogenomic analyses of conserved single-copy protein domains. *Mol. Biol. Evol.* **29**, 531–544 (2012).
11. Posada, D. & Buckley, T. R. Model selection and model averaging in phylogenetics: advantages of akaike information criterion and bayesian approaches over likelihood ratio tests. *Syst. Biol.* **53**, 793–808 (2004).
12. Ashkenazy, H., Sela, I., Levy Karin, E., Landan, G. & Pupko, T. Multiple Sequence Alignment Averaging Improves Phylogeny Reconstruction. *Syst. Biol.* **68**, 117–130 (2019).
13. Kass, R. E. & Raftery, A. E. Bayes Factors. *J. Am. Stat. Assoc.* **90**, 773–795 (1995).
14. Bergsten, J., Nilsson, A. N. & Ronquist, F. Bayesian tests of topology hypotheses with

an example from diving beetles. *Syst. Biol.* **62**, 660–673 (2013).

15. Suchard, M. A., Weiss, R. E. & Sinsheimer, J. S. Models for estimating bayes factors with applications to phylogeny and tests of monophyly. *Biometrics* **61**, 665–673 (2005).
16. Pett, W. *et al.* The Role of Homology and Orthology in the Phylogenomic Analysis of Metazoan Gene Content. *Mol. Biol. Evol.* **36**, 643–649 (2019).
17. Ronquist, F. *et al.* MrBayes 3.2: efficient Bayesian phylogenetic inference and model choice across a large model space. *Syst. Biol.* **61**, 539–542 (2012).
